# Trends of Medicinal Plant Use over the Last 2000 Years in Central Europe

**DOI:** 10.3390/plants12010135

**Published:** 2022-12-27

**Authors:** Maja Dal Cero, Reinhard Saller, Marco Leonti, Caroline S. Weckerle

**Affiliations:** 1 Department of Systematic and Evolutionary Botany, University of Zurich, Zollikerstrasse 107, 8008 Zurich, Switzerland; 2 Albisstrasse 20, 8038 Zurich, Switzerland; 3 Department of Biomedical Sciences, University of Cagliari, Cittadella Universitaria, 09042 Monserrato, Italy

**Keywords:** historical ethnobotany, medicinal plants, Central Europe, traditional use, historical ethnopharmacology

## Abstract

Medicinal plant knowledge in Central Europe can be traced back from the present to antiquity, through written sources. Approximately 100 medicinal plant taxa have a history of continuous use. In this paper, we focus on use patterns over time and the link between historical and traditional uses with the current scientific evidence. We discuss our findings against the backdrop of changing eras and medicinal concepts. Based on use-records from totally 16 historical, popular and scientific herbals, we analyze how use categories of 102 medicinal plant taxa developed over time. Overall, 56 of the 102 taxa maintained continuous use throughout all time periods. For approximately 30% of the continuous uses, scientific evidence supporting their use exists, compared to 11% for recently added uses and 6% for discontinuous uses. Dermatology and gastroenterology are use categories that are relevant across all time periods. They are associated with a high diversity of medicinal taxa and continuously used medicinal species with scientific evidence. Antidotes, apotropaic (protective) magic, and humoral detoxification were important use categories in the past. New applications reflecting biomedical progress and epidemiological challenges are cardiovascular and tonic uses. Changes in medicinal concepts are mirrored in plant use and specifically in changes in the importance of use categories. Our finding supports the concept of social validation of plant uses, i.e., the assumption that longstanding use practice and tradition may suggest efficacy and safety.

## 1. Introduction

Different types of historical studies on medicinal plant use exist. Historical ethnobotanical studies in Europe have been interested in the mechanisms of knowledge transmission, e.g., by Dioscorides and Galen [1,2], or the influence of ancient herbals on recent medicinal plant use, e.g., Tabernaemontanus 16th century [3], Hildegard von Bingen 12th century [4,5], Iatrosophia texts in Cyprus [6,7], Corpus Hippocraticum 5th century BC [8,9], Nordic countries [10], Northeastern Europe 19th century [11], Celtic Provenance Medieval Wales [12] and several Western pharmacopeias [13]. Ancient herbals were also used for extracting information that appears to be relevant for drug discovery programs (e.g., [14,15,16]).

**Figure 1:**
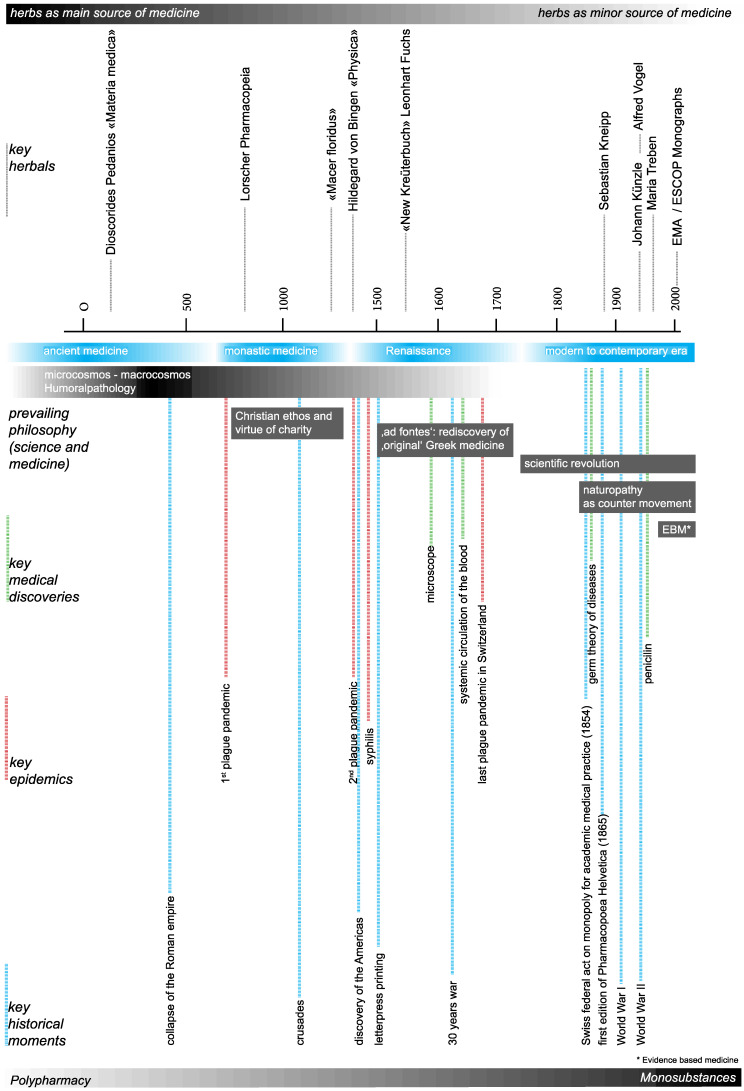
Synchronoptic view of key drivers influencing medicinal plant use.

In this paper, we are interested in patterns in historical and traditional medicinal plant uses and their links with current scientific evidence. We discuss our findings in the context of social validation of medicinal plant uses, which is relevant for assessing the efficacy and safety of traditional herbal remedies in Europe [17,18]. Use patterns are also discussed against the backdrop of changing eras over the last 2000 years. These include epidemiological factors, alterations in philosophical, scientific and medicinal theories, and key medical discoveries and major historical events (Figure 1). For our investigation, we focused on around 100 medicinal plant species which were uninterruptedly used for therapeutic purposes in Central Europe over the last two millennia [19].

The following research questions are addressed: (1) Which are the general trends of medicinal plant use patterns over time? For example, which uses are restricted to specific periods and which are practiced across time? (2) What percentage of continuously used medicinal plants show a link between historical and traditional uses and current scientific evidence?

### Historical Context of Medicinal Plant Use in Central Europe

The history of medicine has been well documented since Antiquity and era-specific changes in the prevailing medical philosophy can easily be traced [20,21,22]. Over almost two millennia, the prevailing medical theory was based on the idea of an analogy between microcosm and macrocosm. This idea originated in ancient Greek philosophy at around the 5th century BC [23]. The theory of humoral pathology arose from this concept and provided a framework for the systematic analysis of complex relationships between humans and their environment. Through Galen’s (ca. 131–201 AD) writings, humoral pathology became the prevailing medical theory until the early 18th century [21].

During medieval times, written knowledge of ancient medicine was retained in Christian monasteries. Old codices were newly compiled, and the Mediterranean *materia medica* was substituted with local species [24]. Ancient predilections and slogans such as “diet over drugs” [8,9,21] are reflected in monastic medicine, e.g., in Hildegard von Bingen’s (1098–1179 AD) *Physica,* where she describes healthy qualities of food plants [25]. Additionally, Christian ethos and charity brought new aspects to medicine and became the drivers for the development of hospitals in Central Europe [21].

During the Renaissance, the ancient sources of medical knowledge were revisited, with an attempt to delete Arabic influences from the texts [21,26]. At the same time, detachment from ancient medical authorities and Christian religion began. The enlightenment movement (18th century) stands for the beginning of modern times and was paralleled by a scientific revolution, resulting in new ideas and theories replacing ancient concepts with an increasingly mechanistic worldview. The reliance on medicinal herbs as the principal resource for multi-target drugs decreased and was largely replaced by the application of mono-substance remedies [27,28].

Thus, since antiquity, the medical landscape of the Old World has been diverse and changeful. Written and institutionalized medicine existed along various forms of oral traditions, which finally resulted in todays’ Central European medical pluralism [26,29,30,31]. In parallel to the scientific revolution leading to biomedicine, naturopathy, as a (health-) political countermovement, arose in the late 18th century [32,33]. This laid the foundation for today’s complementary and alternative medicine, which still considers ancient ideas of bodily humors as so-called ‘constitutional factors’ and the idea that a body in balance prevents sickness. Additionally, ‘blood cleansing’ and detoxifying strategies are still commonly used in popular medicine [30,34].

## 2. Methods

### 2.1. Written Sources

For the present analysis, we reassessed the selection of 24 written documents used in Dal Cero et al. [19], which covered the most important Central European herbals from classical antiquity to Renaissance [35]. The selection of books was based as far as possible on medical texts compiled by doctors and not on recipe collections. It can, therefore, be assumed that there was a practical review and critical appreciation of the texts corresponding to the time of the authors. The distinguished time periods are Antiquity, monastic medicine, Renaissance, and the modern to contemporary era (Table 1). In total, 14 documents were selected, which provided detailed information about the medical uses of 102 taxa that were uninterruptedly used for therapeutic purposes through all time periods. We omitted herbals which did not add new uses [36,37,38,39,40] or did not provide detailed information about the medical use of single species (index of *Capitulare de villis* [41] and index of Lonicero [42]).

For modern and contemporary herbals, we differentiated between (1) popular herbals based on folk medicinal practices and personal experience, and (2) scientific herbals with evidence of efficacy and safety [43]. The choice of modern herbals was largely based on interviews with 61 herbalists, who were asked about the medicinal plant books they use [34]. We did not consider homeopathy [44], anthroposophic medicine [45] and Bach flowers [46]. In addition to the scientific herbals, we used ESCOP [47,48] and EMA Monographs [49] (accessed 2022) to check for scientific evidence of efficacy. Table 1 shows the written sources on which our analysis was based.

Primary data are provided as Supplementary Material.

**Table 1:** Books used for the analysis of use categories.

Time Period	Book Title «Short Title»	Author	First Edition/Edition Used	Abbreviation
Antiquity 1st century CE	*De Materia Medica*	Dioscorides Pedanios from Anazarbos	1st century CE/ Berendes (1902) [50]	DIOS ^1)^
Monastic medicine 8th–12th century	Lorscher Pharmacopoeia	Anonymus	8th century/ Stoll (1992) [24]	LO
	«Macer floridus»	Odo Magdunensis	ca. 1100/ Mayer and Goehl (2001) [51]	MF
	«Physica»	Hildegard von Bingen	ca. 1151 Portmann (1991) [25]	HvB
Renaissance 16th–17th century	«New Kreüterbuch»	Leonhart Fuchs	1543/ Dobat and Dressendörfer (2001) [52]	LF
	«Neuw Kreuterbuch»	Tabernaemontanus; Jacob Theodor	1588/ Edition anno 1625 [53]	TAB ^2)^
Modern to contemporary era since 19th century	Popular herbals ^3)^			
	So sollt ihr leben	Sebastian Kneipp	1889/ Kneipp (2010) [54]	KN
	Das grosse Kräuterheilbuch	Johann Künzle	1945/ Künzle (1945) [55]	JK
	Der kleine Doktor	Alfred Vogel	1952/Vogel (1952) [56]	AV
	Phytothérapie: Traitement des Maladies par les Plantes	Jean Valnet	1983/Valnet (1992) [57]	VAL
	Gesundheit aus der Apotheke Gottes	Maria Treben	1980/Treben (2011) [58]	MT
	Natürlich gesund mit Heilpflanzen	Bruno Vonarburg	1988/Vonarburg (1988) [59]	BVA
	Praxis-Lehrbuch der modernen Heilpflanzenkunde	Ursel Bühring	2005/Bühring (2005) [60]	UB
	Scentific herbals			
	Teedrogen und Phytopharmaka	Max Wichtl (ed.)	1984/Wichtl (2008) [43]	WI
	ESCOP Monographs and supplement	European Scientific Cooperative of Phytotherapy	2003 and 2009 [47,48]	ESCOP
	EMA Monographs	Committee on Herbal Medicinal Products (HMPC)	Webpages 1995 –2022/accessed Oct. 2022 [49]	EMA

^1)^ We used the modern translation of Dioscorides’ *De Materia Medica* from Berendes (1902) [50] as a surrogate for earlier Dioscorides translations. We crosschecked for ethnotaxa with Matthioli (1568) [61] as one of the most widespread Renaissance translations of Dioscorides’ *De Materia Medica* [2]. ^2)^ For those species not included in Leonhart Fuchs (*Acorus calamus, Malus sylvestris,* and *Pyrus communis*) we consulted the herbal of Tabernaemontanus (1625) [53]. ^3)^ These popular herbals are the sources of information for herbalists in Switzerland at present (cf. [34]).

### 2.2. Use-Records and Use Categories

For the analysis, we recorded each documentation of a specific taxon for a specific use as one use-record. All uses were grouped into 18 use categories related to organs, symptoms and route of administration (Table 2). The categories follow [2] and [34]. To match historical uses with modern use categories, we consulted Hoefler (1899) [62].

We considered a use category with scientific evidence when the use category appeared to be validated for a specific taxon in either Wichtl (2008) [43], ESCOP [47,48] or EMA [49].

### 2.3. Medicinal Plant Taxa

For species identification, we relied on recent editions of ancient, monastic and Renaissance herbals, which include Latin names of the plants (Table 1). For a few species, which were not mentioned in the recent edition of Fuchs’ «New Kreüterbuch», we relied on the original plant list of Tabernaemontanus (1588) [53]. All of these taxa were easily identifiable, such as, *Acorus calamus*.

Taxonomically, this study was based on the ‘Flora indicativa’ [63] which covers plants of the Swiss flora and the Alps. For several species, we used species complexes (aggregates, agg.) [63]. These aggregates comprise closely related Swiss and Alpine species and tend to reflect so-called ethnotaxa, i.e., species with identical or similar local names and uses. The following adjustments were made with respect to Dal Cero et al. (2014) [19]: we added *Helleborus* spp., *Peucedanum* spp., *Teucrium* spp., *Salvia* spp. and *Urtica* spp. as ethnotaxa. Different species of these genera, also as local substitutes for Mediterranean species, have been used since Antiquity. In addition, we merged the following species into ethnotaxa as they have been used interchangeably in one or several time periods: *Abies alba* and *Larix decidua* (*Abies* spp.), *Lepidium officinale* and *Nasturtium officinale* (*Lepidium* spp.), *Matricaria chamomilla* and *Anthemis* spp. (*Matricaria chamomilla*), *Mercurialis annua* and *M. perennis* (*Mercurialis* spp.), *Prunus avium*, *P. domestica* and *P. spinosa* (*Prunus* spp.), *Sambucus nigra* and *Sambucus ebulus* (*Sambucus* spp.), *Sinapis alba* and *Brassica nigra* (*Sinapis* spp.), as well as *Solanum nigrum* and *Solanum dulcamara* (*Solanum* spp.). In total, we analysed 102 taxa (species, aggregates and ethnotaxa). Accordingly, we used the term ‘plant taxa’ instead of ‘plant species’. Nomenclature follows Plants of the World Online [64], and the APG system [65].

### 2.4. Analysis of Data and Diachronic Patterns

Diachronic patterns were analyzed from the perspective of (1) use categories, i.e., diversity of medicinal taxa over time per use categories; and (2) medicinal taxa, i.e., diversity of use categories over time per taxon. In addition, typical diachronic patterns were highlighted with the example of a few medicinal taxa.

Descriptive statistics (mean ± standard deviation) was used to describe changes in taxa per use category and use categories per taxa.

### 2.5. Abbreviations

UCatUse categoryUCat_const_Use category constant since AntiquityUCat_recent_Use category added in contemporary periodURUse-record

## 3. Results

### 3.1. Use-Records Per Time Period

In total, 3993 use-records were found for the102 medicinal plant taxa: Antiquity 891 use-records, monastic medicine 677 UR, Renaissance 1036 UR, modern to contemporary era 1154 UR from popular herbals, and 235 UR for 53 taxa from scientific herbals.

### 3.2. Diachronic Changes: The Use Category Perspective

**Figure 2:**
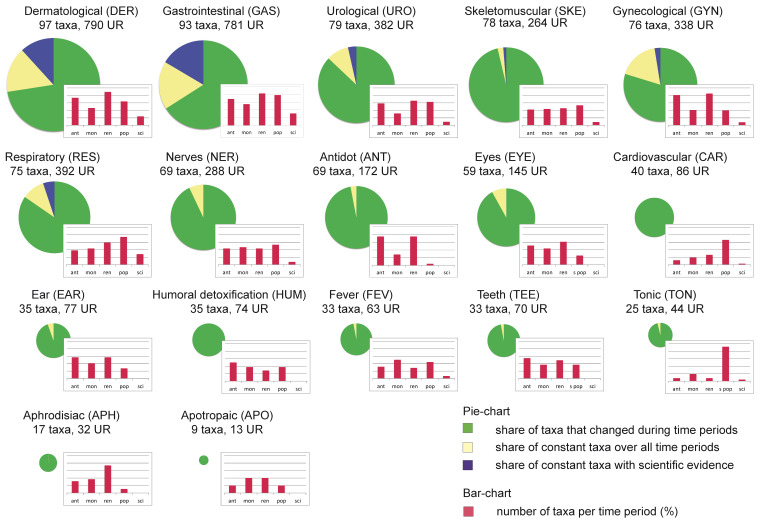
Pie charts show the total number of taxa used for a specific use category. Bar charts show the percentage of total taxa used in different eras.

The plant taxa used for specific use categories change over time. The share of taxa per use-category utilized uninterruptedly across all time periods ranges between 0–29% (Figure 2, pie charts). The highest numbers of taxa constantly used across all time periods were found for categories GAS (33 taxa), DER (28), GYN (16), RES (12), and URO (11). For the categories GAS, DER and RES, the highest percentage was found for constantly used taxa with scientific evidence (GAS: constantly used 33 taxa [34%], 16 taxa with scientific evidence; DER: constantly used 28 taxa [27.5%], 12 taxa with scientific evidence; RES constantly used 12 taxa [15.5%], 4 taxa with scientific evidence).

A steady increase is observable over time in the number of taxa used for the categories RES, CAR and TON (Figure 2, bar charts). Taxa used for cardiovascular problems increased from five in Antiquity, to eight in monastic medicine, 11 in the Renaissance period to 28 in modern and contemporary herbals, but not one single taxon was used through all time periods. TON is associated with a higher number of taxa in contemporary popular herbals (22 taxa, e.g., *Avena sativa, Origanum vulgare, Thymus vulgaris, Urtica dioica*), whereas in Antiquity only *Artemisia absinthium* and *Ficus carica* were considered as general tonics. The concept of antidots was important until the Renaissance, with 14 documented taxa since Antiquity, whereas in contemporary herbals, this indication is only documented for *Allium sativum* and *Ruta graveolens*.

### 3.3. Diachronic Changes: The Medicinal Taxon Perspective

Table 3 provides an overview of use categories per taxa over time. In 129 cases (12.6% of all possible cases, i.e., all use categories across all taxa), use categories for a specific taxon remained constant since Antiquity (Table 3, black fields; 1283 UR; found among 56 taxa). For 31.8% of these constant use categories, scientific evidence exists (Table 3, black field with white x; 41 cases, 93 UR). This includes, e.g., *Achillea millefolium* for DER, *Allium sativum* for RES, *Artemisia absinthium* for GAS, *Foeniculum vulgare* for GAS and GYN, *Urtica dioica* for SKE. Taxa with high numbers of constant use categories since Antiquity are: *Urtica dioica* (6 UCat_const_ with 102 UR), *Ruta graveolens* (8 UCat_const_, 88 UR), *Artemisia absinthium* (6 UCat_const_, 67 UR), *Allium sativum* (5 UCat_const_, 61 UR), *Rosa* spp. (5 UCat_conat_, 55 UR), *Thymus* spp. (5 UCat_const_, 54 UR), and *Matricaria chamomilla* (5 UCat_const_, 51 UR).

In 159 cases, specific use categories occurred for the first time in contemporary popular herbals (Table 3, grey boxes; 14.1% of total cases; 301 UR; 31 taxa). For 11.3% of these recent use categories, scientific evidence exists (Table 3, gray field with white x; 13 cases, 28 UR). The following species show relatively high numbers of recent use categories: *Achillea millefolium* (7 UCat_recent_, 33 UR), *Sambucus nigra* (5 UCat_recent_, 18 UR), *Valeriana officinalis* (4 UCat_recent_, 21 UR), *Salix alba* (3 UCat_recent_, 13 UR) and *Hypericum perforatum* (3 UCat_recent_, 11 UR).

In 745 cases (73.3% of all cases; 2174 UR), use categories of a specific taxon were documented in one or several time periods, but without continuity (Table 3, light grey fields). For 6.5% of these categories, scientific evidence exists (Table 3, light grey fields with white x; 48 cases; 104 UR).

The average number of total use categories per taxon over all time periods is 10.1 ± 2.9. Per time period, the average number of use categories per taxon is 5.2 ± 2.2. This varies from 11.3 ± 1.3 (*Ruta graveolens*), with a total of 14 use categories over all eras, to 1.0 ± 0.0 (*Colchicum autumnale*), with a total of 2 use categories over all eras. Other species with many use categories are, for example: *Artemisia absinthium* (11.0 ± 2.2, total 16 UCat), *Allium sativum* (10.5 ± 2.5, total 17 UCat)*, Allium cepa* (9.8 ± 2.5, total 15 UCat)*, Urtica dioica* (9.3 ± 1.3, total 11 UCat) and *Rosa* spp. (9.3 ± 2.6, total 16 UCat).

Few use categories were found for, e.g., *Clematis vitalba* (2.3 ± 1.3, total 7 UCat over all eras)*, Cannabis sativa* (2.3 ± 1.9, total 6 UCat)*, Conium maculatum* (2.3 ± 1.9, total 7 UCat), *Onopordum acanthium* (2.3 ± 2.9, total 8 UCat)*, Pyrus communis* (2.0 ± 1.2, total 6 UCat)*, Euphorbia esula* (1.8 ± 1.0, total 5 UCat), and *Secale cereale* (1.3 ± 0.6, total 3 UCat).

### 3.4. Diachronic Patterns at the Example of Specific Taxa

**Figure 3:**
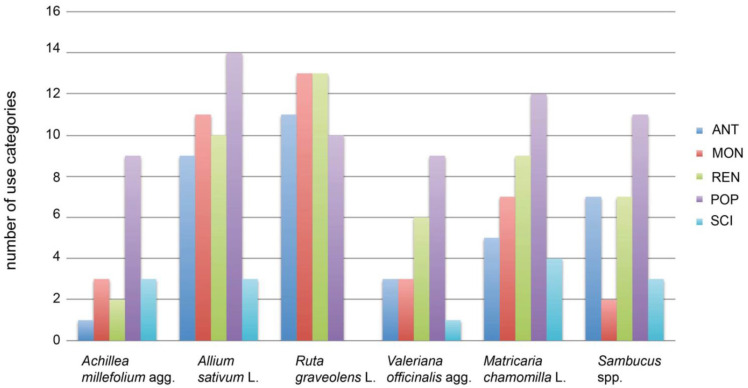
Number of use categories for 6 species during different eras, showing different diachronic trends.

Relatively few use categories were documented through time, but many new categories in modern and contemporary era were found for, e.g., *Achillea millefolium* (1 UCat_const_, 26 UR; 7 UCat_recent_, 33 UR; Table 3 and Figure 3). *Achillea millefolium* was broadly used, with a total of 11 use categories over all time periods. Only dermatological uses have been stable since antiquity and are also documented in the EMA Monograph (2020) [62,63]. In the contemporary era, seven use categories, CAR, GAS, RES, SKE, TEE, TON, and URO, were added. For application in GAS, scientific evidence exists. GYN was documented since the Renaissance and is backed by scientific evidence (EMA Monograph 2020) [66,67].

High numbers of use categories through all or several time periods were observed for, e.g., *Allium sativum* (17 UCat, 107 UR); FEV is the only category in which *Allium sativum* was never documented. ANT, DER, GAS, RES remained stable during all time periods and, for RES, scientific evidence exists.

Use categories remained stable over all or several time periods and no additional use categories occurred during the modern and contemporary era for *Ruta graveolens* (14 UCat, 107 UR). *Ruta graveolens* is an example for a species with a very constant use over time. Eight out of 14 categories remained stable, including ANT, DER, EAR, EYE, GAS, GYN, NER, RES (88 UR of 115 UR). However, there is no scientific evidence for any of the uses.

No stable use category existed over time, but a use category added in the modern and contemporary era, backed by scientific evidence, was found for *Valeriana officinalis* (11 UCat, 50 UR; 0 UCat_const_; 3 UCat_recent_, 26 UR). *Valeriana officinalis* was broadly used but without any constant use over time. Contemporary popular herbals added four categories, CAR, GAS, NER and RES, where NER has scientific evidence.

A high number of stable use categories over all time periods plus scientific evidence can be observed for, e.g., *Matricaria chamomilla* (5 UCat_const,_ 74 UR); DER, GAS and GYN (58 UR of 100 UR) remained stable over time and are backed by scientific evidence. RES was added in the contemporary era and is also sustained by scientific evidence (EMA-monograph 2015) [68].

## 4. Discussion

### 4.1. Medicinal Plant Use Patterns over Time

While some medicinal plants were constantly used for the same reason over the last two millennia, others have a changing use history. A general diversification or decrease in uses over time does not exist; instead, different use trends occur for different species.

More than half of the analyzed taxa (56 out of 102) show specific use categories that were continuously recommended through all time periods. This adds to 12.6% of use categories across all taxa and stands for a body of medicinal plant knowledge and uses continuously practiced in Central Europe over the last two millennia [9].

Changes in medicinal concepts are mirrored in plant use, and specifically in the changing importance of use categories. While some categories are heavily bound to specific medicinal concepts, others remain stable, independently of changing eras and worldviews. For example, dermatology (DER) and gastroenterology (GAS) are use categories that were relevant across all periods, with high species diversity, and a high share of constantly used species sustained by scientific evidence. These use categories also figure prominently in neighboring Mediterranean medicinal floras [2,6], as well as medicinal floras from all over the world, e.g., [69,70,71]. Obviously, the universal need for effective GAS and DER treatments is largely independent of medicinal concepts and time periods.

Other categories were more susceptible to change. For example, antidotes (ANT), apotropaic magic (APO) and humoral detoxification (HUM) were important use categories in the past, but rarely play a role in contemporary herbals. Instead, new applications reflecting scientific progress and epidemiological challenges arose, such as cardiovascular (CAR) and tonic (TON) uses. The anatomic understanding of blood circulation in the 17th century fueled uses for cardiovascular disorders. At present, they are prominently found in popular herbals, as cardiovascular diseases are among the most common causes of death in Central Europe [72]. Some of the plants used for cardiovascular applications do not directly influence heart activity, but rather have a relaxant and stress reducing effect (e.g., *Melissa officinalis, Rosa* spp.). It is thus little surprising that they were formerly used for nerves (NER) and only recently became important for cardiovascular problems.

Furthermore, the humoral (HUM) applications of the past seem to be replaced by tonic (TON) applications in more recent times. Interestingly, the general purpose of a ‘tonic’, namely, to restore and maintain physiological functioning of an organ system, largely corresponds with the circumscription of humoral detoxification according to the theory of four humors [21,73]. Plants that are used for both categories, HUM and TON, such as, e.g., *Artemisia absinthium* and *Urtica dioica*, usually support digestion and/or have a diuretic effect [74,75].

### 4.2. Link between Historical and Traditional Uses of Taxa and Scientific Evidence

Approximately 30% of the continuous uses have scientific evidence, compared to 11% among recently added uses and 6% among the discontinuous uses.

This finding seems to support the concept of the social validation of specific plant uses, i.e., the assumption that longstanding use practice suggests efficacy and safety [17,76]. In many European countries, it is possible to register traditionally used medicinal plants as *Traditional Herbal Medicinal Products* (Directive 2004/24/EC) [77]. As a proof of traditional use, an uninterrupted use of the product for at least 30 years, 15 of which in the European Union, is required. From an ethnological and historical perspective, this time period does not adequately represent the multifaceted concept of tradition [17]. In particular, products that have been "forgotten" cannot be reintroduced under the concept of tradition. The present data may be used as a resource for traditional herbal medicinal products.

All plants in Table 3 were scientifically investigated to different degrees, but not necessarily tested for specific use categories. Some are considered toxic and, therefore, no longer recommended, such as, e.g., *Tussilago farfara* (pyrrolizidine alkaloids) or *Arum maculatum* (oxalate needles, saponins). For half of the plants, a monograph of Commission E (predecessor of HMPC and EMA) exists. In the case of *Iris germanica* agg., according to Commission E, clinical efficacy was not proven. Since the EMA monographs are prepared in a regulatory context for simplified approvals based on traditional or well-established use, the listed areas of applications are often very narrow. Therefore, many use categories have not been investigated, and the abovementioned 30% cases of continuous use with scientific evidence can be seen as a conservative estimate.

### 4.3. Diversity of Diachronic Use Patterns Exemplified by Specific Taxa

*Allium sativum* has been used for all categories throughout time but fever (17 out of 18 UCat). Since antiquity, *Allium sativum* was seen as both a medicine and food [8]. This might be one of the reasons for its very broad use. Its blood-thinning properties have been documented during Renaissance [52] and, since the early 20th century, its popularity increased as pharmacological and clinical studies showed cardiotonic and anti-atherosclerotic effects [78,79].

A feedback loop and mutual impact of scientific discoveries and local popular knowledge can be assumed for *Valeriana officinalis* and its prominent contemporary use as ‘nervinum’ (neurotonic) [80,81]. The common use of *Valeriana officinalis* as a sedative at present has been known since the late middle-ages [24]. However, broader acceptance only came with pharmaceutical studies in the late 19th century [82].

The use history of *Ruta graveolens* in the Mediterranean is impressive. Gynecological and respiratory uses have been documented in the Hippocratic corpus but dermatological uses and the uses for swollen spleen are also very old [83]. These ancient uses are still practiced in the Mediterranean [2,6]. *Ruta graveolens* is also described in Central European popular herbals. However, at least for Switzerland, there is little evidence of its current use, although the plant is cultivated in gardens [34,84,85,86,87,88]. It seems that *Ruta graveolens* never fully arrived in Central European medicinal practice but is instead a Mediterranean relict.

Both *Achillea millefolium* and *Matricaria chamomilla* are very popular in modern and contemporary times. *Matricaria chamomilla* is by far the most-used medicinal plant among laypeople and experts [34,89]. Abundant phytopharmacological and clinical studies show scientific evidence for use categories documented since antiquity, such as dermatological- (DER), gastroenterological- (GAS), gynecological- (GYN) and respiratory (RES) applications [90,91]. In contrast, *Achillea millefolium* shows a broad expansion of uses in modern and contemporary popular herbals. An expansion of uses is visible for many of the 102 taxa used over the last two millennia and probably reflects an intensive exchange among different cultures and schools of knowledge related to the medicinal landscape.

## 5. Conclusions

Diachronic insight into medicinal plant use over two millennia highlights changes in specific use categories, which are in line with changes in medicinal concepts, pharmaceutical technologies and new needs. Many medicinal plants show a general extension of uses over time. However, a constant body of specific uses over time for a number of taxa was also identified. These medicinal plants are used in the same way as in Antiquity, monastic medicine and the Renaissance, regardless of basic changes in medicinal concepts and technological development. Overall, they show the highest share of scientific evidence, which supports the concept of social validation, stressing that longstanding use practice may suggest efficacy and safety. With our results, we present a historically based dataset that can be used as source of traditional plant use in a regulatory context. A more detailed look into use patterns through the consideration of herbal drugs and their mode of preparation would deepen our understanding of the linkage between traditional uses, scientific evidence, and the concept of social validation.

## Figures and Tables

**Table 2:** Use categories related to organs and symptoms.

Abbreviation	Organ/Symptom	Notes
ANT	Antidote	bites and stings of poisonous and mad animals, intoxication
APH	Aphrodisiac	and anaphrodisiac
APO	Apotropaic	against ‘bad influence’ and ailments [no internal use], charms
CAR	Cardiovascular	blood circulation, heart diseases, systemic applications for hemorrhoids and veins
DER	Dermatological	skin, wounds, ulcers, topic applications for hemorrhoids and veins
EAR	Ear	ear infections, deafness
EYE	Ophthalmic	eye infections, blindness
FEV	Fever	including malaria
GAS	Gastrointestinal	digestion, stomachache, diarrhea, icterus
GYN	Gynecological	menstrual problems, perinatal
HUM ^1)^	Humoral detoxification	general indication for purification and detoxification
NER	Nerves	sleeplessness, nervousness, general analgesics
RES	Respiratory	cough, lungs
SKE	Skeletomuscular	musculoskeletal pain and disability, rheumatism, injuries
TEE	Teeth	toothache
TON ^2)^	Tonic	general strengthening, immunomodulatory, roborants, anemia
URO	Urological	bladder, kidney disease
VAR	Varia	including anti-inflammatory, blood, cancer, diabetes, diet, metabolic disorders, parasites, spleen

^1)^ ‘humoral detoxification’ is used only for general detoxifying indications without a link to diuretic (->URO) or laxative effects (->GAS), mainly for ‘removing of bad humors’ (blood, cholera, phlegm), in the sense of the ancient theory of the four humors and humoral pathology. ^2)^ ‘tonic’ is used in a strict sense and all indications with a link to appetite and digestion (e.g., orexygenic) are allocated in GAS; indications with a link to fatigue or nervous exhaustion are allocated in NER.

**Table 3:** Use categories per taxa documented over the last two millennia. black field: category occurs through all time periods; grey field: category occurs in contemporary era only; light grey: category occurs in several time periods; X: scientific evidence.

	Use Categories per Taxon per Era [Mean ± Sd]	Min per Era	Max per Era	ANT (Antidote)	APH (Aphrodisiac)	APO (Apotropaic)	CAR (Cardiovascular)	DER (Dermatological)	EAR (Ear)	EYE (Ophthalmic)	FEV (Fever)	GAS (Gastrointestinal)	GYN (Gynecological)	HUM (Humoral detoxification)	NER (Nerves)	RES (Respiratory)	SKE (Skeletomuscular)	TEE (Teeth)	TON (Tonic)	URO (Urological)	VAR (Varia)	Total Use-Records
*Abies* spp.	6.0 ± 2.5	4	9																			38
*Achillea millefolium* agg.	3.8 ± 3.6	1	9					**X**				**X**	**X**									67
*Acorus calamus* L.	4.3 ± 2.5	1	7					**X**				**X**										30
*Adiantum capillus-veneris* L.	4.3 ± 3.2	1	7																			21
*Agrimonia eupatoria* L.	4.5 ± 3.2	3	9					**X**				**X**				**X**						30
*Allium cepa* L.	9.8 ± 2.5	7	13																			72
*Allium sativum* L.	10.5 ± 2.5	8	14				**X**									**X**	**X**					104
*Althaea officinalis* L.	7.0 ± 1.8	5	9					**X**				**X**	**X**			**X**						63
*Anagallis arvensis* agg.	5.3 ± 3.0	1	6																			22
*Anethum graveolens* L.	5.5 ± 1.7	4	7																			30
*Arctium lappa* agg.	3.0 ± 1.0	2	4					**X**												**X**		21
*Artemisia abrotanum* L.	7.5 ± 2.5	4	10																			36
*Artemisia absinthium* L.	11.0 ± 2.2	9	14									**X**										96
*Arum maculatum* agg.	3.0 ± 1.2	2	4																			22
*Asarum europaeum* agg.	5.0 ± 3.4	1	9																			25
*Avena sativa* agg.	4.8 ± 4.2	2	11					**X**														34
*Beta vulgaris* L.	5.0 ± 2.7	1	7																			23
*Cannabis sativa* L.	2.3 ± 1.9	1	5																			12
*Capsella bursa-pastoris* agg.	3.5 ± 2.5	1	7					**X**					**X**									33
*Carum carvi* L.	3.3 ± 2.6	1	7									**X**										28
*Chelidonium majus* L.	4.5 ± 2.4	3	8									**X**										27
*Cichorium intybus* L.	5.0 ± 2.2	2	7									**X**										34
*Clematis vitalba* L.	2.3 ± 1.3	1	4																			9
*Colchicum autumnale* agg.	1.0 ± 0.0	1	1																			5
*Conium maculatum* L.	2.3 ± 1.9	1	5																			11
*Convallaria* spp.	2.5 ± 1.7	1	5																			13
*Coriandrum sativum* L.	2.8 ± 1.5	1	4									**X**										18
*Corylus avellana* L.	3.0 ± 1.4	2	5																			14
*Crocus sativus* L.	4.5 ± 4.0	1	8																			23
*Cucurbita pepo* L.	3.8 ± 2.2	1	6																	**X**		30
*Cydonia oblonga* Mill.	4.2 ± 2.2	2	5																			29
*Daucus carota* L.	4.8 ± 3.0	1	8																			35
*Eryngium campestre* L.	4.0 ± 2.9	1	7																			19
*Euphorbia esula* agg.	1.8 ± 1.0	1	3																			12
*Ficus carica* L.	7.3 ± 4.5	2	11																			66
*Foeniculum vulgare* agg.	7.0 ± 2.5	5	10									**X**	**X**			**X**						76
*Fumaria officinalis* agg.	2.8 ± 1.3	1	4									**X**										22
*Gentiana lutea* agg.	4.0 ± 2.3	2	6									**X**							**X**			28
*Hedera helix L.*	6.0 ± 4.1	1	9													**X**						34
*Helleborus* spp.	6.0 ± 4.2	1	10																			32
*Heracleum sphondylium* agg.	5.3 ± 3.1	1	8																			25
*Hordeum vulgare* agg.	3.8 ± 2.5	1	7																			32
*Hyoscyamus niger* L.	8.0 ± 1.8	6	10																			41
*Hypericum perforatum* agg.	3.6 ± 3.1	1	8					**X**				**X**			**X**							52
*Inula helenium* L.	5.8 ± 1.6	4	7																			30
*Iris germanica* agg.	7.5 ± 1.5	1	11																			55
*Juglans regia* L.	5.3 ± 1.9	4	8					**X**														48
*Juniperus communis* agg.	6.3 ± 2.2	3	8									**X**					**X**					70
*Juniperus sabina* L.	2.8 ± 1.0	2	4																			23
*Lepidium* spp.	7.5 ± 3.0	5	11									**X**				**X**						51
*Levisticum officinale* L.	5.8 ± 1.7	4	8																	**X**		38
*Linum usitatissimum* agg.	4.0 ± 2.0	1	5					**X**				**X**										38
*Malus sylvestris* agg.	6.2 ± 2.5	1	7																			23
*Malva sylvestris* agg.	8.0 ± 2.0	7	11									**X**				**x**						68
*Marrubium vulgare* L.	6.8 ± 1.0	6	8									**X**				**X**						57
*Matricaria chamomilla* L.	7.8 ± 2.5	5	11					**X**				**X**	**X**			**X**						73
*Melissa officinalis* L.	7.8 ± 0.5	7	8					**X**				**X**			**X**							65
*Mentha pulegium* L.	8.0 ± 1.0	2	11																			48
*Mentha spicata* agg.	8.3 ± 0.5	8	9					**X**				**X**					**X**					86
*Mercurialis* spp.	2.5 ± 2.1	1	5																			11
*Meum athamanticum* Jacq.	3.3 ± 1.0	1	5																			18
*Morus nigra* L.	3.5 ± 1.0	2	5																			23
*Ocimum basilicum* L.	5.5 ± 3.5	2	8																			29
*Onopordum acanthium* L.	2.3 ± 2.9	1	6																			11
*Origanum vulgare* agg.	7.3 ± 5.3	1	11																			45
*Papaver somniferum* L.	7.0 ± 0.6	3	9																			49
*Petasites hybridus* (L.) P. Gaertn.	4.5 ± 4.1	1	9									**X**				**X**				**X**		33
*Petroselinum crispum* (Mill.) Fuss	4.3 ± 1.0	4	5									**X**								**X**	**X**	30
*Peucedanum* spp.	8.3 ± 1.5	6	10																			52
*Pimpinella saxifraga* agg.	3.0 ± 2.3	1	5													**X**						20
*Polygonum aviculare* agg.	5.0 ± 3.6	1	8																			34
*Polypodium vulgare* L.	2.8 ± 1.3	3	9									**X**				**X**						17
*Potentilla* spp.	5.8 ± 2.8	3	9					**X**				**X**										43
*Prunus* spp.	4.5 ± 1.5	2	8																			37
*Pyrus communis* agg.	2.0 ± 1.2	1	3																			10
*Quercus robur* agg.	4.0 ± 2.2	1	6					**X**				**X**										36
*Raphanus sativus* L.	5.3 ± 3.3	2	9																			36
*Rosa* spp.	9.3 ± 2.6	7	13					**X**								**X**	**X**				**X**	91
*Rubia tinctorum* L.	4.3 ± 3.2	1	7																			20
*Rubus idaeus* L.	3.3 ± 2.1	1	5					**X**				**X**	**X**									23
*Rumx* spp.	6.3 ± 2.2	4	7																			41
*Ruta graveolens* L.	11.5 ± 1.3	10	13																			114
*Salix alba* agg.	4.8 ± 2.5	1	6								**X**				**X**		**X**					41
*Salvia officinalis* agg.	8.0 ± 2.9	4	11					**X**			**X**	**X**										78
*Sambucus nigra* L.	6.8 ± 2.9	3	10									**X**				**X**				**X**		62
*Saponaria officinalis* L.	5.8 ± 2.6	3	8													**X**						34
*Secale cereale*	1.3 ± 0.6	1	2																			6
*Sinapis* spp.	7.5 ± 4.2	2	12													**X**					**X**	49
*Solanum* spp.	4.5 ± 1.0	4	6					**X**														24
*Symphytum officinale* agg.	3.3 ± 2.2	1	6					**X**				**X**					**X**					46
*Teucrium* spp.	5.0 ± 4.2	1	9																			31
*Thymus* spp.	8.8 ± 1.3	7	9					**X**								**X**						87
*Triticum aestivum* agg.	4.0 ± 2.0	1	7																			36
*Tussilago farfara* L.	2.6 ± 2.2	1	6													**X**						26
*Urtica dioica* L.	9.3 ± 1.3	8	11					**X**									**X**			**X**		127
*Valeriana officinalis* agg.	4.8 ± 3.3	1	9												**X**							44
*Veratrum album* agg.	4.8 ± 3.8	2	10																			24
*Verbascum thapsus* agg.	5.5 ± 2.5	2	8													**X**						40
*Verbena officinalis* L.	6.8 ± 2.1	6	11													**X**						60
*Vinca minor* L.	3.0 ± 1.7	1	4																			18
*Viola hirta* agg.	6.8 ± 2.5	4	10					**X**														56
*Vitis vinifera* agg.	5.8 ± 3.3	1	8					**X**														39

## Data Availability

Not applicable.

## Supplementary Materials

The following supporting information can be downloaded at: https://www.mdpi.com/article/10.3390/plants12010135/s1.

## References

[1] Leonti M., Staub P.O., Cabras S., Castellanos M.E., Casu L. (2015). From cumulative cultural transmission to evidence-based medicine: Evolution of medicinal plant knowledge in Southern Italy. Front. Pharmacol..

[2] Leonti M., Cabras S., Weckerle C.S., Solinas M.N., Casu L. (2010). The causal dependence of present plant knowledge on herbals–Contemporary medicinal plant use in Campania (Italy) compared to Matthioli (1568). J. Ethnopharmacol..

[3] Clair S. (2011). Die Kräutermedizin des Renaissance-Arztes Tabernaemontanus (16. Jh.) und Phytotherapie heute—Was ist geblieben, was hat sich verändert?. Schweiz. Z. Ganzheitsmed..

[4] Uehleke B., Hopfenmueller W., Stange R., Saller R. (2012). Are the Correct Herbal Claims by Hildegard von Bingen Only Lucky Strikes? A New Statistical Approach. Complement. Med. Res..

[5] Mayer-Nicolai C. (2008). Vergleich der Durch die Historischen Autoren Hildegard von Bingen und Leonhart Fuchs Pflanzlichen Arzneimitteln Zugeschriebenen mit Aktuell Anerkannten Indikationen.

[6] Lardos A., Prieto-Garcia J., Heinrich M. (2011). Resins and gums in historical *iatrosophia* texts from Cyprus–a botanical and medico-pharmacological approach. Front. Pharmacol..

[7] Lardos A., Heinrich M. (2013). Continuity and change in medicinal plant use: The example of monasteries on Cyprus and historical *iatrosophia* texts. J. Ethnopharmacol..

[8] Totelin L. (2015). When foods become remedies in ancient Greece: The curious case of garlic and other substances. J. Ethnopharmacol..

[9] Touwaide A., Appetiti E. (2015). Food and medicines in the Mediterranean tradition. A systematic analysis of the earliest extant body of textual evidence. J. Ethnopharmacol..

[10] Teixidor-Toneu I., Kool A., Greenhill S.J., Kjesrud K., Sandstedt J.J., Manzanilla V., Jordan F.M. (2021). Historical, archaeological and linguistic evidence test the phylogenetic inference of Viking-Age plant use. Philos. Trans. R. Soc. Lond. B Biol. Sci..

[11] Prakofjewa J., Anegg M., Kalle R., Simanova A., Prūse B., Pieroni A., Sõukand R. (2022). Diverse in Local, Overlapping in Official Medical Botany: Critical Analysis of Medicinal Plant Records from the Historic Regions of Livonia and Courland in Northeast Europe, 1829–1895. Plants.

[12] Wagner C., De Gezelle J., Komarnytsky S. (2020). Celtic Provenance in Traditional Herbal Medicine of Medieval Wales and Classical Antiquity. Front. Pharmacol..

[13] Vos P. (2010). European materia medica in historical texts: Longevity of a tradition and implications for future use. J. Ethnopharmacol..

[14] Kong L.Y., Tan R.X. (2015). Artemisinin, a miracle of traditional Chinese medicine. Nat. Prod. Rep..

[15] Adams M., Berset C., Kessler M. (2009). Medicinal herbs for the treatment of rheumatic disorders—A survey of European herbals from the 16th and 17th century. J. Ethnopharmacol..

[16] Adams M., Gmünder F., Hamburger M. (2007). Plants traditionally used in age related brain disorders—A survey of ethnobotanical literature. J. Ethnopharmacol..

[17] Jütte R., Heinrich M., Helmstädter A., Langhorst J., Menge G., Niebling W., Pommerening T., Tramisch H.J. (2017). Herbal medicinal products—Evidence and tradition from a historical perspective. J. Ethnopharmacol..

[18] Helmstädter A., Staiger C. (2014). Traditional use of medicinal agents: A valid source of evidence. Drug Discov. Today.

[19] Dal Cero M., Saller R., Weckerle C.S. (2014). The use of the local flora in Switzerland: A comparison of past and recent medicinal plant knowledge. J. Ethnopharmacol..

[20] Ackerknecht E.H. (1970). Therapie von den Primitiven bis zum 20 Jahrhundert.

[21] Porter R. (1997). The Greatest Benefit to Mankind: A Medical History of Humanity.

[22] Eckart W.U. (2000). Geschichte der Medizin.

[23] Rothschuh K.E. (1962). Idee und Methode in ihrer Bedeutung für die geschichtliche Entwicklung der Physiologie. Sudhoffs Arch. Z. Für Wiss..

[24] Stoll U. (1992). Das “Lorscher Arzneibuch”; Ein medizinisches Kompendium des 8. Jahrhunderts (Codex Bambergensis Medicinalis 1). Sudhoffs Arch. Z. Für Wiss..

[25] Portmann L. (1991). Hildegard von Bingen; Heilkraft der Natur: “Physica”.

[26] Bruchhausen W., Schott H. (2008). Geschichte, Theorie und Ethik der Medizin.

[27] Gertsch J. (2011). Botanical Drug, Synergy, and Network Pharmacology: Forth and Back to Intelligent Mixtures. Planta Med..

[28] Ledermann F. (2001). Von Nephentes zur modernen Pharmakognosie; ein kurzer Abriss der Geschichte der Phytotherapie. Schweiz. Med. Z. Phytother..

[29] Jenny M., Sharma R. (2009). Heilerinnen und Heiler in der Deutschschweiz: Magnetopathen, Gebetsheiler, Einrenker.

[30] Glaser C. (2006). Das Sachranger Rezeptbuch.

[31] Hoefert H.W., Uehleke B. (2009). Komplementäre Heilverfahren im Gesundheitswesen. Analyse und Bewertung.

[32] Wolff E. (2014). Volksmedizin. In *Historisches Lexikon der Schweiz*.

[33] Roth S. (1991). Im Streit um Heilwissen, Zürcher Naturheilvereine Anfangs des 20. Jahrhunderts.

[34] Dal Cero M., Saller R., Weckerle C.S. (2015). Herbalists of Today’s Switzerland and Their Plant Knowledge. A Preliminary Analysis from an Ethnobotanical Perspective. Forsch. Komplementärmed..

[35] Heilmann K.E. (1973). Kräuterbücher in Bild und Geschichte.

[36] Wurzer W. (2000). Die grosse Enzyklop.die der Heilpflanzen; ihre Anwendung und ihre Natürliche Heilkraft.

[37] Saller R., Reichling J., Hellenbrecht D. (1995). Phytotherapie; Klinische, Pharmakologische und Pharmazeutische Grundlagen.

[38] Flück H. (1941). Unsere Heilpflanzen.

[39] Leclerc H. (1976). Précis de Phytothérapie; Thérapeutique par les Plantes Francaises.

[40] Weiss R.F. (1985). Lehrbuch der Phytotherapie.

[41] Strank K.J., Meurers-Balke J. (2008). Obst, Gemüse und Kräuter Karls des Grossen.

[42] Lonicero A. (1679). Kreuterbuch.

[43] Wichtl M. (2008). Teedrogen und Phytopharmaka: Ein Handbuch für die Praxis auf Wissenschaftlicher Grundlage.

[44] Madaus G. (1938). Lehrbuch der Biologischen Heilmittel.

[45] Schramm H. (1997). Heilmittel- Fibel zur Anthroposophischen Medizin.

[46] Scheffer M. (2011). Die Original-Bachblüten-Therapie: Das Gesamte Theoretische und Praktische Bachblüten-Wissen.

[47] ESCOP (Ed.) (2009). ESCOP Monographs: The scientific Foundation for Herbal Medicinal Products: Supplement.

[48] ESCOP (Ed.) (2003). ESCOP Monographs: The Scientific Foundation for Herbal Medicinal Products.

[49] (2014). EMA (European Medicines Agency). http://www.ema.europa.eu/ema/index.jsp?curl=/pages/medicines/landing/herbal_search.

[50] Berendes J. (1902). Des Pedanios Dioskurides aus Anazarbos Arzneimittellehre in fünf Büchern.

[51] Mayer J.G., Goehl K. (2001). Höhepunkte der Klostermedizin; Der «Macer floridus» und das Herbarium des Vitus Auslasser.

[52] Dobat K., Dressendörfer W. (2001). Leonhart Fuchs. The New Herbal of 1543.

[53] Tabernaemontanus J.T.N. (1664). Kräuter-Buch, Jetzt Widerumb Auffs Newe Übersehen vnd Anderm Vermehret durch Hieronymus Bauhin.

[54] Kneipp S. (2010). Meine Wasserkur; So Sollt ihr Leben. Die Weltberühmten Ratgeber in Einem Band.

[55] Künzle J. (1945). Das grosse Kräuterheilbuch; Ratgeber für Gesunde und Kranke Tage.

[56] Vogel A. (1952). Der kleine Doktor.

[57] Valnet J. (1992). Phytotherapie: Traitement des Maladies par les Plantes.

[58] Treben M. (2011). Gesundheit aus der Apotheke Gottes.

[59] Vonarburg B. (1988). Natürlich Gesund mit Heilpflanzen.

[60] Bühring U. (2005). Praxis-Lehrbuch der Modernen Pflanzenheilkunde.

[61] Matthioli P.A. (1568). I Discorsi di M. Pietro Andrea Matthioli. Sanese, Medico Cesareo, et del Serenissimo Principe Ferdinando Archiduca d’Austria & c.

[62] Höfler M. (1899). Deutsches Krankheitsnamen-Buch.

[63] Landolt E. (2010). Flora indicativa; Ökologische Zeigerwerte und Biologische Kennzeichen zur Flora der Schweiz und der Alpen.

[64] KEWScience, Plants of the World online. https://powo.science.kew.org/.

[65] Stevens P.F. Angiosperm Phylogeny Website; 2001. http://mobot.org/MOBOT/research/APWeb/.

[66] Committee on Herbal Medicinal Products (HMPC) (Ed.) (2011). Community Herbal Monograph on *Achillea millefolium* L., Flos Final. EMA/HMPC/143949/2010. https://www.ema.europa.eu/en/medicines/herbal/millefolii-flos.

[67] Committee on Herbal Medicinal Products (HMPC) (Ed.) (2020). European Union Herbal Monograph on *Achillea millefolium* L., herba Final—Revision 1. EMA/HMPC/376416/2019. https://www.ema.europa.eu/en/medicines/herbal/millefolii-herba.

[68] Committee on Herbal Medicinal Products (HMPC) (Ed.) (2015). European Union Herbal Monograph on *Matricaria recutita* L., flos Final. EMA/HMPC/55843/2011. https://www.ema.europa.eu/en/medicines/herbal/matricariae-flos.

[69] Weckerle C.S., Huber F., Yongping Y., Weibang S. (2006). Plant Knowledge of the Shuhi in the Hengduan Mountains, Southwest China. Econ. Bot..

[70] Bradacs G., Heilmann J., Weckerle C.S. (2011). Medicinal plant use in Vanuatu: A comparative ethnobotanical study of three islands. J. Ethnopharmacol..

[71] Monigatti M., Bussmann R., Weckerle C.S. (2012). Medicinal plant use in two Andean communities located at different altitudes in the Bolìvar Province, Peru. J. Ethnopharmacol..

[72] (2015). B.F.S. Bundesamt für Statistik. http://www.bfs.admin.ch/bfs/portal/de/index/themen/14/02/04/key/01.html#parsys_60885.

[73] Götti R.P., Melzer J., Saller R. (2014). An Approach to the Concept of Tonic: Suggested Definitions and Historical Aspects. Forsch. Komplementärmed..

[74] Joshi B.C., Mukhija M., Kalia A.N. (2014). Pharmacognostical review of Urtica dioica L.. Int. J. Green Pharm..

[75] Szopa A., Pajor J., Klin P., Rzepiela A., Elansary H.O., Al-Mana F.A., Mattar M.A., Ekiert H. (2020). Artemisia absinthium L.-Importance in the History of Medicine, the Latest Advances in Phytochemistry and Therapeutical, Cosmetological and Culinary Uses. Plants.

[76] Crellin J.K. (2001). Social validation: An historian’s look at complementary/alternative medicine. Pharm. Hist..

[77] Directive 2004/24/EC. https://health.ec.europa.eu/medicinal-products/herbal-medicinal-products_en.

[78] Suleria H.A.R., Butt M.S., Khalid N., Sultan S., Raza A., Aleem M., Abbas M. (2015). Garlic (*Allium sativum*): Diet based therapy of 21st century—A review. Asian Pac. J. Trop. Dis..

[79] Chan W.-J.J., McLachlan A.J., Luca E.J., Harnett J.E. (2020). Garlic (*Allium sativum* L.) in the management of hypertension and dyslipidemia—A systematic review. J. Herb. Med..

[80] Abascal K., Yarnell E. (2004). Nervine Herbs for Treating Anxiety. Altern. Complement. Ther..

[81] Fischer B., Hartwich C. (1903). Hagers Handbuch der Pharmazeutischen Praxis.

[82] Anagnostou S. (2011). Johanniskraut, Baldrian und Passionsblume; Die Geschwister der Seele. Pharmazeutische Zeitung Online Ausgabe. Volume 48. http://www.pharmazeutische-zeitung.de/index.php?id=40172.

[83] Pollio A., Natale A., Appetiti E. (2008). Continuity and change in the Mediterranean medical tradition: *Ruta* spp. (Rutaceae) in Hippocratic medicine and present practices. J. Ethnopharmacol..

[84] Wegmann U. (2013). Ethnobotanik im Prättigau. Medizinalpflanzen—Nutzung und Wissen. Master’s Thesis.

[85] Poncet A. (2005). Pflanzen und Menschen im Emmental; Eine Ethnobotanische Studie über den Kräuterhandel einer Bauernfamilie des Voralpengebiets. Master’s Thesis.

[86] Broquet C. (2006). Chasseral, à la Rencontre de L’homme et du Vegetal: Enquêtes Ethnobotaniques sur L’utilisation des Plantes dans une Région de la Chaîne Jurassienne. Master’s Thesis.

[87] Brühschweiler S. (2008). Plantes et Savoirs des Alpes: L’exemple du Val d’Anniviers.

[88] Poretti G. (2011). La Malva Tücc i maa i a Calma: Inventario Etnobotanico delle Piante Medicinali del Cantone Ticino.

[89] Kummer G. (1953). Schaffhauser Volksbotanik; 1. Die Wildwachsenden Pflanzen.

[90] Gardiner P. (2007). Complementary, Holistic, and Integrative Medicine: Chamomile. Pediatr. Rev..

[91] McKay D.L., Blumberg J.B. (2006). A Review of the Bioactivity and Potential Health Benefits of Chamomile Tea (*Matricaria recutita* L.). Phytother. Res..

