# Supplementary material for: Trends of Medicinal Plant Use over the Last 2000 Years in Central Europe
Source: Plants (Basel). 2022 Dec 27;12(1):135. doi: 10.3390/plants12010135 (PMC9823631; doi:10.3390/plants12010135)
Supplement: Supplementary file 1 [file plants-12-00135-s001.zip › plants-2074055-supplementary.pdf]

Taxon	Familie	era	Herbal	German Name	inter	use-categ	source / chapter	Original text
Abies spp. (*Abies* spp., *Larix decidua*)	Pinaceae	ant	DIOS	Tanne	syst	DER	p. 92	Weiter dient es zum Zusammenkleben der Haare an den Augenlidern und mit Grünspan, Kupfervitriol und Natron gegen Aussatz, mit Honig und Öl gegen eiterige Ohren und gegen Jucken an den Geschlechtsteilen
Abies spp. (*Abies* spp., *Larix decidua*)	Pinaceae	ant	DIOS	Tanne	syst	OTH	p. 92	Endlich hilft es auch bei Seitenschmerzen für sich allein eingeschmiert oder aufgelegt.
Abies spp. (*Abies* spp., *Larix decidua*)	Pinaceae	ant	DIOS	Tanne	syst	RES	p. 92	Husten und Schwindsucht als Leckmittel für sich allein oder mit Honig, es ist auch ein Expectorans.
Abies spp. (*Abies* spp., *Larix decidua*)	Pinaceae	ant	DIOS	Lärche	syst	RES	92	welches in der Latwerge und für sich allein vorzügliche Dienste leistet gegen chronischen Husten
Abies spp. (*Abies* spp., *Larix decidua*)	Pinaceae	ant	DIOS	Tanne	syst	URO	p. 273	Die Arzneiweine sind den Gesunden nicht zuträglich; alle sind sie erwärmend, harntreibend, etwas adstringirend,
Abies spp. (*Abies* spp., *Larix decidua*)	Pinaceae	ant	DIOS	Tanne	syst	URO	p. 92	Ferner ist es harntreibend und verdichtend und erweicht den Leib
Abies spp. (*Abies* spp., *Larix decidua*)	Pinaceae	mon	HvB	Tanne	n.d.	APO	2-23	Zauber und Magie haben dort weniger Kraft und herrschen weniger vor als an anderen Orten
Abies spp. (*Abies* spp., *Larix decidua*)	Pinaceae	mon	HvB	Tanne	top	CAR	2-23	dessen Stärke im Herzen versagt
Abies spp. (*Abies* spp., *Larix decidua*)	Pinaceae	mon	HvB	Tanne	top	DER	2-23	wenn Krebse den Menschen zerfressen, soll das Pulver auf jene Wunden streuen, Mund und Lippen aus irgendwinem Grund schwellen
Abies spp. (*Abies* spp., *Larix decidua*)	Pinaceae	mon	HvB	Tanne	top	EYE	2-23	Kopf waschen, dies heilt den Kopf und macht die Augen klar
Abies spp. (*Abies* spp., *Larix decidua*)	Pinaceae	mon	HvB	Tanne	top	GAS	2-23	Magenschmerzen
Abies spp. (*Abies* spp., *Larix decidua*)	Pinaceae	mon	HvB	Tanne	top	NER	2-23	Kopfschmerzen, Geisteskrank, hirnwütig'
Abies spp. (*Abies* spp., *Larix decidua*)	Pinaceae	mon	HvB	Tanne	top	OTH	2-23	Milz, wenn er dort leidet
Abies spp. (*Abies* spp., *Larix decidua*)	Pinaceae	mon	HvB	Tanne	syst	RES	2-23	auf der Brust dämpfig ist und hustet und auch Schmerzen in der Lunge hat . . .
Abies spp. (*Abies* spp., *Larix decidua*)	Pinaceae	mon	HvB	Tanne	vol	RES	2-23	Fluss reichlich aus der Nase fliesst
Abies spp. (*Abies* spp., *Larix decidua*)	Pinaceae	mon	HvB	Tanne	top	SKE	2-23	Gicht
Abies spp. (*Abies* spp., *Larix decidua*)	Pinaceae	ren	LF	Lärche	top	DER	CCLXXX / CLXXXIX	schaeden so umb sich fressen
Abies spp. (*Abies* spp., *Larix decidua*)	Pinaceae	ren	LF	Lärche	top	DER	CCLXXX / CLXXXIX	so den wolff geritten haben und heylet die geschwaer der haut und den brand
Abies spp. (*Abies* spp., *Larix decidua*)	Pinaceae	ren	LF	Lärche	top	DER	CCLXXX / CLXXXIX	entzündet wunden, wunden heylen
Abies spp. (*Abies* spp., *Larix decidua*)	Pinaceae	ren	LF	Lärche	top	DER	CCLXXX / CLXXXIX	dass das har an den augenbrauen ausfellt, vertreibt allerley grind, rauden, geflecht am leib
Abies spp. (*Abies* spp., *Larix decidua*)	Pinaceae	ren	LF	Lärche	top	EAR	CCLXXX / CLXXXIX	eitrechten ohren
Abies spp. (*Abies* spp., *Larix decidua*)	Pinaceae	ren	LF	Lärche	syst	GAS	CCLXXX / CLXXXIX	stellt den bauchfluss
Abies spp. (*Abies* spp., *Larix decidua*)	Pinaceae	ren	LF	Lärche	syst	GAS	CCLXXX / CLXXXIX	macht einen weichen stuhlgang
Abies spp. (*Abies* spp., *Larix decidua*)	Pinaceae	ren	LF	Lärche	vol	GYN	CCLXXX / CLXXXIX	treibt das bürdlin von den frawen
Abies spp. (*Abies* spp., *Larix decidua*)	Pinaceae	ren	LF	Lärche	syst	n.d.	CCLXXX / CLXXXIX	zeitiget
Abies spp. (*Abies* spp., *Larix decidua*)	Pinaceae	ren	LF	Lärche	top	n.d.	CCLXXX / CLXXXIX	weetagen der seiten
Abies spp. (*Abies* spp., *Larix decidua*)	Pinaceae	ren	LF	Lärche	syst	RES	CCLXXX / CLXXXIX	husten, schwindsüchtigen, seubert die brust
Abies spp. (*Abies* spp., *Larix decidua*)	Pinaceae	ren	LF	Lärche	top	TEE	CCLXXX / CLXXXIX	stillet das zahnwee
Abies spp. (*Abies* spp., *Larix decidua*)	Pinaceae	ren	LF	Lärche	syst	URO	CCLXXX / CLXXXIX	[stellt den bauchfluss] und treibt den harn
Abies spp. (*Abies* spp., *Larix decidua*)	Pinaceae	ren	LF	Lärche	syst	URO	CCLXXX / CLXXXIX	treibt den harn
Abies spp. (*Abies* spp., *Larix decidua*)	Pinaceae	s pop	AV	Tanne	syst	RES	p. 34	Katarrhe
Abies spp. (*Abies* spp., *Larix decidua*)	Pinaceae	s pop	JK	Weisstanne	top	DER	p. 426	Hautkranke
Abies spp. (*Abies* spp., *Larix decidua*)	Pinaceae	s pop	JK	Weisstanne	top	DER	p. 426	Wundsalbe
Abies spp. (*Abies* spp., *Larix decidua*)	Pinaceae	s pop	JK	Weisstanne	syst	FEV	p. 426	fiebersenkend, blutreinigend
Abies spp. (*Abies* spp., *Larix decidua*)	Pinaceae	s pop	JK	Weisstanne	top	NER	p. 426	Nervenkranke
Abies spp. (*Abies* spp., *Larix decidua*)	Pinaceae	s pop	JK	Weisstanne	syst	RES	p. 426	Hustensirup
Abies spp. (*Abies* spp., *Larix decidua*)	Pinaceae	s pop	JK	Weisstanne	vol	RES	p. 426	Astmah und Lungenkranke
Abies spp. (*Abies* spp., *Larix decidua*)	Pinaceae	s pop	KN	Tanne	syst	RES	p. 469	kräftigen die Brust und wirken merkwürdig stärkend auf die innneren Gefässe
Achillea millefolium agg.	Asteraceae	ant	DIOS	Schafgarbe	n.d.	DER	p. 239	Blutflüsse, alte und frische Wunden und Fisteln
Achillea millefolium agg.	Asteraceae	mon	HvB	Schafgarbe	syst	DER	1-113	Körperinnern eine Wunde
Achillea millefolium agg.	Asteraceae	mon	HvB	Schafgarbe	top	DER	1-113	Wunden
Achillea millefolium agg.	Asteraceae	mon	HvB	Schafgarbe	top	EYE	1-113	vom Vergiessen der Tränen in den Augen verdunkelt
Achillea millefolium agg.	Asteraceae	mon	HvB	Schafgarbe	syst	FEV	1-113	Dreitagefieber
Achillea millefolium agg.	Asteraceae	ren	LF	Schafgarbe	n.d.	DER	CCCCXV / CCLXXVIII	treiben auch das gerunnen Blut und seind in summa treffenliche Wundkreüter
Achillea millefolium agg.	Asteraceae	ren	LF	Schafgarbe	n.d.	DER	CCCCXV / CCLXXVIII	böse Fistel
Achillea millefolium agg.	Asteraceae	ren	LF	Schafgarbe	syst	DER	CCCCXV / CCLXXVIII	allerley eüsserlichen und innerlichen wunden und geschwaeren
Achillea millefolium agg.	Asteraceae	ren	LF	Schafgarbe	top	DER	CCCCXV / CCLXXVIII	allerley eüsserlichen und innerlichen wunden und geschwaeren
Achillea millefolium agg.	Asteraceae	ren	LF	Schafgarbe	top	DER	CCCCXV / CCLXXVIII	stellen Blut, zerstossen und übergelegt
Achillea millefolium agg.	Asteraceae	ren	LF	Schafgarbe	top	GYN	CCCCXV / CCLXXVIII	nützen den Frawen so sie zuviel fliessen ihrer Krankheyt
Achillea millefolium agg.	Asteraceae	s pop	AV	Schafgarbe	syst	CAR	p. 180	Kreislaufunterstützung (Thrombose / Embolien)
Achillea millefolium agg.	Asteraceae	s pop	AV	Schafgarbe	syst	CAR	p. 180	Hämorrhoiden
Achillea millefolium agg.	Asteraceae	s pop	AV	Schafgarbe	syst	DER	p. 529	Skrofulose, Neigung zu Ekzemen
Achillea millefolium agg.	Asteraceae	s pop	AV	Schafgarbe	top	DER	p. 38, 71	akute Venenentzündung, Thrombosen, Venenpflege
Achillea millefolium agg.	Asteraceae	s pop	AV	Schafgarbe	syst	GAS	p. 38	Appetitlosigkeit, Durchfall (Säuglinge)
Achillea millefolium agg.	Asteraceae	s pop	AV	Schafgarbe	syst	URO	p. 251	Blasenentzündung
Achillea millefolium agg.	Asteraceae	s pop	AV	Schafgarbe	syst	URO	p. 529	Wassersucht
Achillea millefolium agg.	Asteraceae	s pop	BVA	Schafgarbe	syst	CAR	p. 185	Zirkulationsstörungen, Hämorrhoiden, Krampfadern, Angina perctoris, Herzenge
Achillea millefolium agg.	Asteraceae	s pop	BVA	Schafgarbe	syst	CAR	p. 185	Zirkulationsstörungen, Hämorrhoiden, Krampfadern, Angina perctoris, Herzenge
Achillea millefolium agg.	Asteraceae	s pop	BVA	Schafgarbe	syst	CAR	p. 185	regt Funktion des Blutgefässsystems an, gegen Schwindel, Blutandrang im Kopf, Wechseljahrbeschwerden
Achillea millefolium agg.	Asteraceae	s pop	BVA	Schafgarbe	syst	DER	p. 185	Nasenbluten, Förderung der Blutgerinnung
Achillea millefolium agg.	Asteraceae	s pop	BVA	Schafgarbe	syst	GAS	p. 185	Nasenbluten, Förderung der Blutgerinnung
Achillea millefolium agg.	Asteraceae	s pop	BVA	Schafgarbe	top	DER	p. 185	Afterriss, Verletzungen
Achillea millefolium agg.	Asteraceae	s pop	BVA	Schafgarbe	top	DER	p. 185	wunde Brustwarzen
Achillea millefolium agg.	Asteraceae	s pop	BVA	Schafgarbe	syst	GAS	p. 185	Magenbeschwerden
Achillea millefolium agg.	Asteraceae	s pop	BVA	Schafgarbe	syst	GAS	p. 185	Magenbeschwerden
Achillea millefolium agg.	Asteraceae	s pop	BVA	Schafgarbe	syst	GYN	p. 185	schmerzhafte Periode, starken / schwachen Menstruationsbllutungen, Weissfluss, Menstruationsanomalien, Regelung der Periode, Gembärmutter katarrh, Gebärmutterblutung, Wecheslјahren
Achillea millefolium agg.	Asteraceae	s pop	BVA	Schafgarbe	syst	GYN	p. 185	schmerzhafte Periode, starken / schwachen Menstruationsbllutungen, Weissfluss, Menstruationsanomalien, Regelung der Periode, Gembärmutter katarrh, Gebärmutterblutung, Wecheslјahren
Achillea millefolium agg.	Asteraceae	s pop	JK	Schafgarbe	syst	CAR	p. 414	Hämorrhoiden
Achillea millefolium agg.	Asteraceae	s pop	JK	Schafgarbe	top	DER	p. 414	Hämorrhoiden
Achillea millefolium agg.	Asteraceae	s pop	JK	Schafgarbe	top	DER	p. 414	Wunden von Stich, Schlag, Schnitt oder Schürfungen
Achillea millefolium agg.	Asteraceae	s pop	JK	Schafgarbe	syst	GAS	p. 414	Magenkrämpfen
Achillea millefolium agg.	Asteraceae	s pop	JK	Schafgarbe	syst	GAS	p. 414	Appetitlosigkeit, gegen starkes Abweichen, Blut im Stohlgang
Achillea millefolium agg.	Asteraceae	s pop	JK	Schafgarbe	syst	GYN	p. 414	stärken Mutterschaftsorgane, Weissfluss, zu starken Blutfluss, regulieren die Periode, bei Frauenleiden eine Kur mit Schafgarbentee angezeigt.
Achillea millefolium agg.	Asteraceae	s pop	JK	Schafgarbe	syst	RES	p. 414	Lungenleiden
Achillea millefolium agg.	Asteraceae	s pop	JK	Schafgarbe	syst	SKE	p. 414	Rheumatismus
Achillea millefolium agg.	Asteraceae	s pop	JK	Schafgarbe	syst	SKE	p. 414	verstocktes Blut in den inneren Organen, das oft eine Folge von Brüchen, Stoss oder Fall ist
Achillea millefolium agg.	Asteraceae	s pop	JK	Schafgarbe	top	SKE	p. 414	Brüchen
Achillea millefolium agg.	Asteraceae	s pop	JK	Schafgarbe	top	TEE	p. 414	Zahnweh
Achillea millefolium agg.	Asteraceae	s pop	JK	Schafgarbe	syst	TON	p. 414	Iva-Likör
Achillea millefolium agg.	Asteraceae	s pop	JK	Schafgarbe	syst	URO	p. 414	Nieren-, Balsen-, Gallensteine
Achillea millefolium agg.	Asteraceae	s pop	KN	Schafgarbe	syst	CAR	p. 484	Zirkulationsfördernde Wirkung
Achillea millefolium agg.	Asteraceae	s pop	KN	Schafgarbe	top	DER	p. 484	Schuppenflechte, schlecht heilden Geschwüre, Wunden
Achillea millefolium agg.	Asteraceae	s pop	KN	Schafgarbe	syst	GAS	p. 475	Gasbildung, Verstopfung, Leberstauung
Achillea millefolium agg.	Asteraceae	s pop	KN	Schafgarbe	syst	GAS	p. 475	Kopfleiden, die von wässrigen Stoffen oder Verschleimung im Kopfe herrühren, leicht Verscleimung von Brust und Lunge

Achillea millefolium agg.	Asteraceae	s pop	KN	Schafgarbe	syst	GAS	p. 484	Appetitlosigkeit, Magen- und Darmkrämpfen, Dickdarmentzündung, Leberschwellung
Achillea millefolium agg.	Asteraceae	s pop	KN	Schafgarbe	syst	GYN	p. 475	Kräftigung der weiblichen Unterleibsorgane
Achillea millefolium agg.	Asteraceae	s pop	KN	Schafgarbe	syst	GYN	p. 484	Unregelmässigkeit der Periode
Achillea millefolium agg.	Asteraceae	s pop	KN	Schafgarbe	syst	URO	p. 475	Bettnässer
Achillea millefolium agg.	Asteraceae	s pop	UB	Schafgarbe	syst	CAR	p. 410	venösen Gefässerkrankungen
Achillea millefolium agg.	Asteraceae	s pop	UB	Schafgarbe	top	DER	p. 305	Wundbehandlung
Achillea millefolium agg.	Asteraceae	s pop	UB	Schafgarbe	top	DER	p. 305	Wundbehandlung
Achillea millefolium agg.	Asteraceae	s pop	UB	Schafgarbe	top	DER	p. 305	Wundbehandlung
Achillea millefolium agg.	Asteraceae	s pop	UB	Schafgarbe	syst	GAS	p.158	Magenerkrankungen / Amara aromatica
Achillea millefolium agg.	Asteraceae	s pop	UB	Schafgarbe	syst	GAS	p.158	Magenerkrankungen / Amara aromatica
Achillea millefolium agg.	Asteraceae	s pop	WI	Schafgarbe	top	DER	p. 397	Volksheilkunde: Hämostyptikum (z.B. Hämorrhoidenblutungen)
Achillea millefolium agg.	Asteraceae	sci	EMA	Schafgarbe	top	DER	HMPC/143949/2010	small superficial wounds
Achillea millefolium agg.	Asteraceae	sci	EMA	Schafgarbe	top	DER	HMPC/143949/2010	small superficial wounds
Achillea millefolium agg.	Asteraceae	sci	EMA	Schafgarbe	top	DER	HMPC/290284/2009	small superficial wounds
Achillea millefolium agg.	Asteraceae	sci	EMA	Schafgarbe	syst	GAS	HMPC/143949/2010	temporary loss of appetite / mild spasmodic gastrointestinal complaints including bloating and flatulence
Achillea millefolium agg.	Asteraceae	sci	EMA	Schafgarbe	syst	GAS	HMPC/290284/2009	temporary loss of appetite / mild spasmodic gastrointestinal complaints including bloating and flatulence
Achillea millefolium agg.	Asteraceae	sci	EMA	Schafgarbe	syst	GAS	HMPC/290284/2009	temporary loss of appetite / mild spasmodic gastrointestinal complaints including bloating and flatulence
Achillea millefolium agg.	Asteraceae	sci	EMA	Schafgarbe	syst	GAS	HMPC/290284/2009	temporary loss of appetite / mild spasmodic gastrointestinal complaints including bloating and flatulence
Achillea millefolium agg.	Asteraceae	sci	EMA	Schafgarbe	syst	GYN	HMPC/290284/2009	minor spasms associated with menstrual periods
Achillea millefolium agg.	Asteraceae	sci	ESCOP	Millefolii herba	top	DER	II p. 176	supportive treatment of small wounds, mild inflammations of the skin and mucous membranes, as an insect repellent
Achillea millefolium agg.	Asteraceae	sci	ESCOP	Millefolii herba	top	GYN	II p. 176	spasm of the small pelvis
Acorus calamus L.	Acoraceae	ant	DIOS	Kalmus	syst	ANT	1/68	mit Vorteil wird aber auch die Wurzel den Gegengiften zu- gemischt.
Acorus calamus L.	Acoraceae	ant	DIOS	Kalmus	syst	ANT	1/68	und den von giftigen Tieren Gebissenen,
Acorus calamus L.	Acoraceae	ant	DIOS	Kalmus	syst	EYE	1/68	Verdunkelungen auf der Pupille
Acorus calamus L.	Acoraceae	ant	DIOS	Kalmus	syst	GAS	1/68	und Leberleiden, bei Leibschneiden, Zerreißungen und Krämpfen
Acorus calamus L.	Acoraceae	ant	DIOS	Kalmus	top	GYN	1/68	eignet sich wie die Schwertlilie zu Sitzbädern bei Frauenkrankheiten
Acorus calamus L.	Acoraceae	ant	DIOS	Kalmus	syst	OTH	1/68	erweicht die Milz
Acorus calamus L.	Acoraceae	ant	DIOS	Kalmus	syst	RES	1/68	gutes Mittel bei Lungen-, Brust-
Acorus calamus L.	Acoraceae	ant	DIOS	Kalmus	syst	URO	1/68	Eine Abkochung davon getrunken treibt den Harn, ist auch ein
Acorus calamus L.	Acoraceae	ant	DIOS	Kalmus	syst	URO	1/68	hilft den an Harnzwang Leidenden
Acorus calamus L.	Acoraceae	mon	LO	Kalmus	syst	OTH	n.d.	n.d.
Acorus calamus L.	Acoraceae	ren	TAB	Kalmus	syst	GYN		treibet fort den weiblichen Monatblumen.
Acorus calamus L.	Acoraceae	ren	TAB	Kalmus	top	GYN		wider die Gebrechen der Geburtglieder/ wie ein Lendenbad gebrauche
Acorus calamus L.	Acoraceae	ren	TAB	Kalmus	top	GYN		in die Mutter gethan/ heylet die umbsich fressende Geschwer derselb
Acorus calamus L.	Acoraceae	ren	TAB	Kalmus	top	OTH		fördert den Schweiss gewaltiglich
Acorus calamus L.	Acoraceae	ren	TAB	Kalmus	vol	RES		unnd den Rauch darvon in den Mundt durch ein Rohr empfangen/ ve
Acorus calamus L.	Acoraceae	ren	TAB	Kalmus	syst	URO		wider die Wassersucht/ Gebrechen der Nieren/ wieder die Harnwindt u
Acorus calamus L.	Acoraceae	ren	TAB	Kalmus	syst	URO		erwärmet den kalten Magen/ ist denjenigen fast dienlich die kein Lust
Acorus calamus L.	Acoraceae	s pop	KN	Kalmus	top	DER	p. 413	Skrofulose
Acorus calamus L.	Acoraceae	s pop	MT	Kalmus	top	DER	p. 27	Forstbeulen, Erfrierungen
Acorus calamus L.	Acoraceae	s pop	MT	Kalmus	syst	GAS	p. 26	bei allgemeiner Schwäche der Verdauungsorgane
Acorus calamus L.	Acoraceae	s pop	MT	Kalmus	syst	OTH	p. 26	Lungenkrebs, Darmkrebs
Acorus calamus L.	Acoraceae	s pop	UB	Kalmus	syst	GAS	p. 156	Magenerkrankungen / Amara aromatica
Acorus calamus L.	Acoraceae	s pop	UB	Kalmus	syst	GAS	p. 156	Magenerkrankungen / Amara aromatica
Acorus calamus L.	Acoraceae	s pop	UB	Kalmus	syst	GAS	p. 156	Magenerkrankungen / Amara aromatica
Acorus calamus L.	Acoraceae	s pop	UB	Kalmus	syst	GAS	p. 156	Magenerkrankungen / Amara aromatica
Acorus calamus L.	Acoraceae	s pop	UB	Kalmus	top	SKE	p. 290	degenerativen rheumatischen Erkrankungen
Acorus calamus L.	Acoraceae	s pop	UB	Kalmus	top	SKE	p. 290	degenerativen rheumatischen Erkrankungen
Acorus calamus L.	Acoraceae	s pop	WI	Kalmus	syst	NER	p. 117	Volksmedizin: Nervinum
Acorus calamus L.	Acoraceae	sci	WI	Kalmus	top	DER	p. 117	Hautreizmittel
Acorus calamus L.	Acoraceae	sci	WI	Kalmus	syst	GAS	p. 117	Amara aromaticum; Stomachikum und Karminativum
Adiantum capillus-veneris L.	Pteridaceae	ant	DIOS	Frauenhaarfarn	syst	ANT	p. 245	hilft gegen den Biss giftiger Thiere
Adiantum capillus-veneris L.	Pteridaceae	ant	DIOS	Frauenhaarfarn	top	ANT	p. 245	bei Bissen giftiger Tliiere benutzt
Adiantum capillus-veneris L.	Pteridaceae	ant	DIOS	Frauenhaarfarn	top	DER	p. 245	befördert nach der Fuchskrankheit den Haarwuchs und vertheilt Drüsen am Halse
Adiantum capillus-veneris L.	Pteridaceae	ant	DIOS	Frauenhaarfarn	syst	GAS	p. 245	hat die Kraft, bei . . . .Gelbsucht / stellt den Durchfall / auch bei Magenfluss
Adiantum capillus-veneris L.	Pteridaceae	ant	DIOS	Frauenhaarfarn	syst	GYN	p. 245	befördert die Menstruation und die Reinigung der Wöchnerinnen
Adiantum capillus-veneris L.	Pteridaceae	ant	DIOS	Frauenhaarfarn	syst	OTH	p. 245	hat die Kraft, bei . . . . Pilzkrankheiten
Adiantum capillus-veneris L.	Pteridaceae	ant	DIOS	Frauenhaarfarn	syst	RES	p. 245	hat die Kraft, bei Asthma, Engbrüstigkeit
Adiantum capillus-veneris L.	Pteridaceae	ant	DIOS	Frauenhaarfarn	syst	URO	p. 245	hat die Kraft, bei . . . .Harnverhaltung, zerztrümmert ferner den Stein
Adiantum capillus-veneris L.	Pteridaceae	mon	LO	Frauenhaarfarn	n.d.	OTH	n.d.	n.d.
Adiantum capillus-veneris L.	Pteridaceae	ren	LF	Frauenhaarfarn	syst	ANT	XLV / XXVIII	denen so von einem giffitgen thier gebissen seind
Adiantum capillus-veneris L.	Pteridaceae	ren	LF	Frauenhaarfarn	n.d.	DER	XLV / XXVIII	vertreibt die kroepff
Adiantum capillus-veneris L.	Pteridaceae	ren	LF	Frauenhaarfarn	top	DER	XLV / XXVIII	denen von einem gifftign thier gebissen seind / macht das har widerumb wachsen
Adiantum capillus-veneris L.	Pteridaceae	ren	LF	Frauenhaarfarn	top	DER	XLV / XXVIII	vertreibt es die schuepe auff dem kopff
Adiantum capillus-veneris L.	Pteridaceae	ren	LF	Frauenhaarfarn	syst	GAS	XLV / XXVIII	denen so die geelsucht haben / stillet den bauchfluss
Adiantum capillus-veneris L.	Pteridaceae	ren	LF	Frauenhaarfarn	syst	GAS	XLV / XXVIII	stellet das blut speyen
Adiantum capillus-veneris L.	Pteridaceae	ren	LF	Frauenhaarfarn	syst	GYN	XLV / XXVIII	brint den frawen ihre bloedigkeit und rreibt auss das bürdlin
Adiantum capillus-veneris L.	Pteridaceae	ren	LF	Frauenhaarfarn	syst	OTH	XLV / XXVIII	den milzsüchtigen
Adiantum capillus-veneris L.	Pteridaceae	ren	LF	Frauenhaarfarn	syst	RES	XLV / XXVIII	denen so einen schwaeren athem haben und keichen
Adiantum capillus-veneris L.	Pteridaceae	ren	LF	Frauenhaarfarn	syst	URO	XLV / XXVIII	treibt den harn / zermalt den stein
Adiantum capillus-veneris L.	Pteridaceae	s pop	VAL	Frauenhaarfarn	syst	RES	p. 200	bronchites, trachéites
Adiantum capillus-veneris L.	Pteridaceae	s pop	VAL	Frauenhaarfarn	syst	URO	p. 200	inflammations urinaires
Agrimonia eupatoria L.	Rosaceae	ant	DIOS	Odermennig	syst	ANT	p. 222	Schlangenbisse
Agrimonia eupatoria L.	Rosaceae	ant	DIOS	Odermennig	top	DER	p. 222	heilen schwer vernarbende Geschwüre
Agrimonia eupatoria L.	Rosaceae	ant	DIOS	Odermennig	syst	GAS	p. 222	dysenterie
Agrimonia eupatoria L.	Rosaceae	mon	HvB	Odermennig	syst	GAS	1-114	kranke Eingeweide, kalter Magen
Agrimonia eupatoria L.	Rosaceae	mon	HvB	Odermennig	syst	GAS	1-114	Stuhldrang, erweicht verhärteten Magen
Agrimonia eupatoria L.	Rosaceae	mon	HvB	Odermennig	top	NER	1-114	Wahnsinn, Wissen und das Gefühl entleert
Agrimonia eupatoria L.	Rosaceae	mon	HvB	Odermennig	syst	RES	1-114	vom Speichel, Auswurf und Ausschneuzen gereinigt
Agrimonia eupatoria L.	Rosaceae	ren	LF	Odermenig	syst	ANT	CXXXV/CX	denen von nattern gebissen
Agrimonia eupatoria L.	Rosaceae	ren	LF	Odermenig	top	DER	CXXXV/CX	heylet die wunden, so sie nt leicht zur heylung schicken wollen
Agrimonia eupatoria L.	Rosaceae	ren	LF	Odermenig	syst	GAS	CXXXV/CX	rote rhur
Agrimonia eupatoria L.	Rosaceae	ren	LF	Odermenig	syst	GAS	CXXXV/CX	lebersüchtigen, verstopfften leber, sterckt auch dieselbigen
Agrimonia eupatoria L.	Rosaceae	s pop	JK	Odermennig	syst	CAR	p. 400	alle Gebresten Herz
Agrimonia eupatoria L.	Rosaceae	s pop	JK	Odermennig	top	DER	p. 400	Geschwulste und Geschwüre
Agrimonia eupatoria L.	Rosaceae	s pop	JK	Odermennig	top	EYE	p. 400	Augenleiden
Agrimonia eupatoria L.	Rosaceae	s pop	JK	Odermennig	syst	GAS	p. 400	alle Gebresten von Magen, Milz, Leber, Galle . . . Und gegen die Würmer
Agrimonia eupatoria L.	Rosaceae	s pop	JK	Odermennig	top	NER	p. 400	Schlaflosigkeit
Agrimonia eupatoria L.	Rosaceae	s pop	JK	Odermennig	syst	OTH	p. 400	Milzleiden
Agrimonia eupatoria L.	Rosaceae	s pop	JK	Odermennig	syst	RES	p. 400	alle Gebresten von Lunge, Husten, Hals- und Mandelentzündung
Agrimonia eupatoria L.	Rosaceae	s pop	JK	Odermennig	syst	SKE	p. 400	Rheumatismus
Agrimonia eupatoria L.	Rosaceae	s pop	JK	Odermennig	top	TON	p. 400	Müdigkeit und Fussschmerz
Agrimonia eupatoria L.	Rosaceae	s pop	UB	Odermennig	syst	GAS	p. 125	Durchfall
Agrimonia eupatoria L.	Rosaceae	s pop	UB	Odermennig	syst	GAS	p. 125	Durchfall
Agrimonia eupatoria L.	Rosaceae	s pop	UB	Odermennig	syst	GAS	p. 125	Durchfall
Agrimonia eupatoria L.	Rosaceae	s pop	UB	Odermennig	top	RES	p. 125	chronischen Pharyngitiden der Redner und Sänger
Agrimonia eupatoria L.	Rosaceae	s pop	WI	Odermennig	syst	GAS	p. 40	Volksheilkunde: Cholezystopathien
Agrimonia eupatoria L.	Rosaceae	sci	EMA	Odermennig	top	DER	HMPC/680597/2013	minor inflammation of the mouth and throat /
Agrimonia eupatoria L.	Rosaceae	sci	EMA	Odermennig	top	DER	HMPC/680597/2013	minor skin inflammation and small superficial wounds
Agrimonia eupatoria L.	Rosaceae	sci	EMA	Odermennig	syst	GAS	HMPC/680597/2013	mild diarrhoea
Agrimonia eupatoria L.	Rosaceae	sci	EMA	Odermennig	syst	GAS	HMPC/680597/2013	mild diarrhoea
Agrimonia eupatoria L.	Rosaceae	sci	ESCOP	Agrimoniae herba	top	RES	II p. 3	inflammation of the oral and pharyngeal mucosa
Allium cepa L.	Amaryllidacea	ant	DIOS	Zwiebel	syst	CAR	152	und für die Hämorrhoiden

Allium cepa L.	Amaryllidacea	ant	DIOS	Zwiebel	top	DER	152	eingestrichen gegen Entzündung der Schlundmuskeln
Allium cepa L.	Amaryllidacea	ant	DIOS	Zwiebel	top	DER	152	Mit Hühnerfett wird er gegen Druck der Sandalen gebraucht
Allium cepa L.	Amaryllidacea	ant	DIOS	Zwiebel	top	DER	152	Mit gleichviel Spodium heilt er Augenkrätze und mit Salz Finnen
Allium cepa L.	Amaryllidacea	ant	DIOS	Zwiebel	top	DER	152	Bei Hundsbiss hilft er als Umschlag mit Salz, Raute und Honig. Mit Essig in der Sonne eingerieben entfernt er weiße Flecken
Allium cepa L.	Amaryllidacea	ant	DIOS	Zwiebel	top	DER	152	und eingerieben gegen Fuchskrankheit, denn rascher als Alkyonium ruft er Haare hervor
Allium cepa L.	Amaryllidacea	ant	DIOS	Zwiebel	top	DER	152	Endlich reift und öffnet sie mit Rosinen und Feigen gekocht als Umschlag Geschwüre
Allium cepa L.	Amaryllidacea	ant	DIOS	Zwiebel	top	EAR	152	Schwerhörigkeit, Ohrenklingen und eiterflüssige Ohren; er dient gegen Anhalten der Feuchtigkeit (des Wassers) in den Ohren
Allium cepa L.	Amaryllidacea	ant	DIOS	Zwiebel	top	EYE	152	hilft gegen Stumpfsichtigkeit, gegen Flimmern, auch gegen entstehende Flecken auf den Augen
Allium cepa L.	Amaryllidacea	ant	DIOS	Zwiebel	syst	GAS	152	Sämtlich sind sie aber beißend und blähend, sie reizen den Appetit, ..., ..., verursachen bei Magenüberfüllung Ekel, ..., sind gut für den Bauch, ...
Allium cepa L.	Amaryllidacea	ant	DIOS	Zwiebel	syst	GAS	152	gegen Bauchfluss
Allium cepa L.	Amaryllidacea	ant	DIOS	Zwiebel	syst	GYN	152	Er befördert und treibt die Menstruation,
Allium cepa L.	Amaryllidacea	ant	DIOS	Zwiebel	syst	HUM	152	verdünnen, ..., ..., reinigen, ..., eröffnen den Weg zur Ausscheidung der übrigen Auswurfstoffe .
Allium cepa L.	Amaryllidacea	ant	DIOS	Zwiebel	top	HUM	152	eingespritzt reinigt er durch die Nase den Kopf.
Allium cepa L.	Amaryllidacea	ant	DIOS	Zwiebel	top	NER	152	Bei krankem Zustande bewirkt ihr reichlicher Genuss Schlafsucht
Allium cepa L.	Amaryllidacea	ant	DIOS	Zwiebel	top	OTH	152	Im Übermaß genossen verursacht die Zwiebel Kopfschmerzen
Allium cepa L.	Amaryllidacea	ant	DIOS	Zwiebel	top	URO	152	gekocht wird sie harntreibender
Allium cepa L.	Amaryllidacea	mon	MF	Zwiebel	top	ANT	33	Zwiebel mit Raute auf Schlangenbisse auflegen, das helfe viel
Allium cepa L.	Amaryllidacea	mon	MF	Zwiebel	top	DER	33	macht sie Hundebisse heilen
Allium cepa L.	Amaryllidacea	mon	MF	Zwiebel	top	DER	33	wundgelaufene Füsse
Allium cepa L.	Amaryllidacea	mon	MF	Zwiebel	top	DER	33	heilt Geschwüre des Mundes
Allium cepa L.	Amaryllidacea	mon	MF	Zwiebel	top	DER	33	Haemorrhoiden aufzulegen
Allium cepa L.	Amaryllidacea	mon	MF	Zwiebel	top	DER	33	Male auf der Haut zu tilgen
Allium cepa L.	Amaryllidacea	mon	MF	Zwiebel	top	DER	33	Haupt von Haar entblöst
Allium cepa L.	Amaryllidacea	mon	MF	Zwiebel	top	EAR	33	vertreibt schwere Ohrschmerzen
Allium cepa L.	Amaryllidacea	mon	MF	Zwiebel	top	EYE	33	macht trübe Augen hellsichtig
Allium cepa L.	Amaryllidacea	mon	MF	Zwiebel	n.d.	GAS	33	erweicht heilsam den harten Leib
Allium cepa L.	Amaryllidacea	mon	MF	Zwiebel	syst	GAS	33	bringt beissenden Schmerz, den Blutstuhl begleitet zur Ruhe
Allium cepa L.	Amaryllidacea	mon	MF	Zwiebel	syst	GAS	33	hilft bei Mundgeruch, bei Ekel und Abschei vor Speisen
Allium cepa L.	Amaryllidacea	mon	MF	Zwiebel	syst	GYN	33	ruft zögernden Monatsfluss zur Ordnung
Allium cepa L.	Amaryllidacea	mon	MF	Zwiebel	top	HUM	33	zwingt er die bösen Säfte aus dem Haupte zu weichen
Allium cepa L.	Amaryllidacea	mon	MF	Zwiebel	syst	NER	33	bringt Schlummer
Allium cepa L.	Amaryllidacea	mon	MF	Zwiebel	syst	NER	33	plötzlicher Krankheitsanfall den Leidenden verstummen macht
Allium cepa L.	Amaryllidacea	mon	MF	Zwiebel	top	TEE	33	jeden Morgen die Zähne reibt
Allium cepa L.	Amaryllidacea	ren	LF	Zwiebeln	top	DER	CCXLI / CLXIII	so die Schuch getruckt haben
Allium cepa L.	Amaryllidacea	ren	LF	Zwiebeln	top	DER	CCXLI / CLXIII	zeitiget er und bricht die geschwaer
Allium cepa L.	Amaryllidacea	ren	LF	Zwiebeln	top	DER	CCXLI / CLXIII	vertreibt weisse und schwarze Masen (?) am Leib
Allium cepa L.	Amaryllidacea	ren	LF	Zwiebeln	top	DER	CCXLI / CLXIII	Macht das Haar wiederum wachsen
Allium cepa L.	Amaryllidacea	ren	LF	Zwiebeln	top	DER	CCXLI / CLXIII	Arzney zu den Wunden, so von unsinnigen Hunden gebissen
Allium cepa L.	Amaryllidacea	ren	LF	Zwiebeln	top	EAR	CCXLI / CLXIII	Saft in die Ohren gethon bringt das Gehör und nimmt das Sausen im Kopf
Allium cepa L.	Amaryllidacea	ren	LF	Zwiebeln	top	EYE	CCXLI / CLXIII	macht lautere Augen, vertreibt den Anfang des Stars
Allium cepa L.	Amaryllidacea	ren	LF	Zwiebeln	top	GYN	CCXLI / CLXIII	bringt den Frawn ihre krankheyt
Allium cepa L.	Amaryllidacea	ren	LF	Zwiebeln	top	HUM	CCXLI / CLXIII	eröffnen die gulden oder rosen Ader
Allium cepa L.	Amaryllidacea	ren	LF	Zwiebeln	top	RES	CCXLI / CLXIII	reinigt das Haupt in die Nase getan
Allium cepa L.	Amaryllidacea	ren	LF	Zwiebeln	syst	URO	CCXLI / CLXIII	wohl gesotten treibt er den Harn
Allium cepa L.	Amaryllidacea	s pop	AV	Zwiebel	syst	CAR	p. 533	Arteriosklerose
Allium cepa L.	Amaryllidacea	s pop	AV	Zwiebel	top	CAR	p. 533	Herzstörungen (Wadenwickel)
Allium cepa L.	Amaryllidacea	s pop	AV	Zwiebel	top	DER	p. 419	Haarwuchsmittel
Allium cepa L.	Amaryllidacea	s pop	AV	Zwiebel	top	EAR	p. 533	Ohrenweh
Allium cepa L.	Amaryllidacea	s pop	AV	Zwiebel	top	EYE	p. 533	Augenentzündung
Allium cepa L.	Amaryllidacea	s pop	AV	Zwiebel	syst	FEV	p. 64	Fieber
Allium cepa L.	Amaryllidacea	s pop	AV	Zwiebel	top	FEV	p. 533	Fieber (Fusssohlenwickel)
Allium cepa L.	Amaryllidacea	s pop	AV	Zwiebel	top	GAS	p. 33	Krämpfe, Unwohlsein
Allium cepa L.	Amaryllidacea	s pop	AV	Zwiebel	syst	OTH	p. 353	Zuckerkrankheit
Allium cepa L.	Amaryllidacea	s pop	AV	Zwiebel	other	RES	p. 533	Fliessschnupfen
Allium cepa L.	Amaryllidacea	s pop	AV	Zwiebel	syst	RES	p. 33, 164	Schnupfen
Allium cepa L.	Amaryllidacea	s pop	AV	Zwiebel	vol	RES	p. 33	Schnupfen
Allium cepa L.	Amaryllidacea	s pop	AV	Zwiebel	syst	SKE	p. 533	Gichtkranke
Allium cepa L.	Amaryllidacea	s pop	AV	Zwiebel	top	TEE	p. 533	Zahnweh
Allium cepa L.	Amaryllidacea	s pop	AV	Zwiebel	other	URO	p. 533	Blasenkrampf usw.
Allium cepa L.	Amaryllidacea	s pop	AV	Zwiebel	syst	URO	p. 533	mild Harnausscheidend
Allium cepa L.	Amaryllidacea	s pop	AV	Zwiebel	top	URO	p. 533	Kopfweh, für die Nerven ein vorzügliches Heilmittel
Allium cepa L.	Amaryllidacea	s pop	JK	Zwiebel	top	EAR	p. 447	Ohrenentzündungen
Allium cepa L.	Amaryllidacea	s pop	JK	Zwiebel	top	EYE	p. 447	Augenentzündungen
Allium cepa L.	Amaryllidacea	s pop	JK	Zwiebel	top	NER	p. 447	Hinrentzündungen
Allium cepa L.	Amaryllidacea	s pop	JK	Zwiebel	syst	RES	p. 447	Erkältungen
Allium cepa L.	Amaryllidacea	s pop	JK	Zwiebel	top	RES	p. 447	Husten, Lungenkatarrh, Brustkatarrh, Lungenentzündung, Brustfellentzündung
Allium cepa L.	Amaryllidacea	s pop	JK	Zwiebel	top	URO	p. 447	Blasenkatarrh
Allium cepa L.	Amaryllidacea	s pop	UB	Zwiebel	syst	CAR	p. 389 / 405	Vorbeugung alertesbedingeter Gefässerkrankungen
Allium cepa L.	Amaryllidacea	s pop	UB	Zwiebel	syst	HUM	p. 462	Ausleitung und Regeneration
Allium cepa L.	Amaryllidacea	s pop	UB	Zwiebel	syst	HUM	p. 462	Ausleitung und Regeneration
Allium cepa L.	Amaryllidacea	s pop	UB	Zwiebel	syst	RES	p. 208 / 226	Bronchialerkrankungen / Asthma
Allium spp. (*A. sativum*, *A. ursinum*)	Amaryllidacea	ant	DIOS	Knoblauch	syst	ANT	153	von der Viper und von der Hämorrhois gebissen sind, wenn anhaltend Wein genom- men, oder wenn er (der Knoblauch) mit Wein zerrieben und getrunken wird.
Allium spp. (*A. sativum*, *A. ursinum*)	Amaryllidacea	ant	DIOS	Knoblauch	top	ANT	153	Ebenso wird er auch gegen den Biss des tollen Hundes mit Erfolg aufgelegt.
Allium spp. (*A. sativum*, *A. ursinum*)	Amaryllidacea	ant	DIOS	Knoblauch	top	ANT	153	nützlich gegen die nachtheilige Wirkung der Veränderung des Wassers
Allium spp. (*A. sativum*, *A. ursinum*)	Amaryllidacea	ant	DIOS	Knoblauch	top	DER	153	Sugillationen unter den Augen und die Fuchs- krankheit; die an Alopekie leiden; heilt er Hautausschlag; Mit Honig vertreibt er weiße Flecken, Flechten, Leberflecken, bösen Grind, Schorf und Aussatz
Allium spp. (*A. sativum*, *A. ursinum*)	Amaryllidacea	ant	DIOS	Knoblauch	syst	GAS	153	Gegessen treibt er den Bandwurm aus
Allium spp. (*A. sativum*, *A. ursinum*)	Amaryllidacea	ant	DIOS	Knoblauch	top	GYN	153	Abkochung der Dolde dient als Sitzbad zur Beförderung der Menstruation und der Nachgeburt. Zu demselben Zwecke wird er in der Räucherung angewandt
Allium spp. (*A. sativum*, *A. ursinum*)	Amaryllidacea	ant	DIOS	Knoblauch	vol	GYN	153	Abkochung der Dolde dient als Sitzbad zur Beförderung der Menstruation und der Nachgeburt. Zu demselben Zwecke wird er in der Räucherung angewandt
Allium spp. (*A. sativum*, *A. ursinum*)	Amaryllidacea	ant	DIOS	Knoblauch	top	n.d.	153	Mit Feigenblättern und römischem Kümmel ist er ein Umschlag gegen die Spitzmaus
Allium spp. (*A. sativum*, *A. ursinum*)	Amaryllidacea	ant	DIOS	Knoblauch	syst	OTH	153	getrunken tötet er Läuse und Wanzen
Allium spp. (*A. sativum*, *A. ursinum*)	Amaryllidacea	ant	DIOS	Knoblauch	syst	RES	153	Luftröhre (die Stimme) hell und bringt Linderung bei anhaltendem Husten
Allium spp. (*A. sativum*, *A. ursinum*)	Amaryllidacea	ant	DIOS	Knoblauch	top	TEE	153	beruhigt Zahnschmerzen, wenn sie im Munde behalten wird.
Allium spp. (*A. sativum*, *A. ursinum*)	Amaryllidacea	ant	DIOS	Knoblauch	syst	URO	153	befördert den Harn; treibt, genossen, den Harn und wirkt eröffnend. Er ist auch den Wassersüchtigen heilsam
Allium spp. (*A. sativum*, *A. ursinum*)	Amaryllidacea	mon	HvB	Knoblauch	syst	EYE	1-79	Augen werden rein
Allium spp. (*A. sativum*, *A. ursinum*)	Amaryllidacea	mon	MF	Knoblauch	syst	ANT	5	weder Trunk unbekannten Wassers, noch häufiger Wechsel verschiedener Aufenthaltsorte
Allium spp. (*A. sativum*, *A. ursinum*)	Amaryllidacea	mon	MF	Knoblauch	top	ANT	5	heilt Allium Stiche und Bisse, die Schlange und Skorpion dem Menschen beibringen, mit Hoing aufgelegt auch Hundebisse
Allium spp. (*A. sativum*, *A. ursinum*)	Amaryllidacea	mon	MF	Knoblauch	syst	ANT	5	heilt Allium Stiche und Bisse, die Schlange und Skorpion dem Menschen beibringen, mit Hoing aufgelegt auch Hundebisse

Allium spp. (*A. sativum* , *A. ursinum*)	Amaryllidacea	mon	MF	Knoblauch	syst	DER	5	unheilbringende Bisse heilen
Allium spp. (*A. sativum* , *A. ursinum*)	Amaryllidacea	mon	MF	Knoblauch	syst	DER	5	grosse Geschwülste
Allium spp. (*A. sativum* , *A. ursinum*)	Amaryllidacea	mon	MF	Knoblauch	top	EAR	5	ins Ohr, wenn es schmerzt
Allium spp. (*A. sativum* , *A. ursinum*)	Amaryllidacea	mon	MF	Knoblauch	syst	GAS	5	vertrebt es Darmwürmer, sowie Bandwürmer
Allium spp. (*A. sativum* , *A. ursinum*)	Amaryllidacea	mon	MF	Knoblauch	syst	GAS	5	hilft es gegen den Stuhlzwang
Allium spp. (*A. sativum* , *A. ursinum*)	Amaryllidacea	mon	MF	Knoblauch	syst	GAS	5	Praxagoras: Gelbssucht
Allium spp. (*A. sativum* , *A. ursinum*)	Amaryllidacea	mon	MF	Knoblauch	vol	GAS	5	durch den Duft werden schädliche Spulwürmer vertrieben
Allium spp. (*A. sativum* , *A. ursinum*)	Amaryllidacea	mon	MF	Knoblauch	syst	GYN	5	Hippokrates: mit dem Rauch die weiblich Scham erwärmt, die Nachgeburt herausgetrieben werden kann
Allium spp. (*A. sativum* , *A. ursinum*)	Amaryllidacea	mon	MF	Knoblauch	syst	NER	5	Schläfen damit bestreicht, lindert es Kopfschmerz
Allium spp. (*A. sativum* , *A. ursinum*)	Amaryllidacea	mon	MF	Knoblauch	syst	RES	5	hilft es gegen verschiedene Lungebeschwerden
Allium spp. (*A. sativum* , *A. ursinum*)	Amaryllidacea	mon	MF	Knoblauch	syst	RES	5	gesottn tut's dem Husten gut und stillt das Keuchen, roh wie gekockt macht es die rauhe Stimme klar
Allium spp. (*A. sativum* , *A. ursinum*)	Amaryllidacea	mon	MF	Knoblauch	syst	TON	5	erschöpften Leib gesund machen
Allium spp. (*A. sativum* , *A. ursinum*)	Amaryllidacea	mon	MF	Knoblauch	syst	URO	5	Wassersüchtigen, trocknet die wasserflüssigen Körpersäfte
Allium spp. (*A. sativum* , *A. ursinum*)	Amaryllidacea	mon	MF	Knoblauch	syst	URO	5	Blasenschmerz und Blasengeschwulst vermindert
Allium spp. (*A. sativum* , *A. ursinum*)	Amaryllidacea	mon	MF	Knoblauch	syst	URO	5	Nierenkranke
Allium spp. (*A. sativum* , *A. ursinum*)	Amaryllidacea	ren	LF	Knoblauch	syst	ANT	CCCCXXI / CCLXXXII	gut denen so von natern oder schlangen gebissen sind
Allium spp. (*A. sativum* , *A. ursinum*)	Amaryllidacea	ren	LF	Knoblauch	syst	ANT	CCCCXXI / CCLXXXII	denen so mancherley Wasser trinken müssen
Allium spp. (*A. sativum* , *A. ursinum*)	Amaryllidacea	ren	LF	Bärlauch	syst	ANT	CCCXXIIII/CCLXXXII	gut denen so von natern oder schlangen gebissen sind
Allium spp. (*A. sativum* , *A. ursinum*)	Amaryllidacea	ren	LF	Bärlauch	syst	ANT	CCCXXIIII/CCLXXXII	denen so mancherley Wasser trinken müssen
Allium spp. (*A. sativum* , *A. ursinum*)	Amaryllidacea	ren	LF	Knoblauch	top	ANT	CCCCXXI / CCLXXXII	gut denen so von einem wütenden Hund gebissen
Allium spp. (*A. sativum* , *A. ursinum*)	Amaryllidacea	ren	LF	Knoblauch	syst	APH	CCCCXXI / CCLXXXII	macht lust zu den eelichen wercken
Allium spp. (*A. sativum* , *A. ursinum*)	Amaryllidacea	ren	LF	Bärlauch	syst	APH	CCCXXIIII/CCLXXXII	macht lust zu den eelichen wercken
Allium spp. (*A. sativum* , *A. ursinum*)	Amaryllidacea	ren	LF	Knoblauch	syst	CAR	CCCCXXI / CCLXXXII	macht dünn gebluet und ein rot angesicht
Allium spp. (*A. sativum* , *A. ursinum*)	Amaryllidacea	ren	LF	Bärlauch	syst	CAR	CCCXXIIII/CCLXXXII	macht dünn gebluet und ein rot angesicht
Allium spp. (*A. sativum* , *A. ursinum*)	Amaryllidacea	ren	LF	Bärlauch	vol	DER	CCCXXIIII/CCLXXXII	vertreibt allerley grind, rauden, masen und flecken
Allium spp. (*A. sativum* , *A. ursinum*)	Amaryllidacea	ren	LF	Knoblauch	top	DER	CCCCXXI / CCLXXXII	vertreibt allerley grind, rauden, masen und flecken
Allium spp. (*A. sativum* , *A. ursinum*)	Amaryllidacea	ren	LF	Knoblauch	top	DER	CCCCXXI / CCLXXXII	vertreibt allerley grind, rauden, masen und flecken
Allium spp. (*A. sativum* , *A. ursinum*)	Amaryllidacea	ren	LF	Bärlauch	top	DER	CCCXXIIII/CCLXXXII	vertreibt allerley grind, rauden, masen und flecken
Allium spp. (*A. sativum* , *A. ursinum*)	Amaryllidacea	ren	LF	Bärlauch	top	DER	CCCXXIIII/CCLXXXII	gut denen so von einem wütenden Hund gebissen
Allium spp. (*A. sativum* , *A. ursinum*)	Amaryllidacea	ren	LF	Bärlauch	top	DER	CCCXXIIII/CCLXXXII	vertreibt allerley grind, rauden, masen und flecken
Allium spp. (*A. sativum* , *A. ursinum*)	Amaryllidacea	ren	LF	Knoblauch	syst	GAS	CCCCXXI / CCLXXXII	lindert den Stulgang
Allium spp. (*A. sativum* , *A. ursinum*)	Amaryllidacea	ren	LF	Knoblauch	syst	GAS	CCCCXXI / CCLXXXII	treibt aus die breiten Würmer
Allium spp. (*A. sativum* , *A. ursinum*)	Amaryllidacea	ren	LF	Bärlauch	syst	GAS	CCCXXIIII/CCLXXXII	lindert den Stulgang
Allium spp. (*A. sativum* , *A. ursinum*)	Amaryllidacea	ren	LF	Bärlauch	syst	GAS	CCCXXIIII/CCLXXXII	treibt aus die breiten Würmer
Allium spp. (*A. sativum* , *A. ursinum*)	Amaryllidacea	ren	LF	Knoblauch	vol	GYN	CCCCXXI / CCLXXXII	vertreibt allerley grind, rauden, masen und flecken
Allium spp. (*A. sativum* , *A. ursinum*)	Amaryllidacea	ren	LF	Knoblauch	syst	NER	CCCCXXI / CCLXXXII	macht neygung zum Schlaf
Allium spp. (*A. sativum* , *A. ursinum*)	Amaryllidacea	ren	LF	Bärlauch	syst	NER	CCCXXIIII/CCLXXXII	macht neygung zum Schlaf
Allium spp. (*A. sativum* , *A. ursinum*)	Amaryllidacea	ren	LF	Knoblauch	syst	OTH	CCCCXXI / CCLXXXII	tödt leüss und nissen
Allium spp. (*A. sativum* , *A. ursinum*)	Amaryllidacea	ren	LF	Bärlauch	syst	OTH	CCCXXIIII/CCLXXXII	tödt leüss und nissen
Allium spp. (*A. sativum* , *A. ursinum*)	Amaryllidacea	ren	LF	Knoblauch	syst	RES	CCCCXXI / CCLXXXII	macht helle stimm und lindert den alten Husten
Allium spp. (*A. sativum* , *A. ursinum*)	Amaryllidacea	ren	LF	Bärlauch	syst	RES	CCCXXIIII/CCLXXXII	macht helle stimm und lindert den alten Husten
Allium spp. (*A. sativum* , *A. ursinum*)	Amaryllidacea	ren	LF	Knoblauch	syst	URO	CCCCXXI / CCLXXXII	treibt den Harn
Allium spp. (*A. sativum* , *A. ursinum*)	Amaryllidacea	ren	LF	Bärlauch	syst	URO	CCCXXIIII/CCLXXXII	treibt den Harn
Allium spp. (*A. sativum* , *A. ursinum*)	Amaryllidacea	s pop	AV	Bärlauch	syst	CAR	. 39, 184, 195, 199	Arteriosklerose, hoher Blutdruck
Allium spp. (*A. sativum* , *A. ursinum*)	Amaryllidacea	s pop	AV	Bärlauch	syst	CAR	. 39, 184, 195, 199	Arteriosklerose, hoher Blutdruck
Allium spp. (*A. sativum* , *A. ursinum*)	Amaryllidacea	s pop	AV	Knoblauch	top	EAR	p. 532	Ohrenweh
Allium spp. (*A. sativum* , *A. ursinum*)	Amaryllidacea	s pop	AV	Bärlauch	syst	GAS	p. 462	Blähungsbeschwerden im Darm
Allium spp. (*A. sativum* , *A. ursinum*)	Amaryllidacea	s pop	AV	Bärlauch	syst	GAS	p. 462	Blähungsbeschwerden im Darm
Allium spp. (*A. sativum* , *A. ursinum*)	Amaryllidacea	s pop	AV	Knoblauch	syst	GAS	p. 45	Verstopfung
Allium spp. (*A. sativum* , *A. ursinum*)	Amaryllidacea	s pop	AV	Knoblauch	syst	GAS	p. 531	Verstopfung, Wurmtreibend
Allium spp. (*A. sativum* , *A. ursinum*)	Amaryllidacea	s pop	AV	Knoblauch	top	NER	p. 532	Kopfweh
Allium spp. (*A. sativum* , *A. ursinum*)	Amaryllidacea	s pop	AV	Knoblauch	syst	OTH	p. 820	Krebs
Allium spp. (*A. sativum* , *A. ursinum*)	Amaryllidacea	s pop	AV	Knoblauch	syst	OTH	p. 531	Lymphdrüsentätigkeit
Allium spp. (*A. sativum* , *A. ursinum*)	Amaryllidacea	s pop	AV	Bärlauch	syst	RES	p. 462	Bronchien zu viel Schleim absondern
Allium spp. (*A. sativum* , *A. ursinum*)	Amaryllidacea	s pop	AV	Bärlauch	syst	RES	p. 462	Bronchien zu viel Schleim absondern
Allium spp. (*A. sativum* , *A. ursinum*)	Amaryllidacea	s pop	AV	Knoblauch	top	TEE	p. 532	Zahnweh
Allium spp. (*A. sativum* , *A. ursinum*)	Amaryllidacea	s pop	AV	Bärlauch	syst	TON	p. 542, 601, 613	Vitamin Mangel im Frühling, Naturnahrung, Schondiät
Allium spp. (*A. sativum* , *A. ursinum*)	Amaryllidacea	s pop	JK	Knoblauch	top	ANT	p. 383	ziehen das Gift aus den Wunden, das durch Schlangen- oder Hundebiss entstanden sind
Allium spp. (*A. sativum* , *A. ursinum*)	Amaryllidacea	s pop	JK	Knoblauch	top	APO	p. 383	Zeiten ansteckender Krankheiten . . . Trage man Knoblauch bei sich; er wird sie neutralisieren
Allium spp. (*A. sativum* , *A. ursinum*)	Amaryllidacea	s pop	JK	Knoblauch	syst	CAR	p. 383	hohem Blutdruck
Allium spp. (*A. sativum* , *A. ursinum*)	Amaryllidacea	s pop	JK	Bärlauch	syst	DER	p. 330	Flechten, Eissen und Ausschlägen, Skrofulöse und Bleichsüchtige
Allium spp. (*A. sativum* , *A. ursinum*)	Amaryllidacea	s pop	JK	Knoblauch	top	DER	p. 383	Schuppen,
Allium spp. (*A. sativum* , *A. ursinum*)	Amaryllidacea	s pop	JK	Knoblauch	top	EAR	p. 383	Ohrenwehtropfen
Allium spp. (*A. sativum* , *A. ursinum*)	Amaryllidacea	s pop	JK	Bärlauch	syst	GAS	p. 330	Reinigung von Magen, Gedärmen und Blut
Allium spp. (*A. sativum* , *A. ursinum*)	Amaryllidacea	s pop	JK	Knoblauch	syst	GAS	p. 383	vertribt die Würmer
Allium spp. (*A. sativum* , *A. ursinum*)	Amaryllidacea	s pop	JK	Knoblauch	syst	OTH	p. 383	macht Ansteckungskeime unschädlich
Allium spp. (*A. sativum* , *A. ursinum*)	Amaryllidacea	s pop	JK	Knoblauch	syst	SKE	p. 383	Gicht und Rheumatimus, insbesondere die schmerzhafte Darmgicht
Allium spp. (*A. sativum* , *A. ursinum*)	Amaryllidacea	s pop	JK	Knoblauch	syst	URO	p. 383	Nierenstein
Allium spp. (*A. sativum* , *A. ursinum*)	Amaryllidacea	s pop	MT	Bärlauch	syst	CAR	p. 11	Arterienverkalkung, hoher Blutdruck, Erkrankungen der Herzkranzgefässe
Allium spp. (*A. sativum* , *A. ursinum*)	Amaryllidacea	s pop	MT	Bärlauch	syst	DER	p. 11	Blutreinigung bei chronisch unreiner Haut
Allium spp. (*A. sativum* , *A. ursinum*)	Amaryllidacea	s pop	MT	Bärlauch	top	DER	p. 11	Entschlackung und Reinigung
Allium spp. (*A. sativum* , *A. ursinum*)	Amaryllidacea	s pop	MT	Bärlauch	syst	GAS	p.11	wirkt sehr günstig auf das Magen-Darmsystem
Allium spp. (*A. sativum* , *A. ursinum*)	Amaryllidacea	s pop	MT	Bärlauch	syst	NER	p. 11	verhelfen zu einem ausgezeichneten Gedächtnis
Allium spp. (*A. sativum* , *A. ursinum*)	Amaryllidacea	s pop	MT	Bärlauch	syst	RES	p. 11	dauernder Brustverschleimung und damit verbundenen Atembeschwerden
Allium spp. (*A. sativum* , *A. ursinum*)	Amaryllidacea	s pop	MT	Bärlauch	syst	URO	p. 11	Lungen- und Wassersucht, reinigen Niere und Blase und fördern den Harnabgang
Allium spp. (*A. sativum* , *A. ursinum*)	Amaryllidacea	s pop	UB	Bärlauch	syst	CAR	p. 387 / 400 / 405	Bluthochdruck / Arteriosklerose / arterielle Durchblutungsstörung
Allium spp. (*A. sativum* , *A. ursinum*)	Amaryllidacea	s pop	UB	Knoblauch	syst	CAR	p. 387 / 400 / 405	Bluthochdruck / Arteriosklerose / arterielle Durchblutungsstörung
Allium spp. (*A. sativum* , *A. ursinum*)	Amaryllidacea	s pop	UB	Bärlauch	syst	GAS	p. 452 / 462	Darmsanierung (Ausleitung / Regeneration)
Allium spp. (*A. sativum* , *A. ursinum*)	Amaryllidacea	s pop	UB	Knoblauch	syst	HUM	p. 454 / 462	Ausleiten und Regeneration
Allium spp. (*A. sativum* , *A. ursinum*)	Amaryllidacea	s pop	UB	Knoblauch	syst	HUM	p. 454 / 462	Ausleiten und Regeneration
Allium spp. (*A. sativum* , *A. ursinum*)	Amaryllidacea	s pop	UB	Knoblauch	syst	HUM	p. 454 / 462	Ausleiten und Regeneration
Allium spp. (*A. sativum* , *A. ursinum*)	Amaryllidacea	s pop	UB	Knoblauch	syst	HUM	p. 454 / 462	Ausleiten und Regeneration
Allium spp. (*A. sativum* , *A. ursinum*)	Amaryllidacea	s pop	UB	Knoblauch	syst	RES	p. 207	gegen Grippeviren
Allium spp. (*A. sativum* , *A. ursinum*)	Amaryllidacea	s pop	WI	Bärlauch	syst	CAR	p. 48	Volksheilkunde: wie Knoblauch Antihypertonikum und Antiarteriosklerotikum
Allium spp. (*A. sativum* , *A. ursinum*)	Amaryllidacea	s pop	WI	Bärlauch	syst	GAS	p. 48	Volksheilkunde: wie Knoblauch Magendarmstörungen
Allium spp. (*A. sativum* , *A. ursinum*)	Amaryllidacea	sci	ESCOP	Allii sativi bulbus	syst	CAR	I p.14	treatment of elevated blood lipid levels
Allium spp. (*A. sativum* , *A. ursinum*)	Amaryllidacea	sci	ESCOP	Allii sativi bulbus	syst	RES	I p.14	upper respiratory tract infection
Allium spp. (*A. sativum* , *A. ursinum*)	Amaryllidacea	sci	ESCOP	Allii sativi bulbus	syst	SKE	I p.14	prophylaxis of arteriosclerosis
Althaea officinalis L.	Malvaceae	ant	DIOS	Eibisch	syst	ANT	208	Trank gegen die Stiche der Bienen und aller kleinen Thiere
Althaea officinalis L.	Malvaceae	ant	DIOS	Eibisch	top	ANT	208	Schutzmittel gegen den Biss giftiger Thiere
Althaea officinalis L.	Malvaceae	ant	DIOS	Eibisch	top	DER	208	bringt weisse Flecken weg
Althaea officinalis L.	Malvaceae	ant	DIOS	Eibisch	top	DER	208	bei Verwundungen und Brandwunden
Althaea officinalis L.	Malvaceae	ant	DIOS	Eibisch	syst	DER	208	Wunden, Drüsen an den Ohren und am Halse, bei Abscessen am After, entzün- deten Brüsten
Althaea officinalis L.	Malvaceae	ant	DIOS	Eibisch	syst	GAS	208	Dysenterie, Blutauswurf und Durchfall
Althaea officinalis L.	Malvaceae	ant	DIOS	Eibisch	syst	GAS	208	Dysenterie
Althaea officinalis L.	Malvaceae	ant	DIOS	Eibisch	top	GYN	208	Zäpfchen gegen Entzündung und Verstopfung der Gebärmutter, Lochien
Althaea officinalis L.	Malvaceae	ant	DIOS	Eibisch	syst	n.d.	208	und inneren Rupturen
Althaea officinalis L.	Malvaceae	ant	DIOS	Eibisch	n.d.	n.d.	208	verdickt die Wurzel auch das Wasser
Althaea officinalis L.	Malvaceae	ant	DIOS	Eibisch	syst	NER	208	Ischias, Zittern
Althaea officinalis L.	Malvaceae	ant	DIOS	Eibisch	syst	SKE	208	Emphysem und Sehnenspannung.
Althaea officinalis L.	Malvaceae	ant	DIOS	Eibisch	top	TEE	208	Zahnschmerzen lindert sie mit Essig gekocht als Mundspülwasser
Althaea officinalis L.	Malvaceae	ant	DIOS	Eibisch	syst	URO	208	hilft bei Harnverhaltung, gegen die Beschwerden der Steinkranken
Althaea officinalis L.	Malvaceae	mon	HvB	Eibisch	syst	FEV	1-141	gegen Fieber

Althaea officinalis L.	Malvaceae	mon	HvB	Eibisch	top	NER	1-141	Kopfweh
Althaea officinalis L.	Malvaceae	mon	MF	Eibisch	syst	ANT	9	vermag Giften zu widerstehen
Althaea officinalis L.	Malvaceae	mon	MF	Eibisch	top	DER	9	heilt verderbenbringende Tierbisse, sowie verbrannte Glieder
Althaea officinalis L.	Malvaceae	mon	MF	Eibisch	top	DER	9	vertreibt entstellende Leberflecken
Althaea officinalis L.	Malvaceae	mon	MF	Eibisch	syst	DER	9	reinigt Wunden, vertreibt böse Halsdrüsen, beruhigt schmerzenden After, heilt auch Prellungen
Althaea officinalis L.	Malvaceae	mon	MF	Eibisch	syst	DER	9	beringt verhärtete Eitergeschwüre zum Aufplatzen
Althaea officinalis L.	Malvaceae	mon	MF	Eibisch	syst	GAS	9	bei Blutstuhl leidenden behebt sie den Durchfall
Althaea officinalis L.	Malvaceae	mon	MF	Eibisch	syst	GYN	9	heilt sie Geschwülste und andere Beschwerden der Gebärmutter
Althaea officinalis L.	Malvaceae	mon	MF	Eibisch	syst	GYN	9	treibt zögernde Nachgeburt hinaus
Althaea officinalis L.	Malvaceae	mon	MF	Eibisch	syst	RES	9	Blutspeienden
Althaea officinalis L.	Malvaceae	mon	MF	Eibisch	syst	SKE	9	entspannt Muskeln
Althaea officinalis L.	Malvaceae	mon	MF	Eibisch	syst	URO	9	treibt Steine aus und leistet bei verchiedenen Blasenleiden Hilfe
Althaea officinalis L.	Malvaceae	ren	LF	Eibisch	syst	ANT	VII / V	heylet alle stich der Immen und gergleichen gethier
Althaea officinalis L.	Malvaceae	ren	LF	Eibisch	top	DER	VII / V	die bletter mit oel vermischt sollen auf die bisse . . . gelegt werden
Althaea officinalis L.	Malvaceae	ren	LF	Eibisch	top	DER	VII / V	vertreibt schwarze und weisse Masen under dem Angesicht
Althaea officinalis L.	Malvaceae	ren	LF	Eibisch	syst	DER	VII / V	heylet wunden, ohrmützel, kroepff, beulen, entzündungen der Brüst, zerknirschung des afterdarms
Althaea officinalis L.	Malvaceae	ren	LF	Eibisch	syst	GAS	VII / V	rote ruhr
Althaea officinalis L.	Malvaceae	ren	LF	Eibisch	syst	GYN	VII / V	treibt aus das Bürdlin und andere Überflüssigkeit nach der Geburt
Althaea officinalis L.	Malvaceae	ren	LF	Eibisch	top	GYN	VII / V	Entzündung der Mutter
Althaea officinalis L.	Malvaceae	ren	LF	Eibisch	top	TEE	VII / V	lindert das Zanwee
Althaea officinalis L.	Malvaceae	ren	LF	Eibisch	syst	URO	VII / V	treibt den harn und den stein
Althaea officinalis L.	Malvaceae	s pop	JK	Eibisch	syst	DER	p. 345	inneren Geschwulsten
Althaea officinalis L.	Malvaceae	s pop	JK	Eibisch	top	DER	p. 345	Halsschmerzen
Althaea officinalis L.	Malvaceae	s pop	JK	Eibisch	top	DER	p. 345	Verbrennungen
Althaea officinalis L.	Malvaceae	s pop	JK	Eibisch	top	EAR	p. 345	Ohrenschmerzen
Althaea officinalis L.	Malvaceae	s pop	JK	Eibisch	syst	RES	p. 345	heftigem Schnupfen, Hustensirup
Althaea officinalis L.	Malvaceae	s pop	JK	Eibisch	syst	URO	p. 345	mildert den Brand in der Blase und behebt das Wasserbrennen
Althaea officinalis L.	Malvaceae	s pop	KN	Stockrose	vol	EAR	p. 478	Ohrendämpfe
Althaea officinalis L.	Malvaceae	s pop	KN	Eibisch	syst	GAS	p. 463	Magengeschwür
Althaea officinalis L.	Malvaceae	s pop	KN	Stockrose	syst	RES	p. 478	Halsgebrechen und Verschleimung auf der Brust
Althaea officinalis L.	Malvaceae	s pop	KN	Stockrose	vol	RES	p. 478	Dämpfe zum Einatmen
Althaea officinalis L.	Malvaceae	s pop	KN	Eibisch	syst	RES	p. 463	Erkältung, Husten und Heiserkeit
Althaea officinalis L.	Malvaceae	s pop	UB	Eibisch	syst	GAS	p. 163	Sodbrennen, Gastritis, Magen- und Zwölffingerdarmgeschwür
Althaea officinalis L.	Malvaceae	s pop	UB	Eibisch	syst	GAS	p. 163	Sodbrennen, Gastritis, Magen- und Zwölffingerdarmgeschwür
Althaea officinalis L.	Malvaceae	s pop	UB	Eibisch	syst	RES	p. 163	Reizhusten
Althaea officinalis L.	Malvaceae	s pop	UB	Eibisch	syst	RES	p. 163	Reizhusten
Althaea officinalis L.	Malvaceae	s pop	WI	Eibisch	top	DER	p. 55	Volksmedizin: Insektenstiche
Althaea officinalis L.	Malvaceae	s pop	WI	Eibisch	syst	GAS	p. 56	Volksmedizin: Diarrhöe
Althaea officinalis L.	Malvaceae	sci	EMA	Eibisch		GAS	/HMPC/98717/2008	mild gastrointestinal discomfort
Althaea officinalis L.	Malvaceae	sci	EMA	Eibisch		RES	/HMPC/98717/2008	oral or pharyngeal irritation and associated dry cough
Althaea officinalis L.	Malvaceae	sci	EMA	Eibisch		RES	/HMPC/98717/2008	oral or pharyngeal irritation and associated dry cough
Althaea officinalis L.	Malvaceae	sci	EMA	Eibisch		RES	/HMPC/98717/2008	oral or pharyngeal irritation and associated dry cough
Althaea officinalis L.	Malvaceae	sci	ESCOP	Althaeae radix	syst	RES	I p.32	dry cough and oral or pharyngeal irritation
Althaea officinalis L.	Malvaceae	sci	WI	Eibisch	top	DER	p. 56	Verbrennungen der Haut, Entzündungen im Enddarm (Proktitis)
Althaea officinalis L.	Malvaceae	sci	WI	Eibisch	syst	GAS	p. 55	Schleimhautentzündungen im Magen- Darmbereich
Althaea officinalis L.	Malvaceae	sci	WI	Eibisch	syst	GAS	p. 56	Gastroenteritis
Althaea officinalis L.	Malvaceae	sci	WI	Eibisch	syst	GYN	p. 56	Zystitis, Fluor albus
Althaea officinalis L.	Malvaceae	sci	WI	Eibisch	syst	RES	p. 55	Reizhusten, Katharrhen im Mund- und Rachenraum
Anagallis arvensis agg.	Primulaceae	ant	DIOS	Gauchheil	syst	ANT	162	Viper gebissen sind,heilsam
Anagallis arvensis agg.	Primulaceae	ant	DIOS	Gauchheil	n.d.	DER	162	ziehen Splitter aus und halten fressende Geschwüre auf; Mastdarmvorfall (blaublütige Anagallis)
Anagallis arvensis agg.	Primulaceae	ant	DIOS	Gauchheil	top	EYE	162	vertreibt er weiße Flecken auf dem Auge und hilft gegen Stumpfsichtigkeit
Anagallis arvensis agg.	Primulaceae	ant	DIOS	Gauchheil	syst	GAS	162	Leberkranken
Anagallis arvensis agg.	Primulaceae	ant	DIOS	Gauchheil	n.d.	OTH	162	sie besänftigen Entzündungen
Anagallis arvensis agg.	Primulaceae	ant	DIOS	Gauchheil	top	RES	162	Ihr Saft, zum Gurgeln gebraucht, fahrt den Schleim aus dem Kopfe ab
Anagallis arvensis agg.	Primulaceae	ant	DIOS	Gauchheil	top	TEE	162	lindert, in die Nase gebracht, Zahnschmerzen, wenn er in das dem leidenden Zahne, gegenüber stehende Nasenloch gegeben wird.
Anagallis arvensis agg.	Primulaceae	ant	DIOS	Gauchheil	syst	URO	162	Nierenkranken
Anagallis arvensis agg.	Primulaceae	mon	LO	Ackergaucheil	syst	OTH		
Anagallis arvensis agg.	Primulaceae	ren	LF	Ackergaucheil	syst	ANT	VIII + IX / VI	heilet die Naterbiss
Anagallis arvensis agg.	Primulaceae	ren	LF	Ackergaucheil	n.d.	DER	VIII + IX / VI	das Weiblein treibt hindersich den ausgetruckten affterdarm, aber das menlein treibt und zeücht den selbigen heraus.
Anagallis arvensis agg.	Primulaceae	ren	LF	Ackergaucheil	top	DER	VIII + IX / VI	legen den Schmerzen, seübern, ziehen aus Dorn und Seissen über gelegt. Seind gut den unreynen wunden dass sie seübern dieselbigen und heylen
Anagallis arvensis agg.	Primulaceae	ren	LF	Ackergaucheil	top	EYE	VIII + IX / VI	in die trüben Augen gelassen macht dieselbigen klar und lauter
Anagallis arvensis agg.	Primulaceae	ren	LF	Ackergaucheil	syst	GAS	VIII + IX / VI	ist gut zu der bresthafitgen Leber
Anagallis arvensis agg.	Primulaceae	ren	LF	Ackergaucheil	syst	TEE	VIII + IX / VI	lindert das Zanwee
Anagallis arvensis agg.	Primulaceae	ren	LF	Ackergaucheil	syst	URO	VIII + IX / VI	ist gut zu der [bresthafitgen Leber] und Nieren
Anagallis arvensis agg.	Primulaceae	S pop	VAL	Gauchheil	syst	CAR	p. 373	hémorroides
Anagallis arvensis agg.	Primulaceae	S pop	VAL	Gauchheil	top	DER	p. 373	plaies fongueuses, torpides, ulcères, dartres, prurit
Anagallis arvensis agg.	Primulaceae	S pop	VAL	Gauchheil	syst	GAS	p. 373	troubles hépatiques, ictère
Anagallis arvensis agg.	Primulaceae	S pop	VAL	Gauchheil	syst	NER	p. 373	épilepsie. Trouble mentaux, mélancolie
Anagallis arvensis agg.	Primulaceae	S pop	VAL	Gauchheil	syst	RES	p. 373	stases bronchiques, coryza spasmodique
Anagallis arvensis agg.	Primulaceae	S pop	VAL	Gauchheil	syst	URO	p. 373	néphrite, lithiase urinaire
Anethum graveolens L.	Apiaceae	ant	DIOS	Dill	top	DER	185	gebrannte Same als Umschlag vertreibt Geschwülste (Kondylome) am After.
Anethum graveolens L.	Apiaceae	ant	DIOS	Dill	top	FEV	84	wirksam gegen die Wechselfieber
Anethum graveolens L.	Apiaceae	ant	DIOS	Dill	syst	GAS	184	lindert Leibschneiden und Blähungen, reinigt den Bauch und stillt leichtes Erbrechen, ... beruhigt den Schlucken
Anethum graveolens L.	Apiaceae	ant	DIOS	Dill	syst	GYN	184	befördert als Trank die Milchabsonderung
Anethum graveolens L.	Apiaceae	ant	DIOS	Dill	top	GYN	185	Abkochung als Sitzbad für hysterische Frauen
Anethum graveolens L.	Apiaceae	ant	DIOS	Dill	top	GYN	84	Kraft, die Gebärmuttergegend zu erweichen und zu eröffnen
Anethum graveolens L.	Apiaceae	ant	DIOS	Dill	syst	OTH	184	bei anhaltendem Genuß aber schwächt sie das Gesicht und unterdrückt die Zeugungskraft
Anethum graveolens L.	Apiaceae	ant	DIOS	Dill	top	SKE	84	gegen Gelenkschmerzen
Anethum graveolens L.	Apiaceae	ant	DIOS	Dill	syst	URO	184	treibt den Harn
Anethum graveolens L.	Apiaceae	mon	HvB	Dill	syst	SKE	1-67	unterdrückt die Gicht
Anethum graveolens L.	Apiaceae	mon	MF	Dill	top	DER	10	frisst wucherndes Fleisch in einer Wunde weg und heilt kriechend fressende Geschwüre, Hämorrhoiden, löst auch Feigwarzen auf
Anethum graveolens L.	Apiaceae	mon	MF	Dill	top	EYE	10	beseitigen Hitze und Brennen der Augen
Anethum graveolens L.	Apiaceae	mon	MF	Dill	syst	GAS	10	vetreibt alle Magenleiden, bedrückender Brechreiz beruhigt
Anethum graveolens L.	Apiaceae	mon	MF	Dill	vol	GAS	10	drängt den Schluckauf zurück
Anethum graveolens L.	Apiaceae	mon	MF	Dill	syst	GYN	10	Muttermilch bis zum Überfluss
Anethum graveolens L.	Apiaceae	mon	MF	Dill	top	GYN	10	hilft der Gebärmutter
Anethum graveolens L.	Apiaceae	mon	MF	Dill	top	RES	10	Rachenzäpfchen aufgeschwollen
Anethum graveolens L.	Apiaceae	mon	MF	Dill	top	SKE	10	Schäden welche durch Frost entstanden sind, Kopfschmerz zähmt, Spannungen der Muskeln löst und Frostzuständen abhilft.
Anethum graveolens L.	Apiaceae	mon	MF	Dill	top	URO	10	treibt den Harn
Anethum graveolens L.	Apiaceae	ren	LF	Dill	top	DER	XVI / IX	heylet die knoepff oder runzel am hindern
Anethum graveolens L.	Apiaceae	ren	LF	Dill	top	DER	XVI / IX	zeitigt die groben Geschwulst, gut zu den feuchten geschwaeren und in sonderheyt heylet es die geschwaer an den heimlichen Orten
Anethum graveolens L.	Apiaceae	ren	LF	Dill	syst	GAS	XVI / IX	stelt das Würgen und den Stulgang, stillet das Grimmen, mildert das hergen (?) und aufstossen des Magens
Anethum graveolens L.	Apiaceae	ren	LF	Dill	syst	GYN	XVI / IX	bringen den Frawen die versiegene Milch wider
Anethum graveolens L.	Apiaceae	ren	LF	Dill	syst	URO	XVI / IX	treibt den Harn
Anethum graveolens L.	Apiaceae	ren	LF	Dill	top	URO	XVI / IX	benimmt das Aufsteigen der Mutter
Anethum graveolens L.	Apiaceae	s pop	AV	Dill	syst	GAS	p. 83	Verdauungs- und Stoffwechselstörungen von kleinen Kindern

Anethum graveolens L.	Apiaceae	s pop	AV	Dill	syst	GYN	p. 529	Förderung der Milch
Anethum graveolens L.	Apiaceae	s pop	AV	Dill	syst	RES	p. 529	Erkältung
Anethum graveolens L.	Apiaceae	s pop	UB	Dill	syst	NER	p. 420	Kinderschlafmittel
Anethum graveolens L.	Apiaceae	s pop	UB	Dill	syst	NER	p. 420	Kinderschlafmittel
Arctium lappa agg.	Asteraceae	ant	DIOS	Klette	top	DER	240	Blätter werden mit Nutzen auf alte Wunden
Arctium lappa agg.	Asteraceae	ant	DIOS	Klette	syst	RES	240	Blutspeien und Lungengeschwüren
Arctium lappa agg.	Asteraceae	ant	DIOS	Klette	top	SKE	240	lindert sie die von Verrenkungen herrührenden Gliederschmerzen
Arctium lappa agg.	Asteraceae	mon	HvB	Klette	top	DER	1-98	Grind auf dem Kopf
Arctium lappa agg.	Asteraceae	mon	HvB	Klette	syst	URO	1-98	Stein im Körper wächst
Arctium lappa agg.	Asteraceae	ren	LF	Grosse Klette	top	ANT	XL / XXIII	von den Natern , wütenden Hunden und anderen gifftigen thiern gebissen sind
Arctium lappa agg.	Asteraceae	ren	LF	Grosse Klette	top	ANT	XL / XXIII	von den Natern , wütenden Hunden und anderen gifftigen thiern gebissen sind
Arctium lappa agg.	Asteraceae	ren	LF	Grosse Klette	top	DER	XL / XXIII	heylen den Brandt
Arctium lappa agg.	Asteraceae	ren	LF	Grosse Klette	top	DER	XL / XXIII	vertreibet die Kroepff
Arctium lappa agg.	Asteraceae	ren	LF	Grosse Klette	syst	RES	XL / XXIII	ist ein köstlich Arzney, denen so Blut und Eyter auswerfen
Arctium lappa agg.	Asteraceae	ren	LF	Grosse Klette	top	SKE	XL / XXIII	alten Schaeden
Arctium lappa agg.	Asteraceae	ren	LF	Grosse Klette	top	SKE	XL / XXIII	zerbrechung oder zerknütschung der beyn und glider
Arctium lappa agg.	Asteraceae	s pop	UB	Grosse Klette	top	DER	p. 366	Haarpflege, Haarausfall
Arctium lappa agg.	Asteraceae	s pop	UB	Grosse Klette	top	DER	p. 366	Haarpflege, Haarausfall
Arctium lappa agg.	Asteraceae	s pop	WI	Grosse Klette	top	DER	p. 99	Volksmedizin: schlecht heilende Wunden, Ekzeme
Arctium lappa agg.	Asteraceae	s pop	WI	Grosse Klette	syst	GAS	p. 99	Volksmedizin: Erkrankungen und Beschwerden des Magen-Darmtraktes
Arctium lappa agg.	Asteraceae	s pop	WI	Grosse Klette	syst	SKE	p. 99	Volksmedizin: Gicht und Rheumatischen Beschwerden
Arctium lappa agg.	Asteraceae	s pop	WI	Grosse Klette	syst	URO	p. 99	Volksmedizin: Diuretikum (Blutreinigungsmittel), Blasensteinleiden
Arctium lappa agg.	Asteraceae	sci	EMA	Klettenwurzel	syst	URO	HPMC/246763/2009	increase the amount of urine to achieve flushing of urinary tract as an adjuvant in minor urinary tract complaints
Arctium lappa agg.	Asteraceae	sci	EMA	Klettenwurzel	syst	URO	HPMC/246763/2009	increase the amount of urine to achieve flushing of urinary tract as an adjuvant in minor urinary tract complaints
Arctium lappa agg.	Asteraceae	sci	WI	Grosse Klette	top	DER	p. 99	Klettenwurzelöl gegen trockene Seborrhoe der Kopfhaut
Artemisia abrotanum L.	Asteraceae	ant	DIOS	Eberraute	syst	ANT	175	Gegengift gegen tödliche Gifte; mit Wein genommen hilft er auch gegen ihre Bisse. Besonders ist er von guter Wirkung gegen Spinnen- und Skorpionstiche;
Artemisia abrotanum L.	Asteraceae	ant	DIOS	Eberraute	top	DER	175	zerteilt er Geschwülste
Artemisia abrotanum L.	Asteraceae	ant	DIOS	Eberraute	top	EYE	175	Heilmittel bei Augenentzündungen
Artemisia abrotanum L.	Asteraceae	ant	DIOS	Eberraute	top	FEV	175	eine Salbe gegen Frostschauer
Artemisia abrotanum L.	Asteraceae	ant	DIOS	Eberraute	syst	GYN	175	Zurückbleiben der Menstruation
Artemisia abrotanum L.	Asteraceae	ant	DIOS	Eberraute	vol	OTH	175	Als Lagerstreu und als Räucherung angezündet verscheucht er die Schlangen
Artemisia abrotanum L.	Asteraceae	ant	DIOS	Eberraute	syst	OTH	175	Orthopnöe
Artemisia abrotanum L.	Asteraceae	ant	DIOS	Eberraute	syst	SKE	175	innere Rupturen, Krämpfe, Ischias
Artemisia abrotanum L.	Asteraceae	ant	DIOS	Eberraute	syst	URO	175	Harnverhaltung
Artemisia abrotanum L.	Asteraceae	mon	HvB	Eberraute	top	DER	1-106	Grind
Artemisia abrotanum L.	Asteraceae	mon	HvB	Eberraute	top	DER	1-106	Beulen, Glieder im Menschen zusammengezogen
Artemisia abrotanum L.	Asteraceae	mon	HvB	Eberraute	top	SKE	1-106	Gicht
Artemisia abrotanum L.	Asteraceae	mon	MF	Eberraute	syst	ANT	2	Geruch vertreibt Schlangen, wenn man es trinkt, nimmt's deren Gift seine Kraft
Artemisia abrotanum L.	Asteraceae	mon	MF	Eberraute	syst	APH	2	Liebeskraft regt es an, sobald du's unters Kissen legts
Artemisia abrotanum L.	Asteraceae	mon	MF	Eberraute	syst	EYE	2	Augenschmerzen, Augenbrennen
Artemisia abrotanum L.	Asteraceae	mon	MF	Eberraute	syst	FEV	2	stillt es die Fieberfröste, noch bevor sie kommen
Artemisia abrotanum L.	Asteraceae	mon	MF	Eberraute	syst	GAS	2	tötet Spulwürmer
Artemisia abrotanum L.	Asteraceae	mon	MF	Eberraute	syst	GYN	2	hilft es den Lenden und allen Beschwerden der weiblichen Scham
Artemisia abrotanum L.	Asteraceae	mon	MF	Eberraute	syst	GYN	2	sorgt für geordneten Monatsfluss
Artemisia abrotanum L.	Asteraceae	mon	MF	Eberraute	syst	HUM	2	purgiert durch den Harn und reinigt Herz und Brust
Artemisia abrotanum L.	Asteraceae	mon	MF	Eberraute	syst	RES	2	hilft es allen Krankheitsfällen in der Brust, Atemnot, drängt Husten zurück
Artemisia abrotanum L.	Asteraceae	mon	MF	Eberraute	syst	SKE	2	hilft es den Muskeln
Artemisia abrotanum L.	Asteraceae	mon	MF	Eberraute	syst	SKE	2	tut gut bei Hüftgicht
Artemisia abrotanum L.	Asteraceae	ren	LF	Eberraute	syst	ANT	III / II	ist krefftig wider allerley tödtlich Gifft
Artemisia abrotanum L.	Asteraceae	ren	LF	Eberraute	n.d.	APH	III / II	bringt Lust zu den Frawn und vertreibt auch allerley Zauberey so disen Lust und begird verhindern.
Artemisia abrotanum L.	Asteraceae	ren	LF	Eberraute	top	DER	III / II	gut den so frostig seind und welchen die haut schaudert
Artemisia abrotanum L.	Asteraceae	ren	LF	Eberraute	top	DER	III / II	für das ausfallen der Haar
Artemisia abrotanum L.	Asteraceae	ren	LF	Eberraute	n.d.	GAS	III / II	töted die Würm im Leib
Artemisia abrotanum L.	Asteraceae	ren	LF	Eberraute	syst	GYN	III / II	bringen auch den Frawn ihr Krankheyt
Artemisia abrotanum L.	Asteraceae	ren	LF	Eberraute	syst	RES	III / II	den keichenden
Artemisia abrotanum L.	Asteraceae	ren	LF	Eberraute	syst	SKE	III / II	gebrochenen, weetagen der hüfft
Artemisia abrotanum L.	Asteraceae	ren	LF	Eberraute	syst	URO	III / II	und denen so schwaerlich harnen
Artemisia abrotanum L.	Asteraceae	s pop	VAL	Eberraute	syst	GAS	p. 160	digestion pénibles
Artemisia abrotanum L.	Asteraceae	s pop	VAL	Eberraute	syst	GYN	p. 160	menstruation difficiles
Artemisia abrotanum L.	Asteraceae	s pop	VAL	Eberraute	syst	OTH	p. 160	parasites intestinaux
Artemisia abrotanum L.	Asteraceae	s pop	VAL	Eberraute	top	URO	p. 160	und denen so schwaerlich harnen
Artemisia absinthium L.	Asteraceae	ant	DIOS	Wermut	syst	ANT	173	gegen die verderbliche Wirkung von (giftigen) Pilzen, mit Wein aber gegen Ixia und Schierling, gegen den Biß der Spitzmaus und den Meerdrachen
Artemisia absinthium L.	Asteraceae	ant	DIOS	Wermut	top	DER	173	Gegen Schlundmuskelentzündung, gegen Epinyktiden mit Wasser, gegen Sugillationen unter den Augen
Artemisia absinthium L.	Asteraceae	ant	DIOS	Wermut	top	EAR	173	eiterflüssige Ohren
Artemisia absinthium L.	Asteraceae	ant	DIOS	Wermut	vol	EAR	173	Ohrenschmerzen
Artemisia absinthium L.	Asteraceae	ant	DIOS	Wermut	top	EYE	173	gegen Stumpfsichtigkeit
Artemisia absinthium L.	Asteraceae	ant	DIOS	Wermut	top	EYE	173	schmerzhafte Augen
Artemisia absinthium L.	Asteraceae	ant	DIOS	Wermut	syst	GAS	173	Verdauung zu befördern, und Magen und Bauch von hineingedrungenen galligen Stoffen zu reinigen; gutes Mittel gegen Blähungen und Bauch- und Magenschmerzen, auch heilt der Aufguß oder die Abkochung davon Appetitlosigkeit und Gelbsucht
Artemisia absinthium L.	Asteraceae	ant	DIOS	Wermut	top	GAS	173	Unterleibs-, gegen Leber- und Magenschmerzen, auch wenn sie chronische Leiden sind
Artemisia absinthium L.	Asteraceae	ant	DIOS	Wermut	syst	GYN	173	befördert es die Katamenien
Artemisia absinthium L.	Asteraceae	ant	DIOS	Wermut	syst	GYN	173	befördert es die Katamenien
Artemisia absinthium L.	Asteraceae	ant	DIOS	Wermut	syst	OTH	173	in den Schränken aufgehängt, die Kleider vor Mottenfraß zu schützen und mit Öl zusammen als Salbe die Mücken abzuhalten, so daß sie den Körper nicht belästigen
Artemisia absinthium L.	Asteraceae	ant	DIOS	Wermut	syst	OTH	173	Wasser- und Milzsüchtigen bringt es Besserung (fieberfreiheit)
Artemisia absinthium L.	Asteraceae	ant	DIOS	Wermut	top	OTH	173	Wasser- und Milzsüchtigen bringt es Besserung
Artemisia absinthium L.	Asteraceae	ant	DIOS	Wermut	syst	OTH	173	verhindert, wenn es vorher genommen wird, den Rausch
Artemisia absinthium L.	Asteraceae	ant	DIOS	Wermut	vol	TEE	173	Zahnschmerzen
Artemisia absinthium L.	Asteraceae	ant	DIOS	Wermut	syst	TON	173	daß er der Gesundheit zuträglich sei
Artemisia absinthium L.	Asteraceae	ant	DIOS	Wermut	syst	URO	173	Wasser- und Milzsüchtigen bringt es Besserung (fieberfreiheit)
Artemisia absinthium L.	Asteraceae	ant	DIOS	Wermut	syst	URO	173	treibt den Harn
Artemisia absinthium L.	Asteraceae	ant	DIOS	Wermut	top	URO	173	Wasser- und Milzsüchtigen bringt es Besserung
Artemisia absinthium L.	Asteraceae	mon	HvB	Beifuss	syst	GAS	1-107	heilt er kranke Eingeweide, und er wärmt den kranken Magen
Artemisia absinthium L.	Asteraceae	mon	HvB	Wermut	syst	HUM	1-109	unterdrückt die Melancholie, macht dieAugen klar, sstärkt das Herz, und lässt nicht zu dass die Lunge krank wird, wärmt den Magen und renigt die Eingeweid, bereitet gute Verdauung
Artemisia absinthium L.	Asteraceae	mon	HvB	Wermut	top	NER	1-109	Kopfschmerzen
Artemisia absinthium L.	Asteraceae	mon	HvB	Wermut	top	RES	1-109	Schmerzen in er Brust, so dass er husest
Artemisia absinthium L.	Asteraceae	mon	HvB	Wermut	top	RES	1-109	um die Brust schmerzen leidet und hustet
Artemisia absinthium L.	Asteraceae	mon	HvB	Wermut	top	SKE	1-109	Gicht
Artemisia absinthium L.	Asteraceae	mon	HvB	Wermut	syst	TEE	1-109	an den Zähnen leidet
Artemisia absinthium L.	Asteraceae	mon	HvB	Wermut	top	TEE	1-109	an den Zähnen leidet
Artemisia absinthium L.	Asteraceae	mon	MF	Wermuthkraut	syst	ANT	3	Gifte von Pilzen vertreibt es, dem todbringenden Schierling, tritt auch den Bissen von giftigen Tieren entgegen

Artemisia absinthium L.	Asteraceae	mon	MF	Wermuthkraut	top	DER	3	bringt sie frischen Wunden Nutzen, fressende Geschwüre auf dem Kopf, Juckreiz, Gechwulst unter der Zunge
Artemisia absinthium L.	Asteraceae	mon	MF	Wermuthkraut	top	DER	3	hält unmässige Schwellung des Gemächts in Schranken
Artemisia absinthium L.	Asteraceae	mon	MF	Wermuthkraut	vol	EAR	3	macht die verstopften Ohren frei, vertreibt jedes Ohrenklingen (mit Rindsgalle vermischt)
Artemisia absinthium L.	Asteraceae	mon	MF	Wermuthkraut	top	EYE	3	Augen werden klar, blau geschlagene Augen, fliesst Eiter aus den Augen, Geisring (evtl. Grauer Star)
Artemisia absinthium L.	Asteraceae	mon	MF	Wermuthkraut	syst	GAS	3	kräftigt den Magen, treibt Spulwürmer aus, erweicht den Leib, stillt Bauchgrimmen
Artemisia absinthium L.	Asteraceae	mon	MF	Wermuthkraut	syst	GAS	3	jeden bösen, unverdaunten Saft und Krankheitsstoff im Magen reinigend austreiben, Seekrankheit
Artemisia absinthium L.	Asteraceae	mon	MF	Wermuthkraut	syst	GAS	3	wirst Du schädliche Abscheu vor Speise beheben
Artemisia absinthium L.	Asteraceae	mon	MF	Wermuthkraut	syst	GAS	3	Gelbsüchtige
Artemisia absinthium L.	Asteraceae	mon	MF	Wermuthkraut	syst	GYN	3	führt Monatsfluss herbei
Artemisia absinthium L.	Asteraceae	mon	MF	Wermuthkraut	syst	GYN	3	führt den ordentlichen Monatsfluss herbei
Artemisia absinthium L.	Asteraceae	mon	MF	Wermuthkraut	syst	NER	3	bringt es Schlaf übers Haupt (nur darf der Kranke nicht bemerken, dass man's ihm unterschiebt).
Artemisia absinthium L.	Asteraceae	mon	MF	Wermuthkraut	syst	OTH	3	dying hair black
Artemisia absinthium L.	Asteraceae	mon	MF	Wermuthkraut	syst	OTH	3	muss man sich vor Schnacken nicht mehr fürchten, hält Motten fern
Artemisia absinthium L.	Asteraceae	mon	MF	Wermuthkraut	syst	OTH	3	Tintenpulver mit Wein, wenn Du damir ein Pergament beschreibst, wird es keine Maus je benagen
Artemisia absinthium L.	Asteraceae	mon	MF	Wermuthkraut	syst	OTH	3	hilft es der Milz
Artemisia absinthium L.	Asteraceae	mon	MF	Wermuthkraut	syst	RES	3	geschwollener Hals
Artemisia absinthium L.	Asteraceae	mon	MF	Wermuthkraut	syst	RES	3	hilft der Brust
Artemisia absinthium L.	Asteraceae	mon	MF	Wermuthkraut	syst	TON	3	ihm [dem Sieger] so einen Preis zu geben, . . ., mit dem er nämlich feste Gesundheit sich bewahren kann
Artemisia absinthium L.	Asteraceae	mon	MF	Wermuthkraut	syst	URO	3	befördert den Harnabgang
Artemisia absinthium L.	Asteraceae	ren	LF	Wermut	syst	ANT	I / I	wider das gifft des wutzerling (Schierling), Spinnen und anderen giftigen thier
Artemisia absinthium L.	Asteraceae	ren	LF	Wermut	syst	ANT	I / I	gut wider gifftigen Schwämme
Artemisia absinthium L.	Asteraceae	ren	LF	Wermut	top	DER	I / I	ist er nützlich den dunklen Augen und vertreibt darin das ungerunnen Blut
Artemisia absinthium L.	Asteraceae	ren	LF	Wermut	vol	EAR	I / I	lindert weetagen der oren
Artemisia absinthium L.	Asteraceae	ren	LF	Wermut	top	EYE	I / I	benimmt den schmerzen der augen
Artemisia absinthium L.	Asteraceae	ren	LF	Wermut	syst	GAS	I / I	nimmt hinweg geelsucht
Artemisia absinthium L.	Asteraceae	ren	LF	Wermut	syst	GAS	I / I	vertreibt den Wind und Bläste im Bauch, auch weetagen des mgensm der gleichen unwillen
Artemisia absinthium L.	Asteraceae	ren	LF	Wermut	top	GAS	I / I	ist dem Magen sehr dienstlich und nützlich
Artemisia absinthium L.	Asteraceae	ren	LF	Wermut	top	GAS	I / I	töted die Würm im Leib
Artemisia absinthium L.	Asteraceae	ren	LF	Wermut	top	GYN	I / I	bringt der den frawen ihr krankheyt
Artemisia absinthium L.	Asteraceae	ren	LF	Wermut	top	OTH	I / I	Milzsüchtigen
Artemisia absinthium L.	Asteraceae	ren	LF	Wermut	vol	TEE	I / I	lindert das Zanwee
Artemisia absinthium L.	Asteraceae	ren	LF	Wermut	top	URO	I / I	Wassersüchtigen
Artemisia absinthium L.	Asteraceae	s pop	AV	Wemut	syst	APH	p. 59	Sexuelle Überreiztheit
Artemisia absinthium L.	Asteraceae	s pop	AV	Wemut	syst	GAS	p. 319, 532	Würmer
Artemisia absinthium L.	Asteraceae	s pop	AV	Wemut	top	GAS	p. 532	Würmer
Artemisia absinthium L.	Asteraceae	s pop	BVA	Wermut	syst	GAS	p. 262	Appetitlosigkeit, Magenträgheit, Bauchkoliken, Verdauungsstörungen, Magenkrämpfen, Blähungen, Übersäuerung des Magens, Völle- und Druckgefühl im Magen, Sodbrennen, gelb belegter Zunge, Mundgeruch, Magenwinden, Leber- und Galleleiden, gegen Würmer
Artemisia absinthium L.	Asteraceae	s pop	BVA	Wermut	syst	GAS	p. 262	Appetitlosigkeit, Magenträgheit, Bauchkoliken, Verdauungsstörungen, Magenkrämpfen, Blähungen, Übersäuerung des Magens, Völle- und Druckgefühl im Magen, Sodbrennen, gelb belegter Zunge, Mundgeruch, Magenwinden, Leber- und Galleleiden, gegen Würmer
Artemisia absinthium L.	Asteraceae	s pop	BVA	Wermut	syst	GAS	p. 262	Appetitlosigkeit, Magenträgheit, Bauchkoliken, Verdauungsstörungen, Magenkrämpfen, Blähungen, Übersäuerung des Magens, Völle- und Druckgefühl im Magen, Sodbrennen, gelb belegter Zunge, Mundgeruch, Magenwinden, Leber- und Galleleiden, gegen Würmer
Artemisia absinthium L.	Asteraceae	s pop	JK	Wermut	top	DER	p. 442	Hautkrankheiten, Biss am Leib, Insekten- und Mückenstiche
Artemisia absinthium L.	Asteraceae	s pop	JK	Wermut	syst	GAS	p. 442	Magenleiden
Artemisia absinthium L.	Asteraceae	s pop	JK	Wermut	syst	GAS	p. 442	Magenleiden
Artemisia absinthium L.	Asteraceae	s pop	JK	Wermut	syst	GAS	p. 442	Magenleiden
Artemisia absinthium L.	Asteraceae	s pop	JK	Wermut	syst	GAS	p. 442	hartnäckiger Würmer
Artemisia absinthium L.	Asteraceae	s pop	JK	Wermut	vol	NER	p. 442	Schlaflosigkeit und Schwäche der Kopfnerven
Artemisia absinthium L.	Asteraceae	s pop	JK	Wermut	syst	URO	p. 442	Nierenleiden
Artemisia absinthium L.	Asteraceae	s pop	JK	Wermut	syst	URO	p. 442	Nierenleiden
Artemisia absinthium L.	Asteraceae	s pop	JK	Wermut	syst	URO	p. 442	Nierenleiden
Artemisia absinthium L.	Asteraceae	s pop	JK	Wermut	syst	URO	p. 442	Nierenleiden
Artemisia absinthium L.	Asteraceae	s pop	KN	Wermut	syst	CAR	p. 491	kreislaufverbessernd
Artemisia absinthium L.	Asteraceae	s pop	KN	Wermut	top	EYE	p. 491	Augenwasser
Artemisia absinthium L.	Asteraceae	s pop	KN	Wermut	syst	GAS	p. 491	Magenmittel, übelen Geruch aus dem Munde, Leber leidet, Gelbsucht, Galle
Artemisia absinthium L.	Asteraceae	s pop	KN	Wermut	syst	GAS	p. 491	Magenmittel, übelen Geruch aus dem Munde, Leber leidet, Gelbsucht, Galle
Artemisia absinthium L.	Asteraceae	s pop	KN	Wermut	syst	GAS	p. 491	Reisende, Magenbeschwerden und Übelkeiten geplagt
Artemisia absinthium L.	Asteraceae	s pop	KN	Wermut	syst	GAS	p. 491	wurmtreibende Wirkung (Spulwürmer)
Artemisia absinthium L.	Asteraceae	s pop	KN	Wermut	syst	GYN	p. 491	fördert die Periode, erleichtert den Geburtsvorgang
Artemisia absinthium L.	Asteraceae	s pop	KN	Wermut	syst	OTH	p. 491	Stoffwelchselstörungen: Fettsucht, Zuckerkrankheit
Artemisia absinthium L.	Asteraceae	s pop	KN	Wermut	syst	SKE	p. 491	Stoffwelchselstödungen: Rheumatismus
Artemisia absinthium L.	Asteraceae	s pop	KN	Wermut	syst	URO	p. 491	Katarrhaen der Harnwege
Artemisia absinthium L.	Asteraceae	s pop	KN	Wermut	syst	URO	p. 491	Katarrhaen der Harnwege
Artemisia absinthium L.	Asteraceae	s pop	KN	Wermut	syst	URO	p. 491	Katarrhaen der Harnwege
Artemisia absinthium L.	Asteraceae	s pop	UB	Wermut	syst	GAS	p.158 /186	Amara aromatica, Magenerkrankungen, Gallestörungen
Artemisia absinthium L.	Asteraceae	s pop	UB	Wermut	syst	GAS	p. 459	Ausleitung und Regneration, Heilpflanzen zur Aktivierung der Ausscheidung
Artemisia absinthium L.	Asteraceae	s pop	UB	Wermut	syst	HUM	p. 459	Ausleitung und Regneration, Heilpflanzen zur Aktivierung der Ausscheidung
Artemisia absinthium L.	Asteraceae	s pop	UB	Wermut	syst	HUM	p. 459	Ausleitung und Regneration, Heilpflanzen zur Aktivierung der Ausscheidung
Artemisia absinthium L.	Asteraceae	sci	EMA	Wermut	syst	GAS	/234463/2008 corr.	temporary loss of appetite / mild dyspeptic / gastrointestinal disorders
Artemisia absinthium L.	Asteraceae	sci	EMA	Wermut	syst	GAS	/234463/2008 corr.	temporary loss of appetite / mild dyspeptic / gastrointestinal disorders
Artemisia absinthium L.	Asteraceae	sci	EMA	Wermut	syst	GAS	/234463/2008 corr.	temporary loss of appetite / mild dyspeptic / gastrointestinal disorders
Artemisia absinthium L.	Asteraceae	sci	EMA	Wermut	syst	GAS	/234463/2008 corr.	temporary loss of appetite / mild dyspeptic / gastrointestinal disorders
Arum maculatum agg.	Araceae	ant	DIOS	Aronstab	syst	OTH	158	Blätter werden als Speise eingesalzen, auch getrocknet für sich allein gekocht und gegessen
Arum maculatum agg.	Araceae	ant	DIOS	Aronstab	syst	OTH	158	Überhaupt ist sie [die Wurzel] wegen der geringeren Schärfe essbar
Arum maculatum agg.	Araceae	ant	DIOS	Aronstab	top	SKE	158	Wurzel mit Rindermist als Umschlag hat gute Wirkung bei Podagra
Arum maculatum agg.	Araceae	mon	HvB	Aronstab	syst	DER	1-49	wenn in irgendeinem Menschen seine Fäulins zu einer schwarzen Pustel wird, in welcher der Tod des Mensche ist, der "seltega" genannt wird, . . .
Arum maculatum agg.	Araceae	mon	HvB	Aronstab	syst	DER	1-49	wenn in irgendeinem Menschen seine Fäulins zu einer schwarzen Pustel wird, in welcher der Tod des Mensche ist, der "seltega" genannt wird, . . .
Arum maculatum agg.	Araceae	mon	HvB	Aronstab	syst	FEV	1-49	ein Mensch, der ein schleimiges Fieber im Magen hat, aus dem verschiedenartige Schüttelfröste erwachsen

Arum maculatum agg.	Araceae	mon	HvB	Aronstab	syst	NER	1-49	ein Mensch, in dem die Melancholie wächst, der hat ein finsteres Gemüt und ist immer traurig
Arum maculatum agg.	Araceae	mon	HvB	Aronstab	syst	SKE	1-49	wenn ein Mensch so unter Gicht leidet, dass alle Glieder versagend fallen, und dass seine Zunge beim Sprechen versagt, . .
Arum maculatum agg.	Araceae	mon	HvB	Aronstab	syst	SKE	1-49	wenn ein Mensch so unter Gicht leidet, dass alle Glieder versagend fallen, und dass seine Zunge beim Sprechen versagt, . .
Arum maculatum agg.	Araceae	ren	LF	Aronstab	top	DER	XXXIX / XXII	heilen die alten boesen schaeden unnd fisteln
Arum maculatum agg.	Araceae	ren	LF	Aronstab	top	DER	XXXIX / XXII	in oel gesotten und warm darauff gesessen heylet die feig blattern
Arum maculatum agg.	Araceae	ren	LF	Aronstab	n.d.	DER	XXXIX / XXII	gut zu den geschwulsten insonderheyt der oren
Arum maculatum agg.	Araceae	ren	LF	Aronstab	top	DER	XXXIX / XXII	macht das angesicht sauber
Arum maculatum agg.	Araceae	ren	LF	Aronstab	top	DER	XXXIX / XXII	so reinget sie alle unseüberkeit der alten wunden und fürderts zur heylung / sie vertreibt auch das faule fleisch so in den wunden wechst / beide bletter un wurzel auf die pestilentz blasen gelegt benemen das gifft der selbigen und heylen sie
Arum maculatum agg.	Araceae	ren	LF	Aronstab	n.d.	OTH	XXXIX / XXII	die auffwerffende kaes in die Aron bletter wickelt . . .so bleibt der kaes gut und wahrhafftig
Arum maculatum agg.	Araceae	s pop	JK	Aronstab	top	DER	p. 326	unreiner Haut und bei fressenden Wunden, vertreibt die Knollen am Leibe, das den austretenden After zurückdrängt
Arum maculatum agg.	Araceae	s pop	JK	Aronstab	syst	FEV	p. 326	wenn ansteckende Krankheiten umgehen, wie Grippe oder Influenza
Arum maculatum agg.	Araceae	s pop	JK	Aronstab	syst	GAS	p. 326	reinigt den Magen
Arum maculatum agg.	Araceae	s pop	JK	Aronstab	syst	GAS	p. 326	Magenverschleimung
Arum maculatum agg.	Araceae	s pop	JK	Aronstab	syst	RES	p. 326	reinigt die Lungen
Arum maculatum agg.	Araceae	s pop	JK	Aronstab	syst	RES	p. 326	reinigt die Lunge von zähem Schleim
Arum maculatum agg.	Araceae	s pop	JK	Aronstab	syst	RES	p. 326	verhockten Lungenkatarrh
Asarum europaeum agg.	Aristolochiace	ant	DIOS	Haselwurz	syst	GYN	71	die monatliche Reinigung befördernd
Asarum europaeum agg.	Aristolochiace	ant	DIOS	Haselwurz	syst	HUM	71	brechenerregend
Asarum europaeum agg.	Aristolochiace	ant	DIOS	Haselwurz	syst	SKE	71	Ischias
Asarum europaeum agg.	Aristolochiace	ant	DIOS	Haselwurz	syst	URO	71	Wassersüchtige
Asarum europaeum agg.	Aristolochiace	ant	DIOS	Haselwurz	syst	URO	71	harntreibend
Asarum europaeum agg.	Aristolochiace	mon	HvB	Haselwurz	syst	OTH	1-48	. . .würde sie ihm grösseren Schmerz verursachen
Asarum europaeum agg.	Aristolochiace	mon	MF	Haselwurz	syst	GAS	46	heilt den Leberschmerz
Asarum europaeum agg.	Aristolochiace	mon	MF	Haselwurz	syst	GAS	46	soll Gelbsucht vertreiben
Asarum europaeum agg.	Aristolochiace	mon	MF	Haselwurz	syst	GYN	46	sorgt für geordneten Monatsfluss
Asarum europaeum agg.	Aristolochiace	mon	MF	Haselwurz	syst	GYN	46	hilft auch bei Leiden der weiblichen Scham
Asarum europaeum agg.	Aristolochiace	mon	MF	Haselwurz	syst	HUM	46	reinigt durch Ausleerung nach oben Herz und Brust (Purgieren)
Asarum europaeum agg.	Aristolochiace	mon	MF	Haselwurz	syst	SKE	46	schlägt Hüftgicht in die Flucht
Asarum europaeum agg.	Aristolochiace	mon	MF	Haselwurz	syst	URO	46	hilft Wassersüchtigen
Asarum europaeum agg.	Aristolochiace	mon	MF	Haselwurz	syst	URO	46	treibt den Harn
Asarum europaeum agg.	Aristolochiace	ren	LF	Haselwurz	syst	ANT	V / III	denen von gifftigen thieren gebissen
Asarum europaeum agg.	Aristolochiace	ren	LF	Haselwurz	syst	CAR	V / III	dient auch wohl den Wassersüchtigen und den weetagen der Hüft
Asarum europaeum agg.	Aristolochiace	ren	LF	Haselwurz	top	DER	V / III	rootlauff
Asarum europaeum agg.	Aristolochiace	ren	LF	Haselwurz	top	EYE	V / III	hitzigen augen ; stercket das hinr und gedechnuss
Asarum europaeum agg.	Aristolochiace	ren	LF	Haselwurz	syst	GYN	V / III	bringt den frawen ihr krankheyt
Asarum europaeum agg.	Aristolochiace	ren	LF	Haselwurz	syst	HUM		treibt aus durch den Stuhlgang
Asarum europaeum agg.	Aristolochiace	ren	LF	Haselwurz	top	NER	V / III	weetagen des haupts
Asarum europaeum agg.	Aristolochiace	ren	LF	Haselwurz	syst	RES	V / III	vertreibt auch das keichen von husten
Asarum europaeum agg.	Aristolochiace	ren	LF	Haselwurz	syst	URO	V / III	treibt den Harn
Asarum europaeum agg.	Aristolochiace	S pop	VAL	Haselwurz	syst	RES	p. 152	Bronchite, Asthma
Asarum europaeum agg.	Aristolochiace	S pop	VAL	Haselwurz	syst	RES	p. 152	Bronchite, Asthma
Avena sativa agg.	Poaceae	ant	DIOS	Hafer	top	DER	137	Kataplasmen [unspezifisch]
Avena sativa agg.	Poaceae	ant	DIOS	Hafer	syst	GAS	137	Durchfall stellt
Avena sativa agg.	Poaceae	ant	DIOS	Hafer	syst	RES	137	gut gegen Husten
Avena sativa agg.	Poaceae	mon	HvB	Hafer	vol	NER	1-3	wer an Gicht leidet und davon einen gespaltenen Geist und nichtige Gedanken hat
Avena sativa agg.	Poaceae	mon	HvB	Hafer	syst	OTH	1-3	, die etwas und mäsig kränkeln, ist er gut zu essen
Avena sativa agg.	Poaceae	ren	LF	Hafer	top	DER	CIII / LXVII	vertreibt die masen, zerteilet geschwulst und geschwaer
Avena sativa agg.	Poaceae	ren	LF	Hafer	syst	GAS	CIII / LXVII	stopft den stulgang
Avena sativa agg.	Poaceae	ren	LF	Hafer	syst	GYN	CIII / LXVII	Hafer gleich wie Gerste: mehret den Weibern die Milch
Avena sativa agg.	Poaceae	s pop	AV	Hafer	syst	CAR	p. 217	nährendes Herzmittel
Avena sativa agg.	Poaceae	s pop	AV	Hafer	top	DER	p. 472	bringt mangelnde Hauttätigkeit wieder in Ordnung
Avena sativa agg.	Poaceae	s pop	AV	Hafer	syst	EYE	p. 124	Augenleiden
Avena sativa agg.	Poaceae	s pop	AV	Hafer	syst	GAS	295, 308, 470, 472	Sodbrennen, Durchfall, Kleinkindnahrung, verdorbener Magen, Magen- Darmschleimhautentzündungen
Avena sativa agg.	Poaceae	s pop	AV	Hafer	syst	GAS	p. 89	Kleinkindnahrung
Avena sativa agg.	Poaceae	s pop	AV	Hafer	syst	GYN	p. 140	Weissfluss
Avena sativa agg.	Poaceae	s pop	AV	Hafer	syst	NER	p. 471	Nerven regeneriert und neue Nervenkraft verleiht
Avena sativa agg.	Poaceae	s pop	AV	Hafer	other	NER	p. 107, 271	Gehirn und Nervengrippe, Schlaflosigkeit
Avena sativa agg.	Poaceae	s pop	AV	Hafer	syst	OTH	p. 176	Lymphgranulomatose Hodgkinsche Krankheit
Avena sativa agg.	Poaceae	s pop	AV	Hafer	syst	RES	p. 471	Katarrhe, Husten, fieberhafte Zustände
Avena sativa agg.	Poaceae	s pop	KN	Hafer	syst	GAS	p. 467	Getränk nahrhaft, leicht verdaulich, kühlend bei vorhandenen inneren Hitzen
Avena sativa agg.	Poaceae	s pop	KN	Hafer	syst	SKE	p. 468	Mischungen . . . die auf gichtisch-rheumatische Krankheitszustände güsntig wirken
Avena sativa agg.	Poaceae	s pop	KN	Hafer	syst	TON	p. 467	für die Rekonvaleszenten, . . ., die doch vor allem neues, gesundes Blut brauchen
Avena sativa agg.	Poaceae	s pop	KN	Hafer	top	URO	p. 411	Nieren-, Blasen und Steinleiden
Avena sativa agg.	Poaceae	s pop	UB	Hafer	syst	CAR	p. 400	Arteriosklerose Vorbeugung
Avena sativa agg.	Poaceae	s pop	UB	Hafer	syst	NER	p. 420	Schlafstörungen, Nervosität und Unruhe
Avena sativa agg.	Poaceae	s pop	WI	Hafer	top	GAS	p. 93	Volksmedizin: Lebererkankungen
Avena sativa agg.	Poaceae	s pop	WI	Hafer	syst	NER	p. 93	Volksmedizin: nervöser Erschöpfung, Schlaflosigkeit und sog. Nervenschwäche
Avena sativa agg.	Poaceae	s pop	WI	Hafer	syst	NER	p. 93	Volksmedizin: nervöser Erschöpfung, Schlaflosigkeit und sog. Nervenschwäche
Avena sativa agg.	Poaceae	s pop	WI	Hafer	top	NER	p. 93	Volksmedizin: Sedativum bei Hypertonikern
Avena sativa agg.	Poaceae	s pop	WI	Hafer	syst	SKE	p. 93	Volksmedizin: Kneipp - Adjuvans bei Rheuma und Gicht
Avena sativa agg.	Poaceae	s pop	WI	Hafer	top	SKE	p. 93	Volksmedizin: Kneipp - Adjuvans bei Rheuma und Gicht
Avena sativa agg.	Poaceae	s pop	WI	Hafer	syst	TON	p. 93	Volksmedizin: Roborantium in der Rekonvaleszenz
Avena sativa agg.	Poaceae	sci	EMA	Hafer	top	DER	HMPC/368600/2007	minor inflammations of the skin (such als sunburn) and as an aid in healing of minor wounds
Avena sativa agg.	Poaceae	sci	EMA	Hafer	top	DER	HMPC/368600/2007	minor inflammations of the skin (such als sunburn) and as an aid in healing of minor wounds
Avena sativa agg.	Poaceae	sci	EMA	Hafer	top	DER	HMPC/368600/2007	minor inflammations of the skin (such als sunburn) and as an aid in healing of minor wounds
Beta vulgaris L.	Chenopodiace	ant	DIOS	Rote Bete	top	DER	145	Mit den rohen Blättern muss man die weißen Flecken, nachdem sie vorher mit Natron behandelt sind, bestreichen, ebenso die kahlen Stellen, nachdem sie zerkratzt sind und die fressenden Geschwüre. Gekocht heilen sie Hautausschlag, Feuerbrandwunden und roseartige Entzündungen
Beta vulgaris L.	Chenopodiace	ant	DIOS	Rote Bete	top	DER	145	vertreibt Schorf [und Nisse]
Beta vulgaris L.	Chenopodiace	ant	DIOS	Rote Bete	vol	DER	145	Frostbeulen
Beta vulgaris L.	Chenopodiace	ant	DIOS	Rote Bete	top	EAR	145	hilft auch bei Ohrenschmerzen.
Beta vulgaris L.	Chenopodiace	ant	DIOS	Rote Bete	syst	GAS	216	bei Dysenterie und Magenleiden zu helfen
Beta vulgaris L.	Chenopodiace	ant	DIOS	Rote Bete	syst	GYN	216	den rothen Fluss der Frauen zu stellen
Beta vulgaris L.	Chenopodiace	ant	DIOS	Rote Bete	top	HUM	145	reinigt ihr Saft den Kopf, wenn er mit Honig in die Nase injiziert wird
Beta vulgaris L.	Chenopodiace	ant	DIOS	Rote Bete	top	OTH	145	vertreibt [Schorf] und Nisse
Beta vulgaris L.	Chenopodiace	mon	LO	Rüeben	n.d.	OTH	n.d.	n.d.
Beta vulgaris L.	Chenopodiace	ren	LF	Rüeben	syst	ANT	CXX / LXXVII	gut wider allerley gifft
Beta vulgaris L.	Chenopodiace	ren	LF	Rüeben	syst	APH	CXX / LXXVII	bringt lust zur unkeüscheyt
Beta vulgaris L.	Chenopodiace	ren	LF	Rüeben	top	DER	CXX / LXXVII	seubern sie das angesict und den ganzen leib
Beta vulgaris L.	Chenopodiace	ren	LF	Rüeben	syst	EYE	CXX / LXXVII	lautere Augen
Beta vulgaris L.	Chenopodiace	ren	LF	Rüeben	syst	GAS	CXX / LXXVII	bringen lust zu essen
Beta vulgaris L.	Chenopodiace	ren	LF	Rüeben	top	SKE	CXX / LXXVII	kalte podagra

Beta vulgaris L.	Chenopodiace	ren	LF	Rüeben	syst	URO	CXX / LXXVII	treiben den Harn
Beta vulgaris L.	Chenopodiace	s pop	AV	Randen	syst	DER	p. 234	Kropf
Beta vulgaris L.	Chenopodiace	s pop	AV	Randen	syst	FEV	p. 108	Grippe, Fieber
Beta vulgaris L.	Chenopodiace	s pop	AV	Randen	syst	GAS	p. 306, 318	Blinddarmentzündung, Dysbakterie, Störungen der Leber und Gallewege
Beta vulgaris L.	Chenopodiace	s pop	AV	Randen	syst	OTH	p. 176	Lymphgranulomatose Hodgkinsche Krankheit
Beta vulgaris L.	Chenopodiace	s pop	AV	Randen	syst	OTH	p. 291	Drüsentätigkeit, Übergewichtsvermeidung
Beta vulgaris L.	Chenopodiace	s pop	AV	Randen	syst	RES	p. 156	Erkrankung der Atmungsorgane
Beta vulgaris L.	Chenopodiace	s pop	JK	Rote Bete	syst	TON	p. 404	blutarme, skrofulöse und sonst schwächliche Kinder
Cannabis sativa L.	Cannabaceae	ant	DIOS	Hanf	syst	APH	209	reichlich genossen, die Zeugung vernichtet
Cannabis sativa L.	Cannabaceae	ant	DIOS	Hanf	top	EAR	209	Grün zu Saft verarbeitet und eingeträufelt ist sie ein gutes Mittel gegen Ohrenleiden
Cannabis sativa L.	Cannabaceae	mon	HvB	Hanf	syst	GAS	1-11	und er ist für gesunde Menchen heilsam zu essen, und in ihrem Magen ist er leicht und nützlich, si dass er den Schleim einigermassen aus dem Magen wegschafft, . . .
Cannabis sativa L.	Cannabaceae	mon	HvB	Hanf	top	GAS	1-11	wer einen kalten Magen hat
Cannabis sativa L.	Cannabaceae	ren	LF	Hanf	syst	APH	CCXX / CXLVIII	trücknet und tilget aus den menschllichen Samen
Cannabis sativa L.	Cannabaceae	ren	LF	Hanf	top	DER	CCXX / CXLVIII	lindert und zerteylet die geschwulst
Cannabis sativa L.	Cannabaceae	ren	LF	Hanf	top	DER	CCXX / CXLVIII	gut zu dem brandt
Cannabis sativa L.	Cannabaceae	ren	LF	Hanf	top	EAR	CCXX / CXLVIII	legt den schmerz derselibgen [ohren]
Cannabis sativa L.	Cannabaceae	ren	LF	Hanf	syst	GAS	CCXX / CXLVIII	vertreibt die blaest und wind im leib
Cannabis sativa L.	Cannabaceae	ren	LF	Hanf	top	SKE	CCXX / CXLVIII	gut zu dem podagra
Cannabis sativa L.	Cannabaceae	ren	LF	Hanf	top	SKE	CCXX / CXLVIII	erweycht die zusamen gezogne gleych
Cannabis sativa L.	Cannabaceae	s pop	UB	Hanf	syst	NER	p. 427	depressiver Verstimmung
Capsella bursa-pastoris agg.	Brassicaceae	ant	DIOS	Hirtentäschchen	syst	GYN	154	befördert die Katamenien und tötet die
Capsella bursa-pastoris agg.	Brassicaceae	ant	DIOS	Hirtentäschchen	syst	HUM	154	Der Same ist scharf, erwärmend er führt die Galle nach oben und unten ab; Genossen führt er auch das Blut ab und öffnet die innerlichen Abszesse
Capsella bursa-pastoris agg.	Brassicaceae	ant	DIOS	Hirtentäschchen	top	SKE	154	Ischiasschmerz
Capsella bursa-pastoris agg.	Brassicaceae	mon	LO	Hirtentäschel	n.d.	OTH	n.d.	n.d.
Capsella bursa-pastoris agg.	Brassicaceae	ren	LF	Hirtentäschel	top	DER	CCCXVII / CCXXXIII	kület die hitzige geschwulst, rotlauff
Capsella bursa-pastoris agg.	Brassicaceae	ren	LF	Hirtentäschel	top	DER	CCCXVII / CCXXXIII	frische Wunden, Nasenbluten
Capsella bursa-pastoris agg.	Brassicaceae	ren	LF	Hirtentäschel	syst	GAS	CCCXVII / CCXXXIII	rote Ruhr, Blutspeien
Capsella bursa-pastoris agg.	Brassicaceae	ren	LF	Hirtentäschel	top	GAS	CCCXVII / CCXXXIII	Magen entzündet ist
Capsella bursa-pastoris agg.	Brassicaceae	ren	LF	Hirtentäschel	syst	GYN	CCCXVII / CCXXXIII	den Weibern so zuviel fliessen
Capsella bursa-pastoris agg.	Brassicaceae	s pop	AV	Hirtentäschel	syst	CAR	p. 214	Hämorrhodialblutung
Capsella bursa-pastoris agg.	Brassicaceae	s pop	AV	Hirtentäschel	syst	GYN	p. 240	Periodenstörung
Capsella bursa-pastoris agg.	Brassicaceae	s pop	BVA	Hirtentäschchen	syst	DER	p. 92	Nasenbluten, blutende Hämorrhoiden
Capsella bursa-pastoris agg.	Brassicaceae	s pop	BVA	Hirtentäschchen	syst	DER	p. 92	Nasenbluten, blutende Hämorrhioiden
Capsella bursa-pastoris agg.	Brassicaceae	s pop	BVA	Hirtentäschchen	top	DER	p. 92	Blutungen bei Verletzugnen stillen
Capsella bursa-pastoris agg.	Brassicaceae	s pop	BVA	Hirtentäschchen	syst	GAS	p. 92	erschlaffte Darmperistaltik
Capsella bursa-pastoris agg.	Brassicaceae	s pop	BVA	Hirtentäschchen	syst	GAS	p. 92	erschlaffte Darmperistaltik
Capsella bursa-pastoris agg.	Brassicaceae	s pop	BVA	Hirtentäschchen	syst	GYN	p. 92	starke Menstruationsblutung, Wechseljahrbeschwerden, Gebärmutterblutung
Capsella bursa-pastoris agg.	Brassicaceae	s pop	BVA	Hirtentäschchen	syst	GYN	p. 92	starke Menstruationsblutung, Wechseljahrbeschwerden, Gebärmutterblutung
Capsella bursa-pastoris agg.	Brassicaceae	s pop	BVA	Hirtentäschchen	syst	TEE	p. 92	Zahnblutungen
Capsella bursa-pastoris agg.	Brassicaceae	s pop	BVA	Hirtentäschchen	syst	TEE	p. 92	Zahnblutungen
Capsella bursa-pastoris agg.	Brassicaceae	s pop	JK	Hirtentäschel	top	DER	p. 373	Verletzungen wo Blut fliesst
Capsella bursa-pastoris agg.	Brassicaceae	s pop	JK	Hirtentäschel	syst	GAS	p. 373	Blutspeien, Durchfall und inneren Brand
Capsella bursa-pastoris agg.	Brassicaceae	s pop	JK	Hirtentäschel	syst	GYN	p. 373	Wechseljahre, srarke Blutungen; jungen Mädchen um den Geschlechtstrieb zu dämpfen
Capsella bursa-pastoris agg.	Brassicaceae	s pop	JK	Hirtentäschel	syst	URO	p. 373	Blut im Urin
Capsella bursa-pastoris agg.	Brassicaceae	s pop	MT	Hirtentäschel	syst	CAR	p. 21	zu hohem, zu niederem Blutdruck
Capsella bursa-pastoris agg.	Brassicaceae	s pop	MT	Hirtentäschel	syst	DER	p. 21	blutenden Hämorrhoiden
Capsella bursa-pastoris agg.	Brassicaceae	s pop	MT	Hirtentäschel	syst	GYN	p. 21	starken Menstruationsblutungen,Pubertätszeit, Wechseljahre, geschwollene Brüsten während des Stillens, Gebärmutterblutungen
Capsella bursa-pastoris agg.	Brassicaceae	s pop	MT	Hirtentäschel	top	SKE	p. 21	äusseren Muskelerkrankungen, Leistenbruch
Capsella bursa-pastoris agg.	Brassicaceae	s pop	UB	Hirtentäschel	top	DER	p. 303	Wundbehandlung
Capsella bursa-pastoris agg.	Brassicaceae	s pop	UB	Hirtentäschel	top	DER	p. 303	Wundbehandlung
Capsella bursa-pastoris agg.	Brassicaceae	s pop	WI	Hirtentäschelkraut	syst	GYN	p. 114	Volksmedizin: blutstillendes Mittel, übermässige Monatsblutung
Capsella bursa-pastoris agg.	Brassicaceae	sci	EMA	Hirtentäschel	syst	GYN	/HPMC/26766/2010	reduction of heavy menstrual bleeding in women with regular cycles
Capsella bursa-pastoris agg.	Brassicaceae	sci	WI	Hirtentäschelkraut	syst	OTH	p. 114	hämostyptische Wirkung
Carum carvi L.	Apiaceae	ant	DIOS	Kümmel	syst	ANT	184	wird auch vorteilhaft den Gegenmitteln
Carum carvi L.	Apiaceae	ant	DIOS	Kümmel	syst	GAS	184	gut für den Magen und angenehm für den Mund, er befördert die Verdauung
Carum carvi L.	Apiaceae	ant	DIOS	Kümmel	syst	OTH	184	Die gekochte Wurzel wird gegessen wie die Möhre
Carum carvi L.	Apiaceae	ant	DIOS	Kümmel	syst	URO	184	harntreibend
Carum carvi L.	Apiaceae	mon	HvB	Kümmel	syst	GAS	1-17	Mensch, der gekochter und gebratener Käse essen will
Carum carvi L.	Apiaceae	ren	LF	Wiesenkümmel	syst	GAS	CCXXII / CL	ist dem Magen und dem Mund ganz dienstlich, vertreibt die Bläst und wind
Carum carvi L.	Apiaceae	ren	LF	Wiesenkümmel	syst	GAS	CCXXII / CL	dem Magen
Carum carvi L.	Apiaceae	ren	LF	Wiesenkümmel	syst	URO	CCXXII / CL	treibt den Harn
Carum carvi L.	Apiaceae	ren	LF	Wiesenkümmel	syst	URO	CCXXII / CL	treibt den Harn
Carum carvi L.	Apiaceae	s pop	BVA	Kümmel	syst	GAS	p. 128	Blähungen, träger Magentätigkeit, Appetitlosigkeit, Darmkrämfen, Verdauungsförderung, Luftschlucken, Unterleibsbeschwerden der Kleinkinder
Carum carvi L.	Apiaceae	s pop	BVA	Kümmel	syst	GAS	p. 128	Blähungen, träger Magentätigkeit, Appetitlosigkeit, Darmkrämfen, Verdauungsförderung, Luftschlucken, Unterleibsbeschwerden der Kleinkinder
Carum carvi L.	Apiaceae	s pop	BVA	Kümmel	syst	GYN	p. 128	Milchbildung der Wöchnerinnen, Gebärmutterkrämpfen
Carum carvi L.	Apiaceae	s pop	BVA	Kümmel	syst	GYN	p. 128	Milchbildung der Wöchnerinnen, Gebärmutterkrämpfen
Carum carvi L.	Apiaceae	s pop	JK	Kümmel	syst	CAR	p. 385	und gegen Schwindelanfälle, die nicht durch Verstopfung hervorgerufen werden
Carum carvi L.	Apiaceae	s pop	JK	Kümmel	syst	GAS	p. 385	Bauchschmerzen
Carum carvi L.	Apiaceae	s pop	JK	Kümmel	syst	GAS	p. 385	verhinder Blähungen und Leibschmerzen
Carum carvi L.	Apiaceae	s pop	JK	Kümmel	syst	GAS	p. 385	stillt das Abweichen und vertreibt Würmer
Carum carvi L.	Apiaceae	s pop	JK	Kümmel	syst	RES	p. 385	Hustengetränk für Kinder
Carum carvi L.	Apiaceae	s pop	JK	Kümmel	syst	URO	p. 385	beim Urinieren Beschwerden oder an Hämorrhoiden leiden
Carum carvi L.	Apiaceae	s pop	KN	Kümmel	syst	GYN	p. 476	Begünstigt Beginn und Verlauf der Periode, wirkt bei der Geburt wehenanregend und erleichtert den Geburtsvorgang
Carum carvi L.	Apiaceae	s pop	KN	Kümmel	syst	URO	p. 476	Wasserausscheidung der Nieren begünstigt
Carum carvi L.	Apiaceae	s pop	UB	Kümmel	syst	GAS	p. 115	blähungstreibend
Carum carvi L.	Apiaceae	s pop	UB	Kümmel	top	GAS	p. 115	blähungstreibend
Carum carvi L.	Apiaceae	s pop	WI	Kümmel	top	DER	p. 135	Volksmedizin: Mundwässer
Carum carvi L.	Apiaceae	s pop	WI	Kümmel	top	HUM	p. 135	Volksmedizin: hautreizende Einreibungen
Carum carvi L.	Apiaceae	sci	ESCOP	Carvi fructus	syst	GAS	I p. 64	spasmodic gastro-intestinal complaints, flatulence, bloating
Carum carvi L.	Apiaceae	sci	WI	Kümmel	syst	GAS	p. 135	Verdauungsbeschwerden
Carum carvi L.	Apiaceae	sci	WI	Kümmel	syst	GAS	p. 135	Verdauungsbeschwerden
Chelidonium majus L.	Papaveraceae	ant	DIOS	Schöllkraut	top	DER	163	und mit Wein als Umschlag Bläschenausschlag
Chelidonium majus L.	Papaveraceae	ant	DIOS	Schöllkraut	top	EYE	163	Der mit Honig gemischte und in einem ehernen Geschirr über Kohlen gekochte Saft dient zur Schärfe des Gesichtes
Chelidonium majus L.	Papaveraceae	ant	DIOS	Schöllkraut	top	GAS	163	Die Wurzel mit Anis und Weißwein getrunken heilt die Gelbsucht
Chelidonium majus L.	Papaveraceae	mon	HvB	Schöllkraut	top	DER	1-138	Unreines isst oder trinkt oder berührt, wovon er geschwürig im Körper wird
Chelidonium majus L.	Papaveraceae	mon	MF	Schöllkraut	top	DER	52	Male auf der Haut beseitigen
Chelidonium majus L.	Papaveraceae	mon	MF	Schöllkraut	top	EYE	52	trübe Augen hellsichtig zu machen
Chelidonium majus L.	Papaveraceae	mon	MF	Schöllkraut	syst	GAS	52	gegen Gelbsucht
Chelidonium majus L.	Papaveraceae	mon	MF	Schöllkraut	top	TEE	52	hält jeden Zahnschmerz fern
Chelidonium majus L.	Papaveraceae	ren	LF	Schöllkraut	top	DER	CCXCVI / CCCXXXIII	in die wunden von schaeden gethon
Chelidonium majus L.	Papaveraceae	ren	LF	Schöllkraut	top	DER	CCXCVI / CCCXXXIII	heylet fisteln, krebs und den wolff damit geschaeschen

Chelidonium majus L.	Papaveraceae	ren	LF	Schöllkraut	top	EYE	CCXCVI / CCCXXXIII	macht klar augen, darin getropfft
Chelidonium majus L.	Papaveraceae	ren	LF	Schöllkraut	syst	GAS	CCXCVI / CCCXXXIII	eröffnet die verstopfung der leber und vertreibt die gelsucht
Chelidonium majus L.	Papaveraceae	s pop	JK	Schöllkraut	top	CAR	p. 418	zieht Blutandrang vom Kopf hinunter (Ch. In den Schuhen)
Chelidonium majus L.	Papaveraceae	s pop	JK	Schöllkraut	syst	DER	p. 418	Hautunreinigkeiten
Chelidonium majus L.	Papaveraceae	s pop	JK	Schöllkraut	top	DER	p. 418	Warzen
Chelidonium majus L.	Papaveraceae	s pop	JK	Schöllkraut	top	DER	p. 418	nehmen dem Kropf das Wachstum, bringen verunstaltende Barthaare zum Verschwinden
Chelidonium majus L.	Papaveraceae	s pop	JK	Schöllkraut	syst	EYE	p. 418	Augenschwäche
Chelidonium majus L.	Papaveraceae	s pop	JK	Schöllkraut	top	EYE	p. 418	Augenleiden, die aus Verstopfung hervorgehen
Chelidonium majus L.	Papaveraceae	s pop	JK	Schöllkraut	syst	GAS	p. 418	Gelbsucht
Chelidonium majus L.	Papaveraceae	s pop	JK	Schöllkraut	top	NER	p. 418	Zittern in den Gliedern
Chelidonium majus L.	Papaveraceae	s pop	JK	Schöllkraut	top	SKE	p. 418	Knoten von Gicht und Rheumatismus
Chelidonium majus L.	Papaveraceae	s pop	JK	Schöllkraut	top	TEE	p. 418	Zahn
Chelidonium majus L.	Papaveraceae	s pop	JK	Schöllkraut	syst	URO	p. 418	Wassersucht
Chelidonium majus L.	Papaveraceae	s pop	UB	Schöllkraut	syst	GAS	p. 185	Gallestörungen
Chelidonium majus L.	Papaveraceae	s pop	UB	Schöllkraut	syst	GAS	p. 185	Gallestörungen
Chelidonium majus L.	Papaveraceae	s pop	UB	Schöllkraut	syst	GAS	p. 459	Ausleitung und Regneration, Heilpflanzen zur Aktivierung der Ausscheidung
Chelidonium majus L.	Papaveraceae	s pop	WI	Schöllkraut	top	DER	p. 149	Volksmedizin: Warzen
Cichorium intybus L.	Asteraceae	ant	DIOS	Wegwarte	top	ANT	148	als Umschlag leisten gegen Skorpionsbiss Hülfe
Cichorium intybus L.	Asteraceae	ant	DIOS	Wegwarte	top	CAR	148	mit Grütze [für sich allein als Umschlag] sind sie von guter Wirkung bei Herzleiden
Cichorium intybus L.	Asteraceae	ant	DIOS	Wegwarte	top	CAR	148	für sich allein als Umschlag sind sie von guter Wirkung bei Herzleiden
Cichorium intybus L.	Asteraceae	ant	DIOS	Wegwarte	top	DER	148	heilen mit Graupen zusammen die Rose; mit Bleiweiß und Essig dient ihr Saft als Salbe in Fällen, welche Abkühlung erheischen
Cichorium intybus L.	Asteraceae	ant	DIOS	Wegwarte	top	EYE	148	Augenentzündungen
Cichorium intybus L.	Asteraceae	ant	DIOS	Wegwarte	syst	GAS	148	gut für den Magen. Gekocht stellen sie den Durchfall, wenn sie mit Essig genommen werden
Cichorium intybus L.	Asteraceae	ant	DIOS	Wegwarte	top	SKE	148	helfen bei Podagra
Cichorium intybus L.	Asteraceae	mon	HvB	Wegwarte	syst	GAS	1-60	keine rechte Verdauung
Cichorium intybus L.	Asteraceae	mon	HvB	Wegwarte	syst	RES	1-60	Brust und Stimme
Cichorium intybus L.	Asteraceae	ren	LF	Wegwarte	top	DER	CCLXXXVII / CCLXIII	rotlauffen
Cichorium intybus L.	Asteraceae	ren	LF	Wegwarte	top	EYE	CCLXXXVII / CCLXIII	hitzingen Geschwulsten der Augen
Cichorium intybus L.	Asteraceae	ren	LF	Wegwarte	syst	GAS	CCLXXXVII / CCLXIII	stellen sie den stuhlgang
Cichorium intybus L.	Asteraceae	ren	LF	Wegwarte	syst	GAS	CCLXXXVII / CCLXIII	lebersüchtigen, gelbsucht
Cichorium intybus L.	Asteraceae	ren	LF	Wegwarte	top	GAS	CCLXXXVII / CCLXIII	weetagen im Magenschlund
Cichorium intybus L.	Asteraceae	ren	LF	Wegwarte	syst	GYN	CCLXXXVII / CCLXIII	bringen den frawen ihr Zeyt
Cichorium intybus L.	Asteraceae	ren	LF	Wegwarte	syst	NER	CCLXXXVII / CCLXIII	machen ziemlich schlaffen
Cichorium intybus L.	Asteraceae	ren	LF	Wegwarte	top	NER	CCLXXXVII / CCLXIII	mildert den Schmerzen des Hauptes
Cichorium intybus L.	Asteraceae	ren	LF	Wegwarte	top	SKE	CCLXXXVII / CCLXIII	hitzigen podagra
Cichorium intybus L.	Asteraceae	ren	LF	Wegwarte	syst	URO	CCLXXXVII / CCLXIII	blasen
Cichorium intybus L.	Asteraceae	s pop	JK	Wegwarte	top	DER	p. 437	äussere Leiden des Unterleibs
Cichorium intybus L.	Asteraceae	s pop	JK	Wegwarte	syst	FEV	p. 437	ist sehr gut bei Fieber
Cichorium intybus L.	Asteraceae	s pop	JK	Wegwarte	syst	GAS	p. 437	gegen Würmer
Cichorium intybus L.	Asteraceae	s pop	JK	Wegwarte	syst	GAS	p. 437	reinigen Magen, Leber; vertreiben überflüssige Galle, heilt Gelbsucht und stillt das Blutspeien
Cichorium intybus L.	Asteraceae	s pop	JK	Wegwarte	syst	URO	p. 437	Nierensteine
Cichorium intybus L.	Asteraceae	s pop	JK	Wegwarte	syst	URO	p. 437	reinigen Nieren, treibt den Urin
Cichorium intybus L.	Asteraceae	s pop	KN	Wegwarte	top	DER	p. 491	schmerzlichen Entzündungen am Körper
Cichorium intybus L.	Asteraceae	s pop	KN	Wegwarte	syst	GAS	p. 491	appetitanregend, drüsenanregend und leicht stuhlfördernd
Cichorium intybus L.	Asteraceae	s pop	KN	Wegwarte	syst	GAS	p. 491	Stauungen in Pfortader, Leber, Milz und Hämorrhoiden werden günstig beeinflusst
Cichorium intybus L.	Asteraceae	s pop	KN	Wegwarte	top	GAS	p. 491	Magendrücken
Cichorium intybus L.	Asteraceae	s pop	UB	Wegwarte	syst	GAS	p. 154 / 186	Amara tonica / simplex / pura, Magenerkrankungen, Gallestörungen
Cichorium intybus L.	Asteraceae	s pop	UB	Wegwarte	syst	GAS	p. 154 / 186	Amara tonica / simplex / pura, Magenerkrankungen, Gallestörungen
Cichorium intybus L.	Asteraceae	s pop	UB	Wegwarte	syst	GAS	p. 154 / 186	Amara tonica / simplex / pura, Magenerkrankungen, Gallestörungen
Cichorium intybus L.	Asteraceae	s pop	UB	Wegwarte	syst	HUM	p. 461	Ausleitung und Regeneration; Heilpflanzen mit Gerbstoffen
Cichorium intybus L.	Asteraceae	sci	EMA	Wegwarte	syst	GAS	IMPC/12181672010	mild digestive disorders and temporary loss of appetite
Clematis vitalba L.	Ranunculacea	ant	DIOS	Clematis	top	DER	260	Blätter als Umschlag vertreiben Aussatz
Clematis vitalba L.	Ranunculacea	ant	DIOS	Clematis	syst	HUM	260	führt Schleim und Galle nach unten ab
Clematis vitalba L.	Ranunculacea	mon	LO	Waldrebe	n.d.	OTH	n.d.	n.d.
Clematis vitalba L.	Ranunculacea	ren	LF	Waldrebe	syst	GYN	LII / XXIII	bringen den frawen ihr bloedigkeit
Clematis vitalba L.	Ranunculacea	ren	LF	Waldrebe	syst	n.d.	LII / XXIII	gleiche Würkung wie Stickwurz?el
Clematis vitalba L.	Ranunculacea	ren	LF	Waldrebe	syst	NER	LII / XXIII	so den fallenden Siechtagen haben, den Schwindel und in den Gliedern erlahmen
Clematis vitalba L.	Ranunculacea	ren	LF	Waldrebe	syst	OTH	LII / XXIII	machen auch das Milz klein
Clematis vitalba L.	Ranunculacea	ren	LF	Waldrebe	syst	URO	LII / XXIII	treiben den Harn
Clematis vitalba L.	Ranunculacea	s pop	VAL	Waldrebe	top	SKE	p. 229	douleurs rhumatismales, névralgies, névrites, cellulalgies
Colchicum autumnale agg.	Liliaceae	ant	DIOS	Herbstzeitlose	syst	OTH	235	Genossen tödtet sie durch Erstickung ähnlich wie die Pilze
Colchicum autumnale agg.	Liliaceae	mon	HvB	Herbstzeitlose	syst	OTH	1-46	Die Herbstzeitlose ist kalt und trocken, und in ihr ist weder Heil noch Gesundheit, und sie taugt keinem Menschen zum Essen, denn wenn er sie ässe . . .
Colchicum autumnale agg.	Liliaceae	ren	LF	Herbstzeitlose	top	OTH	CII / CXXXIIII	allerley leuss damit zu vertreiben
Colchicum autumnale agg.	Liliaceae	ren	LF	Herbstzeitlose	syst	OTH	CII / CXXXIIII	wurzel von Zeitlosen gegessen toeted; einer aber unwissend gegessen kue milch zu trinken geben.
Colchicum autumnale agg.	Liliaceae	s pop	UB	Herbstzeitlose	syst	SKE	p. 285	Gicht
Conium maculatum L.	Apiaceae	ant	DIOS	Schierling	top	DER	233	kriechende Geschwüre und Rose beseitigt er als Salbe
Conium maculatum L.	Apiaceae	ant	DIOS	Schierling	top	EYE	233	ausgepresste Saft mit Wein gemischt vortheilhaft den schmerzlindernden Kollyrien zutresetzt
Conium maculatum L.	Apiaceae	ant	DIOS	Schierling	top	GYN	233	Sie vertreiben ferner die Milch und verhindern ein Grösserwerden der jungfräulichen Brüste
Conium maculatum L.	Apiaceae	ant	DIOS	Schierling	syst	OTH	233	Auch dieses gehört zu den vernichtenden Giften, indem es in Folge von Unterkühlung tödtet.
Conium maculatum L.	Apiaceae	ant	DIOS	Schierling	top	URO	233	Das Kraut und die Dolde, fein gestossen als Umschlag um die Hoden gelegt, helfen gegen Pollutionen, auch lassen sie als Kataplasma die Genitalien erschlaffen; lassen auch die Hoden der Knaben verkümmern
Conium maculatum L.	Apiaceae	mon	HvB	Schierling	top	SKE	1-39	der mit Knütteln und Stangen arg zerbläut ist, oder wer aus irgendeiner Höhe gefallen ist, so dass sein Fleisch und die Glieder hart mitgenommen sind . . .
Conium maculatum L.	Apiaceae	ren	LF	Schierling	top	DER	CCXXVIII / CLIIII	legung und linderung der Schmerzen zu külen alle hitzigen gebresten als ist der rotlauf
Conium maculatum L.	Apiaceae	ren	LF	Schierling	top	GYN	CCXXVIII / CLIIII	das er den seugenden Weibern die Milch vertreibe
Conium maculatum L.	Apiaceae	s pop	VAL	Schierling	top	NER	p. 227	calmant, analgesique, cataplasme résloutif et sédatif
Conium maculatum L.	Apiaceae	s pop	VAL	Schierling	syst	NER	p. 227	raideurs muscularies (Parkinson), spasmes
Conium maculatum L.	Apiaceae	s pop	VAL	Schierling	syst	NER	p. 227	raideurs muscularies (Parkinson), spasmes
Convallaria spp.	Asparagaceae	ant	DIOS	Weisswurz	top	DER	p. 215	gutes Mittel für Wunden ist. Sie entfernt auch Flecken im Gesicht
Convallaria spp.	Asparagaceae	ant	DIOS	Ephemeron	top	DER	p. 235	vertheilen Oedeme und Geschwülste, welche noch keine Flüssigkeit enthalten
Convallaria spp.	Asparagaceae	ant	DIOS	Ephemeron	top	TEE	p. 235	Mundspülwasser ein Mittel gegen Zahnschmerzen
Convallaria spp.	Asparagaceae	mon	HvB	Maiglöckchen	syst	DER	1-159	Skrofeln, Ausschläge, irgendein Geschwür, in dem Gift ist
Convallaria spp.	Asparagaceae	mon	HvB	Maiglöckchen	syst	NER	1-159	Epilepsie
Convallaria spp.	Asparagaceae	ren	LF	Maiglöckchen	syst	CAR	CXXXIII / LXXXVIII	ist krefftig zu sterken das herz, [hirn und leber].
Convallaria spp.	Asparagaceae	ren	LF	Maiglöckchen	syst	CAR	CXXXIII / LXXXVIII	ist krefftig zu sterken das herz, hirn und leber.
Convallaria spp.	Asparagaceae	ren	LF	Maiglöckchen	top	DER	CXXXIII / LXXXVIII	zerteylen die geschwulsts darin sich das eyter noch nicht gesammelt hat
Convallaria spp.	Asparagaceae	ren	LF	Maiglöckchen	syst	DER	CXXXIII / LXXXVIII	dem aussatz were
Convallaria spp.	Asparagaceae	ren	LF	Maiglöckchen	top	EYE	CXXXIII / LXXXVIII	fürtrefflich zu den Augen, dann er mach sie lauter und vertreibt die dunkelheyt derselbigen

Convallaria spp.	Asparagaceae	ren	LF	Maiglöckchen	syst	NER	CXXXIII / LXXXVIII	ohnmacht, schwindel und fallende sucht
Convallaria spp.	Asparagaceae	ren	LF	Maiglöckchen	top	TEE	CXXXIII / LXXXVIII	Wurzel gesotten und das wasser im mund gehalten vertreibt das zanwee
Convallaria spp.	Asparagaceae	s pop	UB	Maiglöckchen	syst	CAR	p. 383	Herzglycoside 2. Ordnung
Coriandrum sativum L.	Apiaceae	ant	DIOS	Korander	syst	APH	185	befördert die Samenbildung
Coriandrum sativum L.	Apiaceae	ant	DIOS	Korander	top	DER	185	gutes Mittel gegen brennende Entzündungen auf der Haut
Coriandrum sativum L.	Apiaceae	ant	DIOS	Korander	top	DER	185	roseartige Entzündungen und kriechende Geschwüre. Mit Honig und Rosinen hilft es bei Epinyktiden, Hodenentzündungen und Karbunkeln; mit Schrot von Hülsenfrüchten zerteilt es Drüsen und Geschwülste
Coriandrum sativum L.	Apiaceae	ant	DIOS	Korander	syst	GAS	185	treibt den Bandwurm aus
Coriandrum sativum L.	Apiaceae	ant	DIOS	Korander	top	OTH	185	Zu viel genommen greift es den Verstand gefährlich an;
Coriandrum sativum L.	Apiaceae	mon	LO	Koriander	n.d.	OTH	n.d.	n.d.
Coriandrum sativum L.	Apiaceae	ren	LF	Koriander	top	DER	CXCV / CXXX	kuelet und leschet die hitzigen gebresten
Coriandrum sativum L.	Apiaceae	ren	LF	Koriander	top	DER	CXCV / CXXX	verzert der die kroepff und andere geschwulst
Coriandrum sativum L.	Apiaceae	ren	LF	Koriander	top	DER	CXCV / CXXX	zerteyllt und verzert er die hitzige geschwulst
Coriandrum sativum L.	Apiaceae	ren	LF	Koriander	syst	GAS	CXCV / CXXX	stelt den bauchfluss / er beschleusst auch den magen unnd behellt die speiss darinn also lang bis sie gedewet und gekocht werden
Coriandrum sativum L.	Apiaceae	ren	LF	Koriander	syst	GAS	CXCV / CXXX	toedtet die würm und mehret den samen
Coriandrum sativum L.	Apiaceae	ren	LF	Koriander	syst	GYN	CXCV / CXXX	ein fraw die ire zeit zuvil hab . .. So gestee der fluss ein tag
Coriandrum sativum L.	Apiaceae	ren	LF	Koriander	n.d.	OTH	CXCV / CXXX	behelt dasselbige [Fleysch] den ganzen summer unversert
Coriandrum sativum L.	Apiaceae	s pop	UB	Koriander	syst	GAS	p. 183	Gallenstörungen
Coriandrum sativum L.	Apiaceae	s pop	WI	Koriander	syst	GAS	p. 166	Volksmedizin: gegen Würmer
Coriandrum sativum L.	Apiaceae	s pop	WI	Koriander	top	SKE	p. 166	Volksmedizin: Einreibemittel gegen Rheuma und Gelenkschmerzen
Coriandrum sativum L.	Apiaceae	sci	WI	Koriander	syst	GAS	p. 166	pflanzliches Magen-Darm-Mittel
Coriandrum sativum L.	Apiaceae	sci	WI	Koriander	syst	GAS	p. 166	pflanzliches Magen-Darm-Mittel
Corylus avellana L.	Betulaceae	ant	DIOS	Haselnuss	top	DER	112	durch die Fuchskrankheit ausfallende Haar
Corylus avellana L.	Betulaceae	ant	DIOS	Haselnuss	top	EYE	112	die Pupillen der blauäugigen Kinder schwarz färben, wenn der Vorderkopf damit eingerieben wird.
Corylus avellana L.	Betulaceae	ant	DIOS	Haselnuss	syst	RES	112	veralteten Husten
Corylus avellana L.	Betulaceae	ant	DIOS	Haselnuss	syst	RES	112	lindern sie den Katarrh
Corylus avellana L.	Betulaceae	mon	HvB	Haselnuss	top	DER	3-11	wo Skrofeln am Menschen sind . . .
Corylus avellana L.	Betulaceae	mon	HvB	Haselnuss	syst	URO	3-11	Ein Mann, dessen Samen von dünnflüssiger Beschaffenheit ist, so dass erkeine Kinder zeugt
Corylus avellana L.	Betulaceae	ren	LF	Haselnuss	syst	ANT	XIII - CCXXIIII / CLI	kommen sie zuhilffdenen so vonn einem scorpion gestochen seind
Corylus avellana L.	Betulaceae	ren	LF	Haselnuss	syst	APO	XIII - CCXXIIII / CLI	dem mag kein gifftig thier denselben tag schaden
Corylus avellana L.	Betulaceae	ren	LF	Haselnuss	top	DER	XIII - CCXXIIII / CLI	macht das har widerumb wachsen
Corylus avellana L.	Betulaceae	ren	LF	Haselnuss	top	EYE	XIII - CCXXIIII / CLI	an die das vorder teyl des haupts salb das sie den kindern die grawen augen schwartz machen
Corylus avellana L.	Betulaceae	ren	LF	Haselnuss	syst	RES	XIII - CCXXIIII / CLI	dienstlich dem langwirigen husten
Corylus avellana L.	Betulaceae	ren	LF	Haselnuss	syst	RES	XIII - CCXXIIII / CLI	verzerehns die hauptflüss
Corylus avellana L.	Betulaceae	s pop	JK	Haselnuss	syst	FEV	p. 368	schweisstreibend, fieberbekämpfend
Corylus avellana L.	Betulaceae	s pop	JK	Haselnuss	syst	RES	p. 368	hartnäckigen Husten
Crocus sativus L.	Iridaceae	ant	DIOS	Safran	top	DER	76	gegen Rose; Mundsalbe (mit Milch)
Crocus sativus L.	Iridaceae	ant	DIOS	Safran	top	DER	85	eitermachend und die Wunden reinigend
Crocus sativus L.	Iridaceae	ant	DIOS	Safran	top	EAR	76	Ohrenflüsse
Crocus sativus L.	Iridaceae	ant	DIOS	Safran	n.d.	EYE	77	Verdunkelungen auf der Pupille reinigende
Crocus sativus L.	Iridaceae	ant	DIOS	Safran	top	EYE	76	gegen Augenflüsse
Crocus sativus L.	Iridaceae	ant	DIOS	Safran	top	EYE	85	es wirkt gegen beginnenden Star,
Crocus sativus L.	Iridaceae	ant	DIOS	Safran	n.d.	GAS	77	erweichende, die Verdauung befördernde und erwärmende Kraft
Crocus sativus L.	Iridaceae	ant	DIOS	Safran	top	GYN	85	Verhärtungen und Verstopfungen in der Gebärmutter und gegen die bösartigen Geschwüre
Crocus sativus L.	Iridaceae	ant	DIOS	Safran	top	NER	85	schlafmachende Kraft, Phrenitis leidende
Crocus sativus L.	Iridaceae	ant	DIOS	Safran	n.d.	OTH	76	gegen den Rausch
Crocus sativus L.	Iridaceae	ant	DIOS	Safran	n.d.	URO	77	hanrtreibende [Kraft]
Crocus sativus L.	Iridaceae	ant	DIOS	Safran	syst	URO	76	treibt den Urin
Crocus sativus L.	Iridaceae	mon	LO	Safran	n.d.	OTH	n.d.	n.d.
Crocus sativus L.	Iridaceae	ren	LF	Safran	syst	APH	CCXLVIII / CLXVII	reizt zu der unkeuschheyt
Crocus sativus L.	Iridaceae	ren	LF	Safran	top	DER	CCXLVIII / CLXVII	lindert die hitz insonderheit das rotlauff
Crocus sativus L.	Iridaceae	ren	LF	Safran	top	EYE	CCXLVIII / CLXVII	ist er gut zu den trieffenden Augen
Crocus sativus L.	Iridaceae	ren	LF	Safran	syst	GAS	CCXLVIII / CLXVII	bekomt wohl dem blöden verkehrten magen
Crocus sativus L.	Iridaceae	ren	LF	Safran	syst	OTH	CCXLVIII / CLXVII	mit süssem Wein getrunken verhütet er die Trunkenheit
Crocus sativus L.	Iridaceae	ren	LF	Safran	syst	RES	CCXLVIII / CLXVII	auch nützlich der Brust, ist nützlich denen so husten
Crocus sativus L.	Iridaceae	ren	LF	Safran	top	SKE	CCXLVIII / CLXVII	weetagen der fuess
Crocus sativus L.	Iridaceae	ren	LF	Safran	syst	URO	CCXLVIII / CLXVII	treibt den harn
Crocus sativus L.	Iridaceae	s pop	WI	Safran	syst	NER	p. 176	Volksmedizin: Sedativum, Spasmolytikum
Crocus sativus L.	Iridaceae	s pop	WI	Safran	syst	NER	p. 176	Volksmedizin: Stomachikum
Cucurbita pepo L.	Cucurbitaceae	ant	DIOS	Kürbis	top	DER	149	besänftigt er Ödeme und Eiterbeulen
Cucurbita pepo L.	Cucurbitaceae	ant	DIOS	Kürbis	top	EYE	149	gegen Augenentzündungen
Cucurbita pepo L.	Cucurbitaceae	ant	DIOS	Kürbis	syst	GAS	149	löst den Bauch gelinde; so erweicht er den Bauch leicht
Cucurbita pepo L.	Cucurbitaceae	ant	DIOS	Kürbis	top	NER	149	Kindern, welche am Sonnenstich leiden
Cucurbita pepo L.	Cucurbitaceae	ant	DIOS	Kürbis	top	SKE	149	Podagra
Cucurbita pepo L.	Cucurbitaceae	ant	DIOS	Kürbis	top	SKE	149	mit Rosenöl eingetröpfelt gegen Ohrenschmerzen
Cucurbita pepo L.	Cucurbitaceae	ant	DIOS	Kürbis	top	SKE	149	auch bei dem durch Hitze angegriffenen Gesicht
Cucurbita pepo L.	Cucurbitaceae	mon	HvB	Kürbis	syst	n.d.	1-87	Und sie sind für Kranke und Gesunde gut zu essen
Cucurbita pepo L.	Cucurbitaceae	ren	LF	Kürbis	syst	APH	CCCXCVIII / CCLVII	so lescht sie den menschlichen Samen und machen unlust zu den ehelichen wercken
Cucurbita pepo L.	Cucurbitaceae	ren	LF	Kürbis	vol	CAR	CCCXCVIII / CCLVII	in onmacht - kommt er wiederum zu sich
Cucurbita pepo L.	Cucurbitaceae	ren	LF	Kürbis	top	DER	CCCXCVIII / CCLVII	angestrichen macht ein schön angesicht, vertreiben sie allerley masen, flecken, rosmucken
Cucurbita pepo L.	Cucurbitaceae	ren	LF	Kürbis	top	DER	CCCXCVIII / CCLVII	biss der hunde
Cucurbita pepo L.	Cucurbitaceae	ren	LF	Kürbis	top	DER	CCCXCVIII / CCLVII	heylt sie die fliessende schwaer
Cucurbita pepo L.	Cucurbitaceae	ren	LF	Kürbis	top	EYE	CCCXCVIII / CCLVII	über die stirn legt, leschen sidie hitz der augen
Cucurbita pepo L.	Cucurbitaceae	ren	LF	Kürbis	syst	GAS	CCCXCVIII / CCLVII	lindern den bauch und sind gut dem hitzigen Magen
Cucurbita pepo L.	Cucurbitaceae	ren	LF	Kürbis	syst	GAS	CCCXCVIII / CCLVII	macht speien
Cucurbita pepo L.	Cucurbitaceae	ren	LF	Kürbis	syst	GAS	CCCXCVIII / CCLVII	macht speien
Cucurbita pepo L.	Cucurbitaceae	ren	LF	Kürbis	syst	URO	CCCXCVIII / CCLVII	sind auch der blasen nützlich
Cucurbita pepo L.	Cucurbitaceae	ren	LF	Kürbis	syst	URO	CCCXCVIII / CCLVII	treibt den Harn
Cucurbita pepo L.	Cucurbitaceae	ren	LF	Kürbis	syst	URO	CCCXCVIII / CCLVII	treibt den Lendenstein
Cucurbita pepo L.	Cucurbitaceae	s pop	JK	Kürbis	top	DER	p. 385	Wundsalbe für brennende Füsse, entzündete Geschwüre, Krampfadern, Greisenbrand
Cucurbita pepo L.	Cucurbitaceae	s pop	JK	Kürbis	syst	GAS	p. 385	seit alters her wirksames Wurmittel, sogar zur Abtreibung des Bandwurms
Cucurbita pepo L.	Cucurbitaceae	s pop	JK	Kürbis	syst	URO	p. 385	Blutreinigungsmittel, Nierenentzündung und Zuckerkrankheit
Cucurbita pepo L.	Cucurbitaceae	s pop	UB	Kürbis	syst	URO	p. 262	benigner Prostatahyperplasie
Cucurbita pepo L.	Cucurbitaceae	s pop	UB	Kürbis	syst	URO	p. 262	benigner Prostatahyperplasie
Cucurbita pepo L.	Cucurbitaceae	sci	EMA	Kürbis	syst	URO	HMPC/136024/2010	for the relief of lower urinary tract symptoms related to benign prostatic hyperplasia . . .
Cucurbita pepo L.	Cucurbitaceae	sci	EMA	Kürbis	syst	URO	HMPC/136024/2010	for the relief of lower urinary tract symptoms related to benign prostatic hyperplasia . . .
Cucurbita pepo L.	Cucurbitaceae	sci	EMA	Kürbis	syst	URO	HMPC/136024/2010	for the relief of lower urinary tract symptoms related to benign prostatic hyperplasia . . .
Cucurbita pepo L.	Cucurbitaceae	sci	ESCOP	Cucurbitae semen	syst	URO	II p. 50	symptomatic treatment of micturition disorders (such as dysuria, pollakisuria, urine retention ans hesitancy) in benign prostatic hyperplasia, irritable bladder
Cucurbita pepo L.	Cucurbitaceae	sci	WI	Kürbis	syst	URO	p. 179	dysurische Beschwerden (benignes Prostata Adenom)
Cydonia oblonga Mill.	Rosaceae	ant	DIOS	Quitte	syst	ANT	82	getrunken gegen Kanthariden, Buprestis und die giftigen Fichtenraupen
Cydonia oblonga Mill.	Rosaceae	ant	DIOS	Quitte	top	DER	82	wirkt gegen krätzige Geschwüre, Kleingrind, Frostbeulen, kriechende Geschwüre
Cydonia oblonga Mill.	Rosaceae	ant	DIOS	Quitte	top	DER	82	Mastdarm [und Gebärmutter]vorfall
Cydonia oblonga Mill.	Rosaceae	ant	DIOS	Quitte	top	DER	82	Condylomen
Cydonia oblonga Mill.	Rosaceae	ant	DIOS	Quitte	top	EYE	82	bei Augenentzündungen

Cydonia oblonga Mill.	Rosaceae	ant	DIOS	Quitte	syst	GAS	108	die an Magen, Dysenterie, Blutspeien und Cholera leiden; Aufguss davon ist denen als Trank zu empfehlen, die Magen- und Bauchfluss
Cydonia oblonga Mill.	Rosaceae	ant	DIOS	Quitte	top	GAS	82	Kataplasmen gemischt zum Stopfen des Durchfalls, gegen krampfhafte Bewegung und Hitze des Magens; Leberverhärtung
Cydonia oblonga Mill.	Rosaceae	ant	DIOS	Quitte	syst	GYN	82	Gegen Blutsturz, Bauchfluss und übermäßige Menstruation
Cydonia oblonga Mill.	Rosaceae	ant	DIOS	Quitte	top	GYN	82	gegen Verwundungen in der Gebärmutter
Cydonia oblonga Mill.	Rosaceae	ant	DIOS	Quitte	top	GYN	82	[Mastdarm und Gebärmutter]vorfall
Cydonia oblonga Mill.	Rosaceae	ant	DIOS	Quitte	top	GYN	82	bei schwärenden Brüsten
Cydonia oblonga Mill.	Rosaceae	ant	DIOS	Quitte	top	OTH	82	hält den Schweiß zurück
Cydonia oblonga Mill.	Rosaceae	ant	DIOS	Quitte	top	OTH	82	Orthopnöe leiden
Cydonia oblonga Mill.	Rosaceae	ant	DIOS	Quitte	syst	URO	82	sind gleichfalls harntreibend
Cydonia oblonga Mill.	Rosaceae	ant	DIOS	Quitte	top	URO	82	stellt als Injektion das zu häufige Harnen
Cydonia oblonga Mill.	Rosaceae	mon	HvB	Quitte	top	DER	3-4	Aber wo es an einem Menschen Geschwüre oder Übelriechendes hat . . .
Cydonia oblonga Mill.	Rosaceae	mon	HvB	Quitte	syst	GAS	3-4	Und wer viel Speichel auswirft, esse oft diese Fruct, so dass der Speichel vermindert wird
Cydonia oblonga Mill.	Rosaceae	mon	HvB	Quitte	syst	SKE	3-4	denn wer gichtkrank ist, esse oft diese Frucht gekocht und gebraten
Cydonia oblonga Mill.	Rosaceae	ren	LF	Quitte	top	DER	CCXI / CXL	heylet die carbunkel, bringt auch das Haar wieder denen es ausfellt
Cydonia oblonga Mill.	Rosaceae	ren	LF	Quitte	syst	EYE	CCXI / CXL	hitzigen Augen
Cydonia oblonga Mill.	Rosaceae	ren	LF	Quitte	syst	GAS	CCXI / CXL	Bauchfluss haben
Cydonia oblonga Mill.	Rosaceae	ren	LF	Quitte	syst	GAS	CCXI / CXL	denen so blut speyen
Cydonia oblonga Mill.	Rosaceae	ren	LF	Quitte	syst	GAS	CCXI / CXL	sind dem Magen dienstlich, bauchfluss, rote rhur und blut speien
Cydonia oblonga Mill.	Rosaceae	ren	LF	Quitte	top	GAS	CCXI / CXL	bauchlauffen haen, dem überwerffen des magens
Cydonia oblonga Mill.	Rosaceae	ren	LF	Quitte	syst	GYN	CCXI / CXL	frawen, die zuviel fliessen
Cydonia oblonga Mill.	Rosaceae	ren	LF	Quitte	top	GYN	CCXI / CXL	treiben die muter so aus dem leib
Cydonia oblonga Mill.	Rosaceae	ren	LF	Quitte	syst	URO	CCXI / CXL	treiben den harn
Cydonia oblonga Mill.	Rosaceae	s pop	FLU	Quitte	top	DER	p. 41	Entzündungen der Mund- und Rachenhöhle, aufgesprungene Haut
Cydonia oblonga Mill.	Rosaceae	s pop	FLU	Quitte	syst	GAS	p. 41	mildes Abführmittel
Daucus carota L.	Apiaceae	ant	DIOS	Wilde Möhre	syst	ANT	183	sowie gegen die Bisse und Stiche giftiger Tiere
Daucus carota L.	Apiaceae	ant	DIOS	Wilde Möhre	syst	APH	183	Ferner befördert er die Empfängnis
Daucus carota L.	Apiaceae	ant	DIOS	Wilde Möhre	syst	APH	183	reizt sowohl zum Beischlaf
Daucus carota L.	Apiaceae	ant	DIOS	Wilde Möhre	top	DER	183	reinigen krebsige Geschwüre
Daucus carota L.	Apiaceae	ant	DIOS	Wilde Möhre	syst	GYN	183	getrunken oder im Zäpfchen eingelegt befördert die Menstruation
Daucus carota L.	Apiaceae	ant	DIOS	Wilde Möhre	top	GYN	183	als auch wirft sie, im Zäpfchen eingelegt, den Embryo hinaus
Daucus carota L.	Apiaceae	ant	DIOS	Wilde Möhre	syst	OTH	183	ist besser zum Essen
Daucus carota L.	Apiaceae	ant	DIOS	Wilde Möhre	syst	OTH	183	Brustfellentzündung
Daucus carota L.	Apiaceae	ant	DIOS	Wilde Möhre	syst	URO	183	Wassersucht,
Daucus carota L.	Apiaceae	ant	DIOS	Wilde Möhre	syst	URO	183	gegen Harnverhaltung
Daucus carota L.	Apiaceae	ant	DIOS	Wilde Möhre	syst	URO	183	selbst harntreibend
Daucus carota L.	Apiaceae	mon	LO	Karotte	syst	OTH	n.d.	n.d.
Daucus carota L.	Apiaceae	ren	LF	Karotte	syst	ANT	- CCCXCI / CCLXIII	denen so von gifftigen thieren gebissen seind
Daucus carota L.	Apiaceae	ren	LF	Karotte	syst	APH	- CCCXCI / CCLXIII	reyzt zu den eelichen wercken
Daucus carota L.	Apiaceae	ren	LF	Karotte	syst	APH	- CCCXCI / CCLXIII	bringen lust zu eelichen wercken
Daucus carota L.	Apiaceae	ren	LF	Karotte	syst	APO	- CCCXCI / CCLXIII	ist er nachmals vor gedachten [gifftigen] thiern sicher
Daucus carota L.	Apiaceae	ren	LF	Karotte	top	DER	- CCCXCI / CCLXIII	reynigen und heylen die umb sich fressende geschwaer
Daucus carota L.	Apiaceae	ren	LF	Karotte	syst	GAS	- CCCXCI / CCLXIII	ist nützlich zu den gebresten der leber
Daucus carota L.	Apiaceae	ren	LF	Karotte	syst	GYN	- CCCXCI / CCLXIII	bringt den frawen ire zeit / er fürdert zu der geburt
Daucus carota L.	Apiaceae	ren	LF	Karotte	syst	GYN	- CCCXCI / CCLXIII	ist gut den frawen so die muter überscih steigt unnd legt den schmerzen der muter
Daucus carota L.	Apiaceae	ren	LF	Karotte	top	GYN	- CCCXCI / CCLXIII	bringt den frawen ire zeit
Daucus carota L.	Apiaceae	ren	LF	Karotte	top	GYN	- CCCXCI / CCLXIII	treibt sie die todte frucht
Daucus carota L.	Apiaceae	ren	LF	Karotte	syst	OTH	- CCCXCI / CCLXIII	[ist nützlich zu den gebresten] des milz
Daucus carota L.	Apiaceae	ren	LF	Karotte	syst	URO	- CCCXCI / CCLXIII	treibt den harn / ist gut den wassersüchtigen
Daucus carota L.	Apiaceae	ren	LF	Karotte	syst	URO	- CCCXCI / CCLXIII	treibt den harn
Daucus carota L.	Apiaceae	ren	LF	Karotte	syst	URO	- CCCXCI / CCLXIII	[ist nützlich zu den gebresten] der nieren un lenden
Daucus carota L.	Apiaceae	s pop	JK	Karotte	top	DER	p. 407	frische Wunden, Frostbeulen und aufgebprungene Wunden
Daucus carota L.	Apiaceae	s pop	JK	Karotte	top	DER	p. 407	Eissen, Furunkeln und Brandwunden, mildern sogar Krebs und Lupus
Daucus carota L.	Apiaceae	s pop	JK	Karotte	top	DER	p. 407	Zahnweh, Mundgeschwulsten und Mundfäule
Daucus carota L.	Apiaceae	s pop	JK	Karotte	syst	GAS	p. 407	Würmer, Gelbsucht
Daucus carota L.	Apiaceae	s pop	JK	Karotte	syst	RES	p. 407	Kehlkopfentzündungen und Brustleiden
Daucus carota L.	Apiaceae	s pop	JK	Karotte	syst	RES	p. 407	Brustwassersucht
Daucus carota L.	Apiaceae	s pop	JK	Karotte	syst	RES	p. 407	Schwindsucht der Kehle und deren Entzündung
Daucus carota L.	Apiaceae	s pop	JK	Karotte	syst	TON	p. 407	Stärkung für Schwindsüchtige
Daucus carota L.	Apiaceae	s pop	UB	Karotte	syst	GAS	p. 125	Durchfall
Eryngium campestre L.	Apiaceae	ant	DIOS	Feldmannstreu	syst	ANT	172	die von giftigen Tieren gebissen sind und die tödliche Gifte genossen haben
Eryngium campestre L.	Apiaceae	ant	DIOS	Feldmannstreu	other	DER	172	verteilt es (als Amulett) umgebunden [und als Kataplasma] Geschwülste
Eryngium campestre L.	Apiaceae	ant	DIOS	Feldmannstreu	top	DER	172	verteilt es [(als Amulett) umgebunden] und als Kataplasma Geschwülste
Eryngium campestre L.	Apiaceae	ant	DIOS	Feldmannstreu	syst	GAS	172	vertreibt Leibschneiden und Blähungen. Mit Wein hilft es denen, die an der Leber leiden
Eryngium campestre L.	Apiaceae	ant	DIOS	Feldmannstreu	syst	GYN	172	[fördert] die Menstruation
Eryngium campestre L.	Apiaceae	ant	DIOS	Feldmannstreu	syst	NER	172	Die Wurzel desselben mit Honigmeth getrunken heilt Opisthotonie und Epilepsie
Eryngium campestre L.	Apiaceae	ant	DIOS	Feldmannstreu	syst	OTH	172	jungen Blätter werden in Salzlake eingemacht als Gemüse
Eryngium campestre L.	Apiaceae	ant	DIOS	Feldmannstreu	syst	URO	172	fördert getrunken den Harn
Eryngium campestre L.	Apiaceae	mon	HvB	Feldmannstreu	syst	CAR	1-99	Stechen in seinem Herzen [oder an einer anderen Stelle. . .]
Eryngium campestre L.	Apiaceae	mon	HvB	Feldmannstreu	syst	NER	1-99	Stechen [in seinem Herzen] oder an einer anderen Stelle oder in einem seiner Glieder Schmerzen hat
Eryngium campestre L.	Apiaceae	ren	LF	Mannstreu	syst	ANT	CLXVI / CXII	und denen so gifft getruncken haben und von gifftigen thiern gebissen
Eryngium campestre L.	Apiaceae	ren	LF	Mannstreu	top	DER	CLXVI / CXII	so verzeret sie geschwulst und knollen
Eryngium campestre L.	Apiaceae	ren	LF	Mannstreu	syst	GAS	CLXVI / CXII	stillt das bauchgrimmen und vertreibt die blaest / sie ist auch gut zu den lebersüchtigen
Eryngium campestre L.	Apiaceae	ren	LF	Mannstreu	syst	GAS	CLXVI / CXII	so das darmgicht haben
Eryngium campestre L.	Apiaceae	ren	LF	Mannstreu	syst	GYN	CLXVI / CXII	und bringt den frawen ire zeit
Eryngium campestre L.	Apiaceae	ren	LF	Mannstreu	syst	NER	CLXVI / CXII	so den fallenden siechtagen haben un den krampff
Eryngium campestre L.	Apiaceae	ren	LF	Mannstreu	syst	URO	CLXVI / CXII	treibt den harn
Eryngium campestre L.	Apiaceae	ren	LF	Mannstreu	syst	URO	CLXVI / CXII	für den lendenstein
Eryngium campestre L.	Apiaceae	s pop	VAL	Feldmanstreu	syst	URO	p. 218	propriétés: diurétique
Euphorbia esula agg.	Euphorbiacea	ant	DIOS	Wolfsmilch	top	DER	254	Der frische Saft entfernt auch die Haare
Euphorbia esula agg.	Euphorbiacea	ant	DIOS	Wolfsmilch	top	DER	254	Aufgestrichen vertreibt er ferner Ge- schwüre und gestielte Warzen, sowie Feigwarzen und Flechten bei übergewachsenen Nägeln und Karbunkeln, bei krebsigen, fressenden Geschwüren und Fisteln
Euphorbia esula agg.	Euphorbiacea	ant	DIOS	Wolfsmilch	syst	HUM	254	leisten dasselbe wie der Saft [Purgieren]
Euphorbia esula agg.	Euphorbiacea	ant	DIOS	Wolfsmilch	syst	HUM	254	Der Saft hat die Kraft, den Bauch nach unten zu reinigen . . .indem er Schleim und Galle abführt
Euphorbia esula agg.	Euphorbiacea	ant	DIOS	Wolfsmilch	syst	HUM	254	reinigt den Bauch
Euphorbia esula agg.	Euphorbiacea	ant	DIOS	Wolfsmilch	top	TEE	254	auch in die hohlen Zähne gesteckt, da er die Schmerzen lindert;
Euphorbia esula agg.	Euphorbiacea	ant	DIOS	Wolfsmilch	top	TEE	254	hilft sie bei Zahnschmerzen
Euphorbia esula agg.	Euphorbiacea	mon	HvB	Wolfsmilch	syst	GAS	1-51	bisweilen gegen einen verhärteten Magen gewissen Tränken beigefügt
Euphorbia esula agg.	Euphorbiacea	ren	LF	Wolfsmilch	top	DER	CCCCLXVII / CCCXVI	har, grind und wartzen damit zu vertreiben
Euphorbia esula agg.	Euphorbiacea	ren	LF	Wolfsmilch	top	DER	CCCCLXVII / CCCXVI	zu dem erbgrind
Euphorbia esula agg.	Euphorbiacea	ren	LF	Wolfsmilch	syst	URO	CCCCLXVII / CCCXVI	treibt aus allerley wässrige feüchtigkeyt, darumb seind die wolffsmilch insonderheyt nützlich den wassersüchtigen
Euphorbia esula agg.	Euphorbiacea	s pop	JK	Wolfsmilch	top	DER	p. 445	warzen, Nasenkrebs
Ficus carica L.	Moraceae	ant	DIOS	Feige	top	ANT	113	Skorpionstiche und Bisse von giftigen Tieren und Hunden

Ficus carica L.	Moraceae	ant	DIOS	Feige	top	ANT	113	wirken sie gegen Hundsbiss und bösartigen Wabengrind
Ficus carica L.	Moraceae	ant	DIOS	Feige	top	ANT	113	Linsenwicken und Wein umgeschlagen helfen sie gegen den Biss der Spitzmaus und des Skolopender
Ficus carica L.	Moraceae	ant	DIOS	Feige	syst	ANT	113	gegen den Genuss von Gips und den Biss der Spinne
Ficus carica L.	Moraceae	ant	DIOS	Feige	top	DER	113	Mit Eisenvitriol heilen sie schwer heilbare und bösartige Schienbeinflüsse
Ficus carica L.	Moraceae	ant	DIOS	Feige	top	DER	113	heilen sie Frostbeulen
Ficus carica L.	Moraceae	ant	DIOS	Feige	top	DER	113	beseitigt er Aussatz, Flechten, Sonnenbrandflecken, Krätze, weiße Hautflecken und Schorf; vertreibt er Warzen
Ficus carica L.	Moraceae	ant	DIOS	Feige	top	DER	113	Mit Granatrinde vertreiben sie übergewachsene Nägel
Ficus carica L.	Moraceae	ant	DIOS	Feige	top	DER	113	Verhärtungen und Drüsen, erweichen Furunkeln und bringen Scham- und Achseldrüsengeschwüre
Ficus carica L.	Moraceae	ant	DIOS	Feige	top	DER	113	heilen sie Schorf, Grind und Epinyktis. Auch werden mit ihnen raue und harte Stellen der Augenlider eingerieben
Ficus carica L.	Moraceae	ant	DIOS	Feige	top	DER	113	Grind und Epinyktis. Auch werden mit ihnen raue und harte Stellen der Augenlider eingerieben; Der weiße Aussatz ferner wird mit den Blättern oder zarten Zweigen der schwarzen Feige als Umschlag behandelt; mit Wachs öffnen sie Furunkeln
Ficus carica L.	Moraceae	ant	DIOS	Feige	top	DER	113	Dasselbe leistet aber auch der Saft aus den Zweigen des wilden Feigenbaumes [vertreibt Warzen]
Ficus carica L.	Moraceae	ant	DIOS	Feige	top	DER	113	kaustischen Mitteln und für gangränöse Geschwüre; fistelartigen tief liegenden großen Geschwüren;
Ficus carica L.	Moraceae	ant	DIOS	Feige	top	EAR	113	helfen sie, in die Ohren gesteckt, bei Sausen und Jucken (in den Ohren)
Ficus carica L.	Moraceae	ant	DIOS	Feige	syst	GAS	113	Trocken aber sind sie nahrhaft, erwärmend, mehr Durst machend, wohltuend für den Bauch [Den Bauch erweichen sie]
Ficus carica L.	Moraceae	ant	DIOS	Feige	top	GYN	113	tyrrhonischem Wachs im Zäpfchen appliziert befördert er die Menstruation
Ficus carica L.	Moraceae	ant	DIOS	Feige	top	GYN	113	für Frauen
Ficus carica L.	Moraceae	ant	DIOS	Feige	vol	GYN	113	für Frauen
Ficus carica L.	Moraceae	ant	DIOS	Feige	syst	NER	113	Epileptiker
Ficus carica L.	Moraceae	ant	DIOS	Feige	top	NER	113	bei Nervenleiden und Krämpfen
Ficus carica L.	Moraceae	ant	DIOS	Feige	syst	RES	113	Heilsam sind sie für den Schlund, die Luftröhre; ferner für die Asthmatiker; [reinigen sie die Brust, sind auch ein gutes Mittel bei altem Husten und chronischem Lungenleiden]
Ficus carica L.	Moraceae	ant	DIOS	Feige	top	RES	113	Abkochung derselben ist bei Luftröhren- und Mandelentzündung
Ficus carica L.	Moraceae	ant	DIOS	Feige	top	SKE	113	dient er zu Kataplasmen bei Podagra
Ficus carica L.	Moraceae	ant	DIOS	Feige	top	SKE	113	ziehen auch Knochen heraus
Ficus carica L.	Moraceae	ant	DIOS	Feige	syst	SKE	113	Getrunken wird sie gegen Gerinnsel des Blutes (Blutthromben), bei Sturzverletzungen, Zerreißungen und Krämpfen,
Ficus carica L.	Moraceae	ant	DIOS	Feige	syst	SKE	113	hilft sie Magen- und Dysenteriekranken
Ficus carica L.	Moraceae	ant	DIOS	Feige	top	TEE	113	geträufelt und in den hohlen Zahn gesteckt
Ficus carica L.	Moraceae	ant	DIOS	Feige	syst	TON	113	welche nach langer Krankheit eine schlechte Farbe haben
Ficus carica L.	Moraceae	ant	DIOS	Feige	syst	URO	113	Wassersüchtigen.
Ficus carica L.	Moraceae	ant	DIOS	Feige	syst	URO	113	die Blase und Nieren
Ficus carica L.	Moraceae	ant	DIOS	Feige	top	URO	113	als Umschlag Wassersüchtigen heilsam
Ficus carica L.	Moraceae	mon	HvB	Feigenbaum	top	EYE	3-14	Oder wenn deine Augen schwären
Ficus carica L.	Moraceae	mon	HvB	Feigenbaum	top	NER	3-14	Und wenn Du Kopfweh hast . . .
Ficus carica L.	Moraceae	mon	HvB	Feigenbaum	top	OTH	3-14	Schmerzen in den Lenden
Ficus carica L.	Moraceae	mon	HvB	Feigenbaum	top	RES	3-14	Schmerzen in der Brust hast
Ficus carica L.	Moraceae	mon	HvB	Feigenbaum	syst	TON	3-14	Für den Kranken aber, der schwach am Körper ist, ist (die Frucht) gut zu essen
Ficus carica L.	Moraceae	ren	LF	Feige	top	DER	CCCCXXXIIII / CCXC	rauden, flechten, zittermal, masen und andere ungestalt des angesichts und des ganzen leibs
Ficus carica L.	Moraceae	ren	LF	Feige	top	DER	CCCCXXXIIII / CCXC	vertreibt die wartzen
Ficus carica L.	Moraceae	ren	LF	Feige	top	DER	CCCCXXXIIII / CCXC	heylen sie erfrornen fuess
Ficus carica L.	Moraceae	ren	LF	Feige	top	DER	CCCCXXXIIII / CCXC	von einem scorpion gestochen oder von einem wuetenden hund gebissen
Ficus carica L.	Moraceae	ren	LF	Feige	top	DER	CCCCXXXIIII / CCXC	verzere allerley geschwulst und erweychen di herte
Ficus carica L.	Moraceae	ren	LF	Feige	top	DER	CCCCXXXIIII / CCXC	weychen sie auff die ohrmützel und andere beulen
Ficus carica L.	Moraceae	ren	LF	Feige	top	DER	CCCCXXXIIII / CCXC	verzeren die kroepff, erweychen und zerteylen auch alle andere geschwulst
Ficus carica L.	Moraceae	ren	LF	Feige	syst	DER	CCCCXXXIIII / CCXC	zerteylet das undergrunnen blut
Ficus carica L.	Moraceae	ren	LF	Feige	top	DER	CCCCXXXIIII / CCXC	heylet den brandt
Ficus carica L.	Moraceae	ren	LF	Feige	top	DER	CCCCXXXIIII / CCXC	heylt alerley boese geschwaer
Ficus carica L.	Moraceae	ren	LF	Feige	top	EAR	CCCCXXXIIII / CCXC	benemen das sausen und klingen darin [ohren]
Ficus carica L.	Moraceae	ren	LF	Feige	syst	GAS	CCCCXXXIIII / CCXC	eröffnet auch den stulgang
Ficus carica L.	Moraceae	ren	LF	Feige	syst	GAS	CCCCXXXIIII / CCXC	erweychen sie den bauch
Ficus carica L.	Moraceae	ren	LF	Feige	top	GYN	CCCCXXXIIII / CCXC	bringt er den frawen ihre zeit
Ficus carica L.	Moraceae	ren	LF	Feige	syst	HUM	CCCCXXXIIII / CCXC	etzt auff den leib und eröffnet die ader
Ficus carica L.	Moraceae	ren	LF	Feige	syst	NER	CCCCXXXIIII / CCXC	die fallende sucht haben
Ficus carica L.	Moraceae	ren	LF	Feige	syst	RES	CCCCXXXIIII / CCXC	seind der kelen [gut]
Ficus carica L.	Moraceae	ren	LF	Feige	syst	RES	CCCCXXXIIII / CCXC	so schwerlich athmen
Ficus carica L.	Moraceae	ren	LF	Feige	syst	RES	CCCCXXXIIII / CCXC	zu der rauhen kelen und den geschwollenen mandeln
Ficus carica L.	Moraceae	ren	LF	Feige	syst	RES	CCCCXXXIIII / CCXC	heylen den langwirigen husten unnd andere dergleichen gebresten der lungen
Ficus carica L.	Moraceae	ren	LF	Feige	top	SKE	CCCCXXXIIII / CCXC	bekompt er wol denen so da Podagra haben
Ficus carica L.	Moraceae	ren	LF	Feige	syst	SKE	CCCCXXXIIII / CCXC	hochgefallen und gebrochen seind
Ficus carica L.	Moraceae	ren	LF	Feige	top	TEE	CCCCXXXIIII / CCXC	lindert das zanwee
Ficus carica L.	Moraceae	ren	LF	Feige	syst	TON	CCCCXXXIIII / CCXC	nach langer krankheyt bleych seind
Ficus carica L.	Moraceae	ren	LF	Feige	syst	URO	CCCCXXXIIII / CCXC	wassersüchtig seind
Ficus carica L.	Moraceae	ren	LF	Feige	syst	URO	CCCCXXXIIII / CCXC	[seind] den nieren und blasen gut
Ficus carica L.	Moraceae	ren	LF	Feige	syst	URO	CCCCXXXIIII / CCXC	treiben den sand oder griess auss den nieren
Ficus carica L.	Moraceae	ren	LF	Feige	top	URO	CCCCXXXIIII / CCXC	übergeschlagen seind sie gut den wassersüchtigen
Ficus carica L.	Moraceae	s pop	VOG	Feige	syst	GAS	p. 44	Verstopfung
Ficus carica L.	Moraceae	s pop	VOG	Feige	syst	TON	p. 147	Stärkung Immunsystem
Foeniculum vulgare agg.	Apiaceae	ant	DIOS	Fenchel	syst	ANT	188	Wein getrunken ist er (der Blütenstengel) ein gutes Mittel gegen Schlangenbisse
Foeniculum vulgare agg.	Apiaceae	ant	DIOS	Fenchel	syst	ANT	188	gegen den Biß giftiger Tiere,
Foeniculum vulgare agg.	Apiaceae	ant	DIOS	Fenchel	top	ANT	188	heilen den Biß des tollen Hundes
Foeniculum vulgare agg.	Apiaceae	ant	DIOS	Fenchel	top	EYE	188	Nutzen zu den Augenmitteln, welche für die Schärfe des Gesichtes dienen
Foeniculum vulgare agg.	Apiaceae	ant	DIOS	Fenchel	top	EYE	188	Augenmittel
Foeniculum vulgare agg.	Apiaceae	ant	DIOS	Fenchel	syst	GAS	188	In Fieberzuständen mit kaltem Wasser getrunken beseitigt er das Übelkeitsempfinden und den Brand des Magens
Foeniculum vulgare agg.	Apiaceae	ant	DIOS	Fenchel	syst	GAS	188	stellen den Durchfall; zertrümmern den Stein und vertreiben die Gelbsucht
Foeniculum vulgare agg.	Apiaceae	ant	DIOS	Fenchel	syst	GYN	188	auch befördert er die Menstruation
Foeniculum vulgare agg.	Apiaceae	ant	DIOS	Fenchel	syst	GYN	188	Kraft, die Menstruation zu befördern
Foeniculum vulgare agg.	Apiaceae	ant	DIOS	Fenchel	syst	GYN	188	befördert die Milchabsonderung und reinigt die Frauen nach der Geburt
Foeniculum vulgare agg.	Apiaceae	ant	DIOS	Fenchel	syst	GYN	188	Kraft, die Menstruation zu befördern
Foeniculum vulgare agg.	Apiaceae	ant	DIOS	Fenchel	top	GYN	188	befördert sie die Menstruation
Foeniculum vulgare agg.	Apiaceae	ant	DIOS	Fenchel	syst	URO	188	als Trank den Nieren- und Blasenleidenden zuträglich, da sie den Urin treibt.
Foeniculum vulgare agg.	Apiaceae	ant	DIOS	Fenchel	syst	URO	188	heilt sie Harnzwang
Foeniculum vulgare agg.	Apiaceae	mon	HvB	Fenchel	syst	EYE	1-66	bringt Augen zu klarem Sehen
Foeniculum vulgare agg.	Apiaceae	mon	HvB	Fenchel	syst	GAS	1-66	unterdrückt den üblen Geruch des Atems
Foeniculum vulgare agg.	Apiaceae	mon	HvB	Fenchel	syst	GAS	1-66	kranker Magen
Foeniculum vulgare agg.	Apiaceae	mon	HvB	Fenchel	syst	TON	1-66	den Kranken stärkt es
Foeniculum vulgare agg.	Apiaceae	mon	MF	Fenchel	syst	ANT	17	widersteht sämtlichen Giften, widersteht sie vergifteten Bissen
Foeniculum vulgare agg.	Apiaceae	mon	MF	Fenchel	syst	APH	17	erregt die Tätigkeit der Liebeskraft
Foeniculum vulgare agg.	Apiaceae	mon	MF	Fenchel	top	EAR	17	tötet er gleich die Ohrwürmlein

Foeniculum vulgare agg.	Apiaceae	mon	MF	Fenchel	top	EYE	17	um alle Augenleiden zu vertreiben
Foeniculum vulgare agg.	Apiaceae	mon	MF	Fenchel	top	EYE	17	salbt so die Augen, vertreibt er alle Finsternis und macht sie klar
Foeniculum vulgare agg.	Apiaceae	mon	MF	Fenchel	syst	GAS	17	Beschwerden von Leber
Foeniculum vulgare agg.	Apiaceae	mon	MF	Fenchel	top	GYN	17	geordneter Monatsfluss
Foeniculum vulgare agg.	Apiaceae	mon	MF	Fenchel	syst	GYN	17	schafft Muttermilch bis zum Überfluss
Foeniculum vulgare agg.	Apiaceae	mon	MF	Fenchel	syst	RES	17	Beschwerden von Lunge
Foeniculum vulgare agg.	Apiaceae	mon	MF	Fenchel	top	SKE	17	Schwellungen und Geschwülste durch Stoss und Schlag
Foeniculum vulgare agg.	Apiaceae	mon	MF	Fenchel	syst	TON	17	auch den Greisen nutzen bringt
Foeniculum vulgare agg.	Apiaceae	mon	MF	Fenchel	syst	URO	17	heilt sie die Nieren, macht sie der Aufschwellung der Wassersüchtigen ein Ende
Foeniculum vulgare agg.	Apiaceae	ren	LF	Fenchel	top	ANT	CCLXXXIII / CXCI	hilft denen so von Schlangen gebissen worden sind
Foeniculum vulgare agg.	Apiaceae	ren	LF	Fenchel	n.d.	APH	CCLXXXIII / CXCI	es mehret den natürlichen Samen
Foeniculum vulgare agg.	Apiaceae	ren	LF	Fenchel	n.d.	DER	CCLXXXIII / CXCI	verzert das undergerunnen blut
Foeniculum vulgare agg.	Apiaceae	ren	LF	Fenchel	top	DER	CCLXXXIII / CXCI	hilft denen so von wuetenden hunden gebissen seind
Foeniculum vulgare agg.	Apiaceae	ren	LF	Fenchel	top	EAR	CCLXXXIII / CXCI	in die ohren getan tödt auch die würm darin
Foeniculum vulgare agg.	Apiaceae	ren	LF	Fenchel	top	EYE	CCLXXXIII / CXCI	gut den trueben augen, vertreibt auch den starn, der neulich angefangen hat
Foeniculum vulgare agg.	Apiaceae	ren	LF	Fenchel	syst	GAS	CCLXXXIII / CXCI	kräftigt den schwachen Magen und legt das unwillen, er ist auch trefflich gut der leber
Foeniculum vulgare agg.	Apiaceae	ren	LF	Fenchel	syst	GAS	CCLXXXIII / CXCI	er ist auch trefflich gut der lunge (und der leber)
Foeniculum vulgare agg.	Apiaceae	ren	LF	Fenchel	syst	GYN	CCLXXXIII / CXCI	macht viel milch
Foeniculum vulgare agg.	Apiaceae	ren	LF	Fenchel	top	GYN	CCLXXXIII / CXCI	bringt den frawn ihre zeit
Foeniculum vulgare agg.	Apiaceae	ren	LF	Fenchel	syst	GYN	CCLXXXIII / CXCI	macht viel milch
Foeniculum vulgare agg.	Apiaceae	ren	LF	Fenchel	syst	URO	CCLXXXIII / CXCI	treibt den stein
Foeniculum vulgare agg.	Apiaceae	ren	LF	Fenchel	top	URO	CCLXXXIII / CXCI	allerley gebresten der nieren und blasen, treibt den harn,
Foeniculum vulgare agg.	Apiaceae	ren	LF	Fenchel	syst	URO	CCLXXXIII / CXCI	ist den wassersüchtigen und so den krampff haben sehr dienstlich
Foeniculum vulgare agg.	Apiaceae	s pop	JK	Fenchel	syst	GAS	p. 356	Darmträgheit und Blähungen
Foeniculum vulgare agg.	Apiaceae	s pop	JK	Fenchel	syst	GYN	p. 356	Milchsekretion gefördert
Foeniculum vulgare agg.	Apiaceae	s pop	JK	Fenchel	syst	RES	p. 356	schleimlösend, gegen Husten, Keuchhusten, Erkältungen, Bronchialkatarrh
Foeniculum vulgare agg.	Apiaceae	s pop	KN	Fenchel	top	EYE	p. 466	Augenwasser
Foeniculum vulgare agg.	Apiaceae	s pop	KN	Fenchel	vol	EYE	p. 466	Augenwasser
Foeniculum vulgare agg.	Apiaceae	s pop	KN	Fenchel	syst	GAS	p. 466	Kolik, krampfartige Zustände
Foeniculum vulgare agg.	Apiaceae	s pop	KN	Fenchel	syst	GAS	p. 466	vertreibt die Gase aus dem Magen
Foeniculum vulgare agg.	Apiaceae	s pop	KN	Fenchel	syst	NER	p. 466	Migräne, Scheinepilepsie (die durch Magen-Darm-Störungen bedingt sind)
Foeniculum vulgare agg.	Apiaceae	s pop	KN	Fenchel	syst	RES	p. 466	Lungenkranken
Foeniculum vulgare agg.	Apiaceae	s pop	KN	Fenchel	vol	RES	p. 466	Kopfdampf behufs Lösung im Innern
Foeniculum vulgare agg.	Apiaceae	s pop	UB	Fenchel	syst	GAS	p. 115	gegen Blähungen
Foeniculum vulgare agg.	Apiaceae	s pop	UB	Fenchel	syst	GAS	p. 115	gegen Blähungen
Foeniculum vulgare agg.	Apiaceae	s pop	UB	Fenchel	syst	GAS	p. 115	gegen Blähungen
Foeniculum vulgare agg.	Apiaceae	s pop	UB	Fenchel	syst	RES	p. 216	Auswurfförderung
Foeniculum vulgare agg.	Apiaceae	s pop	UB	Fenchel	syst	RES	p. 216	Auswurfförderung
Foeniculum vulgare agg.	Apiaceae	s pop	UB	Fenchel	syst	RES	p. 216	Auswurfförderung
Foeniculum vulgare agg.	Apiaceae	s pop	UB	Fenchel	syst	RES	p. 216	Auswurfförderung
Foeniculum vulgare agg.	Apiaceae	sci	EMA	Fenchel	syst	GAS	HMPC/428963/2006	mild, spasmodic gastrointestinal complaints including bloating and flatulence
Foeniculum vulgare agg.	Apiaceae	sci	EMA	Fenchel	syst	GYN	HMPC/428963/2006	minor spasms associated with menstrual periods
Foeniculum vulgare agg.	Apiaceae	sci	EMA	Fenchel	syst	RES	HMPC/263292/2006	expectorant in cough and cold
Foeniculum vulgare agg.	Apiaceae	sci	EMA	Fenchel	syst	RES	HMPC/428963/2006	expectorant in cough and cold
Foeniculum vulgare agg.	Apiaceae	sci	WI	Fenchel	syst	RES	p. 219	Expectorans
Fumaria officinalis agg.	Papaveraceae	ant	DIOS	Erdrauch	top	DER	p. 240	Er hat die Kraft, das Wiederwachsen der aus den Augenlidern ausgezogenen Haare zu verhindern, wenn er mit Gummi aufgestrichen wird.
Fumaria officinalis agg.	Papaveraceae	ant	DIOS	Erdrauch	n.d.	EYE	p. 240	schärft das Gesicht und reizt zu Tränen
Fumaria officinalis agg.	Papaveraceae	ant	DIOS	Erdrauch	syst	URO	p. 240	Genossen treibt das Kraut den galligen Harn.
Fumaria officinalis agg.	Papaveraceae	mon	LO	Erdrauch	n.d.	OTH	n.d.	n.d.
Fumaria officinalis agg.	Papaveraceae	ren	LF	Erdrauch	syst	DER	CXCI / CXXVII	ist auch nützlich denen so mit der raeuden und anderen boesen grinden beladen seind
Fumaria officinalis agg.	Papaveraceae	ren	LF	Erdrauch	top	DER	CXCI / CXXVII	verhuetet er das an den augenbrawen zwyfache hoernlin wachsen
Fumaria officinalis agg.	Papaveraceae	ren	LF	Erdrauch	n.d.	EYE	CXCI / CXXVII	scherfft das gesicht und macht klare augen, macht weynen
Fumaria officinalis agg.	Papaveraceae	ren	LF	Erdrauch	syst	GAS	CXCI / CXXVII	treibt die gallen durch den harn aus und heylet die verstopfung der leber und derselbigen schwachheyt / das pulver mit meth getrunken treibt durch den stuhlgang / mit geringem wein eingenommen sterckt den magen
Fumaria officinalis agg.	Papaveraceae	s pop	JK	Erdrauch	syst	CAR	p. 352	Arterienverkalkung und Überröti
Fumaria officinalis agg.	Papaveraceae	s pop	JK	Erdrauch	syst	DER	p. 352	Ausschläge aller Art, Milchschorf
Fumaria officinalis agg.	Papaveraceae	s pop	JK	Erdrauch	top	DER	p. 352	Flechten und Mundfäule
Fumaria officinalis agg.	Papaveraceae	s pop	JK	Erdrauch	syst	GAS	p. 352	Leberleiden, Milzleiden
Fumaria officinalis agg.	Papaveraceae	s pop	UB	Erdrauch	top	DER	p. 340	Psoriasis
Fumaria officinalis agg.	Papaveraceae	s pop	UB	Erdrauch	top	DER	p. 340	Psoriasis
Fumaria officinalis agg.	Papaveraceae	s pop	WI	Erdrauch	syst	DER	p. 235	Volksmedizin: bei Hautleiden
Fumaria officinalis agg.	Papaveraceae	s pop	WI	Erdrauch	syst	URO	p. 235	Volksmedizin: diuretisch, laxierend
Fumaria officinalis agg.	Papaveraceae	sci	EMA	Erdrauch	syst	GAS	HPMC/574766/2010	increase bile flow for the relief of symptoms of indigestion
Fumaria officinalis agg.	Papaveraceae	sci	EMA	Erdrauch	syst	GAS	HPMC/574766/2010	increase bile flow for the relief of symptoms of indigestion
Fumaria officinalis agg.	Papaveraceae	sci	EMA	Erdrauch	syst	GAS	HPMC/574766/2010	increase bile flow for the relief of symptoms of indigestion
Fumaria officinalis agg.	Papaveraceae	sci	EMA	Erdrauch	syst	GAS	HPMC/574766/2010	increase bile flow for the relief of symptoms of indigestion
Fumaria officinalis agg.	Papaveraceae	sci	EMA	Erdrauch	syst	GAS	HPMC/574766/2010	increase bile flow for the relief of symptoms of indigestion
Fumaria officinalis agg.	Papaveraceae	sci	ESCOP	Fumariae herba	syst	GAS	II p. 121	digestive complaints (e.g. stomach ache, nausea, vomiting, feeling of fullness, flatulence) due to hepatobiliary disturbance
Gentiana lutea agg.	Gentianaceae	ant	DIOS	Gelber Enzian	syst	ANT	167	gegen den Biß giftiger Tiere
Gentiana lutea agg.	Gentianaceae	ant	DIOS	Gelber Enzian	top	DER	167	Wundmittel, auch ein Heilmittel bei fistelartig hinkriechenden fressenden Geschwüren; Wurzel entfernt auch weiße Flecken
Gentiana lutea agg.	Gentianaceae	ant	DIOS	Gelber Enzian	syst	EYE	167	der ausgezogene Saft, auch als Salbe bei Augenentzündungen
Gentiana lutea agg.	Gentianaceae	ant	DIOS	Gelber Enzian	syst	GAS	167	Leber- und Magenkranken
Gentiana lutea agg.	Gentianaceae	ant	DIOS	Gelber Enzian	syst	GYN	167	treibt sie den Embryo aus
Gentiana lutea agg.	Gentianaceae	ant	DIOS	Gelber Enzian	syst	n.d.	167	gegen Seitenschmerzen
Gentiana lutea agg.	Gentianaceae	ant	DIOS	Gelber Enzian	syst	SKE	167	bei Sturzverletzungen, inneren Rupturen und Krämpfen
Gentiana lutea agg.	Gentianaceae	mon	HvB	Gelber Enzian	syst	CAR	1-31	Schmerz des Herzens
Gentiana lutea agg.	Gentianaceae	mon	HvB	Gelber Enzian	syst	GAS	1-31	Fieber im Magen
Gentiana lutea agg.	Gentianaceae	ren	LF	Gelber Enzian	syst	ANT	CXI / LXXIIII	denen von thieren gebissen sind
Gentiana lutea agg.	Gentianaceae	ren	LF	Gelber Enzian	top	DER	CXI / LXXIIII	wunden, seer tieff seind und um sich fressen
Gentiana lutea agg.	Gentianaceae	ren	LF	Gelber Enzian	top	DER	CXI / LXXIIII	dient zu allerley ungestalt und befleckung der haut
Gentiana lutea agg.	Gentianaceae	ren	LF	Gelber Enzian	top	EYE	CXI / LXXIIII	über die augen geschlagen, leschet er hie hitz derselbigen
Gentiana lutea agg.	Gentianaceae	ren	LF	Gelber Enzian	syst	GAS	CXI / LXXIIII	lebersüchtigen, und denen so schwachen magen haben
Gentiana lutea agg.	Gentianaceae	ren	LF	Gelber Enzian	top	GYN	CXI / LXXIIII	treibt aus die tot geburt
Gentiana lutea agg.	Gentianaceae	ren	LF	Gelber Enzian	syst	SKE	CXI / LXXIIII	vertreibt das seitenwee, bekommt wohl denen so hoch herabgefallen oder inwendig gebrochen seind, dass erzerteylet oder führet aus das gerunneblut.
Gentiana lutea agg.	Gentianaceae	s pop	KN	Gelber Enzian	syst	GAS	p. 464	Magenmittel, Magendrücken, aufgetriebenen Magen, Aufstossen, Sodbrennen
Gentiana lutea agg.	Gentianaceae	s pop	KN	Gelber Enzian	syst	GAS	p. 464	Magenmittel, Magendrücken, aufgetriebenen Magen, Aufstossen, Sodbrennen
Gentiana lutea agg.	Gentianaceae	s pop	KN	Gelber Enzian	syst	GAS	p. 464	Magenmittel, Magendrücken, aufgetriebenen Magen, Aufstossen, Sodbrennen
Gentiana lutea agg.	Gentianaceae	s pop	KN	Gelber Enzian	syst	GAS	p. 464	Übelkeiten und Anfälle von Ohnmachten
Gentiana lutea agg.	Gentianaceae	s pop	KN	Gelber Enzian	syst	TON	p. 464	Blutarmut und Bleichsucht (blutbildende Wirkung der Bitterstoffe)
Gentiana lutea agg.	Gentianaceae	s pop	UB	Gelber Enzian	syst	GAS	p. 153	Appetitlosigkeit und funktionelle Verdauungsstörung
Gentiana lutea agg.	Gentianaceae	s pop	UB	Gelber Enzian	syst	GAS	p. 153	Appetitlosigkeit und funktionelle Verdauungsstörung
Gentiana lutea agg.	Gentianaceae	s pop	UB	Gelber Enzian	syst	GAS	p. 153	Appetitlosigkeit und funktionelle Verdauungsstörung
Gentiana lutea agg.	Gentianaceae	sci	EMA	Gelber Enzian	syst	GAS	HMPC/578324/2008	Traditional herbal medicinal product used in mild dyspeptic/gastrointestinal disorders, and/or in temporary loss of appetite.

Gentiana lutea agg.	Gentianaceae	sci	EMA	Gelber Enzian	syst	GAS	HMPC/578324/2008	Traditional herbal medicinal product used in mild dyspeptic/gastrointestinal disorders, and/or in temporary loss of appetite.
Gentiana lutea agg.	Gentianaceae	sci	WI	Gelber Enzian	syst	GAS	p. 255	kräftiges Bittermittel
Gentiana lutea agg.	Gentianaceae	sci	WI	Gelber Enzian	syst	TON	p. 255	Roborans und Tonikum
Hedera helix L.	Araliaceae	ant	DIOS	Efeu	syst	ANT	162	hilft gegen den Biss der Spinnen.
Hedera helix L.	Araliaceae	ant	DIOS	Efeu	top	DER	162	Brandwunden
Hedera helix L.	Araliaceae	ant	DIOS	Efeu	top	DER	162	Wein gekocht dienen als Umschlag auf jedes Geschwür; böse Brandwunden und Sonnenbrandflecken heilen sie
Hedera helix L.	Araliaceae	ant	DIOS	Efeu	top	DER	162	Saft in die Nasenlöcher getröpfelt beseitigt schlechten Geruch und faulige Geschwüre.
Hedera helix L.	Araliaceae	ant	DIOS	Efeu	top	EAR	162	Ohrenschmerzen und eiterflüssige Ohren heilt er mit Öl
Hedera helix L.	Araliaceae	ant	DIOS	Efeu	syst	GAS	162	Dysenterie
Hedera helix L.	Araliaceae	ant	DIOS	Efeu	syst	GYN	162	befördern die Katamenien
Hedera helix L.	Araliaceae	ant	DIOS	Efeu	syst	GYN	162	bewirken sie Unfruchtbarkeit
Hedera helix L.	Araliaceae	ant	DIOS	Efeu	syst	GYN	162	in die Gebärmutter gelegt, befördert die Menstruation und treibt den Embryo aus
Hedera helix L.	Araliaceae	ant	DIOS	Efeu	top	NER	162	zum Begießen (des Kopfes) angewandt
Hedera helix L.	Araliaceae	ant	DIOS	Efeu	top	NER	162	in die Nase gebracht, er hilft dann gegen chronische Kopfschmerzen
Hedera helix L.	Araliaceae	ant	DIOS	Efeu	syst	OTH	162	greift die Nerven an, bewirkt Schlaffheit und im Übermaß Erschütterung des Verstandes
Hedera helix L.	Araliaceae	ant	DIOS	Efeu	top	OTH	162	färben die Fruchtdolden die Haare schwarz.
Hedera helix L.	Araliaceae	ant	DIOS	Efeu	top	OTH	162	entfernt die Haare und tötet, eingeschmiert, die Läuse.
Hedera helix L.	Araliaceae	ant	DIOS	Efeu	syst	OTH	162	sind für die Milz heilsam
Hedera helix L.	Araliaceae	ant	DIOS	Efeu	syst	TEE	162	in das dem leidenden Zahn gegenüberliegende Ohr getröpfelt, so lindern sie die Zahnschmerzen
Hedera helix L.	Araliaceae	mon	HvB	Efeu	top	GAS	1-140	Gelbsucht
Hedera helix L.	Araliaceae	ren	LF	Efeu	syst	ANT	+ CCXXXVIII / CLX	gut denen so vonn gifftigen thieren gebissen seind
Hedera helix L.	Araliaceae	ren	LF	Efeu	top	DER	+ CCXXXVIII / CLX	gut übergelegt dem brand
Hedera helix L.	Araliaceae	ren	LF	Efeu	top	DER	+ CCXXXVIII / CLX	machen schwarzes Haar
Hedera helix L.	Araliaceae	ren	LF	Efeu	top	DER	+ CCXXXVIII / CLX	zu allerley wunden und schaeden; vertreiben sie die maeler under dem angesicht und heylen den brand krefftiglich
Hedera helix L.	Araliaceae	ren	LF	Efeu	top	EAR	+ CCXXXVIII / CLX	gut zu den weetagen der ohren und so sie schwaeren
Hedera helix L.	Araliaceae	ren	LF	Efeu	syst	GAS	+ CCXXXVIII / CLX	heylen die rhur
Hedera helix L.	Araliaceae	ren	LF	Efeu	syst	GAS	+ CCXXXVIII / CLX	treiben auss die würm im leib
Hedera helix L.	Araliaceae	ren	LF	Efeu	top	GAS	+ CCXXXVIII / CLX	treiben auss die würm im leib
Hedera helix L.	Araliaceae	ren	LF	Efeu	top	GYN	+ CCXXXVIII / CLX	bringen den frawen ihre kranckheyt
Hedera helix L.	Araliaceae	ren	LF	Efeu	top	NER	+ CCXXXVIII / CLX	über das haupt geschlagen nommt die weetagen desselbigen
Hedera helix L.	Araliaceae	ren	LF	Efeu	top	OTH	+ CCXXXVIII / CLX	vertreibt die leuss
Hedera helix L.	Araliaceae	ren	LF	Efeu	top	OTH	+ CCXXXVIII / CLX	und über das milz geschlagen heylen die gebrechen desselbigen
Hedera helix L.	Araliaceae	ren	LF	Efeu	top	RES	+ CCXXXVIII / CLX	treibt den boesen gestank und reyniget die geschwaer darinn
Hedera helix L.	Araliaceae	ren	LF	Efeu	syst	URO	+ CCXXXVIII / CLX	treiben den stein
Hedera helix L.	Araliaceae	s pop	UB	Efeu	syst	RES	p. 220	Auswurfförderung
Hedera helix L.	Araliaceae	sci	EMA	Efeu	syst	RES	HMPC/289430/2009	Traditional herbal medicinal product used as an expectorant in cough associated with cold.
Hedera helix L.	Araliaceae	sci	EMA	Efeu	syst	RES	HMPC/289430/2009	Well-established-use: Herbal preparations in solid or liquid dosage forms for oral use.
Helleborus spp.	Ranunculacea	ant	DIOS	Schwarze Nieswurz	other	APO	249	Man streut ihn auch in den Wohnungen umher, weil man ihn für reinigend hält
Helleborus spp.	Ranunculacea	ant	DIOS	Schwarze Nieswurz	top	CAR	249	Mit Gerstenmehl und Wein gibt er ein heilsames Kataplasma für Wassersüchtige
Helleborus spp.	Ranunculacea	ant	DIOS	Schwarze Nieswurz	top	DER	249	Fisteln reinigt er, wenn er eingelegt
Helleborus spp.	Ranunculacea	ant	DIOS	Schwarze Nieswurz	top	DER	249	Die Krätze heilt er in einer Salbe mit Weihrauch oder Wachs, Theer und Cedernöl. Für sich allein mit Essig als Kataplasma heilt er weisse Flecken, Flechten und Aussatz
Helleborus spp.	Ranunculacea	ant	DIOS	Schwarze Nieswurz	top	EAR	249	In gleicher Weise wird er bei Schwerhörigkeit in die Ohren gesteckt
Helleborus spp.	Ranunculacea	ant	DIOS	Schwarze Nieswurz	syst	GYN	249	befördert er die Katamenien und tödtet den Embryo
Helleborus spp.	Ranunculacea	ant	DIOS	Schwarze Nieswurz	other	HUM	249	Wenn er neben die Wurzeln des Weinstockes gepflanzt wird, so macht er den aus diesen gewonnenen Wein purgirend
Helleborus spp.	Ranunculacea	ant	DIOS	Schwarze Nieswurz	syst	HUM	249	reinigt nach unten den Bauch, indem er Schleim und Galle abführt
Helleborus spp.	Ranunculacea	ant	DIOS	Schwarze Nieswurz	syst	NER	249	Er hilft bei Epilepsie, Melancholie, Wuthanfällen, [Gicht] und Paralyse
Helleborus spp.	Ranunculacea	ant	DIOS	Schwarze Nieswurz	n.d.	OTH	249	Auch den fäulnisswidrigen Mitteln wird er zugesetzt
Helleborus spp.	Ranunculacea	ant	DIOS	Schwarze Nieswurz	syst	SKE	249	Er hilft bei [Epilepsie, Melancholie, Wuthanfällen,] Gicht [und Paralyse]
Helleborus spp.	Ranunculacea	ant	DIOS	Schwarze Nieswurz	top	TEE	249	als Mundspülwasser lindert er Zahnschmerzen
Helleborus spp.	Ranunculacea	mon	MF	Schwarze Nieswurz	top	CAR	57	trocknest du eine geschwollene Wassersucht aus
Helleborus spp.	Ranunculacea	mon	MF	Schwarze Nieswurz	syst	CAR	57	hilft bei Wassersucht
Helleborus spp.	Ranunculacea	mon	MF	Schwarze Nieswurz	top	DER	57	behebt die Schwellung der Fisteln
Helleborus spp.	Ranunculacea	mon	MF	Schwarze Nieswurz	top	DER	57	entfernt die Male auf der Haut, renigt den Aussatz und vertreibt alle Art von Krätze
Helleborus spp.	Ranunculacea	mon	MF	Schwarze Nieswurz	top	DER	57	verhärtete Geschwülste, die vereitert sind
Helleborus spp.	Ranunculacea	mon	MF	Schwarze Nieswurz	top	EAR	57	gibt das Gehör zurück
Helleborus spp.	Ranunculacea	mon	MF	Schwarze Nieswurz	syst	EYE	57	macht trübe Augen hellsichtig
Helleborus spp.	Ranunculacea	mon	MF	Schwarze Nieswurz	syst	FEV	57	vertreibt Fieber
Helleborus spp.	Ranunculacea	mon	MF	Schwarze Nieswurz	top	GYN	57	sorgt für georndeten Monatsfluss und treibt die Leibesfrucht ab
Helleborus spp.	Ranunculacea	mon	MF	Schwarze Nieswurz	syst	NER	57	heilt sie die Wahnwitzigen
Helleborus spp.	Ranunculacea	mon	MF	Schwarze Nieswurz	syst	SKE	57	nützt der Fussgicht
Helleborus spp.	Ranunculacea	mon	MF	Schwarze Nieswurz	syst	SKE	57	der Gelähmte fühlt ganz wunderbare Hilfe
Helleborus spp.	Ranunculacea	mon	MF	Schwarze Nieswurz	syst	SKE	57	behebt verschiedene Leiden der Gelenke
Helleborus spp.	Ranunculacea	mon	MF	Schwarze Nieswurz	top	TEE	57	soll ausserordentlich den Zahnschmerz lindern
Helleborus spp.	Ranunculacea	ren	LF	Christrose	syst	CAR	CLII + CLIII / CV	wassersucht
Helleborus spp.	Ranunculacea	ren	LF	Christrose	top	DER	CLII + CLIII / CV	alte schaeden oder fisteln
Helleborus spp.	Ranunculacea	ren	LF	Christrose	top	DER	CLII + CLIII / CV	heylet sie alle boese grind, rauden, flechten und gergleychen ungestalt des leibs
Helleborus spp.	Ranunculacea	ren	LF	Christrose	top	GYN	CLII + CLIII / CV	bringt sie den frawen ire zeyt mit gewalt
Helleborus spp.	Ranunculacea	ren	LF	Christrose	syst	NER	CLII + CLIII / CV	mit der fallenden sucht beladen, unsinnig oder wanwitzig seind, . . . Leme der glieder
Helleborus spp.	Ranunculacea	s pop	VAL	Schwarze Nieswurz	syst	GYN	p. 304	absence de règles
Heracleum sphondylium agg.	Apiaceae	ant	DIOS	Bärenklau	top	ANT	190	mit Raute als Umschlag heilt sie Schlangenbisse
Heracleum sphondylium agg.	Apiaceae	ant	DIOS	Bärenklau	top	DER	190	Ringsum abgeschabt und eingelegt bringt sie Wulste in Fisteln zum Verschwinden
Heracleum sphondylium agg.	Apiaceae	ant	DIOS	Bärenklau	top	EAR	190	ein gutes Mittel gegen geschwürige und eiterflüssige Ohren
Heracleum sphondylium agg.	Apiaceae	ant	DIOS	Bärenklau	syst	GAS	190	weiter heilt sie, getrunken, Leberleiden, Gelbsucht
Heracleum sphondylium agg.	Apiaceae	ant	DIOS	Bärenklau	syst	GAS	190	Auch die Wurzel wird Gelbsüchtigen und Leberleidenden gegeben
Heracleum sphondylium agg.	Apiaceae	ant	DIOS	Bärenklau	syst	GYN	190	weiter heilt sie, getrunken, [] Mutterkrämpfe
Heracleum sphondylium agg.	Apiaceae	ant	DIOS	Bärenklau	syst	HUM	190	Seine Frucht scheidet, getrunken, den Schleim durch den Stuhlgang aus
Heracleum sphondylium agg.	Apiaceae	ant	DIOS	Bärenklau	syst	NER	190	weiter heilt sie, getrunken, [] Epilepsie
Heracleum sphondylium agg.	Apiaceae	ant	DIOS	Bärenklau	top	NER	190	gutes Mittel für solche, die an Gehirnkrankheit, Lethargie und Kopfschmerzen leiden
Heracleum sphondylium agg.	Apiaceae	ant	DIOS	Bärenklau	vol	NER	190	In der Räucherung weckt sie die von Schlafsucht Befallenen auf.
Heracleum sphondylium agg.	Apiaceae	ant	DIOS	Bärenklau	syst	OTH	190	weiter heilt sie, getrunken, [] Orthopnöe
Heracleum sphondylium agg.	Apiaceae	mon	LO	Wiesenbärenklau	n.d.	OTH		n.d.
Heracleum sphondylium agg.	Apiaceae	ren	LF	Wiesenbärenklau	top	DER	XXIX - XXX / XV	seind nutzlich zum brandt
Heracleum sphondylium agg.	Apiaceae	ren	LF	Wiesenbärenklau	syst	GAS	XXIX - XXX / XV	stellen den stulgang
Heracleum sphondylium agg.	Apiaceae	ren	LF	Wiesenbärenklau	syst	RES	XXIX - XXX / XV	den schwindsichtigen und gebrochnen
Heracleum sphondylium agg.	Apiaceae	ren	LF	Wiesenbärenklau	top	SKE	XXIX - XXX / XV	podagra
Heracleum sphondylium agg.	Apiaceae	ren	LF	Wiesenbärenklau	top	SKE	XXIX - XXX / XV	denen die gleych verruckt und ausseinander seind
Heracleum sphondylium agg.	Apiaceae	ren	LF	Wiesenbärenklau	syst	URO	XXIX - XXX / XV	treiben den harn
Heracleum sphondylium agg.	Apiaceae	s pop	VAL	Wiesenbärenklau	syst	APH	p. 172	aphrodisiaque, asthénie sexuelle
Heracleum sphondylium agg.	Apiaceae	s pop	VAL	Wiesenbärenklau	syst	CAR	p. 172	hypertension artérielle
Heracleum sphondylium agg.	Apiaceae	s pop	VAL	Wiesenbärenklau	top	DER	p. 172	oedèmes, tumeurs, abcès, furoncles, ulcères atones

Heracleum sphondylium agg.	Apiaceae	s pop	VAL	Wiesenbärenklau	syst	GAS	p. 172	digestions pénible, météorisme
Heracleum sphondylium agg.	Apiaceae	s pop	VAL	Wiesenbärenklau	syst	NER	p. 172	épilepsie
Heracleum sphondylium agg.	Apiaceae	s pop	VAL	Wiesenbärenklau	syst	OTH	p. 172	blenorragie (gonorrhoe)
Heracleum sphondylium agg.	Apiaceae	s pop	VAL	Wiesenbärenklau	syst	URO	p. 172	insuffisamce rénale
Hordeum vulgare agg.	Poaceae	ant	DIOS	Gerste	syst	DER	136	gegen Schärfe, gegen Rauheit und Geschwüre der Luftröhre
Hordeum vulgare agg.	Poaceae	ant	DIOS	Gerste	top	DER	136	gekochte Schleim bildet Eiter
Hordeum vulgare agg.	Poaceae	ant	DIOS	Gerste	top	DER	136	reift es Drüsen
Hordeum vulgare agg.	Poaceae	ant	DIOS	Gerste	top	DER	136	heilt es Aussatz
Hordeum vulgare agg.	Poaceae	ant	DIOS	Gerste	top	DER	136	zerteilt Eitergeschwüre und Geschwülste, es bringt ferner mit Pech, Harz und Taubenmist Verhärtungen zur Reife
Hordeum vulgare agg.	Poaceae	ant	DIOS	Gerste	syst	GAS	136	macht geschmeidig, treibt die Blähungen, ist dem Magen nicht zuträglich
Hordeum vulgare agg.	Poaceae	ant	DIOS	Gerste	syst	GAS	136	bereiteten Graupen stellen den Bauch und lindern Entzündungen
Hordeum vulgare agg.	Poaceae	ant	DIOS	Gerste	top	GAS	136	dient es als Umschlag bei Aufblähen der Eingeweide
Hordeum vulgare agg.	Poaceae	ant	DIOS	Gerste	top	GAS	136	stellt es den Bauchfluss
Hordeum vulgare agg.	Poaceae	ant	DIOS	Gerste	syst	GYN	136	mit Fenchelsamen gekocht und geschlürft, die Milchabsonderung
Hordeum vulgare agg.	Poaceae	ant	DIOS	Gerste	top	n.d.	136	Gelenkflüssen angebracht.
Hordeum vulgare agg.	Poaceae	ant	DIOS	Gerste	top	NER	136	Mit Steinklee hilft es bei Kopf und Brustschmerzen
Hordeum vulgare agg.	Poaceae	ant	DIOS	Gerste	syst	OTH	136	reift die Ödeme
Hordeum vulgare agg.	Poaceae	ant	DIOS	Gerste	top	SKE	136	Podagraanschwellungen
Hordeum vulgare agg.	Poaceae	ant	DIOS	Gerste	syst	URO	136	treibt den Harn
Hordeum vulgare agg.	Poaceae	mon	HvB	Gerste	top	DER	1-4	aber wer im Gesicht eine harte und rauhe Haut hat
Hordeum vulgare agg.	Poaceae	mon	HvB	Gerste	top	n.d.	1-4	und wenn der Kopf des Menschen krank ist
Hordeum vulgare agg.	Poaceae	mon	HvB	Gerste	syst	OTH	1-4	so krank ist, dass er kein Brot essen kann
Hordeum vulgare agg.	Poaceae	mon	HvB	Gerste	top	TON	1-4	Aber der Kranke, der schon am ganzen Körper ermattet ist, ...
Hordeum vulgare agg.	Poaceae	ren	LF	Gerste	top	DER	/I - CCXLVII / CLXVI	erweycht mit bech, hartz un taubenkot vermischt allerley herte geschwulst
Hordeum vulgare agg.	Poaceae	ren	LF	Gerste	top	DER	/I - CCXLVII / CLXVI	zerteylet und verzert geschwulst und geschwaer
Hordeum vulgare agg.	Poaceae	ren	LF	Gerste	top	DER	/I - CCXLVII / CLXVI	vertreibt sie die rossmucken undem dem angesicht
Hordeum vulgare agg.	Poaceae	ren	LF	Gerste	top	DER	/I - CCXLVII / CLXVI	aussetzigen
Hordeum vulgare agg.	Poaceae	ren	LF	Gerste	top	DER	/I - CCXLVII / CLXVI	macht zeitig die kroepff
Hordeum vulgare agg.	Poaceae	ren	LF	Gerste	top	GAS	/I - CCXLVII / CLXVI	vertreibt die blaest und wind im bauch
Hordeum vulgare agg.	Poaceae	ren	LF	Gerste	top	GAS	/I - CCXLVII / CLXVI	stellt es den bauchfluss
Hordeum vulgare agg.	Poaceae	ren	LF	Gerste	syst	GYN	/I - CCXLVII / CLXVI	mehret und bringt den weibern die versiegene milch wider
Hordeum vulgare agg.	Poaceae	ren	LF	Gerste	top	n.d.	/I - CCXLVII / CLXVI	über die Seiten gelegt legt es den Schmerzen derselbigen
Hordeum vulgare agg.	Poaceae	ren	LF	Gerste	top	SKE	/I - CCXLVII / CLXVI	dem hitzigen podagram
Hordeum vulgare agg.	Poaceae	ren	LF	Gerste	top	SKE	/I - CCXLVII / CLXVI	zu den flüssen die in die gleych gon
Hordeum vulgare agg.	Poaceae	ren	LF	Gerste	top	SKE	/I - CCXLVII / CLXVI	gut zu den verzuckten glidern
Hordeum vulgare agg.	Poaceae	s pop	VOG	Gerste	syst	TON	p. 665, 89	Stärkungmittel, Kleinkindernahrung, Leberdiät
Hyoscyamus niger L.	Solanaceae	ant	DIOS	Bilsenkraut	top	DER	299	angeschwollenen Hoden
Hyoscyamus niger L.	Solanaceae	ant	DIOS	Bilsenkraut	syst	EAR	299	gegen Ohrenschmerzen
Hyoscyamus niger L.	Solanaceae	ant	DIOS	Bilsenkraut	syst	FEV	299	heilen bösartige Fieber
Hyoscyamus niger L.	Solanaceae	ant	DIOS	Bilsenkraut	syst	GYN	299	bei Fluss der Frauen und sonstigem Blutverlust
Hyoscyamus niger L.	Solanaceae	ant	DIOS	Bilsenkraut	syst	NER	299	schmerzstillenden Kollyrien
Hyoscyamus niger L.	Solanaceae	ant	DIOS	Bilsenkraut	syst	NER	299	sowie gegen heftigen und heissen Fluss
Hyoscyamus niger L.	Solanaceae	ant	DIOS	Bilsenkraut	syst	NER	299	Gebärmutterleiden
Hyoscyamus niger L.	Solanaceae	ant	DIOS	Bilsenkraut	syst	NER	299	Augen-, Fuss- und sonstige Entzündungen
Hyoscyamus niger L.	Solanaceae	ant	DIOS	Bilsenkraut	top	NER	299	schmerzstillenden Arzneien
Hyoscyamus niger L.	Solanaceae	ant	DIOS	Bilsenkraut	top	NER	299	und nach der Niederkunft entzündeten Brüsten
Hyoscyamus niger L.	Solanaceae	ant	DIOS	Bilsenkraut	syst	OTH	299	bewirken sie gelinden Wahnsinn, der ein Geschwür im Kolon hat, im Klystier beibringt, dieselbe Wirkung eintritt
Hyoscyamus niger L.	Solanaceae	ant	DIOS	Bilsenkraut	syst	RES	299	wirksam ist bei Husten, Katarrh, Fluss und heftigen Schmerzen der Augen
Hyoscyamus niger L.	Solanaceae	ant	DIOS	Bilsenkraut	syst	SKE	299	ein gutes Mittel bei Podagra
Hyoscyamus niger L.	Solanaceae	ant	DIOS	Bilsenkraut	top	TEE	299	lindert als Mundspülwasser Zahnschmerzen
Hyoscyamus niger L.	Solanaceae	mon	HvB	Bilsenkraut	top	DER	1-110	Aber wo Finnen im Menschen sind, so dass sein Fleisch geschwürig machen
Hyoscyamus niger L.	Solanaceae	mon	HvB	Bilsenkraut	top	DER	1-110	Aber wo an einer Stelle der Glieder des Menschen zu grosse Hitze entsteht
Hyoscyamus niger L.	Solanaceae	mon	HvB	Bilsenkraut	top	OTH	1-110	Damit aber ein Betrunkener wieder zu sich kommt, lege er Bilsenkraut in kaltes Wasser, und befeuchte seine Stirn, Schläfen und Kehle damit . . .
Hyoscyamus niger L.	Solanaceae	mon	MF	Bilsenkraut	top	ANT	61	vielerlei Pflastern und Antidoten oder Gegengiften
Hyoscyamus niger L.	Solanaceae	mon	MF	Bilsenkraut	top	DER	61	beliebige Geschwülste
Hyoscyamus niger L.	Solanaceae	mon	MF	Bilsenkraut	top	EAR	61	tötet Ohrwürlein und pflegt verschiedenartige Ohrenschmerzen zu lindern
Hyoscyamus niger L.	Solanaceae	mon	MF	Bilsenkraut	top	EYE	61	zähklebrigen Säftefluss bezähmen, wenn man die Augen des Leidenden oft damit salbt
Hyoscyamus niger L.	Solanaceae	mon	MF	Bilsenkraut	syst	GAS	61	Blutspeiende damit geheilt werden können
Hyoscyamus niger L.	Solanaceae	mon	MF	Bilsenkraut	syst	GYN	61	für Frauen nützlich, die einen Säftefluss aus der Gebärmutter erleiden
Hyoscyamus niger L.	Solanaceae	mon	MF	Bilsenkraut	top	GYN	61	geschwollene Brüste
Hyoscyamus niger L.	Solanaceae	mon	MF	Bilsenkraut	top	SKE	61	Fussgicht
Hyoscyamus niger L.	Solanaceae	mon	MF	Bilsenkraut	top	TEE	61	selbst unmässigen Zahnschmerzen beruhigen
Hyoscyamus niger L.	Solanaceae	mon	MF	Bilsenkraut	top	URO	61	geschwollene Hoden behandeln
Hyoscyamus niger L.	Solanaceae	ren	LF	Bilsenkraut	top	DER	CLXXVII / CCCXXIIII	trucken nieder und treiben hinder sich allerley geschwulst
Hyoscyamus niger L.	Solanaceae	ren	LF	Bilsenkraut	top	EAR	CLXXVII / CCCXXIIII	legt und stillt die stich in den ohren und den schmerzen
Hyoscyamus niger L.	Solanaceae	ren	LF	Bilsenkraut	top	EYE	CLXXVII / CCCXXIIII	hitzigen, trieffenden und schmerzlichen augen
Hyoscyamus niger L.	Solanaceae	ren	LF	Bilsenkraut	top	GYN	CLXXVII / CCCXXIIII	stellen das bluten und übrige fliessen der frawen
Hyoscyamus niger L.	Solanaceae	ren	LF	Bilsenkraut	syst	NER	CLXXVII / CCCXXIIII	bringt den schlaaf
Hyoscyamus niger L.	Solanaceae	ren	LF	Bilsenkraut	top	NER	CLXXVII / CCCXXIIII	bringt den schlaaf
Hyoscyamus niger L.	Solanaceae	ren	LF	Bilsenkraut	top	SKE	CLXXVII / CCCXXIIII	vertreiben sie die weetagen der glieder, das podagra
Hyoscyamus niger L.	Solanaceae	ren	LF	Bilsenkraut	top	TEE	CLXXVII / CCCXXIIII	stillt den grossen und unleidlichen schmerzen der zaen
Hyoscyamus niger L.	Solanaceae	s pop	VAL	Bilsenkraut	top	EAR	p. 324	inflammation des oreilles
Hyoscyamus niger L.	Solanaceae	s pop	VAL	Bilsenkraut	syst	GAS	p. 324	spasmes gastriques, oesophagiens, vésiculaires, intestinaux
Hyoscyamus niger L.	Solanaceae	s pop	VAL	Bilsenkraut	top	GYN	p. 324	mammite
Hyoscyamus niger L.	Solanaceae	s pop	VAL	Bilsenkraut	syst	NER	p. 324	tremblement des parkinsoniens, délire alcoolique, mélancolie anxieuse, agitation des maladies mentales
Hyoscyamus niger L.	Solanaceae	s pop	VAL	Bilsenkraut	top	SKE	p. 324	goutte, rhumatismes, contusions, entorses,
Hyoscyamus niger L.	Solanaceae	s pop	VAL	Bilsenkraut	top	TEE	p. 324	névralgies dentaires et autres
Hypericum perforatum agg.	Hypericaceae	ant	DIOS	Johanniskraut	top	DER	210	gutes Mittel als Umschlag bei Feuerbrandwunden
Hypericum perforatum agg.	Hypericaceae	ant	DIOS	Johanniskraut	syst	NER	210	wirksam bei Ischias . . . denn sie führt viel gallige Unreinigkeit ab
Hypericum perforatum agg.	Hypericaceae	mon	HvB	Johanniskraut	n.d.	n.d.	1-222	Für die Medizin jedoch taugt es nicht, weil es ein verwildertes und vernachlässigtes Kräutlein ist
Hypericum perforatum agg.	Hypericaceae	ren	LF	Johanniskraut	top	DER	CCLXXVI / CCCXXIII	faulen schaeden und geschwaer
Hypericum perforatum agg.	Hypericaceae	ren	LF	Johanniskraut	top	DER	CCLXXVI / CCCXXIII	heylet den brandt
Hypericum perforatum agg.	Hypericaceae	ren	LF	Johanniskraut	syst	FEV	CCLXXVI / CCCXXIII	vertreibt es das dreitägliche und viertägliche fieber
Hypericum perforatum agg.	Hypericaceae	ren	LF	Johanniskraut	syst	GYN	CCLXXVI / CCCXXIII	bringt den frawn ir zeit
Hypericum perforatum agg.	Hypericaceae	ren	LF	Johanniskraut	syst	SKE	CCLXXVI / CCCXXIII	heylet das hüftwee
Hypericum perforatum agg.	Hypericaceae	ren	LF	Johanniskraut	syst	URO	CCLXXVI / CCCXXIII	treibt den harn
Hypericum perforatum agg.	Hypericaceae	ren	LF	Johanniskraut	syst	URO	CCLXXVI / CCCXXIII	stellt den bauchfluss und ist trefflich gut zu den blasenstein
Hypericum perforatum agg.	Hypericaceae	s pop	BVA	Johanniskraut	syst	GAS	p. 111	Gallenstauung, Appetitlosigkeit
Hypericum perforatum agg.	Hypericaceae	s pop	BVA	Johanniskraut	syst	GAS	p. 111	Gallenstauung, Appetitlosigkeit
Hypericum perforatum agg.	Hypericaceae	s pop	BVA	Johanniskraut	syst	GYN	p. 111	Mensctruationsbeschwerden, Beschwerden bei der Menarche, Gebärmutterschmerzen
Hypericum perforatum agg.	Hypericaceae	s pop	BVA	Johanniskraut	syst	GYN	p. 111	Mensctruationsbeschwerden, Beschwerden bei der Menarche, Gebärmutterschmerzen
Hypericum perforatum agg.	Hypericaceae	s pop	BVA	Johanniskraut	syst	HUM	p. 111	Blutreinigung, Bluterfrischung
Hypericum perforatum agg.	Hypericaceae	s pop	BVA	Johanniskraut	syst	HUM	p. 111	Blutreinigung, Bluterfrischung
Hypericum perforatum agg.	Hypericaceae	s pop	BVA	Johanniskraut	syst	NER	p. 111	Nervosität, nervöser Unruhe, nervöser Erschöpfing, geistigerÜberanstrengung, Schlaflosigkeit, Neuralgie, Ischias, Hexenschuss

Hypericum perforatum agg.	Hypericaceae	s pop	BVA	Johanniskraut	syst	NER	p. 111	Nervosität, nervöser Unruhe, nervöser Erschöpfing, geistigerÜberanstrengung, Schlaflosigkeit, Neuralgie, Ischias, Hexenschuss
Hypericum perforatum agg.	Hypericaceae	s pop	BVA	Johanniskraut	syst	SKE	p. 111	Hexenschuss
Hypericum perforatum agg.	Hypericaceae	s pop	BVA	Johanniskraut	syst	SKE	p. 111	Hexenschuss
Hypericum perforatum agg.	Hypericaceae	s pop	BVA	Johanniskraut	top	SKE	p. 111	Blutergüsse, Quetschungen
Hypericum perforatum agg.	Hypericaceae	s pop	JK	Johanniskraut	top	DER	p. 379	Verbrennungen, Verbrühungen, Fettspritzer ins Auge, Wunden, Schürfungen, Insektenstiche
Hypericum perforatum agg.	Hypericaceae	s pop	JK	Johanniskraut	syst	GAS	p. 379	Gelbsucht
Hypericum perforatum agg.	Hypericaceae	s pop	JK	Johanniskraut	syst	RES	p. 379	Lungenentzündung, Brustfellentzündung, Grippe
Hypericum perforatum agg.	Hypericaceae	s pop	JK	Johanniskraut	syst	URO	p. 379	Niere, Blase, Bettnässen, Blasenkatarrh
Hypericum perforatum agg.	Hypericaceae	s pop	KN	Johanniskraut	syst	GAS	p. 475	Gasbildung, Verstopfung, Leberstauung
Hypericum perforatum agg.	Hypericaceae	s pop	KN	Johanniskraut	syst	GAS	p. 475	Kopfleiden, die von wässrigen Stoffen oder Verschleimung im Kopfe herrühren, leicht Verscleimung von Brust und Lunge
Hypericum perforatum agg.	Hypericaceae	s pop	KN	Johanniskraut	syst	URO	p. 475	Bettnässer
Hypericum perforatum agg.	Hypericaceae	s pop	MT	Johanniskraut	syst	GAS	p. 25	Durchfall
Hypericum perforatum agg.	Hypericaceae	s pop	MT	Johanniskraut	syst	GYN	p. 25	Unergelmässigkeiten der monatl. Periode
Hypericum perforatum agg.	Hypericaceae	s pop	MT	Johanniskraut	syst	NER	p. 25	Nervenverletzungen und nervösen Beschwerden
Hypericum perforatum agg.	Hypericaceae	s pop	MT	Johanniskraut	top	NER	p. 25	Nervenleiden, Nervenentzündungen usw.
Hypericum perforatum agg.	Hypericaceae	s pop	MT	Johanniskraut	syst	SKE	p. 25	Stossverletzungen Überheben
Hypericum perforatum agg.	Hypericaceae	s pop	UB	Johanniskraut	top	DER	p. 337	Neurodermitis
Hypericum perforatum agg.	Hypericaceae	s pop	UB	Johanniskraut	syst	GAS	p. 124	Durchfall
Hypericum perforatum agg.	Hypericaceae	s pop	UB	Johanniskraut	syst	NER	p. 428	depressive Verstimmung und Ängste
Hypericum perforatum agg.	Hypericaceae	s pop	UB	Johanniskraut	syst	NER	p. 428	depressive Verstimmung und Ängste
Hypericum perforatum agg.	Hypericaceae	s pop	UB	Johanniskraut	syst	NER	p. 428	depressive Verstimmung und Ängste
Hypericum perforatum agg.	Hypericaceae	s pop	UB	Johanniskraut	top	SKE	p. 290	degenerativen Rheumatischen Erkrankungen
Hypericum perforatum agg.	Hypericaceae	s pop	UB	Johanniskraut	top	SKE	p. 323	Verstauchungen, Blutergüssen, Quetschungen
Hypericum perforatum agg.	Hypericaceae	s pop	WI	Johanniskraut	syst	SKE	p. 310	Volksmedizin: Rheumatismus und Gicht
Hypericum perforatum agg.	Hypericaceae	sci	EMA	Johanniskraut	top	DER	HMPC/745582/2009	Traditional herbal medicinal product for the symptomatic treatment of minor inflammations of the skin (such as sunburn) and as an aid in healing of minor wounds.
Hypericum perforatum agg.	Hypericaceae	sci	EMA	Johanniskraut	top	DER	HMPC/745582/2009	Traditional herbal medicinal product for the symptomatic treatment of minor inflammations of the skin (such as sunburn) and as an aid in healing of minor wounds.
Hypericum perforatum agg.	Hypericaceae	sci	EMA	Johanniskraut	top	DER	HMPC/745582/2009	Traditional herbal medicinal product for the symptomatic treatment of minor inflammations of the skin (such as sunburn) and as an aid in healing of minor wounds.
Hypericum perforatum agg.	Hypericaceae	sci	EMA	Johanniskraut	syst	GAS	HMPC/745582/2009	Traditional herbal medicinal product for the symptomatic relief of mild gastrointestinal discomfort.
Hypericum perforatum agg.	Hypericaceae	sci	EMA	Johanniskraut	syst	NER	HMPC/101304/2008	Well-established-use:Herbal medicinal product for the treatment of mild to moderate depressive episodes (according to ICD-10).
Hypericum perforatum agg.	Hypericaceae	sci	EMA	Johanniskraut	syst	NER	HMPC/745582/2009	Traditional herbal medicinal product for the relief of temporary mental exhaustion.
Hypericum perforatum agg.	Hypericaceae	sci	EMA	Johanniskraut	syst	NER	HMPC/745582/2009	Traditional herbal medicinal product for the relief of temporary mental exhaustion.
Hypericum perforatum agg.	Hypericaceae	sci	EMA	Johanniskraut	syst	NER	HMPC/745582/2009	Traditional herbal medicinal product for the relief of temporary mental exhaustion.
Hypericum perforatum agg.	Hypericaceae	sci	EMA	Johanniskraut	syst	NER	HMPC/745582/2009	Traditional herbal medicinal product for the relief of temporary mental exhaustion.
Hypericum perforatum agg.	Hypericaceae	sci	EMA	Johanniskraut	syst	NER	HMPC/745582/2009	Traditional herbal medicinal product for the relief of temporary mental exhaustion.
Hypericum perforatum agg.	Hypericaceae	sci	WI	Johanniskraut	syst	NER	p. 310	leichte Formen von Depressionen
Inula helenium L.	Asteraceae	ant	DIOS	Alant	syst	ANT	77	gegen den Biss giftiger Tiere
Inula helenium L.	Asteraceae	ant	DIOS	Alant	syst	GAS	77	Krämpfe, Blähungen
Inula helenium L.	Asteraceae	ant	DIOS	Alant	syst	GYN	77	treibt[den Urin und] die Menstruation
Inula helenium L.	Asteraceae	ant	DIOS	Alant	syst	n.d.	77	innere Rupturen,
Inula helenium L.	Asteraceae	ant	DIOS	Alant	top	NER	77	Ischias Leidenden aufgelegt
Inula helenium L.	Asteraceae	ant	DIOS	Alant	syst	OTH	77	Zerstoßen und genossen hilft sie denen, die an Blutsturz leiden
Inula helenium L.	Asteraceae	ant	DIOS	Alant	syst	URO	77	treibt den Urin [und die Menstruation]
Inula helenium L.	Asteraceae	mon	HvB	Alant	syst	EYE	1-95	reinigt die Augen
Inula helenium L.	Asteraceae	mon	HvB	Alant	syst	NER	1-95	unterdrückt Migräne
Inula helenium L.	Asteraceae	mon	HvB	Alant	syst	RES	1-95	Schmerzen in der Lunge
Inula helenium L.	Asteraceae	mon	MF	Alant	syst	GAS	44	verstopften Bauch erweichen
Inula helenium L.	Asteraceae	mon	MF	Alant	syst	GYN	44	sorgt für geordneten Moonatsfluss, wirkt abtreibend
Inula helenium L.	Asteraceae	mon	MF	Alant	syst	RES	44	beschwichtigt den Husten und heilt Atemnot
Inula helenium L.	Asteraceae	mon	MF	Alant	syst	SKE	44	Leistenbruch zuverlässig von Nutzen sein
Inula helenium L.	Asteraceae	mon	MF	Alant	top	SKE	44	vertreibt Hüftgicht
Inula helenium L.	Asteraceae	mon	MF	Alant	syst	URO	44	wirkt harntreibend
Inula helenium L.	Asteraceae	mon	MF	Alant	top	URO	44	nützt den Nierenleidenden
Inula helenium L.	Asteraceae	ren	LF	Alant	syst	ANT	CXXXIIII / LXXXIX	für das aufflaeen von biss der gifftigen thier
Inula helenium L.	Asteraceae	ren	LF	Alant	syst	GAS	CXXXIIII / LXXXIX	ist dem magen nützlich
Inula helenium L.	Asteraceae	ren	LF	Alant	syst	GYN	CXXXIIII / LXXXIX	bringt den frawen ir bloedigkeit
Inula helenium L.	Asteraceae	ren	LF	Alant	syst	RES	CXXXIIII / LXXXIX	dienstlich denen so husten und keichen, inwendig gebrochen sind und blut speien
Inula helenium L.	Asteraceae	ren	LF	Alant	top	SKE	CXXXIIII / LXXXIX	lindert das hüftwee
Inula helenium L.	Asteraceae	ren	LF	Alant	top	SKE	CXXXIIII / LXXXIX	verzert es die geschwulst so sch von kalter feuchtigkeyt erhebt haben
Inula helenium L.	Asteraceae	ren	LF	Alant	syst	URO	CXXXIIII / LXXXIX	treibt den harn
Inula helenium L.	Asteraceae	s pop	JK	Alant	syst	RES	p. 322	Katarrh, Lungenverschleimung, Astmah
Inula helenium L.	Asteraceae	s pop	WI	Alant	top	DER	p. 285	Volksmedizin: Exanthemen und einfektiösen Hauterkrankungen
Inula helenium L.	Asteraceae	s pop	WI	Alant	syst	GAS	p. 285	Volksmedizin: Stomachikum, Karminativum, Cholagogum
Inula helenium L.	Asteraceae	s pop	WI	Alant	syst	GAS	p. 285	Volksmedizin: Anthelmintikum
Inula helenium L.	Asteraceae	s pop	WI	Alant	syst	URO	p. 285	Volksmedizin: Infektionen der ableitenden Harnwege
Inula helenium L.	Asteraceae	sci	WI	Alant	syst	RES	p. 285	Exepktorans
Iris germanica agg.	Iridaceae	ant	DIOS	Schwertlilie	syst	ANT	1/68	benehmen einem verderbenbringenden Mittel die Kraft, wenn sie mit Honigmeth genossen werden
Iris germanica agg.	Iridaceae	ant	DIOS	Schwertlilie	syst	ANT	1/68	Mit Essig genommen sind sie denen heilsam, die von giftigen Tieren gebissen sind
Iris germanica agg.	Iridaceae	ant	DIOS	Schwertlilie	top	DER	1/68	mit doppelt so viel weißer Nieswurz aufgelegt, Sonnenbrandflecken und Leberflecken. Sie füllen die
Iris germanica agg.	Iridaceae	ant	DIOS	Schwertlilie	top	DER	1/68	Sie erweichen auch Drüsen und alte Verhärtungen, wenn sie gekocht umgeschlagen worden
Iris germanica agg.	Iridaceae	ant	DIOS	Schwertlilie	syst	FEV	1/68	die von Kälte und Frostschauern ergriffen sind
Iris germanica agg.	Iridaceae	ant	DIOS	Schwertlilie	syst	GYN	1/68	Mit Wein genommen, befördern sie die Katamenien,
Iris germanica agg.	Iridaceae	ant	DIOS	Schwertlilie	syst	GYN	1/68	auch ist die Abkochung davon zu Blähungen für Frauen sehr geeignet, indem sie die Stellen erweicht und öffnet
Iris germanica agg.	Iridaceae	ant	DIOS	Schwertlilie	syst	GYN	1/68	Mit Honig als Paste eingeführt, ziehen sie den Embryo heraus
Iris germanica agg.	Iridaceae	ant	DIOS	Schwertlilie	top	n.d.	1/68	Endlich werden sie auch den Zäpfchen, Pflastern und Salben zugemischt und sind zu Vielem nützlich
Iris germanica agg.	Iridaceae	ant	DIOS	Schwertlilie	n.d.	n.d.	1/68	verursachen Tränen und heilen Leibschneiden
Iris germanica agg.	Iridaceae	ant	DIOS	Schwertlilie	n.d.	NER	1/68	Sie sind aber auch schlafmachend
Iris germanica agg.	Iridaceae	ant	DIOS	Schwertlilie	syst	NER	1/68	denen, die an Krämpfen leiden
Iris germanica agg.	Iridaceae	ant	DIOS	Schwertlilie	top	NER	1/68	Ferner sind sie wohltuend bei Kopfschmerzen wenn sie mit Essig und
Iris germanica agg.	Iridaceae	ant	DIOS	Schwertlilie	syst	OTH	1/68	Milzsüchtigen
Iris germanica agg.	Iridaceae	ant	DIOS	Schwertlilie	n.d.	RES	1/68	verdünnen die schwer auszuwerfenden Flüssigkeiten
Iris germanica agg.	Iridaceae	ant	DIOS	Schwertlilie	syst	URO	1/68	denen, die an Samenfluss leiden
Iris germanica agg.	Iridaceae	mon	HvB	Schwertlilie	top	DER	1-118	Und jenen, der die kleine Krätze hat, den salbe ot mit dieser Salbe
Iris germanica agg.	Iridaceae	mon	HvB	Schwertlilie	top	DER	1-118	Und wer im Gesicht harte Haut hat wie Rinde, oder wer dort beulig ist . . . .
Iris germanica agg.	Iridaceae	mon	HvB	Schwertlilie	top	DER	1-118	und gegen frische Lepra
Iris germanica agg.	Iridaceae	mon	HvB	Schwertlilie	syst	GAS	1-118	und gib es warm so jenem zu trinken, der einen Stein hat
Iris germanica agg.	Iridaceae	mon	HvB	Schwertlilie	syst	NER	1-118	und gib sie diesem Verrückten, das heisst Hirnwütigen oft zu essen

Iris germanica agg.	Iridaceae	mon	HvB	Schwertlilie	top	NER	1-118	umwinden den Kopf eines Verrückten und dessen der hinrwütig ist so warm, und binde ein Tuch darüber, damit er schläft
Iris germanica agg.	Iridaceae	mon	HvB	Schwertlilie	syst	URO	1-118	und wer von Schwierigkeiten des Harnlassens zusammengeschnürt wird, in dem erweicht er den Stein
Iris germanica agg.	Iridaceae	mon	MF	Schwertlilie	syst	ANT	43	falls einer Gift eingenommen hat, solches entschärfen
Iris germanica agg.	Iridaceae	mon	MF	Schwertlilie	top	DER	43	Fisteln heilt
Iris germanica agg.	Iridaceae	mon	MF	Schwertlilie	top	DER	43	trocknet nässende Wunden, lässt Knochen wieder wachsen
Iris germanica agg.	Iridaceae	mon	MF	Schwertlilie	top	DER	43	reinigt das Angesicht vom Leberfleck und vertreibt auch den Grind
Iris germanica agg.	Iridaceae	mon	MF	Schwertlilie	syst	GAS	43	stillt das Bauchgrimmen
Iris germanica agg.	Iridaceae	mon	MF	Schwertlilie	syst	GYN	43	sorgt für geordneten Monatsfluss
Iris germanica agg.	Iridaceae	mon	MF	Schwertlilie	top	GYN	43	schnell die Nachgeburt austreibt
Iris germanica agg.	Iridaceae	mon	MF	Schwertlilie	top	GYN	43	erweicht Verährtungen in der Gebärmutter
Iris germanica agg.	Iridaceae	mon	MF	Schwertlilie	syst	HUM	43	wenn Eiter in der Herzgegend versteckt, Herz und Brust schädigt, . . . Und treibt hiermit den Eiter aus
Iris germanica agg.	Iridaceae	mon	MF	Schwertlilie	syst	NER	43	führen Schlummer herbei
Iris germanica agg.	Iridaceae	mon	MF	Schwertlilie	syst	OTH	43	Milzsüchtige
Iris germanica agg.	Iridaceae	mon	MF	Schwertlilie	syst	RES	43	stillen den Husten
Iris germanica agg.	Iridaceae	mon	MF	Schwertlilie	syst	SKE	43	verkrüppelten oder durch Kälte verkrampften Gliedern helfen
Iris germanica agg.	Iridaceae	mon	MF	Schwertlilie	top	SKE	43	Hüftgichtschmerz gut tun
Iris germanica agg.	Iridaceae	ren	LF	Schwertlilie	syst	ANT	CLXXVIII / CXIX	denen so von gifftigen thieren gebissen sind
Iris germanica agg.	Iridaceae	ren	LF	Schwertlilie	top	DER	CLXXVIII / CXIX	reynigen sie die unreynen wunden, heylen die fisteln
Iris germanica agg.	Iridaceae	ren	LF	Schwertlilie	top	DER	CLXXVIII / CXIX	runzel und klunse (?) oder spalt des hindern
Iris germanica agg.	Iridaceae	ren	LF	Schwertlilie	syst	FEV	CLXXVIII / CXIX	denen so erkaltet seind unnd des frosts warten im feber
Iris germanica agg.	Iridaceae	ren	LF	Schwertlilie	syst	GAS	CLXXVIII / CXIX	stillen das bauchgrimmen
Iris germanica agg.	Iridaceae	ren	LF	Schwertlilie	syst	GYN	CLXXVIII / CXIX	bringen den frawen ihre zeyt
Iris germanica agg.	Iridaceae	ren	LF	Schwertlilie	top	GYN	CLXXVIII / CXIX	baeung der verherten muter
Iris germanica agg.	Iridaceae	ren	LF	Schwertlilie	syst	NER	CLXXVIII / CXIX	bringen ruw und machen schlaffen
Iris germanica agg.	Iridaceae	ren	LF	Schwertlilie	top	NER	CLXXVIII / CXIX	lindert sie den schmerzen des haupts
Iris germanica agg.	Iridaceae	ren	LF	Schwertlilie	syst	OTH	CLXXVIII / CXIX	milzsüchtigen
Iris germanica agg.	Iridaceae	ren	LF	Schwertlilie	syst	RES	CLXXVIII / CXIX	seind gut zu dem husten . . .
Iris germanica agg.	Iridaceae	ren	LF	Schwertlilie	top	RES	CLXXVIII / CXIX	macht sie guten atem
Iris germanica agg.	Iridaceae	ren	LF	Schwertlilie	top	SKE	CLXXVIII / CXIX	übergelegt, zeucht si heraus die gebrochnen bei
Iris germanica agg.	Iridaceae	ren	LF	Schwertlilie	top	SKE	CLXXVIII / CXIX	clystier denen so das hüfftwee haben, mit harz vermengt . . . Legt sie den schmerzen der flachsadern (?), lenden und hüfften
Iris germanica agg.	Iridaceae	ren	LF	Schwertlilie	top	TEE	CLXXVIII / CXIX	die zan weetagen haben
Iris germanica agg.	Iridaceae	ren	LF	Schwertlilie	syst	URO	CLXXVIII / CXIX	treibt aus die wassersucht, den lendenstein und den harn
Iris germanica agg.	Iridaceae	ren	LF	Schwertlilie	syst	URO	CLXXVIII / CXIX	denen so den samen nit künden behalten
Iris germanica agg.	Iridaceae	s pop	WI	Schwertlilie	syst	RES	p. 317	Volksmedizin: Expektorans und Muzilaginosum
Juglans regia L.	Juglandaceae	ant	DIOS	Walnuss	syst	ANT	79	Gegenmittel für tödliche Gifte, wenn sie vorher oder nachher mit Feigen und Raute
Juglans regia L.	Juglandaceae	ant	DIOS	Walnuss	top	ANT	79	Mit Zwiebeln, Salz und Honig wirken sie beim Biss des Hundes und Menschen
Juglans regia L.	Juglandaceae	ant	DIOS	Walnuss	top	DER	79	hat die Kraft, Male, Leberflecken, Finnen und das Dunkle der Narben zu vertreiben
Juglans regia L.	Juglandaceae	ant	DIOS	Walnuss	top	DER	79	heilen Gangrän, Karbunkeln, Gaisaugen und Fuchskrankheit; Als Umschlag beseitigen sie auch blutunterlaufene Stellen
Juglans regia L.	Juglandaceae	ant	DIOS	Walnuss	top	DER	79	als Um- schlag auf entzündete (schwärende) Brüste, auf Ablagerungen (Abszesse)
Juglans regia L.	Juglandaceae	ant	DIOS	Walnuss	top	DER	79	Wein und Öl zerriebene Schale bewirkt als Pomade bei Kindern schönes Haar und stärkt das nach der Fuchskrankheit ausfallende.
Juglans regia L.	Juglandaceae	ant	DIOS	Walnuss	top	EAR	79	hilft es gegen Ohrenschmerzen, Ohrensausen und Ohrenklingen.
Juglans regia L.	Juglandaceae	ant	DIOS	Walnuss	syst	GAS	79	Reichlich genossen treiben sie den Bandwurm aus
Juglans regia L.	Juglandaceae	ant	DIOS	Walnuss	top	GAS	79	auf den Nabel gelegt, lindern sie Leibschneiden
Juglans regia L.	Juglandaceae	ant	DIOS	Walnuss	top	GYN	79	Auch die Menstruation stellt der Kern
Juglans regia L.	Juglandaceae	ant	DIOS	Walnuss	syst	HUM	79	auch den Bauch zu reinigen. Dem Magen ist es nicht zuträglich
Juglans regia L.	Juglandaceae	ant	DIOS	Walnuss	syst	HUM	79	sind als Speise dazu dienlich, um bei dem Nüchternen Brechen zu erregen
Juglans regia L.	Juglandaceae	ant	DIOS	Walnuss	syst	OTH	79	sind genossen schwer zu verdauen, dem Magen schädlich, sie machen Galle, Kopfschmerzen und sind denen, die an Husten leiden, schädlich
Juglans regia L.	Juglandaceae	ant	DIOS	Walnuss	top	SKE	79	und Verrenkungen gelegt
Juglans regia L.	Juglandaceae	mon	HvB	Walnuss	top	DER	3-4	wo die Würmer den Menschen plagen, oder wo Maden oder andere Würmer in ihm wachsen [. . .] Lepra . . . Salbe
Juglans regia L.	Juglandaceae	mon	HvB	Walnuss	top	DER	3-4	Kopfgrind
Juglans regia L.	Juglandaceae	mon	HvB	Walnuss	syst	GAS	3-4	Würmer in deinem Magen
Juglans regia L.	Juglandaceae	mon	HvB	Walnuss	syst	HUM	3-4	wer viel Schleim in sich hat
Juglans regia L.	Juglandaceae	mon	HvB	Walnuss	top	SKE	3-4	Gicht, nehme die Erde, die um die Wurzel eines Baumes liegt
Juglans regia L.	Juglandaceae	ren	LF	Walnuss	syst	ANT	CCXIII / CXLII	welcher speien will, widerstreben sie allem tödlichen gifft
Juglans regia L.	Juglandaceae	ren	LF	Walnuss	top	ANT	CCXIII / CXLII	heylen die biss eines wütenden hundes
Juglans regia L.	Juglandaceae	ren	LF	Walnuss	top	DER	CCXIII / CXLII	macht schoen har
Juglans regia L.	Juglandaceae	ren	LF	Walnuss	top	DER	CCXIII / CXLII	vertreiben die blawen maeler am leib
Juglans regia L.	Juglandaceae	ren	LF	Walnuss	top	DER	CCXIII / CXLII	guten gegen geschwaeren der brust und verzuckten glieder
Juglans regia L.	Juglandaceae	ren	LF	Walnuss	top	DER	CCXIII / CXLII	gut zu menschen oder hundebiss
Juglans regia L.	Juglandaceae	ren	LF	Walnuss	top	DER	CCXIII / CXLII	heylt die geschwaere des munds
Juglans regia L.	Juglandaceae	ren	LF	Walnuss	top	GAS	CCXIII / CXLII	stillen das bauchgrimmen
Juglans regia L.	Juglandaceae	ren	LF	Walnuss	syst	GAS	CCXIII / CXLII	treiben sie die breyten würm aus
Juglans regia L.	Juglandaceae	ren	LF	Walnuss	syst	GYN	CCXIII / CXLII	stellen den frawen ihre krankheyt
Juglans regia L.	Juglandaceae	s pop	JK	Walnuss	top	DER	p. 433	heilt Wunden und bring tWarzen zum Verschwinden
Juglans regia L.	Juglandaceae	s pop	JK	Walnuss	syst	DER	p. 433	Ausschlag,
Juglans regia L.	Juglandaceae	s pop	JK	Walnuss	syst	DER	p. 433	Bleichsucht und Skrofulose
Juglans regia L.	Juglandaceae	s pop	JK	Walnuss	top	DER	p. 433	Leiden im Hals, Mandeln, Kelhkopf; Frostbeulen, Krätze
Juglans regia L.	Juglandaceae	s pop	JK	Walnuss	top	DER	p. 433	Kopfwaschmittel bei Haarausfall,
Juglans regia L.	Juglandaceae	s pop	JK	Walnuss	top	EYE	p. 433	Augen
Juglans regia L.	Juglandaceae	s pop	JK	Walnuss	syst	GAS	p. 433	Magenstärkend, stillt den Durchfall
Juglans regia L.	Juglandaceae	s pop	JK	Walnuss	syst	GAS	p. 433	Zuckerkrankheit und Gelbsucht
Juglans regia L.	Juglandaceae	s pop	JK	Walnuss	syst	n.d.	p. 433	Greisenbrand
Juglans regia L.	Juglandaceae	s pop	JK	Walnuss	syst	SKE	p. 433	Rheumatismus
Juglans regia L.	Juglandaceae	s pop	KN	Walnuss	top	DER	p. 413	hauptsächlich Kinder, Skrofulose, Lymphytismus
Juglans regia L.	Juglandaceae	s pop	UB	Walnuss	syst	DER	p. 331	Ekzeme, Neurodermitis und Psoriasis
Juglans regia L.	Juglandaceae	s pop	UB	Walnuss	top	DER	p. 331	Ekzeme, Neurodermitis und Psoriasis
Juglans regia L.	Juglandaceae	s pop	UB	Walnuss	syst	GAS	p. 461	Heilpflanzen mit Gerbstoffen zum Binden, Ausleitung und Regeneration
Juglans regia L.	Juglandaceae	s pop	WI	Walnuss	syst	GAS	p. 320	Volksmedizin: Magen-Damrkatarrhen, Anthelmintikum
Juglans regia L.	Juglandaceae	s pop	WI	Walnuss	syst	URO	p. 320	Volksmedizin: Blutreinigungsmittel
Juglans regia L.	Juglandaceae	sci	EMA	Walnuss	top	DER	/HPMC/34737/2011	minor inflamatory conditions of the skin
Juglans regia L.	Juglandaceae	sci	EMA	Walnuss	top	DER	/HPMC/34737/2011	used in exessive perspiration of hands and feet
Juglans regia L.	Juglandaceae	sci	WI	Walnuss	top	DER	p. 320	Hautleiden
Juniperus communis agg.	Cupressaceae	ant	DIOS	Wacholder	syst	ANT	95	gegen den Biss wilder Tiere
Juniperus communis agg.	Cupressaceae	ant	DIOS	Wacholder	syst	GAS	95	Blähungen , Leibschneiden
Juniperus communis agg.	Cupressaceae	ant	DIOS	Wacholder	syst	GYN	95	bei Mutterkrämpfen
Juniperus communis agg.	Cupressaceae	ant	DIOS	Wacholder	syst	n.d.	95	inneren Zerreißungen
Juniperus communis agg.	Cupressaceae	ant	DIOS	Wacholder	syst	NER	95	auch bei Krämpfen
Juniperus communis agg.	Cupressaceae	ant	DIOS	Wacholder	vol	OTH	95	vertreiben sie die wilden Tiere.
Juniperus communis agg.	Cupressaceae	ant	DIOS	Wacholder	syst	RES	95	Getrunken wirkt sie bei Brustleiden, Husten,
Juniperus communis agg.	Cupressaceae	ant	DIOS	Wacholder	syst	URO	95	auch unrintreibend
Juniperus communis agg.	Cupressaceae	mon	HvB	Wacholder	top	FEV	3-43	verschiedene und üble Fieber
Juniperus communis agg.	Cupressaceae	mon	HvB	Wacholder	syst	GAS	3-43	Schmerzen in der Leber
Juniperus communis agg.	Cupressaceae	mon	HvB	Wacholder	syst	RES	3-43	Schmerzen in der Brust oder Lunge
Juniperus communis agg.	Cupressaceae	ren	LF	Wacholder	vol	APO	XLIII / XXVI	vertreibt die schlangen und den vergifften luft
Juniperus communis agg.	Cupressaceae	ren	LF	Wacholder	top	DER	XLIII / XXVI	vertreibet die raeuden
Juniperus communis agg.	Cupressaceae	ren	LF	Wacholder	n.d.	DER	XLIII / XXVI	heylet und trücknet aus die fisteln, hände und füss zerschunden sind
Juniperus communis agg.	Cupressaceae	ren	LF	Wacholder	top	DER	XLIII / XXVI	stirn gestrichen verstelt das bluten der nasen
Juniperus communis agg.	Cupressaceae	ren	LF	Wacholder	syst	GAS	XLIII / XXVI	stercken den magen, vertreiben das bauchblehen

Juniperus communis agg.	Cupressaceae	ren	LF	Wacholder	syst	GAS	XLIII / XXVI	verstelt es auch das würgen und die rote rhur
Juniperus communis agg.	Cupressaceae	ren	LF	Wacholder	top	GAS	XLIII / XXVI	verstelt es auch das würgen und speyen, den bauchfluss
Juniperus communis agg.	Cupressaceae	ren	LF	Wacholder	n.d.	GAS	XLIII / XXVI	toedt die würm im leib
Juniperus communis agg.	Cupressaceae	ren	LF	Wacholder	syst	GYN	XLIII / XXVI	gut den weibern, denen die muter über sich steigt
Juniperus communis agg.	Cupressaceae	ren	LF	Wacholder	n.d.	GYN	XLIII / XXVI	stellet den weibern ihr krankheyt
Juniperus communis agg.	Cupressaceae	ren	LF	Wacholder	syst	RES	XLIII / XXVI	vertreiben den husten
Juniperus communis agg.	Cupressaceae	ren	LF	Wacholder	vol	RES	XLIII / XXVI	stellet die schnupffen
Juniperus communis agg.	Cupressaceae	ren	LF	Wacholder	top	SKE	XLIII / XXVI	denen den krampff haben und das hüftwee
Juniperus communis agg.	Cupressaceae	ren	LF	Wacholder	syst	URO	XLIII / XXVI	treiben den harn
Juniperus communis agg.	Cupressaceae	s pop	BVA	Wacholder	top	DER	p. 239	Flechten, Hautausschläge
Juniperus communis agg.	Cupressaceae	s pop	BVA	Wacholder	syst	GAS	p. 239	Stärkung von Magen und Darm
Juniperus communis agg.	Cupressaceae	s pop	BVA	Wacholder	syst	HUM	p. 239	Förderung des Stoffwechsels
Juniperus communis agg.	Cupressaceae	s pop	BVA	Wacholder	syst	SKE	p. 239	Ausscheidung von harnsauren Salzen bei Rheuma und Gicht
Juniperus communis agg.	Cupressaceae	s pop	BVA	Wacholder	top	SKE	p. 239	Einreiben bei Rheuma
Juniperus communis agg.	Cupressaceae	s pop	BVA	Wacholder	syst	TON	p. 239	Steigerung der Widerstandskraft des Körpers
Juniperus communis agg.	Cupressaceae	s pop	BVA	Wacholder	syst	URO	p. 239	vermehrte Harnausscheidung bei Wassersucht, Ödemen, Schwellungen und bei Abmagerungskuren
Juniperus communis agg.	Cupressaceae	s pop	JK	Wacholder	syst	GAS	p. 431	Magenschwache
Juniperus communis agg.	Cupressaceae	s pop	JK	Wacholder	syst	GAS	p. 431	reinigt Magen und Gedärme
Juniperus communis agg.	Cupressaceae	s pop	JK	Wacholder	vol	RES	p. 431	Hals- und Brustleiden, Lungenkatarrh, Keuchhusten
Juniperus communis agg.	Cupressaceae	s pop	JK	Wacholder	top	SKE	p. 431	Gicht und Rheumatismus
Juniperus communis agg.	Cupressaceae	s pop	JK	Wacholder	syst	URO	p. 431	reinigt Niere und Blase
Juniperus communis agg.	Cupressaceae	s pop	JK	Wacholder	syst	URO	p. 431	Blutreinigungsmittel, Wasserabsonderung
Juniperus communis agg.	Cupressaceae	s pop	KN	Wacholder	vol	APO	p. 488	als Räucherwerk verbreitet sie in Zimmern und Gängen einen angenehmen Geruch
Juniperus communis agg.	Cupressaceae	s pop	KN	Wacholder	syst	GAS	p. 488	schwacher Magen (Kur)
Juniperus communis agg.	Cupressaceae	s pop	KN	Wacholder	vol	RES	p. 488	feuchter Bronchitis, Wirkung auf die Lunge
Juniperus communis agg.	Cupressaceae	s pop	KN	Wacholder	top	SKE	p. 488	Gelenkleiden und tiefliegenden Entzündungen
Juniperus communis agg.	Cupressaceae	s pop	KN	Wacholder	syst	URO	p. 488	harntreibend
Juniperus communis agg.	Cupressaceae	s pop	KN	Wacholder	syst	URO	p. 488	beginnender Wassersucht, Nierenausscheidungen
Juniperus communis agg.	Cupressaceae	s pop	UB	Wacholder	top	SKE	p. 293	degenerative rheumatische Erkankungen
Juniperus communis agg.	Cupressaceae	s pop	UB	Wacholder	syst	URO	p. 257	Durchspülung
Juniperus communis agg.	Cupressaceae	s pop	WI	Wacholder	syst	DER	p. 323	Volksmedizin: schlecht heilende Wunden
Juniperus communis agg.	Cupressaceae	s pop	WI	Wacholder	syst	SKE	p. 323	Volksmedizin: Gicht und Rheuma
Juniperus communis agg.	Cupressaceae	sci	EMA	Wacholder	syst	GAS	HMPC/441929/2008	for symptomatic relief of digestive disorders such as dyspepsia and flatulence
Juniperus communis agg.	Cupressaceae	sci	EMA	Wacholder	syst	GAS	HMPC/441929/2008	for symptomatic relief of digestive disorders such as dyspepsia and flatulence
Juniperus communis agg.	Cupressaceae	sci	EMA	Wacholder	top	SKE	/HMPC/12402/2010	as adjuvant in the relief of minor muscular and articular pain
Juniperus communis agg.	Cupressaceae	sci	EMA	Wacholder	syst	URO	/HMPC/12402/2010	to increase the amount of urine to achieve flushing of the urinary tract as an adjuvant in minor urinary tract complaints
Juniperus communis agg.	Cupressaceae	sci	EMA	Wacholder	syst	URO	HMPC/441929/2008	to increase the amount of urine to achieve flushing of the urinary tract as an adjuvant in minor urinary tract complaints
Juniperus communis agg.	Cupressaceae	sci	EMA	Wacholder	syst	URO	HMPC/441929/2008	to increase the amount of urine to achieve flushing of the urinary tract as an adjuvant in minor urinary tract complaints
Juniperus communis agg.	Cupressaceae	sci	EMA	Wacholder	syst	URO	HMPC/441929/2008	to increase the amount of urine to achieve flushing of the urinary tract as an adjuvant in minor urinary tract complaints
Juniperus sabina L.	Cupressaceae	ant	DIOS	Sadebaum	syst	GYN	96	treiben den Fötus aus;
Juniperus sabina L.	Cupressaceae	ant	DIOS	Sadebaum	syst	GYN	96	treiben den Fötus aus;
Juniperus sabina L.	Cupressaceae	ant	DIOS	Sadebaum	vol	GYN	96	treiben den Fötus aus;
Juniperus sabina L.	Cupressaceae	ant	DIOS	Sadebaum	top	n.d.	96	Blätter beider hemmen um sich fressende Geschwüre und lindern im Umschlag Entzündungen, reinigen, mit Honig aufgeschmiert, von schwarzen Massen und Schmutz und reißen ringsum die Karbunkeln auf
Juniperus sabina L.	Cupressaceae	ant	DIOS	Sadebaum	top	n.d.	96	erwärmenden Salbölen
Juniperus sabina L.	Cupressaceae	ant	DIOS	Sadebaum	vol	n.d.	96	Einige wenden die Blätter zum Räuchern an
Juniperus sabina L.	Cupressaceae	ant	DIOS	Sadebaum	syst	URO	96	führen sie auch das Blut durch den Urin ab
Juniperus sabina L.	Cupressaceae	ant	DIOS	Sadebaum	syst	URO	96	führen sie auch das Blut durch den Urin ab
Juniperus sabina L.	Cupressaceae	ant	DIOS	Sadebaum	vol	URO	96	führen sie auch das Blut durch den Urin ab
Juniperus sabina L.	Cupressaceae	mon	HvB	Sadebaum	top	DER	3-21	Geschwüre, wo immer Würmer einen Menschen in seinem Körper annagen
Juniperus sabina L.	Cupressaceae	mon	HvB	Sadebaum	syst	RES	3-21	Schmerzen in der Lunge, so dass seine Lunge giftig ist und faul
Juniperus sabina L.	Cupressaceae	mon	MF	Sabina	top	DER	12	heilt Wunden und fressende Geschwüre, reinigt und beseitigt auch den Eiter
Juniperus sabina L.	Cupressaceae	mon	MF	Sabina	top	DER	12	verleiht der Haut Glanz
Juniperus sabina L.	Cupressaceae	mon	MF	Sabina	top	DER	12	Milzbrand (gute Bläschen)
Juniperus sabina L.	Cupressaceae	mon	MF	Sabina	syst	GYN	12	geordneter Monatsfluss
Juniperus sabina L.	Cupressaceae	mon	MF	Sabina	top	GYN	12	von unten an Muttermund geführt, zieht sie eine abgestorbene Leibesfrucht aus der Gebärmutter
Juniperus sabina L.	Cupressaceae	mon	MF	Sabina	top	NER	12	hilft Menschen, die an Schwindel leiden
Juniperus sabina L.	Cupressaceae	ren	LF	Sevistrauch	top	DER	LXXXII / LIIII	geschwaer, die um sich fressen
Juniperus sabina L.	Cupressaceae	ren	LF	Sevistrauch	syst	GAS	LXXXII / LIIII	vertreiben sie die geelsucht
Juniperus sabina L.	Cupressaceae	ren	LF	Sevistrauch	syst	GYN	LXXXII / LIIII	treiben tote frucht aus muter leib
Juniperus sabina L.	Cupressaceae	ren	LF	Sevistrauch	top	GYN	LXXXII / LIIII	bringen den frawen ihr bloedigkeit
Juniperus sabina L.	Cupressaceae	s pop	VAL	Sadebaum	top	DER	p. 453	plaies putrides (ulcères putrides ou gangréneux)
Juniperus sabina L.	Cupressaceae	s pop	VAL	Sadebaum	syst	GYN	p. 453	métrorragies
Lepidium spp. (*Lepidium off., Nasturtiun*	Brassicaceae	ant	DIOS	Gartenkresse	top	DER	160	auch den Aussatz nimmt es weg
Lepidium spp. (*Lepidium off., Nasturtiun*	Brassicaceae	ant	DIOS	Brunnenkresse	top	DER	147	vertreibt Leber- und Sonnenbrandflecken
Lepidium spp. (*Lepidium off., Nasturtiun*	Brassicaceae	ant	DIOS	Gartenkresse	top	OTH	160	Milzschmerzen
Lepidium spp. (*Lepidium off., Nasturtiun*	Brassicaceae	ant	DIOS	Gartenkresse	top	SKE	160	als Umschlag ein sehr hilfreiches Mittel gegen Ischias
Lepidium spp. (*Lepidium off., Nasturtiun*	Brassicaceae	ant	DIOS	Gartenkresse	top	TEE	160	scheint auch Zahnschmerzen zu lindern
Lepidium spp. (*Lepidium off., Nasturtiun*	Brassicaceae	ant	DIOS	Brunnenkresse	syst	URO	147	treibt den Harn
Lepidium spp. (*Lepidium off., Nasturtiun*	Brassicaceae	mon	HvB	Brunnenkresse	syst	GAS	1-73	gegessene Speisen kaum verdauen
Lepidium spp. (*Lepidium off., Nasturtiun*	Brassicaceae	mon	HvB	Brunnenkresse	syst	GAS	1-73	Gelbsucht
Lepidium spp. (*Lepidium off., Nasturtiun*	Brassicaceae	mon	LO	Gartenkresse	n.d.	OTH	n.d.	n.d.
Lepidium spp. (*Lepidium off., Nasturtiun*	Brassicaceae	mon	MF	Brunnenkresse	syst	ANT	30	soll sich den Schlangengiften widersetzen
Lepidium spp. (*Lepidium off., Nasturtiun*	Brassicaceae	mon	MF	Brunnenkresse	syst	APH	30	zähmt die Liebeskraft
Lepidium spp. (*Lepidium off., Nasturtiun*	Brassicaceae	mon	MF	Brunnenkresse	top	DER	30	stösst ein Furunkel seinen Eiter aus, treibt die Krätze fort, Jucken und scheussliche Schwären dein Haupt verunzieren
Lepidium spp. (*Lepidium off., Nasturtiun*	Brassicaceae	mon	MF	Brunnenkresse	top	DER	30	soll die ausfallenden Haare zurück halten
Lepidium spp. (*Lepidium off., Nasturtiun*	Brassicaceae	mon	MF	Brunnenkresse	top	DER	30	Milzbrand (gute Bläschen)
Lepidium spp. (*Lepidium off., Nasturtiun*	Brassicaceae	mon	MF	Brunnenkresse	syst	GAS	30	treibt Darmwürmer und andere Tierchen, die im Bauch ihr Wesen treiben
Lepidium spp. (*Lepidium off., Nasturtiun*	Brassicaceae	mon	MF	Brunnenkresse	syst	GYN	30	treibt die Leibesfrucht ab
Lepidium spp. (*Lepidium off., Nasturtiun*	Brassicaceae	mon	MF	Brunnenkresse	vol	OTH	30	vertreibt allein sein Duft sämtliche Schlangen
Lepidium spp. (*Lepidium off., Nasturtiun*	Brassicaceae	mon	MF	Brunnenkresse	syst	OTH	30	mildert Milzschwellung, wenn er häufig getrunken wird
Lepidium spp. (*Lepidium off., Nasturtiun*	Brassicaceae	mon	MF	Brunnenkresse	syst	RES	30	ein jeder Schmerz, der deine Brust beklemmt, bezähmt der Samen aber deinen Husten
Lepidium spp. (*Lepidium off., Nasturtiun*	Brassicaceae	mon	MF	Brunnenkresse	top	SKE	30	der schmerzenden Hüftgicht aufgelegt
Lepidium spp. (*Lepidium off., Nasturtiun*	Brassicaceae	mon	MF	Brunnenkresse	top	TEE	30	Zahnweh, giesst man die Saft ins Ohr der Kopfseite, wo der böse Zahn sitzt
Lepidium spp. (*Lepidium off., Nasturtiun*	Brassicaceae	ren	LF	Gartenkresse	syst	ANT	CCIIII / CXXXVI	widersteet er schlangen un gifftige thier
Lepidium spp. (*Lepidium off., Nasturtiun*	Brassicaceae	ren	LF	Gartenkresse	n.d.	APH	CCIIII / CXXXVI	reizt zu unkeüscheyt
Lepidium spp. (*Lepidium off., Nasturtiun*	Brassicaceae	ren	LF	Gartenkresse	n.d.	DER	CCIIII / CXXXVI	heylet alle blatern und schebigkeyt des grinds, böse masen und flecken
Lepidium spp. (*Lepidium off., Nasturtiun*	Brassicaceae	ren	LF	Gartenkresse	top	DER	CCIIII / CXXXVI	zerteylt er die kroepff
Lepidium spp. (*Lepidium off., Nasturtiun*	Brassicaceae	ren	LF	Gartenkresse	top	DER	CCIIII / CXXXVI	vertreibt die schuepen un grind auff dem haupt
Lepidium spp. (*Lepidium off., Nasturtiun*	Brassicaceae	ren	LF	Gartenkresse	top	DER	CCIIII / CXXXVI	zerteylt also gebraucht allerley geschwulst
Lepidium spp. (*Lepidium off., Nasturtiun*	Brassicaceae	ren	LF	Gartenkresse	top	DER	CCIIII / CXXXVI	heylet er die geschwaer un schaeden so umb sich fressen
Lepidium spp. (*Lepidium off., Nasturtiun*	Brassicaceae	ren	LF	Gartenkresse	top	DER	CCIIII / CXXXVI	behelt die har vest und steiff
Lepidium spp. (*Lepidium off., Nasturtiun*	Brassicaceae	ren	LF	Gartenkresse	top	EAR	CCIIII / CXXXVI	bringt wider das gehoer
Lepidium spp. (*Lepidium off., Nasturtiun*	Brassicaceae	ren	LF	Gartenkresse	syst	EYE	CCIIII / CXXXVI	reynigt auch das haupt un scherpfft das gesicht
Lepidium spp. (*Lepidium off., Nasturtiun*	Brassicaceae	ren	LF	Gartenkresse	n.d.	GAS	CCIIII / CXXXVI	reyniget den bauch
Lepidium spp. (*Lepidium off., Nasturtiun*	Brassicaceae	ren	LF	Gartenkresse	syst	GAS	CCIIII / CXXXVI	treibt aus die gallen durch den stulgang

Lepidium spp. (*Lepidium off., Nasturtium*	Brassicaceae	ren	LF	Gartenkresse	n.d.	GYN	CCIIII / CXXXVI	bringt den frawen ire krankheyt
Lepidium spp. (*Lepidium off., Nasturtium*	Brassicaceae	ren	LF	Gartenkresse	n.d.	NER	CCIIII / CXXXVI	scherpfft die sinn
Lepidium spp. (*Lepidium off., Nasturtium*	Brassicaceae	ren	LF	Gartenkresse	vol	OTH	CCIIII / CXXXVI	vertreibt alle dergleichen vergifftige thier
Lepidium spp. (*Lepidium off., Nasturtium*	Brassicaceae	ren	LF	Gartenkresse	top	OTH	CCIIII / CXXXVI	macht das milz kleiner
Lepidium spp. (*Lepidium off., Nasturtium*	Brassicaceae	ren	LF	Gartenkresse	syst	RES	CCIIII / CXXXVI	zerteylt er den zaehen schleim umb die brust. Ist derhalben dene so schwaerlich athmen un keichen seer nütz
Lepidium spp. (*Lepidium off., Nasturtium*	Brassicaceae	ren	LF	Gartenkresse	top	SKE	CCIIII / CXXXVI	denen so das hüftwee haben
Lepidium spp. (*Lepidium off., Nasturtium*	Brassicaceae	s pop	JK	Brunnenkresse	top	DER	p. 341	tilgen die Flecken der Haut
Lepidium spp. (*Lepidium off., Nasturtium*	Brassicaceae	s pop	JK	Brunnenkresse	syst	GAS	p. 341	reinigen Magen, Gedärme, vertreibt Würmer
Lepidium spp. (*Lepidium off., Nasturtium*	Brassicaceae	s pop	JK	Brunnenkresse	syst	RES	p. 341	reinigt Lunge
Lepidium spp. (*Lepidium off., Nasturtium*	Brassicaceae	s pop	JK	Brunnenkresse	syst	URO	p. 341	Nierenleiden, Steinleiden
Lepidium spp. (*Lepidium off., Nasturtium*	Brassicaceae	s pop	UB	Brunnenkresse	syst	RES	p. 205	Katarrhe der Atemwege
Lepidium spp. (*Lepidium off., Nasturtium*	Brassicaceae	s pop	UB	Brunnenkresse	syst	RES	p. 205	Katarrhe der Atemwege
Lepidium spp. (*Lepidium off., Nasturtium*	Brassicaceae	s pop	UB	Brunnenkresse	syst	URO	p. 248	Harnwegsinfekte
Lepidium spp. (*Lepidium off., Nasturtium*	Brassicaceae	s pop	UB	Brunnenkresse	syst	URO	p. 248	Harnwegsinfekte
Lepidium spp. (*Lepidium off., Nasturtium*	Brassicaceae	s pop	WI	Brunnenkresse	syst	HUM	p. 409	Volksheilkunde: Blutreinigungs- Mittel, Frühjahrskur
Lepidium spp. (*Lepidium off., Nasturtium*	Brassicaceae	sci	WI	Brunnenkresse	syst	GAS	p. 409	Cholagogum
Lepidium spp. (*Lepidium off., Nasturtium*	Brassicaceae	sci	WI	Brunnenkresse	syst	RES	p. 409	Katarrhe der Lutfwege
Lepidium spp. (*Lepidium off., Nasturtium*	Brassicaceae	sci	WI	Brunnenkresse	top	RES	p. 409	Dermatikum
Levisticum officinale L.	Apiaceae	ant	DIOS	Liebstöckel	syst	ANT	182	und gegen den Biß giftiger Tiere
Levisticum officinale L.	Apiaceae	ant	DIOS	Liebstöckel	syst	GAS	182	Dieser ist dem Magen sehr zuträglich
Levisticum officinale L.	Apiaceae	ant	DIOS	Liebstöckel	syst	GAS	182	die Verdauung befördernd, auch dient sie gegen Leiden der Eingeweide, zur Verdauung, gegen Ödeme und Blähungen, besonders des Magens
Levisticum officinale L.	Apiaceae	ant	DIOS	Liebstöckel	syst	GYN	182	befördern sie den Harn
Levisticum officinale L.	Apiaceae	ant	DIOS	Liebstöckel	syst	GYN	182	befördern [sie den Harn] und die Menstruation
Levisticum officinale L.	Apiaceae	ant	DIOS	Liebstöckel	syst	URO	182	befördern [sie den Harn] und die Menstruation
Levisticum officinale L.	Apiaceae	mon	HvB	Liebstöckel	syst	RES	1-139	in der Brust hustet
Levisticum officinale L.	Apiaceae	mon	HvB	Liebstöckel	top	RES	1-139	an Drüsen am Hals Schmerzen leidet, so dass die Halsadern aufgebläht sind
Levisticum officinale L.	Apiaceae	mon	MF	Liebstöckel	syst	ANT	25	heilt Giftbisse
Levisticum officinale L.	Apiaceae	mon	MF	Liebstöckel	top	ANT	25	heilt Giftbisse
Levisticum officinale L.	Apiaceae	mon	MF	Liebstöckel	syst	GAS	25	Verdauung fördern
Levisticum officinale L.	Apiaceae	mon	MF	Liebstöckel	syst	GAS	25	heilt einen geblähten Magen, unterstützt die Verdauungskraft, hilft allen Leiden der Gedärme
Levisticum officinale L.	Apiaceae	mon	MF	Liebstöckel	syst	GYN	25	sorgt für geordneten Monatsfluss
Levisticum officinale L.	Apiaceae	mon	MF	Liebstöckel	syst	URO	25	treibt den Harn
Levisticum officinale L.	Apiaceae	ren	LF	Liebstöckel	syst	ANT	CCCCXXXVI / CCXCII	ist gut denen so von den nattern und schlangen gebissen seind
Levisticum officinale L.	Apiaceae	ren	LF	Liebstöckel	top	DER	CCCCXXXVI / CCXCII	klar angesicht, wundheilung
Levisticum officinale L.	Apiaceae	ren	LF	Liebstöckel	top	DER	CCCCXXXVI / CCXCII	verzert sie allerley geschwulst und herte, sie heylet wunden zusammen
Levisticum officinale L.	Apiaceae	ren	LF	Liebstöckel	syst	GAS	CCCCXXXVI / CCXCII	stellen den bauchfluss
Levisticum officinale L.	Apiaceae	ren	LF	Liebstöckel	syst	GAS	CCCCXXXVI / CCXCII	gut zu allerley verstopfung der leber, der milz
Levisticum officinale L.	Apiaceae	ren	LF	Liebstöckel	syst	GAS	CCCCXXXVI / CCXCII	nimmt hinweg das aufblaehen des Magens
Levisticum officinale L.	Apiaceae	ren	LF	Liebstöckel	syst	GAS	CCCCXXXVI / CCXCII	hilft dem kalten magen
Levisticum officinale L.	Apiaceae	ren	LF	Liebstöckel	syst	GYN	CCCCXXXVI / CCXCII	bringt den frawen ihr zeit und treibt aus das nachbürdlin
Levisticum officinale L.	Apiaceae	ren	LF	Liebstöckel	syst	HUM	CCCCXXXVI / CCXCII	bringet den schweiss
Levisticum officinale L.	Apiaceae	ren	LF	Liebstöckel	syst	RES	CCCCXXXVI / CCXCII	lindert husten und den schweren athemb
Levisticum officinale L.	Apiaceae	ren	LF	Liebstöckel	syst	SKE	CCCCXXXVI / CCXCII	denen die das hüftwee haben
Levisticum officinale L.	Apiaceae	ren	LF	Liebstöckel	syst	URO	CCCCXXXVI / CCXCII	den wassersüchtigen
Levisticum officinale L.	Apiaceae	ren	LF	Liebstöckel	syst	URO	CCCCXXXVI / CCXCII	der nieren und blasen
Levisticum officinale L.	Apiaceae	ren	LF	Liebstöckel	syst	URO	CCCCXXXVI / CCXCII	zermalt den stein
Levisticum officinale L.	Apiaceae	ren	LF	Liebstöckel	syst	URO	CCCCXXXVI / CCXCII	treibt den harn
Levisticum officinale L.	Apiaceae	s pop	JK	Liebstöckel	syst	CAR	p. 389	Herzleiden
Levisticum officinale L.	Apiaceae	s pop	JK	Liebstöckel	top	DER	p. 389	Wundmittel
Levisticum officinale L.	Apiaceae	s pop	JK	Liebstöckel	syst	URO	p. 389	Wassersucht, Schwäche der Harnwerkzeuge
Levisticum officinale L.	Apiaceae	s pop	UB	Liebstöckel	syst	URO	p. 255	Durchspülung
Levisticum officinale L.	Apiaceae	s pop	UB	Liebstöckel	syst	URO	p. 255	Durchspülung
Levisticum officinale L.	Apiaceae	s pop	WI	Liebstöckel	syst	GAS	p. 341	Volksmedizin: Stomachikum, Karminativum
Levisticum officinale L.	Apiaceae	s pop	WI	Liebstöckel	syst	GYN	p. 341	Volksmedizin: Emenagogum
Levisticum officinale L.	Apiaceae	s pop	WI	Liebstöckel	syst	RES	p. 341	Volksmedizin: Katarrhe der Luftwege
Levisticum officinale L.	Apiaceae	sci	EMA	Liebstöckel	syst	URO	HMPC/524621/2011	to increase the amount of urine to achieve flushing of the urinary tract as an adjuvant in minor urinary complaints
Linum usitatissimum agg.	Linaceae	ant	DIOS	Lein	syst	APH	139	Mit Honig und Pfeffer gemischt und als Kuchen reichlich genommen reizt er zum Liebesgenuss
Linum usitatissimum agg.	Linaceae	ant	DIOS	Lein	syst	DER	139	er zerteilt und erweicht jede innere und äußere Geschwulst
Linum usitatissimum agg.	Linaceae	ant	DIOS	Lein	top	DER	139	entfernt er Sonnenbrandflecken und Finnen, mit Lauge zerteilt er Drüsen neben dem Ohre und Verhärtungen;mit Wein gekocht nimmt er fressende und grind- artige Geschwüre weg. Mit gleichviel Kresse und Honig entfernt er schuppige Nägel
Linum usitatissimum agg.	Linaceae	ant	DIOS	Lein	top	GAS	139	und zum Herausbefördern der Exkremente
Linum usitatissimum agg.	Linaceae	ant	DIOS	Lein	top	GYN	139	Die Abkochung desselben dient als Klistier bei Verwundungen der Eingeweide und der Gebärmutter
Linum usitatissimum agg.	Linaceae	ant	DIOS	Lein	top	GYN	139	bei Entzündung der Gebärmutter leistet sie, wie die des Bockshorns, als Sitzbad gute Dienste
Linum usitatissimum agg.	Linaceae	ant	DIOS	Lein	top	GYN	139	bei Entzündung der Gebärmutter leistet sie, wie die des Bockshorns, als Sitzbad gute Dienste
Linum usitatissimum agg.	Linaceae	ant	DIOS	Lein	syst	RES	139	reinigt er die Brust und lindert den Husten
Linum usitatissimum agg.	Linaceae	mon	HvB	Leinsamen	top	DER	1-194	vom Feuer verbrannt
Linum usitatissimum agg.	Linaceae	mon	HvB	Leinsamen	top	n.d.	1-194	Schmerzen in der Seite
Linum usitatissimum agg.	Linaceae	ren	LF	Lein	syst	APH	CCLXV / CLXXVIII	bringt lust zu den natürlichen wercken
Linum usitatissimum agg.	Linaceae	ren	LF	Lein	top	DER	CCLXV / CLXXVIII	zeytigt er allerley beulen und geschwaer, splitter, verhindert dass schaeden um sich fressen
Linum usitatissimum agg.	Linaceae	ren	LF	Lein	top	DER	CCLXV / CLXXVIII	erweycht und verzert er allerley beulen, vertreibt er allerley maelern von dem angesicht
Linum usitatissimum agg.	Linaceae	ren	LF	Lein	top	DER	CCLXV / CLXXVIII	ohrmützel und allerley geschwulst
Linum usitatissimum agg.	Linaceae	ren	LF	Lein	top	DER	CCLXV / CLXXVIII	hals geschwaeren
Linum usitatissimum agg.	Linaceae	ren	LF	Lein	top	DER	CCLXV / CLXXVIII	verletzt an daerme oder der muter
Linum usitatissimum agg.	Linaceae	ren	LF	Lein	top	DER	CCLXV / CLXXVIII	und nimmt hinweg die rauhen, scharpfen negel
Linum usitatissimum agg.	Linaceae	ren	LF	Lein	n.d.	EYE	CCLXV / CLXXVIII	scherfft das gesicht
Linum usitatissimum agg.	Linaceae	ren	LF	Lein	top	GAS	CCLXV / CLXXVIII	rote rhur, bauchfluss
Linum usitatissimum agg.	Linaceae	ren	LF	Lein	syst	RES	CCLXV / CLXXVIII	schwindsüchtigen
Linum usitatissimum agg.	Linaceae	ren	LF	Lein	syst	RES	CCLXV / CLXXVIII	reyniget er die brust und mildert den husten
Linum usitatissimum agg.	Linaceae	s pop	JK	Lein	top	DER	p. 358	Geschwulsten, Eissen, Furunkeln und Entzündungen
Linum usitatissimum agg.	Linaceae	s pop	JK	Lein	syst	GAS	p. 358	Unterleibsentzündungen, Darmentzündung, Kolik,
Linum usitatissimum agg.	Linaceae	s pop	JK	Lein	syst	RES	p. 358	starkem Husten, Brustleiden und Blutspeien
Linum usitatissimum agg.	Linaceae	s pop	JK	Lein	top	SKE	p. 358	Einreibmittel bei Rheumatismus
Linum usitatissimum agg.	Linaceae	s pop	KN	Lein	top	DER	p. 477	Brandsalbe
Linum usitatissimum agg.	Linaceae	s pop	KN	Lein	top	DER	p. 477	Geschwülsten und Geschwüren, wunde Füsse
Linum usitatissimum agg.	Linaceae	s pop	KN	Lein	syst	GAS	p. 477	mildes Abführmittel
Linum usitatissimum agg.	Linaceae	s pop	KN	Lein	syst	GAS	p. 477	katarrhalischen Magenveränderungen, Magen- und Zwölffingerdarmgeschwüren
Linum usitatissimum agg.	Linaceae	s pop	KN	Lein	syst	URO	p. 477	Nieren-, Nierenbeckenentzündung, Blasenentzündung, Gallenstein- Nierenstein-Koliken
Linum usitatissimum agg.	Linaceae	s pop	UB	Leinsamen	top	DER	p. 314	Abszesse, Furunkel, Karbunkel
Linum usitatissimum agg.	Linaceae	s pop	UB	Leinsamen	top	GAS	p. 130	Schleimmittel zum Abführen
Linum usitatissimum agg.	Linaceae	s pop	UB	Leinsamen	top	GAS	p. 165	Sodbrennen, Gastritis, Magen- und Zwölffingerdarmgeschwür
Linum usitatissimum agg.	Linaceae	s pop	WI	Lein	syst	GAS	p. 347	Volksmedizin: akuter und chronischer Gastritis
Linum usitatissimum agg.	Linaceae	sci	EMA	Leinsamen	syst	GAS	list entry draft	Traditional herbal medicinal product for use as a demulcent preparation for the symptomatic treatment of gastrointestinal discomfort exclusively based on long-standing use
Linum usitatissimum agg.	Linaceae	sci	ESCOP	Lini Semen	top	DER	I p. 290	painful skin inflammations
Linum usitatissimum agg.	Linaceae	sci	ESCOP	Lini Semen	syst	GAS	I p. 290	constipation, irritable bowel syndrome, diverticular disease, symptomatic short-term treatment of gastritis and eteritis

Linum usitatissimum agg.	Linaceae	sci	WI	Lein	syst	GAS	p. 347	Quell-Abführmittel
Malus sylvestris agg.	Rosaceae	ant	DIOS	Apfel	n.d.	OTH	108	Auch die unreife Frucht erweist sich zusammenziehend, die ausgereifte aber nicht in gleichem Grade.
Malus sylvestris agg.	Rosaceae	ant	DIOS	Apfel	n.d.	OTH	108	Die Blätter, Blühen und Zweige von jeglichem Apfelbaum, am meisten von der Quitte, adstringiren
Malus sylvestris agg.	Rosaceae	mon	HvB	Apfelbaum	top	EYE	3-1	irgendeine Augentrübung
Malus sylvestris agg.	Rosaceae	mon	HvB	Apfelbaum	top	GAS	3-1	Leber- und Milzschwäche, oder von üblen Säften des Bauches oder Magens
Malus sylvestris agg.	Rosaceae	mon	HvB	Apfelbaum	top	NER	3-1	Migräne im Kopf
Malus sylvestris agg.	Rosaceae	mon	HvB	Apfelbaum	syst	OTH	3-1	gut zu essen für Gesunde und Kranke
Malus sylvestris agg.	Rosaceae	mon	HvB	Apfelbaum	top	SKE	3-1	Schmerzen in Schultern, Bauch, Lenden
Malus sylvestris agg.	Rosaceae	mon	HvB	Apfelbaum	top	SKE	3-1	Gich in den Nieren und Lendengegend
Malus sylvestris agg.	Rosaceae	ren	TAB	Apfelbaum	syst	ANT	n.d.	widerstreben sie dem Gifft/ und treiben die Würm auss dem Leib
Malus sylvestris agg.	Rosaceae	ren	TAB	Apfelbaum	top	EYE	n.d.	uber das Aug gelegt/ stillet die Schmertzen und Wehetagen desselbig
Malus sylvestris agg.	Rosaceae	ren	TAB	Apfelbaum	syst	FEV	n.d.	werden nützlich in den Fiebern im Mund gehalten/ denselbigen darmit
Malus sylvestris agg.	Rosaceae	ren	TAB	Apfelbaum	syst	GAS	n.d.	welchen der Mage von vieler Feuchtigkeit verderbt ist / erweichen etw
Malus sylvestris agg.	Rosaceae	ren	TAB	Apfelbaum	syst	OTH	n.d.	und treiben die Würm auss dem Leib
Malus sylvestris agg.	Rosaceae	ren	TAB	Apfelbaum	top	OTH	n.d.	stechen der Seiten
Malus sylvestris agg.	Rosaceae	s pop	JK	Apfelbaum	syst	CAR	p. 324	Herzklopfen, Herzschwäche
Malus sylvestris agg.	Rosaceae	s pop	JK	Apfelbaum	top	DER	p. 323	desinfiziert die Mundhöle
Malus sylvestris agg.	Rosaceae	s pop	JK	Apfelbaum	top	DER	p. 324	Halsleiden, Mandelschwellungen und Geschwüren
Malus sylvestris agg.	Rosaceae	s pop	JK	Apfelbaum	syst	FEV	p. 324	Fieber
Malus sylvestris agg.	Rosaceae	s pop	JK	Apfelbaum	syst	GAS	p. 323	regt die Leber an, bindet die überschüssigen Säuren des Magens, födert die Tätigkeit der [Nieren] und Gedärme, schützt vor Verdauungsbeschwerden, Fettleibigkeit, Durchfall
Malus sylvestris agg.	Rosaceae	s pop	JK	Apfelbaum	syst	NER	p. 323	wirkt vorteilhaft auf das Gehirn
Malus sylvestris agg.	Rosaceae	s pop	JK	Apfelbaum	syst	NER	p. 324	stärkt die Nerven, Gedächnis
Malus sylvestris agg.	Rosaceae	s pop	JK	Apfelbaum	syst	RES	p. 323	schützt vor Halskrankheiten
Malus sylvestris agg.	Rosaceae	s pop	JK	Apfelbaum	syst	URO	p. 324	reinigt das Blut, Stein- und Gichtleiden, Nieren und Blasenkrankheiten
Malva sylvestris agg.	Malvaceae	ant	DIOS	Malve	syst	ANT	144	hilft gegen alle tödlichen Gifte, die, welche sie genießen, müssen aber anhaltend erbrechen; ferner gegen den Biss der Spinne
Malva sylvestris agg.	Malvaceae	ant	DIOS	Malve	top	DER	144	Aufgestrichen wirkt sie auch gegen die Stiche der Bienen und Wespen
Malva sylvestris agg.	Malvaceae	ant	DIOS	Malve	top	DER	144	zur Vernarbung
Malva sylvestris agg.	Malvaceae	ant	DIOS	Malve	top	DER	144	heilt sie Schorf und Kleingrind, so heilen sie Feuerbrandwunden und roseartige Entzündungen
Malva sylvestris agg.	Malvaceae	ant	DIOS	Malve	top	DER	144	gegen Beschädigungen der Eingeweide, der Gebärmutter und des Afters
Malva sylvestris agg.	Malvaceae	ant	DIOS	Malve	top	EYE	144	Umschlag die Kraft, die Tränenfistel (Aegilopie) zu heilen
Malva sylvestris agg.	Malvaceae	ant	DIOS	Malve	n.d.	GAS	144	aber gut für den Bauch und am besten die Stengel
Malva sylvestris agg.	Malvaceae	ant	DIOS	Malve	top	GYN	144	als Sitzbad erweicht die Gebärmutter
Malva sylvestris agg.	Malvaceae	ant	DIOS	Malve	syst	GYN	144	befördert die Milchabsonderung
Malva sylvestris agg.	Malvaceae	ant	DIOS	Malve	top	OTH	144	bleibt er von den Stichen verschont
Malva sylvestris agg.	Malvaceae	ant	DIOS	Malve	syst	URO	144	lindert die Schmerzen der Blase
Malva sylvestris agg.	Malvaceae	ant	DIOS	Malve	n.d.	URO	144	Heilsam ist sie für die Eingeweide und die Blase.
Malva sylvestris agg.	Malvaceae	mon	HvB	Malve	top	EYE	1-97	Sehvermögen der Augen erhellen
Malva sylvestris agg.	Malvaceae	mon	HvB	Malve	syst	GAS	1-97	kranker Magen
Malva sylvestris agg.	Malvaceae	mon	HvB	Malve	top	NER	1-97	Melancholie im Gehirn Schmerzen bereitet
Malva sylvestris agg.	Malvaceae	mon	MF	Malve	syst	ANT	62	begegne sie giftigen Tränken
Malva sylvestris agg.	Malvaceae	mon	MF	Malve	n.d.	APH	62	soll Liebeskraft erregen
Malva sylvestris agg.	Malvaceae	mon	MF	Malve	n.d.	APO	62	Krankheit der Brüste abwehren
Malva sylvestris agg.	Malvaceae	mon	MF	Malve	top	DER	62	Brandwunden lindern
Malva sylvestris agg.	Malvaceae	mon	MF	Malve	top	DER	62	heilst du Bienenstiche
Malva sylvestris agg.	Malvaceae	mon	MF	Malve	top	DER	62	schändliche Kopfgrindflechten und Schuppen
Malva sylvestris agg.	Malvaceae	mon	MF	Malve	top	DER	62	blutende Wunden geheilt werden, Prellungen
Malva sylvestris agg.	Malvaceae	mon	MF	Malve	top	EYE	62	Augengeschwüre
Malva sylvestris agg.	Malvaceae	mon	MF	Malve	syst	GAS	62	nützt sie den Därmen
Malva sylvestris agg.	Malvaceae	mon	MF	Malve	top	GAS	62	Krankheitsfällen an den Därmen sowie am After
Malva sylvestris agg.	Malvaceae	mon	MF	Malve	top	GYN	62	abreibend auf die Leibesfrucht
Malva sylvestris agg.	Malvaceae	mon	MF	Malve	top	GYN	62	Verhärtungen in der Gebärmutter erweicht
Malva sylvestris agg.	Malvaceae	mon	MF	Malve	top	OTH	62	Antoniusfeuer löschen
Malva sylvestris agg.	Malvaceae	mon	MF	Malve	top	TEE	62	weher Zahn
Malva sylvestris agg.	Malvaceae	mon	MF	Malve	syst	URO	62	heile sie die Blase
Malva sylvestris agg.	Malvaceae	ren	LF	Malve	top	ANT	CCLXXXIX / CXCIII	heylen die stich der imen, bisse der gifftigen thier
Malva sylvestris agg.	Malvaceae	ren	LF	Malve	top	DER	CCLXXXIX / CXCIII	geschwaer so zwüschen der nasen und den augen sich erheben
Malva sylvestris agg.	Malvaceae	ren	LF	Malve	top	DER	CCLXXXIX / CXCIII	fliessenden erbgrind (?) und die schuppen auf dem haupt, rotlauff und brandt, allerley spreissel
Malva sylvestris agg.	Malvaceae	ren	LF	Malve	syst	DER	CCLXXXIX / CXCIII	geschwaer so zwüschen der nasen und den augen sich erheben
Malva sylvestris agg.	Malvaceae	ren	LF	Malve	top	GAS	CCLXXXIX / CXCIII	heylet die schwierigen daerm, muter und den hindern
Malva sylvestris agg.	Malvaceae	ren	LF	Malve	syst	GYN	CCLXXXIX / CXCIII	macht den frawen milch, leichter geberen
Malva sylvestris agg.	Malvaceae	ren	LF	Malve	top	GYN	CCLXXXIX / CXCIII	erweichen die verherte muter
Malva sylvestris agg.	Malvaceae	ren	LF	Malve	syst	NER	CCLXXXIX / CXCIII	macht schlaffen
Malva sylvestris agg.	Malvaceae	ren	LF	Malve	top	RES	CCLXXXIX / CXCIII	gebresten der brust und lungen
Malva sylvestris agg.	Malvaceae	ren	LF	Malve	syst	URO	CCLXXXIX / CXCIII	schmerzen der blasen
Malva sylvestris agg.	Malvaceae	s pop	BVA	Käslikraut - Malve	top	DER	p. 121	Mundschleimhautentzündung
Malva sylvestris agg.	Malvaceae	s pop	BVA	Käslikraut - Malve	top	DER	p. 121	Furunkeln, Umleauf, Eiterherden, entzündeten Wunden, Nagelbettentzündung, nässenden und eitrigen Ekzemen, Hautentzündungen
Malva sylvestris agg.	Malvaceae	s pop	BVA	Käslikraut - Malve	syst	GAS	p. 121	Magen- Darmkoliken, Gastritis
Malva sylvestris agg.	Malvaceae	s pop	BVA	Käslikraut - Malve	syst	GAS	p. 121	Magen- Darmkoliken, Gastritis
Malva sylvestris agg.	Malvaceae	s pop	BVA	Käslikraut - Malve	syst	GYN	p. 121	Entzündungen im Unterleib der Frau
Malva sylvestris agg.	Malvaceae	s pop	BVA	Käslikraut - Malve	syst	GYN	p. 121	Entzündungen im Unterleib der Frau
Malva sylvestris agg.	Malvaceae	s pop	BVA	Käslikraut - Malve	syst	RES	p. 121	Erkrankungen der Atmungsorgane und Lungenerkrankungen; Bronchitis, Husten, Heiserkeit, Rachen- und Kehlkopfkatarrh, Angina, Mandelentzündungen und Lungenemphysem
Malva sylvestris agg.	Malvaceae	s pop	BVA	Käslikraut - Malve	syst	RES	p. 121	Erkrankungen der Atmungsorgane und Lungenerkrankungen; Bronchitis, Husten, Heiserkeit, Rachen- und Kehlkopfkatarrh, Angina, Mandelentzündungen und Lungenemphysem
Malva sylvestris agg.	Malvaceae	s pop	JK	Wilde Malve	top	DER	p. 394	Insektenstichen
Malva sylvestris agg.	Malvaceae	s pop	JK	Wilde Malve	top	DER	p. 394	Geschwulsten und Eiterungen
Malva sylvestris agg.	Malvaceae	s pop	JK	Wilde Malve	syst	GAS	p. 394	schwache Därme, Darmgeschwüre
Malva sylvestris agg.	Malvaceae	s pop	JK	Wilde Malve	syst	OTH	p. 394	allen inneren Entzündungen
Malva sylvestris agg.	Malvaceae	s pop	JK	Wilde Malve	syst	OTH	p. 394	allen inneren Entzündungen
Malva sylvestris agg.	Malvaceae	s pop	JK	Wilde Malve	top	SKE	p. 394	Quetschungen
Malva sylvestris agg.	Malvaceae	s pop	JK	Wilde Malve	syst	URO	p. 394	bricht Steine in Nieren und Blase
Malva sylvestris agg.	Malvaceae	s pop	UB	Malve	top	DER	p. 330	allgemeine Behanldung von Ekzemen, Neurodermitis, Psoriasis
Malva sylvestris agg.	Malvaceae	s pop	UB	Malve	top	DER	p. 330	allgemeine Behandlung von Ekzemen, Neurodermitis, Psoriasis
Malva sylvestris agg.	Malvaceae	s pop	UB	Malve	syst	GAS	p. 166	Sodbrennen, Gastritis, Magen- und Zwölffingerdarmgeschwür
Malva sylvestris agg.	Malvaceae	s pop	UB	Malve	syst	RES	p. 166	unproduktiver Reizhusten
Malva sylvestris agg.	Malvaceae	s pop	UB	Malve	top	RES	p. 166	Entzündungen der Mund- und Rachenschleimhaut
Malva sylvestris agg.	Malvaceae	s pop	WI	Malve	top	DER	p. 364	Volksmedizin:Wundbehandlung
Malva sylvestris agg.	Malvaceae	sci	ESCOP	Malvae flos	syst	GAS	II p. 157	irritation of the gastric mucosa
Malva sylvestris agg.	Malvaceae	sci	ESCOP	Malvae flos	syst	RES	II p. 157	dry cough, irritation of the oral, pharyngal
Malva sylvestris agg.	Malvaceae	sci	ESCOP	Malvae flos	top	RES	II p. 157	irritation of the oral, pharyngal or gastric mucosa
Malva sylvestris agg.	Malvaceae	sci	WI	Malve	syst	GAS	p. 364	Gastroenteritis
Malva sylvestris agg.	Malvaceae	sci	WI	Malve	syst	GAS	p. 368	Magen- Darmentzündungen
Malva sylvestris agg.	Malvaceae	sci	WI	Malve	syst	RES	p. 364	Erkältungskrankheiten
Malva sylvestris agg.	Malvaceae	sci	WI	Malve	syst	RES	p. 368	Erkältungskrankheiten
Marrubium vulgare L.	Lamiaceae	ant	DIOS	Andorn	top	DER	198	reinigen schmutzige Geschwüre, halten Pterygien und fressende Geschwüre auf
Marrubium vulgare L.	Lamiaceae	ant	DIOS	Andorn	top	EAR	198	ist ein gutes Mittel bei Ohrenschmerzen
Marrubium vulgare L.	Lamiaceae	ant	DIOS	Andorn	top	EYE	198	schärft auch das Gesicht
Marrubium vulgare L.	Lamiaceae	ant	DIOS	Andorn	syst	EYE	198	Phthisis
Marrubium vulgare L.	Lamiaceae	ant	DIOS	Andorn	syst	GAS	198	beseitigt ferner auch die Gelbsucht durch die Nase

Marrubium vulgare L.	Lamiaceae	ant	DIOS	Andorn	top	OTH	198	und lindern Seitenschmerzen
Marrubium vulgare L.	Lamiaceae	ant	DIOS	Andorn	syst	RES	198	Asthma und Husten Leidenden gegeben; fahren sie auch den dicken Schleim aus der Brust.
Marrubium vulgare L.	Lamiaceae	ant	DIOS	Andorn	syst	RES	198	die Menstruation und die Nachgeburt zu befördern, ebenso auch denen, die eine schwere Geburt haben
Marrubium vulgare L.	Lamiaceae	ant	DIOS	Andorn	syst	RES	198	die von giftigen Tieren gebissen sind und die Gift geschluckt haben
Marrubium vulgare L.	Lamiaceae	ant	DIOS	Andorn	syst	RES	198	Der Blase aber und den Nieren sind sie nicht zuträglich
Marrubium vulgare L.	Lamiaceae	mon	HvB	Andorn	top	EAR	1-33	taube Ohren
Marrubium vulgare L.	Lamiaceae	mon	HvB	Andorn	syst	GAS	1-33	kranke und gebrochene Eingeweide
Marrubium vulgare L.	Lamiaceae	mon	HvB	Andorn	syst	RES	1-33	kranke Kehle
Marrubium vulgare L.	Lamiaceae	mon	HvB	Andorn	syst	RES	1-33	Husten (Fenchel, Dill)
Marrubium vulgare L.	Lamiaceae	mon	MF	Andorn	top	DER	42	reinigt eiternde Wunden und führt Geschwüre, die das Fleisch wegfressen einer Heilung zu
Marrubium vulgare L.	Lamiaceae	mon	MF	Andorn	top	EAR	42	drückenden Ohrenschmerz beheben
Marrubium vulgare L.	Lamiaceae	mon	MF	Andorn	top	EYE	42	macht der die schwachen, trüben Augen hellsichtig
Marrubium vulgare L.	Lamiaceae	mon	MF	Andorn	top	GAS	42	hilft er Gelbsüchtigen
Marrubium vulgare L.	Lamiaceae	mon	MF	Andorn	syst	GYN	42	beschleunigt die Kindsgeburt und Nachgeburt
Marrubium vulgare L.	Lamiaceae	mon	MF	Andorn	syst	n.d.	42	den Seitenschmerz beruhigen
Marrubium vulgare L.	Lamiaceae	mon	MF	Andorn	syst	RES	42	Schwindsüchtige
Marrubium vulgare L.	Lamiaceae	mon	MF	Andorn	syst	RES	42	bezähmt die verschiedenen Brustkrankheiten, hilft den Astmathikern und treibt den husten fort
Marrubium vulgare L.	Lamiaceae	ren	LF	Andorn	syst	ANT	CCCXXXXV / CCXXV	denen so von nattern gebissen sind oder gifft getrunken haben
Marrubium vulgare L.	Lamiaceae	ren	LF	Andorn	top	DER	CCCXXXXV / CCXXV	renygen die alten schaeden, die geschwaer der negel, und verhueten dass die geschwaer um sich fressen, heylet die flecht
Marrubium vulgare L.	Lamiaceae	ren	LF	Andorn	top	DER	CCCXXXXV / CCXXV	verzehren die kropff
Marrubium vulgare L.	Lamiaceae	ren	LF	Andorn	top	EAR	CCCXXXXV / CCXXV	leht den schmerzen der ohren
Marrubium vulgare L.	Lamiaceae	ren	LF	Andorn	top	EYE	CCCXXXXV / CCXXV	macht klare augen
Marrubium vulgare L.	Lamiaceae	ren	LF	Andorn	syst	GAS	CCCXXXXV / CCXXV	eröffnen die verstopffte elber und die milz
Marrubium vulgare L.	Lamiaceae	ren	LF	Andorn	syst	GYN	CCCXXXXV / CCXXV	bringen den frawen ir krankheyt und treiben aus das nachbürdlin, so schwerlich geberen
Marrubium vulgare L.	Lamiaceae	ren	LF	Andorn	top	n.d.	CCCXXXXV / CCXXV	lindern die schmerzen der seiten
Marrubium vulgare L.	Lamiaceae	ren	LF	Andorn	syst	RES	CCCXXXXV / CCXXV	denen so schwerlich athmen, den husten und die schwindsüchtigen
Marrubium vulgare L.	Lamiaceae	ren	LF	Andorn	syst	RES	CCCXXXXV / CCXXV	denen so schwerlich athmen, den husten und die schwindsüchtigen
Marrubium vulgare L.	Lamiaceae	s pop	BVA	Andorn	syst	GAS	p. 20	Galle
Marrubium vulgare L.	Lamiaceae	s pop	BVA	Andorn	syst	GAS	p. 20	Galle
Marrubium vulgare L.	Lamiaceae	s pop	BVA	Andorn	syst	GYN	p. 20	schwache Menstruation
Marrubium vulgare L.	Lamiaceae	s pop	BVA	Andorn	syst	GYN	p. 20	schwache Menstruation
Marrubium vulgare L.	Lamiaceae	s pop	BVA	Andorn	syst	OTH	p. 20	Unterfunktion des Knochenmarks
Marrubium vulgare L.	Lamiaceae	s pop	BVA	Andorn	syst	OTH	p. 20	Unterfunktion des Knochenmarks
Marrubium vulgare L.	Lamiaceae	s pop	BVA	Andorn	syst	RES	p.20	Asthma, Bronchitis, Keuchhusten, chronischem Husten,
Marrubium vulgare L.	Lamiaceae	s pop	BVA	Andorn	syst	RES	p.20	Asthma, Bronchitis, Keuchhusten, chronischem Husten,
Marrubium vulgare L.	Lamiaceae	s pop	BVA	Andorn	syst	TON	p.20	elderly people
Marrubium vulgare L.	Lamiaceae	s pop	BVA	Andorn	syst	TON	p.20	elderly people
Marrubium vulgare L.	Lamiaceae	s pop	JK	Andorn	top	DER	p. 322	Hautkrankheiten
Marrubium vulgare L.	Lamiaceae	s pop	JK	Andorn	syst	GAS	p. 322	Reinigung und Kräftigung von Leber und Milz, Verschleimung der Leber, Gelbsucht und gegen die Würmer
Marrubium vulgare L.	Lamiaceae	s pop	JK	Andorn	syst	GYN	p. 322	monatliche Periode Schmerzen empfinden, säubern den Unterleib
Marrubium vulgare L.	Lamiaceae	s pop	JK	Andorn	syst	NER	p. 322	Nervenschwäche
Marrubium vulgare L.	Lamiaceae	s pop	JK	Andorn	syst	RES	p. 322	Reinigung und Kräftigung von Lungen, Brustverschleimung, Andorntee kräftigt die Brust und stillt Blutspeien und Krämpfe
Marrubium vulgare L.	Lamiaceae	s pop	JK	Andorn	syst	URO	p. 322	hilft denen die schwierig unrinieren, löst Sand und Stein auf
Marrubium vulgare L.	Lamiaceae	s pop	UB	Andorn	syst	GAS	p. 150	reine Bittermittel
Marrubium vulgare L.	Lamiaceae	s pop	UB	Andorn	syst	GAS	p. 150	reine Bittermittel
Marrubium vulgare L.	Lamiaceae	sci	EMA	Andorn	syst	GAS	HMPC/604271/2012	Traditional herbal medicinal product used for symptomatic treatment of mild dyspeptic complaints such as bloating and flatulence./ Traditional herbal medicinal product used in temporary loss of appetite
Marrubium vulgare L.	Lamiaceae	sci	EMA	Andorn	syst	GAS	HMPC/604271/2012	Traditional herbal medicinal product used for symptomatic treatment of mild dyspeptic complaints such as bloating and flatulence./ Traditional herbal medicinal product used in temporary loss of appetite
Marrubium vulgare L.	Lamiaceae	sci	EMA	Andorn	syst	GAS	HMPC/604271/2012	Traditional herbal medicinal product used for symptomatic treatment of mild dyspeptic complaints such as bloating and flatulence./ Traditional herbal medicinal product used in temporary loss of appetite
Marrubium vulgare L.	Lamiaceae	sci	EMA	Andorn	syst	GAS	HMPC/604271/2012	Traditional herbal medicinal product used for symptomatic treatment of mild dyspeptic complaints such as bloating and flatulence./ Traditional herbal medicinal product used in temporary loss of appetite
Marrubium vulgare L.	Lamiaceae	sci	EMA	Andorn	syst	RES	HMPC/604271/2012	Traditional herbal medicinal product used as an expectorant in cough associated with cold.
Marrubium vulgare L.	Lamiaceae	sci	EMA	Andorn	syst	RES	HMPC/604271/2012	Traditional herbal medicinal product used as an expectorant in cough associated with cold.
Marrubium vulgare L.	Lamiaceae	sci	EMA	Andorn	syst	RES	HMPC/604271/2012	Traditional herbal medicinal product used as an expectorant in cough associated with cold.
Matricaria chamomilla L.	Asteraceae	ant	DIOS	Kamille	top	DER	206	helfen sie auch bei Geissauge
Matricaria chamomilla L.	Asteraceae	ant	DIOS	Kamille	top	DER	206	heilen sie Soor
Matricaria chamomilla L.	Asteraceae	ant	DIOS	Kamille	top	FEV	206	um das periodische Fieber zu vertreiben
Matricaria chamomilla L.	Asteraceae	ant	DIOS	Kamille	syst	GAS	206	gegen Blähungen und Darmverschlingung getrunken, vertreiben die Gelbsucht und heilen Leberleiden
Matricaria chamomilla L.	Asteraceae	ant	DIOS	Kamille	syst	GYN	206	im Trank und Sitzbade befördern sie die Menstruation, treiben den Embryo aus
Matricaria chamomilla L.	Asteraceae	ant	DIOS	Kamille	top	GYN	206	im Trank und Sitzbade befördern sie die Menstruation, treiben den Embryo aus
Matricaria chamomilla L.	Asteraceae	ant	DIOS	Kamille	syst	URO	206	sowie den Stein und den Urin; Abkochung gegen Blasenentzündung
Matricaria chamomilla L.	Asteraceae	ant	DIOS	Kamille	top	URO	206	sowie den Stein und den Urin
Matricaria chamomilla L.	Asteraceae	mon	HvB	Kamille	syst	GAS	1-116	wer in den Eingeweiden Schmerzen hat
Matricaria chamomilla L.	Asteraceae	mon	HvB	Kamille	syst	GYN	1-116	und wenn die Frauen den Monatsfluss haben, sollen sie dies Suppe, wie vorhin gesagt, bereiten und essen, und dies bereitet eine angenehme und leichte Reinigung . . .
Matricaria chamomilla L.	Asteraceae	mon	MF	Kamille	syst	ANT	14	so hindert si durch diesen Trunk verderbenbringende Schlangenbisse
Matricaria chamomilla L.	Asteraceae	mon	MF	Kamille	top	DER	14	tilgt es Schuppen und Leberflecken im Gesicht
Matricaria chamomilla L.	Asteraceae	mon	MF	Kamille	top	DER	14	Hautentzündungen
Matricaria chamomilla L.	Asteraceae	mon	MF	Kamille	top	EYE	14	Augenschwellungen sowie eiter fressende Schwären
Matricaria chamomilla L.	Asteraceae	mon	MF	Kamille	top	FEV	14	Fieberkranken wärmend salbst, wirst du Schüttelfrost und oftmals Fieber selbst vertreiben
Matricaria chamomilla L.	Asteraceae	mon	MF	Kamille	syst	GAS	14	beruhigt das Grimmen und eine Aufblähung des Magens, hilft Gelbsüchtigen, bei Beschwerden des Magens
Matricaria chamomilla L.	Asteraceae	mon	MF	Kamille	syst	GYN	14	sorgt für ordentlichen Monatsfluss, mit Wein getrunken die Leibesfrucht abtreiben
Matricaria chamomilla L.	Asteraceae	mon	MF	Kamille	top	n.d.	14	geschwollener Weichbauch behandeln
Matricaria chamomilla L.	Asteraceae	mon	MF	Kamille	syst	URO	14	treibt Harn, zerbricht die Blasensteine
Matricaria chamomilla L.	Asteraceae	mon	MF	Kamille	syst	URO	14	reinigt sie durch den Harnfluss die Milz
Matricaria chamomilla L.	Asteraceae	ren	LF	Kamille	syst	ANT	XIII / VIII	denen so von nattern gebissen sind
Matricaria chamomilla L.	Asteraceae	ren	LF	Kamille	top	DER	XIII / VIII	vetreiben die mundfeule
Matricaria chamomilla L.	Asteraceae	ren	LF	Kamille	top	DER	XIII / VIII	heylet alte wunden und schaeden
Matricaria chamomilla L.	Asteraceae	ren	LF	Kamille	top	EYE	XIII / VIII	fisteln der augen
Matricaria chamomilla L.	Asteraceae	ren	LF	Kamille	top	FEV	XIII / VIII	clystier im fieber gebraucht

Matricaria chamomilla L.	Asteraceae	ren	LF	Kamille	syst	GAS	XIII / VIII	vertreiben den blaest und wind und auch den schmerz der kleinen daerm, reinigt die geelsüchtigen und ist nützlich den lebersüchtigen
Matricaria chamomilla L.	Asteraceae	ren	LF	Kamille	syst	GYN	XIII / VIII	bringen den frawen ir zeyt
Matricaria chamomilla L.	Asteraceae	ren	LF	Kamille	top	GYN	XIII / VIII	bringen den frawen ir zeyt
Matricaria chamomilla L.	Asteraceae	ren	LF	Kamille	top	SKE	XIII / VIII	lindert auch allerley schmerzen und nimpt hinweg die muede der glieder
Matricaria chamomilla L.	Asteraceae	ren	LF	Kamille	syst	URO	XIII / VIII	treiben den harn und den stein
Matricaria chamomilla L.	Asteraceae	ren	LF	Kamille	top	URO	XIII / VIII	treiben den harn und den stein, lindern den schmerzen der blase
Matricaria chamomilla L.	Asteraceae	s pop	BVA	Kamille	syst	GAS	p. 115	Magenkrämpfe, Magenschleimhautkatarrh, Koliken im Magen- Darm-Trakt, krampfartige Unterleibsschmerzen, Blähungen, Durchfall, Brechreiz
Matricaria chamomilla L.	Asteraceae	s pop	BVA	Kamille	syst	NER	p. 115	innere Unruhe
Matricaria chamomilla L.	Asteraceae	s pop	BVA	Kamille	top	RES	p. 115	Schnupfen, Neben- und Stirnhöhlenkatarrh
Matricaria chamomilla L.	Asteraceae	s pop	BVA	Kamille	syst	TEE	p. 115	Entzündung in Mund- und Rachenhöhle, Linderung nach Zahnoperationen
Matricaria chamomilla L.	Asteraceae	s pop	BVA	Kamille	syst	URO	p. 115	Blasenentzündung
Matricaria chamomilla L.	Asteraceae	s pop	JK	Kamille	top	DER	p. 380	Geschwulsten, Beulen
Matricaria chamomilla L.	Asteraceae	s pop	JK	Kamille	top	DER	p. 380	Zahnoperation
Matricaria chamomilla L.	Asteraceae	s pop	JK	Kamille	top	EAR	p. 380	Ohrenleiden
Matricaria chamomilla L.	Asteraceae	s pop	JK	Kamille	top	EYE	p. 380	Augenleiden
Matricaria chamomilla L.	Asteraceae	s pop	JK	Kamille	syst	GAS	p. 380	Gelbsucht
Matricaria chamomilla L.	Asteraceae	s pop	JK	Kamille	top	GAS	p. 380	Blähungen, Druck
Matricaria chamomilla L.	Asteraceae	s pop	JK	Kamille	syst	GYN	p. 380	inneren Kräpfen (der Frauen)
Matricaria chamomilla L.	Asteraceae	s pop	JK	Kamille	top	GYN	p. 380	inneren Kräpfen (der Frauen)
Matricaria chamomilla L.	Asteraceae	s pop	JK	Kamille	top	NER	p. 380	Kopfschmerzen
Matricaria chamomilla L.	Asteraceae	s pop	JK	Kamille	syst	RES	p. 380	Enge auf der Brust
Matricaria chamomilla L.	Asteraceae	s pop	JK	Kamille	top	SKE	p. 380	Quetschungen, Rheumatismus, Gicht
Matricaria chamomilla L.	Asteraceae	s pop	JK	Kamille	syst	URO	p. 380	Steinleiden
Matricaria chamomilla L.	Asteraceae	s pop	KN	Kamille	top	DER	p. 476	Spülflüssigkeit (Entzündungen der Mund- und Halsschleimhäute, Angina . .Zanfleischeiterungen)
Matricaria chamomilla L.	Asteraceae	s pop	KN	Kamille	top	EYE	p. 476	Spülflüssigkeit (Lidrand)
Matricaria chamomilla L.	Asteraceae	s pop	KN	Kamille	syst	FEV	p. 476	schweisstreibendes Mittel
Matricaria chamomilla L.	Asteraceae	s pop	KN	Kamille	top	n.d.	p. 476	n.d.
Matricaria chamomilla L.	Asteraceae	s pop	KN	Kamille	syst	RES	p. 476	Erkältungen
Matricaria chamomilla L.	Asteraceae	s pop	KN	Kamille	top	URO	p. 476	Spülflüssigkeit (Unterleibs- und Blasenkatarrh)
Matricaria chamomilla L.	Asteraceae	s pop	MT	Kamille	top	DER	p. 29	Wundschmerzen
Matricaria chamomilla L.	Asteraceae	s pop	MT	Kamille	syst	GAS	p. 29	Blähungen, Durchfall, Magenleiden, - Verschleimungen
Matricaria chamomilla L.	Asteraceae	s pop	MT	Kamille	syst	GYN	p. 29	Menstruationsstörungen
Matricaria chamomilla L.	Asteraceae	s pop	MT	Kamille	syst	NER	p. 29	Schlaflosigkeit
Matricaria chamomilla L.	Asteraceae	s pop	MT	Kamille	vol	RES	p. 29	Schnupfen, Nebenhöhlenkatarrh
Matricaria chamomilla L.	Asteraceae	s pop	MT	Kamille	top	TEE	p. 29	Zahnschmerzen
Matricaria chamomilla L.	Asteraceae	s pop	MT	Kamille	syst	URO	p. 29	Nebenhodenentzzündungen
Matricaria chamomilla L.	Asteraceae	s pop	UB	Kamille	top	DER	p. 303	Wundbehandlung
Matricaria chamomilla L.	Asteraceae	s pop	UB	Kamille	top	DER	p. 303	Wundbehandlung
Matricaria chamomilla L.	Asteraceae	s pop	UB	Kamille	top	DER	p. 303	Wundbehandlung
Matricaria chamomilla L.	Asteraceae	s pop	UB	Kamille	syst	GAS	p. 146 / 164	akute Magenerkrankungen/ Sodbrennen, Gastritis, Magen- und Zwölffingerdarmgeschwür
Matricaria chamomilla L.	Asteraceae	sci	EMA	Kamille	top	DER	HMPC/278814/2010	Traditional herbal medicinal product used for adjuvant therapy of irritations of skin and mucosae in the anal and genital region, after serious conditions have been excluded by a medical doctor.
Matricaria chamomilla L.	Asteraceae	sci	EMA	Kamille	top	DER	/HMPC/55843/2011	Traditional herbal medicinal product (indication 3) for the treatment of minor ulcers and inflammations of the mouth and throat.
Matricaria chamomilla L.	Asteraceae	sci	EMA	Kamille	top	DER	/HMPC/55843/2011	Traditional herbal medicinal product (indication 4) used for adjuvant therapy of irritations of skin and mucosae in the anal and genital region, after serious conditions have been excluded by a medical doctor.
Matricaria chamomilla L.	Asteraceae	sci	EMA	Kamille	top	DER	/HMPC/55843/2011	Traditional herbal medicinal product (indication 5) used for the treatment of minor inflammation of the skin (sunburn) and superficial wounds and small boils (furuncles).
Matricaria chamomilla L.	Asteraceae	sci	EMA	Kamille	syst	GAS	/HMPC/55843/2011	Traditional herbal medicinal product (indication 1) used for the symptomatic treatment of minor gastro-intestinal complaints such as bloating and minor spasms
Matricaria chamomilla L.	Asteraceae	sci	EMA	Kamille	vol	RES	/HMPC/55843/2011	Traditional herbal medicinal product (indication 2) used for the relief of symptoms of common cold.
Matricaria chamomilla L.	Asteraceae	sci	ESCOP	Matricariae flos	top	RES	I p. 312	respiratory tract
Matricaria chamomilla L.	Asteraceae	sci	WI	Kamille	syst	GYN	p. 376	Menstruationsbeschwerden
Melissa officinalis L.	Lamiaceae	ant	DIOS	Melisse	syst	ANT	197	gutes Mittel gegen Skorpion und Spinnenstiche und Hundsbisse, durch den Genuß von (giftigen) Pilzen gepeinigt werden
Melissa officinalis L.	Lamiaceae	ant	DIOS	Melisse	top	ANT	197	gutes Mittel gegen Skorpion und Spinnenstiche und Hundsbisse
Melissa officinalis L.	Lamiaceae	ant	DIOS	Melisse	vol	ANT	197	gutes Mittel gegen Skorpion und Spinnenstiche und Hundsbisse
Melissa officinalis L.	Lamiaceae	ant	DIOS	Melisse	top	DER	197	zerteilen sie Drüsen am Halse und reinigen Geschwüre
Melissa officinalis L.	Lamiaceae	ant	DIOS	Melisse	syst	GAS	197	und gegen Leibschneiden
Melissa officinalis L.	Lamiaceae	ant	DIOS	Melisse	top	GAS	197	als Klistier bei Dysenterie
Melissa officinalis L.	Lamiaceae	ant	DIOS	Melisse	top	GYN	197	zur Beförderung der Katamenien
Melissa officinalis L.	Lamiaceae	ant	DIOS	Melisse	syst	RES	197	den an Orthopnöe Leidenden
Melissa officinalis L.	Lamiaceae	ant	DIOS	Melisse	top	SKE	197	Gelenkschmerzen besänftigen sie als Umschlag
Melissa officinalis L.	Lamiaceae	ant	DIOS	Melisse	top	TEE	197	als Mundspülwasser bei Zahnschmerzen
Melissa officinalis L.	Lamiaceae	mon	HvB	Melisse	top	EYE	1-59	Aber wenn das Weisse im Auge wächst
Melissa officinalis L.	Lamiaceae	mon	HvB	Melisse	syst	OTH	1-59	und ein Mensch der sie isst, lacht gern, weil ihre Wärme die Milz berührt und daher das Herz erfreut wird
Melissa officinalis L.	Lamiaceae	mon	MF	Melisse	syst	DER	50	reinigt fressende Geschwüre, falls sie eitern
Melissa officinalis L.	Lamiaceae	mon	MF	Melisse	top	DER	50	altverhärtete Beulen, Hamorhoiden
Melissa officinalis L.	Lamiaceae	mon	MF	Melisse	top	DER	50	Bienen-, Wespen- ,Spinnenstich
Melissa officinalis L.	Lamiaceae	mon	MF	Melisse	top	DER	50	Hundebiss
Melissa officinalis L.	Lamiaceae	mon	MF	Melisse	top	EYE	50	macht trübe Augen hellsichtig
Melissa officinalis L.	Lamiaceae	mon	MF	Melisse	syst	GAS	50	verjagt es schädliche Aufblähungen
Melissa officinalis L.	Lamiaceae	mon	MF	Melisse	syst	GAS	50	Blutstuhl und Bauchgrimmen leidenden
Melissa officinalis L.	Lamiaceae	mon	MF	Melisse	syst	GYN	50	Reinigung der Frau
Melissa officinalis L.	Lamiaceae	mon	MF	Melisse	syst	GYN	50	sorgt für geordneten Monatsfluss
Melissa officinalis L.	Lamiaceae	mon	MF	Melisse	syst	RES	50	Astmah und Atemnot
Melissa officinalis L.	Lamiaceae	mon	MF	Melisse	syst	SKE	50	lindert das Leiden aller Gelenke
Melissa officinalis L.	Lamiaceae	mon	MF	Melisse	top	TEE	50	pflegt den Zahnschmerz zu dämpfen
Melissa officinalis L.	Lamiaceae	ren	LF	Melisse	syst	ANT	CCLXXXII / CXC	wider die biss der schlangen, und anderen vergifften thier, so einer giftige schwaemme gessen hat
Melissa officinalis L.	Lamiaceae	ren	LF	Melisse	top	ANT	CCLXXXII / CXC	wider die biss der schlangen, und anderen vergifften thier
Melissa officinalis L.	Lamiaceae	ren	LF	Melisse	top	DER	CCLXXXII / CXC	vertreiben die kroepff, reyningen und heylen die wunden, lindern den schmerzen
Melissa officinalis L.	Lamiaceae	ren	LF	Melisse	top	EYE	CCLXXXII / CXC	klars gesicht
Melissa officinalis L.	Lamiaceae	ren	LF	Melisse	top	GAS	CCLXXXII / CXC	rote rhur
Melissa officinalis L.	Lamiaceae	ren	LF	Melisse	syst	GYN	CCLXXXII / CXC	verhindert das auffsteigen der muter
Melissa officinalis L.	Lamiaceae	ren	LF	Melisse	top	GYN	CCLXXXII / CXC	bringt den frawen ir zeit
Melissa officinalis L.	Lamiaceae	ren	LF	Melisse	syst	NER	CCLXXXII / CXC	denen die traurig sind, dann es macht froelich, sinnreich mache und gute leichte troeum
Melissa officinalis L.	Lamiaceae	ren	LF	Melisse	syst	RES	CCLXXXII / CXC	die den athemb nit mögen haben denn auffrecht
Melissa officinalis L.	Lamiaceae	ren	LF	Melisse	top	TEE	CCLXXXII / CXC	still den weetagen der zaen
Melissa officinalis L.	Lamiaceae	s pop	BVA	Zitronenmelisse	top	DER	p. 276	Fieberbläschen an Lippen und Mund
Melissa officinalis L.	Lamiaceae	s pop	BVA	Zitronenmelisse	syst	GAS	p. 276	Krämpfe im Darm und Unterleib, Brechreiz, Übelkeit (Schwangerschaft), nervöse Magenbeschwerden, Darmkatarrh, Appetitlosigkeit
Melissa officinalis L.	Lamiaceae	s pop	BVA	Zitronenmelisse	syst	GAS	p. 276	Blähungen mit Herzbescherweden
Melissa officinalis L.	Lamiaceae	s pop	BVA	Zitronenmelisse	syst	GYN	p. 276	schmerzhafte Periode,
Melissa officinalis L.	Lamiaceae	s pop	BVA	Zitronenmelisse	top	GYN	p. 276	Menstruationsbeschwerden

Melissa officinalis L.	Lamiaceae	s pop	BVA	Zitronenmelisse	syst	NER	p. 276	Nervosität, Herzneurosen, Herzklopfen,Schlaflosigkeit durch Reizüberflutung, nervösem Kopfweh,
Melissa officinalis L.	Lamiaceae	s pop	BVA	Zitronenmelisse	top	NER	p. 276	Nervenschmerzen, Kopfweh
Melissa officinalis L.	Lamiaceae	s pop	BVA	Zitronenmelisse	top	NER	p. 276	Nervosität, Schlaflsoigkeit, Verkrampfungen
Melissa officinalis L.	Lamiaceae	s pop	BVA	Zitronenmelisse	top	SKE	p. 276	Muskelkater, Rheuma
Melissa officinalis L.	Lamiaceae	s pop	BVA	Zitronenmelisse	syst	TON	p. 276	Stärkung, Erfrischung
Melissa officinalis L.	Lamiaceae	s pop	JK	Melisse	syst	CAR	p. 397	Herstärkung, Herzbeklemmung, Schwindelgefühle
Melissa officinalis L.	Lamiaceae	s pop	JK	Melisse	syst	CAR	p. 397	Ohnmachten, Herzgeschichten
Melissa officinalis L.	Lamiaceae	s pop	JK	Melisse	top	DER	p. 397	allerlei Hautunreinigkeiten
Melissa officinalis L.	Lamiaceae	s pop	JK	Melisse	syst	GAS	p. 397	Übelkeit
Melissa officinalis L.	Lamiaceae	s pop	JK	Melisse	syst	NER	p. 397	Schmermut, Migräne
Melissa officinalis L.	Lamiaceae	s pop	UB	Melisse	top	DER	p. 350	Herpes labialis, Herpes simplex
Melissa officinalis L.	Lamiaceae	s pop	UB	Melisse	syst	GAS	p. 147	aktue Magenbeschwerden
Melissa officinalis L.	Lamiaceae	s pop	UB	Melisse	syst	GAS	p. 147	aktue Magenbeschwerden
Melissa officinalis L.	Lamiaceae	s pop	UB	Melisse	syst	GAS	p. 147	aktue Magenbeschwerden
Melissa officinalis L.	Lamiaceae	s pop	UB	Melisse	syst	GAS	p. 147	aktue Magenbeschwerden
Melissa officinalis L.	Lamiaceae	s pop	UB	Melisse	syst	NER	p. 422	Spasmolytikum und Beruhigungsmittel
Melissa officinalis L.	Lamiaceae	s pop	UB	Melisse	top	NER	p. 422	Spasmolytikum und Beruhigungsmittel
Melissa officinalis L.	Lamiaceae	s pop	WI	Melisse	syst	CAR	p. 384	Volksmedizin: funktioneller Kreislaufschwäche
Melissa officinalis L.	Lamiaceae	s pop	WI	Melisse	syst	RES	p. 384	Volksmedizin: Erkältungskrankheiten
Melissa officinalis L.	Lamiaceae	sci	EMA	Melisse	syst	GAS	HMPC/196745/2012	Traditional herbal medicinal product for symptomatic treatment of mild gastrointestinal complaints including bloating and flatulence.
Melissa officinalis L.	Lamiaceae	sci	EMA	Melisse	syst	GAS	HMPC/196745/2012	Traditional herbal medicinal product for symptomatic treatment of mild gastrointestinal complaints including bloating and flatulence.
Melissa officinalis L.	Lamiaceae	sci	EMA	Melisse	syst	GAS	HMPC/196745/2012	Traditional herbal medicinal product for symptomatic treatment of mild gastrointestinal complaints including bloating and flatulence.
Melissa officinalis L.	Lamiaceae	sci	EMA	Melisse	syst	NER	HMPC/196745/2012	Traditional herbal medicinal product for relief of mild symptoms of mental stress and to aid sleep.
Melissa officinalis L.	Lamiaceae	sci	EMA	Melisse	syst	NER	HMPC/196745/2012	Traditional herbal medicinal product for relief of mild symptoms of mental stress and to aid sleep.
Melissa officinalis L.	Lamiaceae	sci	EMA	Melisse	syst	NER	HMPC/196745/2012	Traditional herbal medicinal product for relief of mild symptoms of mental stress and to aid sleep.
Melissa officinalis L.	Lamiaceae	sci	ESCOP	Melissa folium	top	DER	I p. 324	Herpes labialis
Mentha pulegium L.	Lamiaceae	ant	DIOS	Poleiminze	syst	ANT	176	hilft es mit Wein getrunken denen, die von giftigen Tieren gebissen
Mentha pulegium L.	Lamiaceae	ant	DIOS	Poleiminze	vol	CAR	176	Ohnmächtigen richtet es auf
Mentha pulegium L.	Lamiaceae	ant	DIOS	Poleiminze	top	DER	176	besänftigt es jede Entzündung; bringt es Finnen weg
Mentha pulegium L.	Lamiaceae	ant	DIOS	Poleiminze	top	DER	176	fein fein gerieben und gebrannt macht es das Zahnfleisch fest
Mentha pulegium L.	Lamiaceae	ant	DIOS	Poleiminze	top	DER	176	zum Bade verwandt lindert Juckreiz
Mentha pulegium L.	Lamiaceae	ant	DIOS	Poleiminze	syst	GAS	176	stillt es Übelkeit und Magenschmerzen
Mentha pulegium L.	Lamiaceae	ant	DIOS	Poleiminze	syst	GYN	176	Getrunken befördert es die Menstruation, treibt die Nachgeburt und die Leibesfrucht aus
Mentha pulegium L.	Lamiaceae	ant	DIOS	Poleiminze	top	GYN	176	als Sitzbad ist sie ein gutes Mittel bei Aufblähung, Verhärtung und Umwendung der Gebärmutter.
Mentha pulegium L.	Lamiaceae	ant	DIOS	Poleiminze	syst	NER	176	hilft bei Krämpfen
Mentha pulegium L.	Lamiaceae	ant	DIOS	Poleiminze	top	OTH	176	Mit Salz umgeschlagen ist es Milzkranken heilsam
Mentha pulegium L.	Lamiaceae	ant	DIOS	Poleiminze	syst	RES	176	reinigt es die Lunge
Mentha pulegium L.	Lamiaceae	ant	DIOS	Poleiminze	top	SKE	176	Podagra
Mentha pulegium L.	Lamiaceae	mon	HvB	Poleiminze	syst	EYE	1-126	und erhellt deine Augen
Mentha pulegium L.	Lamiaceae	mon	HvB	Poleiminze	top	EYE	1-126	und wem die Augen sich verdunkeln
Mentha pulegium L.	Lamiaceae	mon	HvB	Poleiminze	syst	GAS	1-126	es reinigt den Magen; der wärmt den Magen, wenn er einen kalten Magen hat; Und auch wenn sein Magen voll von Gift ist, das ist Eiter, reingt und heilt es ihn
Mentha pulegium L.	Lamiaceae	mon	HvB	Poleiminze	top	NER	1-126	und wer im Gehirn Schmerzen hat, so dass er krank ist, der lege Polei . . . .und der Wahnsinn in ihm wird unterdrückt.
Mentha pulegium L.	Lamiaceae	mon	MF	Poleiminze	syst	ANT	16	widersetzt sich den vergifteten Bissen von Schlangen
Mentha pulegium L.	Lamiaceae	mon	MF	Poleiminze	syst	APH	16	erregt Liebeskraft
Mentha pulegium L.	Lamiaceae	mon	MF	Poleiminze	vol	CAR	16	Ohnmacht (Fieber, heftiger Blutfluss)
Mentha pulegium L.	Lamiaceae	mon	MF	Poleiminze	top	DER	16	stärkt und verfestigt man das Zahnfleisch
Mentha pulegium L.	Lamiaceae	mon	MF	Poleiminze	top	DER	16	wird nicht am Jucken leiden
Mentha pulegium L.	Lamiaceae	mon	MF	Poleiminze	top	DER	16	trennt es den Grind vom Leib
Mentha pulegium L.	Lamiaceae	mon	MF	Poleiminze	top	DER	16	beseitigt sämtliche Geschwülste
Mentha pulegium L.	Lamiaceae	mon	MF	Poleiminze	syst	GAS	16	Brechreiz oder beissender Magenschmerz
Mentha pulegium L.	Lamiaceae	mon	MF	Poleiminze	syst	GYN	16	treibt es fast immer die Leibesfrucht ab, sorgt für geordneten Monatsfluss, zieht eine zögerliche Nachgeburt heraus, gewährt Muttermilch im Überfluss
Mentha pulegium L.	Lamiaceae	mon	MF	Poleiminze	syst	RES	16	zähklebrige Säfte in der Brust verdünnen
Mentha pulegium L.	Lamiaceae	mon	MF	Poleiminze	top	SKE	16	verkrüppelten oder verkrampften Gliedern helfen
Mentha pulegium L.	Lamiaceae	mon	MF	Poleiminze	top	SKE	16	lindert Fussgicht
Mentha pulegium L.	Lamiaceae	mon	MF	Poleiminze	top	URO	16	reichlich Harn zu lassen
Mentha pulegium L.	Lamiaceae	ren	LF	Poleiminze	syst	ANT	CX / LXXIII	von den giftigen thiern gebissen seind
Mentha pulegium L.	Lamiaceae	ren	LF	Poleiminze	vol	CAR	CX / LXXIII	vor die nasen gehalten bringt herwider die so in onmacht fallen
Mentha pulegium L.	Lamiaceae	ren	LF	Poleiminze	top	DER	CX / LXXIII	miltert er allerley brand
Mentha pulegium L.	Lamiaceae	ren	LF	Poleiminze	top	DER	CX / LXXIII	sterckt das zanfleysch
Mentha pulegium L.	Lamiaceae	ren	LF	Poleiminze	top	DER	CX / LXXIII	legt das jucken der glider
Mentha pulegium L.	Lamiaceae	ren	LF	Poleiminze	syst	GAS	CX / LXXIII	unwillen und das grimmen und nagen im magen schlund
Mentha pulegium L.	Lamiaceae	ren	LF	Poleiminze	syst	GYN	CX / LXXIII	bringt den frawen ir krankheyt, tribt aus das bürdlin und die geburt
Mentha pulegium L.	Lamiaceae	ren	LF	Poleiminze	top	GYN	CX / LXXIII	blaeen, erte und den krampff der muter
Mentha pulegium L.	Lamiaceae	ren	LF	Poleiminze	syst	NER	CX / LXXIII	fallende sucht
Mentha pulegium L.	Lamiaceae	ren	LF	Poleiminze	top	NER	CX / LXXIII	weetagen des haupt und den schwindel
Mentha pulegium L.	Lamiaceae	ren	LF	Poleiminze	vol	NER	CX / LXXIII	so ein kalt und feucht hirn haben
Mentha pulegium L.	Lamiaceae	ren	LF	Poleiminze	n.d.	OTH	CX / LXXIII	ungesund wasser trinken muss
Mentha pulegium L.	Lamiaceae	ren	LF	Poleiminze	top	OTH	CX / LXXIII	bekommt es wohl dem milzsüchtigen
Mentha pulegium L.	Lamiaceae	ren	LF	Poleiminze	syst	RES	CX / LXXIII	reiniget er die lungen
Mentha pulegium L.	Lamiaceae	ren	LF	Poleiminze	top	SKE	CX / LXXIII	so den krampff haben, wenn sie sich damit reiben lassen; bekommt auch trefflich wohl dem hüftwee
Mentha pulegium L.	Lamiaceae	ren	LF	Poleiminze	top	SKE	CX / LXXIII	podagrische glieder
Mentha pulegium L.	Lamiaceae	ren	LF	Poleiminze	syst	URO	CX / LXXIII	treibt den harn und den stein der nieren
Mentha pulegium L.	Lamiaceae	s pop	VAL	Poleiminze	syst	GAS	p. 428	insuffisance biliaire, atonie gastrique
Mentha pulegium L.	Lamiaceae	s pop	VAL	Poleiminze	syst	RES	p. 428	bronchites, coqueluche
Mentha spicata agg.	Lamiaceae	ant	DIOS	Minze	top	ANT	177	Kataplasma gegen Hundsbiß
Mentha spicata agg.	Lamiaceae	ant	DIOS	Minze	syst	APH	177	reizt zum Liebesgenuß
Mentha spicata agg.	Lamiaceae	ant	DIOS	Minze	top	DER	147	gegen Wespen- und Bienenstiche
Mentha spicata agg.	Lamiaceae	ant	DIOS	Minze	top	DER	177	zerteilt er Abszesse
Mentha spicata agg.	Lamiaceae	ant	DIOS	Minze	top	EAR	177	Honigmeth stillt Ohrenschmerzen
Mentha spicata agg.	Lamiaceae	ant	DIOS	Minze	syst	GAS	147	stillen sie auch das Erbrechen
Mentha spicata agg.	Lamiaceae	ant	DIOS	Minze	syst	GAS	177	Überhaupt ist er dem Magen zuträglich und eine gute Würze
Mentha spicata agg.	Lamiaceae	ant	DIOS	Minze	syst	GAS	177	stellt der Saft mit Essig getrunken den Blutauswurf
Mentha spicata agg.	Lamiaceae	ant	DIOS	Minze	syst	GAS	177	tötet ferner die runden Würmer
Mentha spicata agg.	Lamiaceae	ant	DIOS	Minze	syst	GAS	177	bringt das Schlucken, den Brechreiz und die Cholera zur Ruhe,
Mentha spicata agg.	Lamiaceae	ant	DIOS	Minze	top	GYN	177	besänftigt geschwollene und (von Milch) strotzende Brüste
Mentha spicata agg.	Lamiaceae	ant	DIOS	Minze	top	GYN	177	den Weibern aber vor dem Beischlaf im Zäpfchen eingelegt behindert er die Empfängnis
Mentha spicata agg.	Lamiaceae	ant	DIOS	Minze	top	NER	147	gegen Kopfleiden auf die Schläfen und das Gesicht gelegt
Mentha spicata agg.	Lamiaceae	ant	DIOS	Minze	syst	NER	147	er beruhigt auch Krämpfe und Schlucken
Mentha spicata agg.	Lamiaceae	ant	DIOS	Minze	top	NER	177	auf die Stirn gelegt lindert er Kopfschmerzen
Mentha spicata agg.	Lamiaceae	ant	DIOS	Minze	syst	URO	147	ist bei Harnzwang und Blasenstein
Mentha spicata agg.	Lamiaceae	mon	HvB	Wilde Minze	top	DER	1-76	Krätzmilben
Mentha spicata agg.	Lamiaceae	mon	HvB	Ackerminze	top	EYE	1-77	Augengeschwür
Mentha spicata agg.	Lamiaceae	mon	HvB	Bachminze	syst	GAS	1-75	Magen von vielen Speisen beschwert
Mentha spicata agg.	Lamiaceae	mon	HvB	Krauseminze	syst	GAS	1-78	kalter Magen
Mentha spicata agg.	Lamiaceae	mon	HvB	Ackerminze	top	GAS	1-77	kalter Magen
Mentha spicata agg.	Lamiaceae	mon	HvB	Bachminze	syst	RES	1-75	kranke Lunge, nur schwer atmet

Mentha spicata agg.	Lamiaceae	mon	HvB	Krauseminze	syst	SKE	1-78	Gicht
Mentha spicata agg.	Lamiaceae	mon	MF	Minze	top	DER	47	Hundebiss, so heilt sie ihn
Mentha spicata agg.	Lamiaceae	mon	MF	Minze	top	DER	47	rauhe Zunge wird glatt
Mentha spicata agg.	Lamiaceae	mon	MF	Minze	top	EAR	47	Ohr, vretreiben den Schmerz
Mentha spicata agg.	Lamiaceae	mon	MF	Minze	syst	GAS	47	fördert die Verdauungskraft, stärkt den Magen, hält den Brechreiz fern und soll die Spulwürmer vertreiben
Mentha spicata agg.	Lamiaceae	mon	MF	Minze	syst	GAS	47	verhilft sie den Blutspeienden
Mentha spicata agg.	Lamiaceae	mon	MF	Minze	syst	GYN	47	Kindsgeburt zu beschleunigen
Mentha spicata agg.	Lamiaceae	mon	MF	Minze	top	GYN	47	. . . bevor ein Beischlaf stattfindet, kann die Frau dabei nicht empfangen
Mentha spicata agg.	Lamiaceae	mon	MF	Minze	top	GYN	47	stockt die Milch in den Brüsten
Mentha spicata agg.	Lamiaceae	mon	MF	Minze	syst	URO	47	verschiedene Leiden der Hoden
Mentha spicata agg.	Lamiaceae	ren	LF	Minze	syst	APH	CLXI - CLXIIII / CX	reizt zur unkeuschheyt
Mentha spicata agg.	Lamiaceae	ren	LF	Minze	top	DER	CLXI - CLXIIII / CX	zerteylen sie die geschwulst
Mentha spicata agg.	Lamiaceae	ren	LF	Minze	top	DER	CLXI - CLXIIII / CX	vertreiben sie der rauhe der zunge
Mentha spicata agg.	Lamiaceae	ren	LF	Minze	top	DER	CLXI - CLXIIII / CX	heylen die geschwaer am kopff der jungen kinder und die flechten
Mentha spicata agg.	Lamiaceae	ren	LF	Minze	top	EAR	CLXI - CLXIIII / CX	benimpt den weetagen
Mentha spicata agg.	Lamiaceae	ren	LF	Minze	n.d.	GAS	CLXI - CLXIIII / CX	dienstlich der kalten leber, stecken den magen und die dewung
Mentha spicata agg.	Lamiaceae	ren	LF	Minze	syst	GAS	CLXI - CLXIIII / CX	macht begierd zu essen
Mentha spicata agg.	Lamiaceae	ren	LF	Minze	syst	GAS	CLXI - CLXIIII / CX	vertreiben sie das grimmen und weetagen der daerm
Mentha spicata agg.	Lamiaceae	ren	LF	Minze	syst	GAS	CLXI - CLXIIII / CX	hergen und würgen / Choleram genannt das ist so unden und oben die gall von einem geet / ser dienstlich dem magen
Mentha spicata agg.	Lamiaceae	ren	LF	Minze	syst	GAS	CLXI - CLXIIII / CX	toedtet die runden wuerm
Mentha spicata agg.	Lamiaceae	ren	LF	Minze	syst	GYN	CLXI - CLXIIII / CX	stellen auch der frawen krankheyt
Mentha spicata agg.	Lamiaceae	ren	LF	Minze	syst	GYN	CLXI - CLXIIII / CX	kommen sie zuhilff den frawen so schwaer gebaeren
Mentha spicata agg.	Lamiaceae	ren	LF	Minze	top	GYN	CLXI - CLXIIII / CX	vertreiben die milch
Mentha spicata agg.	Lamiaceae	ren	LF	Minze	top	NER	CLXI - CLXIIII / CX	mildern sie das hauptwee
Mentha spicata agg.	Lamiaceae	ren	LF	Minze	vol	NER	CLXI - CLXIIII / CX	macht mut und froelich
Mentha spicata agg.	Lamiaceae	ren	LF	Minze	syst	OTH	CLXI - CLXIIII / CX	stellt das blut
Mentha spicata agg.	Lamiaceae	ren	LF	Minze	syst	RES	CLXI - CLXIIII / CX	stillen sie das blutspeien
Mentha spicata agg.	Lamiaceae	s pop	BVA	Pfefferminze	syst	CAR	p. 164	Reisekrankheit
Mentha spicata agg.	Lamiaceae	s pop	BVA	Pfefferminze	top	DER	p. 164	Hautjucken, Mundspülung, wunde Zunge
Mentha spicata agg.	Lamiaceae	s pop	BVA	Pfefferminze	syst	GAS	p. 164	ungenügende Gallenabsonderung, Reisebeschwerden, Übelkeit, Erbrechen, Krämpfen im Unterleib und Verdauungsorganen, Magenverstimmung, Übersäuerungdes Magens, Appetitlosigkeit
Mentha spicata agg.	Lamiaceae	s pop	BVA	Pfefferminze	syst	GYN	p. 164	Regelbeschwerden
Mentha spicata agg.	Lamiaceae	s pop	BVA	Pfefferminze	top	NER	p. 164	Neuralgien, Kopfweh
Mentha spicata agg.	Lamiaceae	s pop	JK	Minzen	syst	CAR	p. 398	stärkt Herz
Mentha spicata agg.	Lamiaceae	s pop	JK	Minzen	syst	CAR	p. 398	Herzanfällen
Mentha spicata agg.	Lamiaceae	s pop	JK	Minzen	syst	GAS	p. 398	stärkt Magen
Mentha spicata agg.	Lamiaceae	s pop	JK	Minzen	syst	GAS	p. 398	Kolik
Mentha spicata agg.	Lamiaceae	s pop	JK	Minzen	top	HUM	p. 398	Fussschweiss wecken
Mentha spicata agg.	Lamiaceae	s pop	JK	Minzen	syst	NER	p. 398	Schwindel, Bewusstlosigkeit, Migräne
Mentha spicata agg.	Lamiaceae	s pop	JK	Minzen	syst	RES	p. 398	Keuchen und schwerer Atem
Mentha spicata agg.	Lamiaceae	s pop	JK	Minzen	syst	RES	p. 398	Unwohlsein und Übelkeit
Mentha spicata agg.	Lamiaceae	s pop	JK	Minzen	top	RES	p. 398	nehmen Schnupfen und Kopfweh
Mentha spicata agg.	Lamiaceae	s pop	JK	Minzen	syst	URO	p. 398	fördert den Wasserabgang
Mentha spicata agg.	Lamiaceae	s pop	JK	Minzen	top	URO	p. 398	Leute, die den Urin nicht halten können
Mentha spicata agg.	Lamiaceae	s pop	KN	Minze	syst	GAS	p. 479	Koilken und blähungstreibendes Mittel
Mentha spicata agg.	Lamiaceae	s pop	KN	Minze	syst	GAS	p. 479	Magen stärken und Verdauung fördern; Leber- Gallestörungen; fördert die Verdauung
Mentha spicata agg.	Lamiaceae	s pop	KN	Minze	syst	GAS	p. 479	übel- und faulriechenden Atem
Mentha spicata agg.	Lamiaceae	s pop	KN	Minze	syst	GAS	p. 479	Blutbrechen
Mentha spicata agg.	Lamiaceae	s pop	KN	Minze	syst	GYN	p. 479	Unterleibsschmerzen
Mentha spicata agg.	Lamiaceae	s pop	KN	Minze	top	NER	p. 479	bei Kofpschmerzen und örtlichen Schmerzen und Neuralgien
Mentha spicata agg.	Lamiaceae	s pop	KN	Minze	top	NER	p. 479	bei heftigem Kopfweh auf die Stinr bindet
Mentha spicata agg.	Lamiaceae	s pop	KN	Minze	syst	TON	p. 479	durch Krankheit sehr Geschwächte, bei jeder Kleinigkeit von Herzklopfen Befallene, viel an Übelkeit und heftigem Erbrechen Leidende
Mentha spicata agg.	Lamiaceae	s pop	UB	Pfefferminze	syst	GAS	p. 116 / 147 / 184	Karminativa, Blähungen / akute Magenerkrankungen / Gallenstörungen
Mentha spicata agg.	Lamiaceae	s pop	UB	Pfefferminze	top	NER	p. 441	Migräne, Kopfschmerzen
Mentha spicata agg.	Lamiaceae	s pop	WI	Krauseminze	syst	NER	p. 392	Volksheilkunde: Sedativum / Nerventees
Mentha spicata agg.	Lamiaceae	sci	EMA	Pfefferminze	syst	GAS	HMPC/349466/2006	Oromucosal use: 5. For the relief of symptoms in coughs and colds
Mentha spicata agg.	Lamiaceae	sci	EMA	Pfefferminze	top	GAS	HMPC/349466/2006	Cutaneous and transdermal use: 1. For the relief of symptoms in coughs and
Mentha spicata agg.	Lamiaceae	sci	EMA	Pfefferminze	syst	GAS	HMPC/193909/2007	Traditional herbal medicinal product for the symptomatic relief of digestive disorders such as dyspepsia and flatulence.
Mentha spicata agg.	Lamiaceae	sci	EMA	Pfefferminze	syst	GAS	HMPC/193909/2007	Traditional herbal medicinal product for the symptomatic relief of digestive disorders such as dyspepsia and flatulence.
Mentha spicata agg.	Lamiaceae	sci	EMA	Pfefferminze	vol	RES	HMPC/349466/2006	Inhalation: 4. For the relief of symptoms in coughs and colds
Mentha spicata agg.	Lamiaceae	sci	ESCOP	Menthae Piperitae A	top	DER	I p. 329	pruritus, urticariaand pain in irritable skin conditions
Mentha spicata agg.	Lamiaceae	sci	ESCOP	Menthae Piperitae A	top	NER	I p. 329	tension-type headache
Mentha spicata agg.	Lamiaceae	sci	ESCOP	Menthae Piperitae A	syst	RES	I p. 329	symptomatic treatment of coughs and colds
Mentha spicata agg.	Lamiaceae	sci	ESCOP	Menthae Piperitae A	top	RES	I p. 329	relief of coougHs and colds
Mentha spicata agg.	Lamiaceae	sci	ESCOP	Menthae Piperitae A	top	SKE	I p. 329	symptomatic relief of rheumatic complaints
Mercurialis spp. (*M. annua*, *M. perennis*)	Euphorbiacea	ant	DIOS	Bingelkraut	syst	GAS	262	werden sie in Wasser gekocht, so führt dieses als Trank Galle und Wässeriges ab.
Mercurialis spp. (*M. annua*, *M. perennis*)	Euphorbiacea	ant	DIOS	Bingelkraut	syst	GYN	262	als ob die Blätter der weiblichen, fein gestossen und getrunken oder nach der Menstruation im Zäpfchen eingelegt, die Empfängniss eines Mädchens, die der männlichen, in derselben Weise angewandt, die Erzeugung eines Knaben bewirken
Mercurialis spp. (*M. annua*, *M. perennis*)	Euphorbiacea	mon	LO	Einjähriges Bingelkr	n.d.	OTH	n.d.	n.d.
Mercurialis spp. (*M. annua*, *M. perennis*)	Euphorbiacea	ren	LF	Einjähriges Bingelkr	top	DER	- CCLXVIII / CLXXX	verzeren und zerteylen knollen und geschwulst
Mercurialis spp. (*M. annua*, *M. perennis*)	Euphorbiacea	ren	LF	Einjähriges Bingelkr	top	EAR	- CCLXVIII / CLXXX	bringt das gehoer
Mercurialis spp. (*M. annua*, *M. perennis*)	Euphorbiacea	ren	LF	Einjähriges Bingelkr	syst	GAS	CCXLIX / CLXVIII	lindern den stulgang, purgieren
Mercurialis spp. (*M. annua*, *M. perennis*)	Euphorbiacea	ren	LF	Bingelkraut	syst	GAS	CCXLIX / CLXVIII	lindern den stulgang, purgieren
Mercurialis spp. (*M. annua*, *M. perennis*)	Euphorbiacea	ren	LF	Einjähriges Bingelkr	top	GYN	- CCLXVIII / CLXXX	bringen sie den frawen ir zeyt, treiben das nachbürdlin
Mercurialis spp. (*M. annua*, *M. perennis*)	Euphorbiacea	ren	LF	Einjähriges Bingelkr	top	URO	- CCLXVIII / CLXXX	treiben den harn und seind der blasen nützlich
Mercurialis spp. (*M. annua*, *M. perennis*)	Euphorbiacea	s pop	VAL	Einjähriges Bingelkr	syst	GAS	p. 366	constipation
Mercurialis spp. (*M. annua*, *M. perennis*)	Euphorbiacea	s pop	VAL	Einjähriges Bingelkr	syst	GYN	p. 366	supression de la sécrétion lactée
Meum athamanticum Jacq.	Apiaceae	ant	DIOS	Bärwurz	syst	GAS	69	gegen Aufblähen des Magens und Leibschneiden
Meum athamanticum Jacq.	Apiaceae	ant	DIOS	Bärwurz	syst	GYN	69	leiten sie das Blut ab durch die Menstruation
Meum athamanticum Jacq.	Apiaceae	ant	DIOS	Bärenwurz	syst	NER	69	auch bei hysterischen Zuständen
Meum athamanticum Jacq.	Apiaceae	ant	DIOS	Bärenwurz	syst	SKE	69	Gelenkleiden
Meum athamanticum Jacq.	Apiaceae	ant	DIOS	Bärenwurz	syst	SKE	69	dienen sie gegen Brustrheumatismus
Meum athamanticum Jacq.	Apiaceae	ant	DIOS	Bärenwurz	syst	URO	69	lindern die Schmerzen bei Blasen- und Nierenverstopfung und sind ein gutes Mittel gegen Harnverhaltung
Meum athamanticum Jacq.	Apiaceae	ant	DIOS	Bärenwurz	top	URO	69	Auf die Schamteile der Kinder gelegt, treiben sie den Harn.
Meum athamanticum Jacq.	Apiaceae	mon	HvB	Bärenfenchel	syst	FEV	1-135	der starke und hitzige Fieber in sich hat
Meum athamanticum Jacq.	Apiaceae	mon	HvB	Bärenfenchel	syst	GAS	1-135	Und wer Gelbsucht hat
Meum athamanticum Jacq.	Apiaceae	mon	HvB	Bärenwurz	syst	SKE	1-135	auch wer Gicht in ihm hat
Meum athamanticum Jacq.	Apiaceae	ren	LF	Bärenwurz	syst	ANT	CCCCLI / CCCV	bewerte artzney für allerley gifft
Meum athamanticum Jacq.	Apiaceae	ren	LF	Bärenwurz	syst	GYN	CCCCLI / CCCV	und bringt den frawen ire zeit
Meum athamanticum Jacq.	Apiaceae	ren	LF	Bärenwurz	syst	GYN	CCCCLI / CCCV	trefflich gut den weibern die den weissen fluss haben
Meum athamanticum Jacq.	Apiaceae	ren	LF	Bärenwurz	syst	RES	CCCCLI / CCCV	fuerret auss den kalten zaehen schleim der lungen unnd brust
Meum athamanticum Jacq.	Apiaceae	ren	LF	Bärenwurz	syst	URO	CCCCLI / CCCV	heylt die gebresten der nieren
Meum athamanticum Jacq.	Apiaceae	ren	LF	Bärenwurz	syst	URO	CCCCLI / CCCV	gut zu den harnwinden
Meum athamanticum Jacq.	Apiaceae	ren	LF	Bärenwurz	syst	URO	CCCCLI / CCCV	treiben den stein
Meum athamanticum Jacq.	Apiaceae	s pop	JK	Bärenwurz	syst	GAS	p. 400	wirkt wärmend und entschleimend auf den Magen
Morus nigra L.	Moraceae	ant	DIOS	Maulbeere	syst	ANT	112	hilft denen, die Sturmhut genossen haben
Morus nigra L.	Moraceae	ant	DIOS	Maulbeere	syst	ANT	112	hilft denen, die von der Spinne gebissen
Morus nigra L.	Moraceae	ant	DIOS	Maulbeere	syst	DER	112	kriechende Geschwüre und Mandelentzündungen
Morus nigra L.	Moraceae	ant	DIOS	Maulbeere	top	DER	112	heilen Verbrennungen

Morus nigra L.	Moraceae	ant	DIOS	Maulbeere	syst	DER	112	Geschwüre öffnet
Morus nigra L.	Moraceae	ant	DIOS	Maulbeere	syst	GAS	112	wirkt er gegen Flüsse
Morus nigra L.	Moraceae	ant	DIOS	Maulbeere	syst	GAS	112	Magenkranken
Morus nigra L.	Moraceae	ant	DIOS	Maulbeere	syst	GAS	112	löst den Bauch, treibt den Bandwurm
Morus nigra L.	Moraceae	ant	DIOS	Maulbeere	syst	GAS	112	den Bauch reinigt
Morus nigra L.	Moraceae	ant	DIOS	Maulbeere	top	OTH	112	färben sie die Haare
Morus nigra L.	Moraceae	ant	DIOS	Maulbeere	top	TEE	112	als Mundwasser bei Zahnschmerzen
Morus nigra L.	Moraceae	ant	DIOS	Maulbeere	syst	TEE	112	welche gegen Zahnschmerzen wirksam
Morus nigra L.	Moraceae	mon	HvB	Maulbeerbaum	syst	ANT	3-9	Und wer Gift durch Essen oder Trinken eingenommen hat
Morus nigra L.	Moraceae	mon	HvB	Maulbeerbaum	top	DER	3-9	und wer die Krätze hat
Morus nigra L.	Moraceae	mon	HvB	Maulbeerbaum	syst	OTH	3-9	Und es ist Üppigkeit in seiner Frucht, ... Aber sie nützt dem Menschen mehr als sie ihm schadet
Morus nigra L.	Moraceae	ren	LF	Maulbeere	syst	DER	CCXCV / CXCVIII	treibt hinder sich die flüss und geschwulst der mandeln, er ist auch nütz zu anderen gebresten des munds
Morus nigra L.	Moraceae	ren	LF	Maulbeere	top	DER	CCXCV / CXCVIII	heilen den brand
Morus nigra L.	Moraceae	ren	LF	Maulbeere	syst	GAS	CCXCV / CXCVIII	lindert den Bauch, stellen den Bauchfluss
Morus nigra L.	Moraceae	ren	LF	Maulbeere	syst	GAS	CCXCV / CXCVIII	lindert den Bauch
Morus nigra L.	Moraceae	ren	LF	Maulbeere	syst	OTH	CCXCV / CXCVIII	treibt auch aus . . . Die breyten würm des bauchs
Morus nigra L.	Moraceae	ren	LF	Maulbeere	top	TEE	CCXCV / CXCVIII	legt das zanwee
Morus nigra L.	Moraceae	s pop	VAL	Maulbeere	syst	OTH	p. 378	diabète
Morus nigra L.	Moraceae	s pop	VAL	Maulbeere	top	RES	p. 378	gargarisme dans les angines, et en bains de bouche dans la stomatites et aphtes
Ocimum basilicum L.	Lamiaceae	ant	DIOS	Basilikum	top	ANT	150	den Biss des Meerdrachen und des Skorpions
Ocimum basilicum L.	Lamiaceae	ant	DIOS	Basilikum	syst	EYE	150	dient es gegen Augenleiden
Ocimum basilicum L.	Lamiaceae	ant	DIOS	Basilikum	top	EYE	150	Sein Saft entfernt Nebelflecken auf den Augen und trocknet die Flüsse
Ocimum basilicum L.	Lamiaceae	ant	DIOS	Basilikum	syst	GAS	150	es erweicht den Bauch, treibt die Winde
Ocimum basilicum L.	Lamiaceae	ant	DIOS	Basilikum	syst	GAS	150	Blähungen
Ocimum basilicum L.	Lamiaceae	ant	DIOS	Basilikum	syst	GYN	150	befördert die Milchabsonderung
Ocimum basilicum L.	Lamiaceae	ant	DIOS	Basilikum	syst	NER	150	gutes Mittel bei Melancholie
Ocimum basilicum L.	Lamiaceae	ant	DIOS	Basilikum	top	OTH	150	verursacht er vieles Niesen
Ocimum basilicum L.	Lamiaceae	ant	DIOS	Basilikum	top	RES	150	heilt es Lungenentzündung
Ocimum basilicum L.	Lamiaceae	ant	DIOS	Basilikum	syst	URO	150	treibt [die Winde und] den Harn
Ocimum basilicum L.	Lamiaceae	ant	DIOS	Basilikum	syst	URO	150	Harnverhaltung
Ocimum basilicum L.	Lamiaceae	mon	HvB	Basilikum	syst	FEV	1-230	wer starke Fieber hat
Ocimum basilicum L.	Lamiaceae	mon	HvB	Basilikum	top	SKE	1-230	Lähmung an seiner Zunge, dass er nicht Sprechen kann
Ocimum basilicum L.	Lamiaceae	ren	LF	Basilikum	top	ANT	VIII - CCCX / CCVIII	trefflich gut so denen von einem scorpion gestochen seind
Ocimum basilicum L.	Lamiaceae	ren	LF	Basilikum	vol	CAR	VIII - CCCX / CCVIII	bring er herwider die so in onmacht liegen
Ocimum basilicum L.	Lamiaceae	ren	LF	Basilikum	top	DER	VIII - CCCX / CCVIII	verzert und zeitigt auch allerley geschwulst
Ocimum basilicum L.	Lamiaceae	ren	LF	Basilikum	top	EYE	VIII - CCCX / CCVIII	leschen alle hitz und weetagen der augen, saft in die augen gethon vertreibt die dunkelheyt
Ocimum basilicum L.	Lamiaceae	ren	LF	Basilikum	syst	GAS	VIII - CCCX / CCVIII	weychen den bauch, machen blaest, werden im magen leichtlich verweandelt
Ocimum basilicum L.	Lamiaceae	ren	LF	Basilikum	syst	GYN	VIII - CCCX / CCVIII	bringen Milch
Ocimum basilicum L.	Lamiaceae	ren	LF	Basilikum	syst	GYN	VIII - CCCX / CCVIII	fürdert die die zeit der frawen
Ocimum basilicum L.	Lamiaceae	ren	LF	Basilikum	n.d.	NER	VIII - CCCX / CCVIII	sterckt das haupt und das herz
Ocimum basilicum L.	Lamiaceae	ren	LF	Basilikum	n.d.	NER	VIII - CCCX / CCVIII	macht freud denen so von der Schwarzen galle in traurigkeyt fallen
Ocimum basilicum L.	Lamiaceae	ren	LF	Basilikum	syst	URO	VIII - CCCX / CCVIII	treiben den harn
Ocimum basilicum L.	Lamiaceae	ren	LF	Basilikum	syst	URO	VIII - CCCX / CCVIII	treibt den harn
Ocimum basilicum L.	Lamiaceae	s pop	WI	Basilikum	top	DER	p. 105	Volksmedizin: Entzündungen des Rachenraums
Ocimum basilicum L.	Lamiaceae	s pop	WI	Basilikum	top	DER	p. 105	Volksmedizin: schlecht heilende Wunden
Ocimum basilicum L.	Lamiaceae	s pop	WI	Basilikum	syst	GAS	p. 105	Volksmedizin: Stomachikum bei Appetitlosigkeit,
Ocimum basilicum L.	Lamiaceae	s pop	WI	Basilikum	syst	GYN	p. 105	Volksmedizin: Galaktagogum
Ocimum basilicum L.	Lamiaceae	s pop	WI	Basilikum	syst	URO	p. 105	Volksmedizin: Diuretikum
Onopordum acanthium L.	Asteraceae	ant	DIOS	Eselsdistel	syst	SKE	171	sind im Trank bei Opisthtonie heilsam
Onopordum acanthium L.	Asteraceae	mon	LO	Eselsdistel	syst	OTH	n.d.	n.d.
Onopordum acanthium L.	Asteraceae	ren	LF	Eselsdistel	syst	ANT	XXXI - XXXII / XVI	[gut den jungen kindern] unnd denen so von den natern gebissen seind
Onopordum acanthium L.	Asteraceae	ren	LF	Eselsdistel	syst	ANT	XXXI - XXXII / XVI	widerstehet auch allem anderen gifft
Onopordum acanthium L.	Asteraceae	ren	LF	Eselsdistel	syst	GAS	XXXI - XXXII / XVI	auch denen so einen bloeden magen haben un die speiss nit wol behalten moegen
Onopordum acanthium L.	Asteraceae	ren	LF	Eselsdistel	syst	RES	XXXI - XXXII / XVI	gut denen so blut aussreüspern
Onopordum acanthium L.	Asteraceae	ren	LF	Eselsdistel	syst	SKE	XXXI - XXXII / XVI	gut den jungen kindern so it dem gicht oder freysch beladen
Onopordum acanthium L.	Asteraceae	ren	LF	Eselsdistel	syst	TEE	XXXI - XXXII / XVI	legt das zanwee
Onopordum acanthium L.	Asteraceae	ren	LF	Eselsdistel	syst	URO	XXXI - XXXII / XVI	treibt auch den harn
Onopordum acanthium L.	Asteraceae	ren	LF	Eselsdistel	syst	URO	XXXI - XXXII / XVI	so mans überstreicht vertreibt sie die geschwulst
Onopordum acanthium L.	Asteraceae	s pop	MA	Eselsdistel	hom	DER		veralteten Ulzera, Lupus und Skrofulose; Hautkarzinom (nicht erste W
Origanum vulgare aggr.	Lamiaceae	ant	DIOS	Dost	syst	ANT	176	helfen gegen den Biß giftiger Tiere
Origanum vulgare aggr.	Lamiaceae	mon	HvB	Dost	top	DER	1-112	rote Lepra
Origanum vulgare aggr.	Lamiaceae	mon	HvB	Dost	syst	FEV	1-112	tägliches Fieber
Origanum vulgare aggr.	Lamiaceae	mon	MF	Dost	syst	ANT	38	heilt alle verderblichen Bisse, falls jemand Aconitum genossen hat, widersetzt sich vielen anderen Giften
Origanum vulgare aggr.	Lamiaceae	mon	MF	Dost	syst	DER	38	heilt er Wunden im Mund
Origanum vulgare aggr.	Lamiaceae	mon	MF	Dost	syst	DER	38	vertrocknet Geschwülste
Origanum vulgare aggr.	Lamiaceae	mon	MF	Dost	top	DER	38	Juckreiz, Kopfgrind, und Male auf der Haut
Origanum vulgare aggr.	Lamiaceae	mon	MF	Dost	top	EAR	38	stillt er Ohrenschmerzen
Origanum vulgare aggr.	Lamiaceae	mon	MF	Dost	syst	GAS	38	zögerliche Verdauung
Origanum vulgare aggr.	Lamiaceae	mon	MF	Dost	top	GAS	38	bessert die Gelbsucht
Origanum vulgare aggr.	Lamiaceae	mon	MF	Dost	syst	GAS	38	befördert die Spulwürmer hinaus
Origanum vulgare aggr.	Lamiaceae	mon	MF	Dost	syst	GYN	38	mindert er überstarken Monatsfluss
Origanum vulgare aggr.	Lamiaceae	mon	MF	Dost	top	GYN	38	mindert er überstarken Monatsfluss
Origanum vulgare aggr.	Lamiaceae	mon	MF	Dost	top	HUM	38	zwingt er böses Blut im Kopf hinauszufliessen
Origanum vulgare aggr.	Lamiaceae	mon	MF	Dost	syst	RES	38	treibt er Husten zurück
Origanum vulgare aggr.	Lamiaceae	mon	MF	Dost	syst	RES	38	heilt er Schwellungen des Rachenzäpfchens und des Schlundes
Origanum vulgare aggr.	Lamiaceae	mon	MF	Dost	syst	SKE	38	heilt er Prellungen
Origanum vulgare aggr.	Lamiaceae	mon	MF	Dost	top	SKE	38	verrenkten und zerstossenen Gliedern
Origanum vulgare aggr.	Lamiaceae	mon	MF	Dost	top	TEE	38	pflegt er den Zahnschmerz zu verjagen
Origanum vulgare aggr.	Lamiaceae	mon	MF	Dost	syst	URO	38	zähmt er die Wassersucht
Origanum vulgare aggr.	Lamiaceae	mon	MF	Dost	syst	URO	38	treibt er den Harn
Origanum vulgare aggr.	Lamiaceae	ren	LF	Dost	syst	ANT	CCCXI / XXIX	ist nützlich denen von den gifftigen thiern gebissen sind, mit süssem wein denen si Schirling oder den Safft von Magsamen gesen oder ingenommen haben. / Gyps oder Zeitlosen gegessen
Origanum vulgare aggr.	Lamiaceae	ren	LF	Dost	top	DER	CCCXI / XXIX	verzerte die kropff
Origanum vulgare aggr.	Lamiaceae	ren	LF	Dost	top	DER	CCCXI / XXIX	denen mit rauden beladen sind
Origanum vulgare aggr.	Lamiaceae	ren	LF	Dost	top	DER	CCCXI / XXIX	geschwollenen Mandeln, dem zaepfling und geschwaeren des munds
Origanum vulgare aggr.	Lamiaceae	ren	LF	Dost	top	DER	CCCXI / XXIX	vertreibt und verzert allerley geschwulst, verzert das undergrunnen blut
Origanum vulgare aggr.	Lamiaceae	ren	LF	Dost	top	EAR	CCCXI / XXIX	lindert den schmerzen der ohren,
Origanum vulgare aggr.	Lamiaceae	ren	LF	Dost	vol	EAR	CCCXI / XXIX	zerteylet das sausen und klingen der ohren
Origanum vulgare aggr.	Lamiaceae	ren	LF	Dost	syst	GAS	CCCXI / XXIX	die geelsucht haben
Origanum vulgare aggr.	Lamiaceae	ren	LF	Dost	syst	GYN	CCCXI / XXIX	bring den frawen ir zeit
Origanum vulgare aggr.	Lamiaceae	ren	LF	Dost	syst	GYN	CCCXI / XXIX	treibt die schwarzen gallen durch den stulgang
Origanum vulgare aggr.	Lamiaceae	ren	LF	Dost	top	n.d.	CCCXI / XXIX	stillt der den weetagen der seiten und das stechen
Origanum vulgare aggr.	Lamiaceae	ren	LF	Dost	syst	NER	CCCXI / XXIX	denen den krampff haben
Origanum vulgare aggr.	Lamiaceae	ren	LF	Dost	syst	RES	CCCXI / XXIX	hützlich zu dem husten
Origanum vulgare aggr.	Lamiaceae	ren	LF	Dost	top	TEE	CCCXI / XXIX	weisse zaen
Origanum vulgare aggr.	Lamiaceae	ren	LF	Dost	syst	URO	CCCXI / XXIX	wassersüchtig
Origanum vulgare aggr.	Lamiaceae	s pop	JK	Dost	syst	CAR	p. 392	verstärken Blutzirkulation
Origanum vulgare aggr.	Lamiaceae	s pop	JK	Dost	syst	GAS	p. 392	reinigen Magen
Origanum vulgare aggr.	Lamiaceae	s pop	JK	Dost	syst	GYN	p. 393	Beschwerden der Unterleibsorgane, verstockte Periode, Weissfluss
Origanum vulgare aggr.	Lamiaceae	s pop	JK	Dost	vol	OTH	p. 396	guter Rauchtabak
Origanum vulgare aggr.	Lamiaceae	s pop	JK	Dost	syst	RES	p. 392	reinigen Lungen
Origanum vulgare aggr.	Lamiaceae	s pop	JK	Dost	top	SKE	p. 395	rheumatische Schmerzen

Origanum vulgare aggr.	Lamiaceae	s pop	JK	Dost	top	TEE	p. 394	Schmerzen, die von hohlen Zähnen her kommen
Origanum vulgare aggr.	Lamiaceae	s pop	JK	Dost	top	TON	p. 393	Kraftbäder; Genesende, schwache Frauen, serbelnde Kinder
Origanum vulgare aggr.	Lamiaceae	s pop	UB	Dost	syst	RES	p. 225	krampflindernde Heilpflanzen [bei Husten]
Papaver somniferum L.	Papaveraceae	ant	DIOS	Schlafmohn	top	DER	228	bei Rose und Wunden
Papaver somniferum L.	Papaveraceae	ant	DIOS	Schlafmohn	top	DER	228	Kataplasma ein gutes Mittel bei Geschwülsten und Rose
Papaver somniferum L.	Papaveraceae	ant	DIOS	Schlafmohn	top	EAR	228	Ohrenschmerzen
Papaver somniferum L.	Papaveraceae	ant	DIOS	Schlafmohn	top	EYE	228	Bei Augenentzündungen
Papaver somniferum L.	Papaveraceae	ant	DIOS	Schlafmohn	syst	GAS	228	und die Verdauung befördernd und [hilft auch bei] Magenaffectionen
Papaver somniferum L.	Papaveraceae	ant	DIOS	Schlafmohn	syst	GAS	228	Leckmittel, welches schmerzstillend wirkt bei [Husten, Fluss (Erkältung) der Luftröhre und] Magenaffectionen.
Papaver somniferum L.	Papaveraceae	ant	DIOS	Schlafmohn	syst	GAS	228	Bauchfluss
Papaver somniferum L.	Papaveraceae	ant	DIOS	Schlafmohn	syst	GYN	228	Fluss der Frauen
Papaver somniferum L.	Papaveraceae	ant	DIOS	Schlafmohn	syst	NER	228	schmerzstillend, schlafmachend; lindert aber auch Kopfschmerzen, wenn er mit Rosenöl aufgesprengt
Papaver somniferum L.	Papaveraceae	ant	DIOS	Schlafmohn	top	NER	228	als Stuhlzäpfchen eingelegt macht er Schlaf
Papaver somniferum L.	Papaveraceae	ant	DIOS	Schlafmohn	syst	NER	228	auch gegen Schlaflosigkeit getrunken
Papaver somniferum L.	Papaveraceae	ant	DIOS	Schlafmohn	vol	NER	228	bewirken die in Wasser gekochten Blätter und Köpfe als Bähmittel Schlaf
Papaver somniferum L.	Papaveraceae	ant	DIOS	Schlafmohn	syst	NER	228	bei den an Schlaflosigkeit Leidenden
Papaver somniferum L.	Papaveraceae	ant	DIOS	Schlafmohn	syst	RES	228	hilft auch bei Husten
Papaver somniferum L.	Papaveraceae	ant	DIOS	Schlafmohn	syst	RES	228	Leckmittel, welches schmerzstillend wirkt bei Husten, Fluss (Erkältung) der Luftröhre [und Magenaffectionen].
Papaver somniferum L.	Papaveraceae	ant	DIOS	Schlafmohn	top	SKE	228	bei Podagra
Papaver somniferum L.	Papaveraceae	mon	HvB	Mohn	syst	DER	1-96	und verhindern den Juckreiz, [und sie unterdrücken die rasenden Läuse und Nisse]
Papaver somniferum L.	Papaveraceae	mon	HvB	Mohn	syst	NER	1-96	seine Körner führen den Schlaf herbei
Papaver somniferum L.	Papaveraceae	mon	HvB	Mohn	syst	OTH	1-96	und sie unterdrücken die rasenden Läuse und Nisse
Papaver somniferum L.	Papaveraceae	mon	MF	Schwarzer Mohn	top	DER	32	Antoniusfeuer löschen
Papaver somniferum L.	Papaveraceae	mon	MF	Weisser Mohn	top	EAR	32	träufelst in die Ohren
Papaver somniferum L.	Papaveraceae	mon	MF	Weisser Mohn	syst	GAS	32	schützt Magen und Leib vor Unregelmässigkeiten
Papaver somniferum L.	Papaveraceae	mon	MF	Schwarzer Mohn	syst	GAS	32	Durchfall hemmen
Papaver somniferum L.	Papaveraceae	mon	MF	Schwarzer Mohn	syst	GYN	32	Monatsfluss hemmen
Papaver somniferum L.	Papaveraceae	mon	MF	Weisser Mohn	syst	NER	32	bringt Schlummer
Papaver somniferum L.	Papaveraceae	mon	MF	Weisser Mohn	top	NER	32	behebst du allen Schmerz, bereitest die Ruhe und Schlummer
Papaver somniferum L.	Papaveraceae	mon	MF	Weisser Mohn	top	NER	32	bringt Schlummer
Papaver somniferum L.	Papaveraceae	mon	MF	Weisser Mohn	vol	NER	32	After aufgetragen oder als Zäpfchen verwendet, pflegt sie die Schlaf herbei zu führen, allein ihrem Duft zu verdanken sein
Papaver somniferum L.	Papaveraceae	mon	MF	Schwarzer Mohn	syst	NER	32	führt als Trunk den Schlaf herbei
Papaver somniferum L.	Papaveraceae	mon	MF	Schwarzer Mohn	top	RES	32	geschwollener Schlund heilen, Stimmbänder .... rauh geworden
Papaver somniferum L.	Papaveraceae	mon	MF	Weisser Mohn	syst	RES	32	vertreibt Husten, trocknet bösen Säftefluss zu den Stimmbändern aus
Papaver somniferum L.	Papaveraceae	mon	MF	Schwarzer Mohn	syst	RES	32	zähmt den bösen Husten;
Papaver somniferum L.	Papaveraceae	mon	MF	Weisser Mohn	top	SKE	32	Fussgicht zu Milde zwingen
Papaver somniferum L.	Papaveraceae	ren	LF	Meisterwurz	n.d.	ANT	CCCXXXVII / CCXCII	zu der zeit der pestilentz wider den vergifften boesen luft gebraucht werden
Papaver somniferum L.	Papaveraceae	ren	LF	Schlafmohn	top	DER	CCXCIII / CXCVI	gut zum rotlauff un dergleichen hitzigen gebresten
Papaver somniferum L.	Papaveraceae	ren	LF	Schlafmohn	top	EAR	CCXCIII / CXCVI	benimmbt er den schmerzen derselbigen [ohren]
Papaver somniferum L.	Papaveraceae	ren	LF	Schlafmohn	top	EYE	CCXCIII / CXCVI	gut zu den hitzigen Augen
Papaver somniferum L.	Papaveraceae	ren	LF	Schlafmohn	syst	GAS	CCXCIII / CXCVI	zu dem bauchfluss
Papaver somniferum L.	Papaveraceae	ren	LF	Schlafmohn	syst	GYN	CCXCIII / CXCVI	und den frawen so zuvil fliessen
Papaver somniferum L.	Papaveraceae	ren	LF	Schlafmohn	syst	NER	CCXCIII / CXCVI	vertreibt das vil wachen
Papaver somniferum L.	Papaveraceae	ren	LF	Schlafmohn	top	NER	CCXCIII / CXCVI	bring den schlaff
Papaver somniferum L.	Papaveraceae	ren	LF	Schlafmohn	top	NER	CCXCIII / CXCVI	bring den schlaff
Papaver somniferum L.	Papaveraceae	ren	LF	Schlafmohn	top	NER	CCXCIII / CXCVI	stillt er den weetagen des haupts
Papaver somniferum L.	Papaveraceae	ren	LF	Schlafmohn	top	NER	CCXCIII / CXCVI	an die tirn un schlaeffen gestrichen vertreibt das vil wachen
Papaver somniferum L.	Papaveraceae	ren	LF	Schlafmohn	syst	RES	CCXCIII / CXCVI	seer nütz zu dem husten
Papaver somniferum L.	Papaveraceae	ren	LF	Schlafmohn	top	SKE	CCXCIII / CXCVI	denen so das podagram haben
Papaver somniferum L.	Papaveraceae	s pop	VAL	Weisser Mohn	syst	GAS	p. 406	diarrhées, dysenterie
Papaver somniferum L.	Papaveraceae	s pop	VAL	Weisser Mohn	syst	NER	p. 406	toutes les douleurs, anxiété, insomnies
Papaver somniferum L.	Papaveraceae	s pop	VAL	Weisser Mohn	syst	RES	p. 406	hémoptysis, affection pulmonaire
Petasites hybridus (L.) Gaertn.	Asteraceae	ant	DIOS	Pestwurz	top	DER	240	es wirkt, fein gestossen, als Umschlag gegen bösartige und krebsige Geschwüre
Petasites hybridus (L.) Gaertn.	Asteraceae	mon	HvB	Grossblättriger Hufl	top	DER	1-210	Skrofeln
Petasites hybridus (L.) Gaertn.	Asteraceae	ren	LF	Pestwurz	top	DER	CCLXVII / CCXLVIII	in die boesen umbfressenden wunden und geschwaer gethon
Petasites hybridus (L.) Gaertn.	Asteraceae	ren	LF	Pestwurz	syst	DER	CCLXVII / CCXLVIII	heylet die seer feuchten wunden und andere unreynigkeyt der haut
Petasites hybridus (L.) Gaertn.	Asteraceae	ren	LF	Pestwurz	syst	FEV	CCLXVII / CCXLVIII	wider die gifftigen und pestilentzischen fieber
Petasites hybridus (L.) Gaertn.	Asteraceae	ren	LF	Pestwurz	syst	GAS	CCLXVII / CCXLVIII	toedt die würm im leib
Petasites hybridus (L.) Gaertn.	Asteraceae	ren	LF	Pestwurz	syst	GYN	CCLXVII / CCXLVIII	wider das grimmen und aufsteigen der muter
Petasites hybridus (L.) Gaertn.	Asteraceae	ren	LF	Pestwurz	syst	GYN	CCLXVII / CCXLVIII	bringt den frawen ir zeit
Petasites hybridus (L.) Gaertn.	Asteraceae	ren	LF	Pestwurz	syst	HUM	CCLXVII / CCXLVIII	treibt den schweyss mit gewalt aus
Petasites hybridus (L.) Gaertn.	Asteraceae	ren	LF	Pestwurz	syst	RES	CCLXVII / CCXLVIII	gut und nützlich denen so schwerlich athmen
Petasites hybridus (L.) Gaertn.	Asteraceae	ren	LF	Pestwurz	syst	URO	CCLXVII / CCXLVIII	treibt den harn und bringt den frawen ir zeit
Petasites hybridus (L.) Gaertn.	Asteraceae	s pop	BVA	Pestwurz	top	DER	p. 161	Krampfadern
Petasites hybridus (L.) Gaertn.	Asteraceae	s pop	BVA	Pestwurz	syst	GAS	p. 161	Magenkrämpfen
Petasites hybridus (L.) Gaertn.	Asteraceae	s pop	BVA	Pestwurz	syst	GAS	p. 161	Magenkrämpfen
Petasites hybridus (L.) Gaertn.	Asteraceae	s pop	BVA	Pestwurz	syst	NER	p. 161	Krämpfen
Petasites hybridus (L.) Gaertn.	Asteraceae	s pop	BVA	Pestwurz	syst	NER	p. 161	Krämpfen
Petasites hybridus (L.) Gaertn.	Asteraceae	s pop	BVA	Pestwurz	syst	NER	p. 161	Nervenschwäche, nervösem Magen
Petasites hybridus (L.) Gaertn.	Asteraceae	s pop	BVA	Pestwurz	syst	NER	p. 161	Nervenschwäche, nervösem Magen
Petasites hybridus (L.) Gaertn.	Asteraceae	s pop	BVA	Pestwurz	syst	RES	p. 161	Husten, Bronchitis
Petasites hybridus (L.) Gaertn.	Asteraceae	s pop	BVA	Pestwurz	syst	RES	p. 161	Husten, Bronchitis
Petasites hybridus (L.) Gaertn.	Asteraceae	s pop	BVA	Pestwurz	top	SKE	p. 161	Gelenkschmerzen
Petasites hybridus (L.) Gaertn.	Asteraceae	s pop	JK	Pestwurz	top	DER	p. 402	Kühlmittel für Brandwunden, offene Wunden, Gichtknoten, Bienen- und Wespenstiche
Petasites hybridus (L.) Gaertn.	Asteraceae	s pop	JK	Pestwurz	syst	HUM	p. 402	stark schweisstreibend
Petasites hybridus (L.) Gaertn.	Asteraceae	s pop	JK	Pestwurz	syst	TON	p. 402	Vorbeugungsmittel gegen Diphterie, Grippe und andere suchenartig ansteckende Krankheiten
Petasites hybridus (L.) Gaertn.	Asteraceae	s pop	MT	Hirtentäschel	top	DER	p. 23	Brand, bösartigen Geschwüren und brennenden Wunden
Petasites hybridus (L.) Gaertn.	Asteraceae	s pop	MT	Hirtentäschel	syst	FEV	p. 23	Fieber
Petasites hybridus (L.) Gaertn.	Asteraceae	s pop	MT	Hirtentäschel	syst	HUM	p. 23	schweisstreibend (Fieber, Atemnot, Gicht, Fallsucht)
Petasites hybridus (L.) Gaertn.	Asteraceae	s pop	MT	Hirtentäschel	top	SKE	p. 23	Verstauchungen, Verrenkungen, wundgelaufende Füsse
Petasites hybridus (L.) Gaertn.	Asteraceae	s pop	UB	Pestwurz	syst	NER	p. 441	Migräne
Petasites hybridus (L.) Gaertn.	Asteraceae	s pop	UB	Pestwurz	syst	URO	p. 259	Nieren- und Harnleitersteine
Petasites hybridus (L.) Gaertn.	Asteraceae	sci	WI	Pestwurz	syst	GAS	p. 429	Magen-Darm-Bereich
Petasites hybridus (L.) Gaertn.	Asteraceae	sci	WI	Pestwurz	syst	RES	p. 429	Astmah
Petasites hybridus (L.) Gaertn.	Asteraceae	sci	WI	Pestwurz	syst	URO	p. 429	ableitenden Harnwege
Petroselinum crispum (Mill.) Fuss	Apiaceae	ant	DIOS	Petersilie	syst	GAS	187	auch ein gutes Mittel gegen Aufblähen des Magens und Kolons und gegen Leibschneiden
Petroselinum crispum (Mill.) Fuss	Apiaceae	ant	DIOS	Petersilie	syst	GYN	187	welcher [den Harn und] die Menstruation befördert
Petroselinum crispum (Mill.) Fuss	Apiaceae	ant	DIOS	Petersilie	syst	URO	187	welcher den Harn [und die Menstruation] befördert
Petroselinum crispum (Mill.) Fuss	Apiaceae	ant	DIOS	Petersilie	syst	URO	187	ebenso gegen Seiten-, Nieren- und Blasenschmerzen
Petroselinum crispum (Mill.) Fuss	Apiaceae	mon	HvB	Petersilie	syst	FEV	1-68	mildert Fiber, die Menschen nicht erschüttern, nur berühren
Petroselinum crispum (Mill.) Fuss	Apiaceae	mon	HvB	Petersilie	syst	GAS	1-68	kranker Magen
Petroselinum crispum (Mill.) Fuss	Apiaceae	mon	HvB	Petersilie	syst	n.d.	1-68	Schmerzen im Herz, in der Milz oder in der Seite
Petroselinum crispum (Mill.) Fuss	Apiaceae	mon	HvB	Petersilie	top	SKE	1-68	Lähmung
Petroselinum crispum (Mill.) Fuss	Apiaceae	mon	HvB	Petersilie	syst	URO	1-68	am Stein leidet
Petroselinum crispum (Mill.) Fuss	Apiaceae	mon	HvB	Petersilie	top	URO	1-68	am Stein leidet
Petroselinum crispum (Mill.) Fuss	Apiaceae	ren	LF	Petersilie	syst	ANT	CCCXXVI / CCXVII	braucht sie auch zu den arzneyen so für das gifft gemacht
Petroselinum crispum (Mill.) Fuss	Apiaceae	ren	LF	Petersilie	top	EYE	CCCXXVI / CCXVII	ist er gut zu den hitzigen augen [aus Kapitel Eppich]
Petroselinum crispum (Mill.) Fuss	Apiaceae	ren	LF	Petersilie	syst	GAS	CCCXXVI / CCXVII	verhindert die aufblaehung des magens, fürdern die dewung, eröffnen das milz und die leber

Petroselinum crispum (Mill.) Fuss	Apiaceae	ren	LF	Petersilie	top	GYN	CCCXXVI / CCXVII	verzert auch die knollen in den brüsten der frawen [aus Kapitel Eppich]
Petroselinum crispum (Mill.) Fuss	Apiaceae	ren	LF	Petersilie	syst	GYN	CCCXXVI / CCXVII	bringen den frawen ir zeit
Petroselinum crispum (Mill.) Fuss	Apiaceae	ren	LF	Petersilie	syst	URO	CCCXXVI / CCXVII	treiben den harn
Petroselinum crispum (Mill.) Fuss	Apiaceae	s pop	JK	Petersilie	syst	CAR	p. 402	Herz-, Milz- und Seitenstechen
Petroselinum crispum (Mill.) Fuss	Apiaceae	s pop	JK	Petersilie	syst	GAS	p. 402	Leberleiden und Gelbsucht
Petroselinum crispum (Mill.) Fuss	Apiaceae	s pop	JK	Petersilie	syst	URO	p. 402	Wassersucht, Steinleiden, Blasenleiden
Petroselinum crispum (Mill.) Fuss	Apiaceae	s pop	JK	Petersilie	syst	URO	p. 402	Blutpissen, schmerzhaftem urinieren, Prostataentzündung
Petroselinum crispum (Mill.) Fuss	Apiaceae	s pop	UB	Petersilie	syst	URO	p. 256	Durchspülung
Petroselinum crispum (Mill.) Fuss	Apiaceae	s pop	UB	Petersilie	syst	URO	p. 256	Durchspülung
Petroselinum crispum (Mill.) Fuss	Apiaceae	s pop	WI	Petersilie	syst	GAS	p. 433	Volksmedizin: Stomachikum
Petroselinum crispum (Mill.) Fuss	Apiaceae	s pop	WI	Petersilie	syst	GYN	p. 433	Volksmedizin: Dysmenorrhoe, Menstruationsbeschwerden / Emenagogum, Galaktagogum
Petroselinum crispum (Mill.) Fuss	Apiaceae	s pop	WI	Petersilie	syst	GYN	p. 433	Volksmedizin: Dysmenorrhoe, Menstruationsbeschwerden / Emenagogum, Galaktagogum
Petroselinum crispum (Mill.) Fuss	Apiaceae	s pop	WI	Petersilie	top	OTH	p. 433	Volksmedizin: Kopfläuse
Petroselinum crispum (Mill.) Fuss	Apiaceae	sci	WI	Petersilie	syst	GAS	p. 433	Stomachikum
Petroselinum crispum (Mill.) Fuss	Apiaceae	sci	WI	Petersilie	top	OTH	p. 433	Kopfläuse
Petroselinum crispum (Mill.) Fuss	Apiaceae	sci	WI	Petersilie	syst	URO	p. 433	kräftiges Diuretikum
Petroselinum crispum (Mill.) Fuss	Apiaceae	sci	WI	Petersilie	syst	URO	p. 433	Diuretikum
Peucedanum sp.	Apiaceae	ant	DIOS	Haarstrang	top	DER	p. 190	reinigt sie schmutzige Geschwüre, zieht Knochensplitter aus undbringt alte Wunden zur Vernarbung.
Peucedanum sp.	Apiaceae	ant	DIOS	Haarstrang	top	EAR	p. 190	eingeträpfelt bei Ohrenleiden
Peucedanum sp.	Apiaceae	ant	DIOS	Haarstrang	syst	GYN	p. 190	hilft vorzüglich bei schweren Geburten.
Peucedanum sp.	Apiaceae	ant	DIOS	Haarstrang	top	NER	p. 190	gutes Mittel bei Lethargie, Hirnkrankheit, Schwindel, Epilepsie, chronischem Kopfschmerz, Paralyse, Ischias, überhaupt bei allen Nervenleiden, wenn er mit Öl und Essig eingeschmiert wird
Peucedanum sp.	Apiaceae	ant	DIOS	Haarstrang	syst	OTH	p. 190	verkleinert die Milz
Peucedanum sp.	Apiaceae	ant	DIOS	Haarstrang	syst	RES	p. 190	bei Husten, ebenso bei Athemnoth
Peucedanum sp.	Apiaceae	ant	DIOS	Haarstrang	syst	RES	p. 190	Leibschneiden und Blähungszuständen
Peucedanum sp.	Apiaceae	ant	DIOS	Haarstrang	top	TEE	p. 190	in den angefressenen Zahn gesteckt bei Zahnschmerzen
Peucedanum sp.	Apiaceae	ant	DIOS	Haarstrang	syst	URO	p. 190	ein gutes Mittel gegen Schmerzen und Spannung der Blase und der Nieren
Peucedanum sp.	Apiaceae	mon	HvB	Meisterwurz	syst	FEV	1-167	Denn wer Fieber hat, welcher Art es auch sei, . . .
Peucedanum sp.	Apiaceae	mon	HvB	Meisterwurz	syst	GAS	1-167	Wer aber die gegessene Speise nicht verdauen kann
Peucedanum sp.	Apiaceae	mon	MF	Meisterwurz	top	DER	26	heilt jedwedes Blatterbläschen
Peucedanum sp.	Apiaceae	mon	MF	Meisterwurz	top	DER	26	reinigt den Aussatz
Peucedanum sp.	Apiaceae	mon	MF	Meisterwurz	syst	GAS	26	vertreibt alle Leiden der Leber, hilft Gelbsüchtigen
Peucedanum sp.	Apiaceae	mon	MF	Meisterwurz	top	GAS	26	hilft den Gelbsüchtigen, wenn sie ihn in die Nase saugen, gemischt mit Weibermilch
Peucedanum sp.	Apiaceae	mon	MF	Meisterwurz	syst	GYN	26	bringt Monatsregel in Gang
Peucedanum sp.	Apiaceae	mon	MF	Meisterwurz	top	GYN	26	soll die Leibesfrucht abtreiben und reichlich Monatsfluss hervorrufen
Peucedanum sp.	Apiaceae	mon	MF	Meisterwurz	syst	RES	26	lindert Husten, hilft auch bei Atemnot
Peucedanum sp.	Apiaceae	mon	MF	Meisterwurz	vol	RES	26	Niesmittel, wenn die Säfte vom Haupt herunter fliessen
Peucedanum sp.	Apiaceae	mon	MF	Meisterwurz	syst	URO	26	soll Blasensteine brechen, bringt Harnfluss in Gang
Peucedanum sp.	Apiaceae	ren	LF	Meisterwurz	top	ANT	CCCXXXVII / CCXCII	gut wider allerley stich und biss der gifftigen thier
Peucedanum sp.	Apiaceae	ren	LF	Meisterwurz	top	ANT	CCCXXXVII / CCXCII	heylet er die wunden son von einem wuetenden hund oder nattern gebissen seind
Peucedanum sp.	Apiaceae	ren	LF	Meisterwurz	n.d.	ANT	CCCXXXVII / CCXCII	seind trefflich nütz und gut wider allerley gifft.
Peucedanum sp.	Apiaceae	ren	LF	Meisterwurz	vol	DER	CCCXXXVII / CCXCII	wartzen so am hindern gewachsen seind
Peucedanum sp.	Apiaceae	ren	LF	Meisterwurz	top	DER	CCCXXXVII / CCXCII	heylet er die flecht
Peucedanum sp.	Apiaceae	ren	LF	Meisterwurz	top	DER	CCCXXXVII / CCXCII	zerteylet die knollechte milch unnd das zusamen gerunnen blut
Peucedanum sp.	Apiaceae	ren	LF	Meisterwurz	syst	GAS	CCCXXXVII / CCXCII	sterckt er den magen, macht lust zu essen
Peucedanum sp.	Apiaceae	ren	LF	Meisterwurz	syst	GAS	CCCXXXVII / CCXCII	treibt den harn
Peucedanum sp.	Apiaceae	ren	LF	Meisterwurz	syst	GAS	CCCXXXVII / CCXCII	geelsucht
Peucedanum sp.	Apiaceae	ren	LF	Meisterwurz	syst	GAS	CCCXXXVII / CCXCII	wassersüchtig seind
Peucedanum sp.	Apiaceae	ren	LF	Meisterwurz	syst	GYN	CCCXXXVII / CCXCII	reynnigt auch die muter und treibt aus die todte frucht
Peucedanum sp.	Apiaceae	ren	LF	Meisterwurz	syst	GYN	CCCXXXVII / CCXCII	bringt den frawen ir zeit
Peucedanum sp.	Apiaceae	ren	LF	Meisterwurz	syst	NER	CCCXXXVII / CCXCII	ist gut zu dem krampff und dergleichen kalten gebresten der nerven
Peucedanum sp.	Apiaceae	ren	LF	Meisterwurz	syst	OTH	CCCXXXVII / CCXCII	heylet die innerlichen wunden
Peucedanum sp.	Apiaceae	ren	LF	Meisterwurz	n.d.	RES	CCCXXXVII / CCXCII	gut zu dem husten der von kellte kompt
Peucedanum sp.	Apiaceae	ren	LF	Meisterwurz	syst	RES	CCCXXXVII / CCXCII	gut zu der kalten lungen dem keichen unnd andern dergleichen gebresten der brust
Peucedanum sp.	Apiaceae	ren	LF	Meisterwurz	top	SKE	CCCXXXVII / CCXCII	gut zu dem hüftwee
Peucedanum sp.	Apiaceae	ren	LF	Meisterwurz	top	SKE	CCCXXXVII / CCXCII	podagra
Peucedanum sp.	Apiaceae	ren	LF	Meisterwurz	syst	TON	CCCXXXVII / CCXCII	gut zu bewegung des schweyss, hilfft dem erstorben kalten mann wider auff
Peucedanum sp.	Apiaceae	ren	LF	Meisterwurz	syst	TON	CCCXXXVII / CCXCII	überkompt der ganze leib ein gute farb
Peucedanum sp.	Apiaceae	s pop	JK	Meisterwurz	syst	ANT	p. 396	inneren Vergiftungen
Peucedanum sp.	Apiaceae	s pop	JK	Meisterwurz	syst	ANT	p. 396	inneren Vergiftungen
Peucedanum sp.	Apiaceae	s pop	JK	Meisterwurz	syst	ANT	p. 396	Blutvergiftungen
Peucedanum sp.	Apiaceae	s pop	JK	Meisterwurz	top	DER	p. 396	Blutvergiftungen durch rostige Nägel, infizierte Wunden, Aissen und Furunkeln
Peucedanum sp.	Apiaceae	s pop	JK	Meisterwurz	syst	GAS	p. 396	Durchfall
Peucedanum sp.	Apiaceae	s pop	JK	Meisterwurz	syst	NER	p. 396	vom Schlag getroffen, das Fallend Weh
Peucedanum sp.	Apiaceae	s pop	JK	Meisterwurz	syst	OTH	p. 396	inneren Blutungen
Peucedanum sp.	Apiaceae	s pop	JK	Meisterwurz	vol	RES	p. 397	Schnupfen
Peucedanum sp.	Apiaceae	s pop	JK	Meisterwurz	top	SKE	p. 396	schmerzhafte Gichtknoten
Peucedanum sp.	Apiaceae	s pop	JK	Meisterwurz	top	TEE	p. 396	Zahnweh
Peucedanum sp.	Apiaceae	s pop	JK	Meisterwurz	syst	TON	p. 396	ansteckende Krankheiten drohen, wie Grippe, Typhus, Cholera
Peucedanum sp.	Apiaceae	s pop	JK	Meisterwurz	syst	URO	p. 396	Nierensteinen
Pimpinella saxifraga aggr.	Apiaceae	ant	DIOS	Bibernelle	syst	URO	150	Es ist harntreibend
Pimpinella saxifraga aggr.	Apiaceae	mon	HvB	Bibiernell	top	APO	1-131	stets an Hals gehängt, nicht von Anrufungen der Dämonen, magischen Worten und von Zauber getäuscht werden
Pimpinella saxifraga aggr.	Apiaceae	ren	LF	Kleine Bibernelle	syst	ANT	CCCXLVI / CCXXXII	wider allerley gifft, insonderheyt aber denen so von den natern gebissen seind
Pimpinella saxifraga aggr.	Apiaceae	ren	LF	Kleine Bibernelle	syst	APO	CCCXLVI / CCXXXII	gut zu der zeit der pestilenz gebraucht
Pimpinella saxifraga aggr.	Apiaceae	ren	LF	Kleine Bibernelle	top	DER	CCCXLVI / CCXXXII	vertreibt die maeler und flecken desselbigen [Angesicht]
Pimpinella saxifraga aggr.	Apiaceae	ren	LF	Kleine Bibernelle	top	DER	CCCXLVI / CCXXXII	renyget er dieselbigen [unreyn wunden]
Pimpinella saxifraga aggr.	Apiaceae	ren	LF	Kleine Bibernelle	top	EYE	CCCXLVI / CCXXXII	macht ein klar gesicht
Pimpinella saxifraga aggr.	Apiaceae	ren	LF	Kleine Bibernelle	syst	URO	CCCXLVI / CCXXXII	bricht den stein der blasen und treibt den harn
Pimpinella saxifraga aggr.	Apiaceae	s pop	JK	Bibernelle	top	DER	p. 334	eiternde Wunden und wildes Fleisch
Pimpinella saxifraga aggr.	Apiaceae	s pop	JK	Bibernelle	top	DER	p. 334	Halsleiden
Pimpinella saxifraga aggr.	Apiaceae	s pop	JK	Bibernelle	syst	GAS	p. 334	Magen und Gedärmen
Pimpinella saxifraga aggr.	Apiaceae	s pop	JK	Bibernelle	syst	RES	p. 334	Heiserkeit, Lungenkatarrh
Pimpinella saxifraga aggr.	Apiaceae	s pop	JK	Bibernelle	syst	RES	p. 334	Brust- du Lundenverschleimung, Lungenentzündung
Pimpinella saxifraga aggr.	Apiaceae	s pop	JK	Bibernelle	n.d.	TON	p. 334	Vorbeugungsmittel gegen ansteckende Krankheiten
Pimpinella saxifraga aggr.	Apiaceae	s pop	UB	Bibernelle	syst	RES	p. 220	Auswurfförderung
Pimpinella saxifraga aggr.	Apiaceae	s pop	UB	Bibernelle	syst	RES	p. 220	Auswurfförderung
Pimpinella saxifraga aggr.	Apiaceae	s pop	WI	Bibernelle	syst	GAS	p. 441	Volksheilkunde: Stomachikum
Pimpinella saxifraga aggr.	Apiaceae	s pop	WI	Bibernelle	syst	URO	p. 441	Volksheilkunde: Diuretikum
Pimpinella saxifraga aggr.	Apiaceae	sci	WI	Bibernelle	top	DER	p. 441	entzündliche Erkrankungen der Mund- und Rachenhöhle
Pimpinella saxifraga aggr.	Apiaceae	sci	WI	Bibernelle	syst	RES	p. 441	hustenlinderndes Mittel
Polygonum aviculare aggr.	Polygonaceae	ant	DIOS	Vogelknöterich	syst	ANT	214	hilft er gegen den Biss giftiger Thiere
Polygonum aviculare aggr.	Polygonaceae	ant	DIOS	Vogelknöterich	syst	DER	214	Geschwüren an den Schamtheilen
Polygonum aviculare aggr.	Polygonaceae	ant	DIOS	Vogelknöterich	top	DER	214	bei kriechenden Geschwüren, rosEartigen Entzündungen und Geschwulsten, ebenso bei Oedemen und frischen Wunden
Polygonum aviculare aggr.	Polygonaceae	ant	DIOS	Vogelknöterich	top	EAR	214	Ohrenleiden und Eiterfluss
Polygonum aviculare aggr.	Polygonaceae	ant	DIOS	Vogelknöterich	syst	FEV	214	gegen das Wechselfieber
Polygonum aviculare aggr.	Polygonaceae	ant	DIOS	Vogelknöterich	syst	GAS	214	Blutspeien, Bauchfluss, Cholera
Polygonum aviculare aggr.	Polygonaceae	ant	DIOS	Vogelknöterich	top	GAS	214	Erhitzung des Magens, bei Blutauswurf.
Polygonum aviculare aggr.	Polygonaceae	ant	DIOS	Vogelknöterich	syst	GYN	214	den Fluss der Weiber

Polygonum aviculare aggr.	Polygonaceae	ant	DIOS	Vogelknöterich	syst	URO	214	Harnzwang leiden, er treibt auch stark den Harn
Polygonum aviculare aggr.	Polygonaceae	mon	HvB	Knöterich	syst	OTH	214	hilft er gegen den Biss giftiger Thiere
Polygonum aviculare aggr.	Polygonaceae	ren	LF	Vogelknöterich	syst	ANT	CCCXLIX / CCXXXV	nützlich denen so von gifftigen thieren gebissen seind
Polygonum aviculare aggr.	Polygonaceae	ren	LF	Vogelknöterich	syst	DER	CCCXLIX / CCXXXV	geschwaer an den gemechten
Polygonum aviculare aggr.	Polygonaceae	ren	LF	Vogelknöterich	top	DER	CCCXLIX / CCXXXV	in die nasen gethon stellt er das blut derselbigen
Polygonum aviculare aggr.	Polygonaceae	ren	LF	Vogelknöterich	top	DER	CCCXLIX / CCXXXV	das rotlauff
Polygonum aviculare aggr.	Polygonaceae	ren	LF	Vogelknöterich	top	DER	CCCXLIX / CCXXXV	allerley frische wunden
Polygonum aviculare aggr.	Polygonaceae	ren	LF	Vogelknöterich	top	EAR	CCCXLIX / CCXXXV	legt der den schmerzen derselbigen [ohren]
Polygonum aviculare aggr.	Polygonaceae	ren	LF	Vogelknöterich	syst	FEV	CCCXLIX / CCXXXV	gut zu dem feber
Polygonum aviculare aggr.	Polygonaceae	ren	LF	Vogelknöterich	syst	GAS	CCCXLIX / CCXXXV	cholerischen bauchfluss
Polygonum aviculare aggr.	Polygonaceae	ren	LF	Vogelknöterich	syst	GAS	CCCXLIX / CCXXXV	stelle sie alle bauchflüss, roten rhur,
Polygonum aviculare aggr.	Polygonaceae	ren	LF	Vogelknöterich	syst	GAS	CCCXLIX / CCXXXV	das blutspeien und sonst allerley bluten
Polygonum aviculare aggr.	Polygonaceae	ren	LF	Vogelknöterich	top	GAS	CCCXLIX / CCXXXV	leschen die grosse hitz des magens
Polygonum aviculare aggr.	Polygonaceae	ren	LF	Vogelknöterich	top	GAS	CCCXLIX / CCXXXV	das blutspeien
Polygonum aviculare aggr.	Polygonaceae	ren	LF	Vogelknöterich	top	GYN	CCCXLIX / CCXXXV	stellt der frawen krankheyt
Polygonum aviculare aggr.	Polygonaceae	ren	LF	Vogelknöterich	syst	GYN	CCCXLIX / CCXXXV	der frawen zeit
Polygonum aviculare aggr.	Polygonaceae	ren	LF	Vogelknöterich	syst	RES	CCCXLIX / CCXXXV	denen so blut speien
Polygonum aviculare aggr.	Polygonaceae	ren	LF	Vogelknöterich	n.d.	URO	CCCXLIX / CCXXXV	treibt den harn, den stein und griess
Polygonum aviculare aggr.	Polygonaceae	s pop	JK	Vogelknöterich	syst	GAS	p. 384	Durchfall
Polygonum aviculare aggr.	Polygonaceae	s pop	JK	Vogelknöterich	syst	URO	p. 384	Blasenleiden, Bettnässer
Polygonum aviculare aggr.	Polygonaceae	s pop	KN	Vogelknöterich	syst	GAS	p. 490	reinigend auf Leber und Magen
Polygonum aviculare aggr.	Polygonaceae	s pop	KN	Vogelknöterich	syst	RES	p. 490	reinigend auf Brust
Polygonum aviculare aggr.	Polygonaceae	s pop	KN	Vogelknöterich	syst	URO	p. 490	Nierengries und -steinchen
Polygonum aviculare aggr.	Polygonaceae	s pop	UB	Vogelknöterich	syst	RES	p. 227	Lungenstärkung
Polygonum aviculare aggr.	Polygonaceae	s pop	WI	Vogelknöterich	syst	DER	p. 453	Volksheilkunde: Hämostyptikum / Hautaffektionen
Polygonum aviculare aggr.	Polygonaceae	s pop	WI	Vogelknöterich	syst	RES	p. 453	Volksheilkunde: Expektorans und Sekretolytikum
Polypodium vulgare L.	Polypodiaceae	ant	DIOS	Tüpfelfarn	syst	DER	261	wirkt auch gut bei [Verdrehungen und] Rissen zwischen den Fingern
Polypodium vulgare L.	Polypodiaceae	ant	DIOS	Tüpfelfarn	syst	GAS	261	führt sie Schleim und Galle ab
Polypodium vulgare L.	Polypodiaceae	ant	DIOS	Tüpfelfarn	syst	HUM	261	Zum Purgiren wird sie mit Hühnerfleisch oder Fischen, Bete oder Malve gekocht gegeben
Polypodium vulgare L.	Polypodiaceae	ant	DIOS	Tüpfelfarn	top	SKE	261	wirkt auch gut bei Verdrehungen [und Rissen zwischen den Fingern]
Polypodium vulgare L.	Polypodiaceae	mon	HvB	Engelsüss	syst	GAS	1-205	der in den Eingeweiden Schmerzen hat, nehme, wenn er mager ist und nicht viel krank ist . . .
Polypodium vulgare L.	Polypodiaceae	ren	LF	Engelsüss	top	DER	CXXXIIII / CCXXIIII	heylet die schrunden an den henden und füssen
Polypodium vulgare L.	Polypodiaceae	ren	LF	Engelsüss	top	DER	CXXXIIII / CCXXIIII	in die nasen gethon verzert das übrige fleysch darinn Polypum geheyssen
Polypodium vulgare L.	Polypodiaceae	ren	LF	Engelsüss	syst	GAS	CXXXIIII / CCXXIIII	derselbige purgiert auch
Polypodium vulgare L.	Polypodiaceae	ren	LF	Engelsüss	syst	GAS	CXXXIIII / CCXXIIII	treibt aus allerley gallen und schleim durch den stulgang
Polypodium vulgare L.	Polypodiaceae	ren	LF	Engelsüss	syst	GAS	CXXXIIII / CCXXIIII	desgleichen thut sie auch [treibt aus allerley gallen und schleim durch den stulgang]
Polypodium vulgare L.	Polypodiaceae	ren	LF	Engelsüss	top	SKE	CXXXIIII / CCXXIIII	ist nützlich denen so sich verrenckt haben
Polypodium vulgare L.	Polypodiaceae	s pop	JK	Engelsüss	syst	GAS	p. 348	Wurmmittel
Polypodium vulgare L.	Polypodiaceae	s pop	JK	Engelsüss	syst	RES	p. 348	Enge auf der Brust, Astmah und Husten
Polypodium vulgare L.	Polypodiaceae	s pop	JK	Engelsüss	syst	RES	p. 348	Husten und Katarrh
Polypodium vulgare L.	Polypodiaceae	s pop	JK	Engelsüss	syst	URO	p. 348	Wasserbeschwerden
Polypodium vulgare L.	Polypodiaceae	sci	EMA	Tüpfelfarn	syst	GAS	HMPC/600668/2007	Traditional herbal medicinal product for short-term use in cases of occasional constipation.
Polypodium vulgare L.	Polypodiaceae	sci	EMA	Tüpfelfarn	syst	RES	HMPC/600668/2007	Traditional herbal medicinal product used as an expectorant in cough and cold.
Potentilla sp.	Rosaceae	ant	DIOS	Blutwurz	syst	ANT	222	Saft der zarten Wurzel wirkt[bei Leber- und Lungenleiden] und gegen tödtliche Gifte
Potentilla sp.	Rosaceae	ant	DIOS	Blutwurz	syst	DER	222	heilt Darmbrüche und stillt Blutungen,
Potentilla sp.	Rosaceae	ant	DIOS	Blutwurz	top	DER	222	Wunden und Fisteln
Potentilla sp.	Rosaceae	ant	DIOS	Blutwurz	top	DER	222	auch beseitigt es als Mundspülwasser die Mundfäule und besänftigt als Gurgelwasser die Rauheit der Luftröhre
Potentilla sp.	Rosaceae	ant	DIOS	Blutwurz	top	DER	222	als Umschlag kriechende Geschwüre auf, vertheilt Drüsen am Halse, Verhärtungen, Aneurysmen und Abscesse, heilt roseartige Entzündungen, überwachsene Fingernägel, Feigwarzen und Krätze
Potentilla sp.	Rosaceae	ant	DIOS	Blutwurz	syst	FEV	222	gegen die periodischen Fieber
Potentilla sp.	Rosaceae	ant	DIOS	Blutwurz	syst	GAS	222	Gelbsucht
Potentilla sp.	Rosaceae	ant	DIOS	Blutwurz	syst	GAS	222	hilft es bei Bauchfluss und Dysenterie
Potentilla sp.	Rosaceae	ant	DIOS	Blutwurz	syst	GAS	222	Saft der zarten Wurzel wirkt bei Leber- [und Lungenleiden und gegen tödtliche Gifte]
Potentilla sp.	Rosaceae	ant	DIOS	Blutwurz	syst	HUM	222	wird auch als Reinigungsund Sühnemittel sowie als Mittel gegen Blutfluss geschnitten
Potentilla sp.	Rosaceae	ant	DIOS	Blutwurz	syst	NER	222	Auch bei Epilepsie helfen die Blätter
Potentilla sp.	Rosaceae	ant	DIOS	Blutwurz	syst	RES	222	Saft der zarten Wurzel wirkt bei [Leber-]und Lungenleiden [und gegen tödtliche Gifte]
Potentilla sp.	Rosaceae	ant	DIOS	Blutwurz	syst	SKE	222	Gicht- und Ischiaskranken
Potentilla sp.	Rosaceae	ant	DIOS	Blutwurz	top	TEE	222	Zahnschmerzen zu heilen,
Potentilla sp.	Rosaceae	mon	HvB	Kriechendes Fingerl	top	EYE	1-166	und wenn jemand an Nebel in den Augen leidet
Potentilla sp.	Rosaceae	mon	HvB	Kriechendes Fingerl	top	FEV	1-166	umwinde damit den Bauch des Menschen, der starkes Fieber hat
Potentilla sp.	Rosaceae	mon	HvB	Kriechendes Fingerl	syst	GAS	1-166	auch wer Gelbsucht hat
Potentilla sp.	Rosaceae	mon	HvB	Blutwurz	syst	HUM	1-166	und ein Menschm der überflüssige und giftige das heisst eitrige Säfte in sich hat, . . .
Potentilla sp.	Rosaceae	ren	LF	Fingerkraut	top	DER	[- CCCLV / CCXXXIX	wunden und andere fressende schaeden der fistel
Potentilla sp.	Rosaceae	ren	LF	Fingerkraut	top	DER	[- CCCLV / CCXXXIX	gegurgelt heylet es die geschwaer des munds und die fertigkeit oder verwundung des hals
Potentilla sp.	Rosaceae	ren	LF	Fingerkraut	top	DER	[- CCCLV / CCXXXIX	verzeret sie die kroepff, herte, geschwulst un die rauden
Potentilla sp.	Rosaceae	ren	LF	Fingerkraut	syst	FEV	[- CCCLV / CCXXXIX	verhindert das schütten des febers
Potentilla sp.	Rosaceae	ren	LF	Fingerkraut	syst	GAS	[- CCCLV / CCXXXIX	geelsucht
Potentilla sp.	Rosaceae	ren	LF	Fingerkraut	syst	GAS	[- CCCLV / CCXXXIX	roten rhur, weetagen der gleych un hüfft?; stelle allerley bauchflüss
Potentilla sp.	Rosaceae	ren	LF	Fingerkraut	syst	GAS	[- CCCLV / CCXXXIX	gebresten der leber
Potentilla sp.	Rosaceae	ren	LF	Fingerkraut	syst	NER	[- CCCLV / CCXXXIX	heylen fallende sucht oder das vergicht
Potentilla sp.	Rosaceae	ren	LF	Fingerkraut	syst	RES	[- CCCLV / CCXXXIX	gebresten der lungen
Potentilla sp.	Rosaceae	ren	LF	Fingerkraut	syst	RES	[- CCCLV / CCXXXIX	und allerley gifft
Potentilla sp.	Rosaceae	ren	LF	Fingerkraut	top	SKE	[- CCCLV / CCXXXIX	heylen den bruch
Potentilla sp.	Rosaceae	ren	LF	Fingerkraut	syst	SKE	[- CCCLV / CCXXXIX	weetagen der gleych un hüfft;
Potentilla sp.	Rosaceae	ren	LF	Fingerkraut	top	TEE	[- CCCLV / CCXXXIX	im mund warm gehalten miltert den weetagen der zaen
Potentilla sp.	Rosaceae	s pop	UB	Blutwurz	top	DER	p. 303	Wundbehandlung
Potentilla sp.	Rosaceae	s pop	UB	Blutwurz	top	DER	p. 303	Wundbehandlung
Potentilla sp.	Rosaceae	s pop	UB	Blutwurz	syst	GAS	p. 121	Durchfall
Potentilla sp.	Rosaceae	s pop	UB	Blutwurz	syst	GAS	p. 121	Durchfall
Potentilla sp.	Rosaceae	s pop	UB	Blutwurz	syst	GAS	p. 121	Durchfall
Potentilla sp.	Rosaceae	s pop	UB	Blutwurz	syst	HUM	p. 460	Gerbstoffe zum Binden; Ausleitung und Regeneration
Potentilla sp.	Rosaceae	s pop	UB	Blutwurz	syst	HUM	p. 460	Gerbstoffe zum Binden; Ausleitung und Regeneration
Potentilla sp.	Rosaceae	s pop	UB	Blutwurz	syst	HUM	p. 460	Gerbstoffe zum Binden; Ausleitung und Regeneration
Potentilla sp.	Rosaceae	sci	EMA	Blutwurz	top	DER	A/HMPC/5513/2010	Traditional herbal medicinal product for the symptomatic treatment of minor inflammations of the oral mucosa.
Potentilla sp.	Rosaceae	sci	EMA	Blutwurz	top	DER	A/HMPC/5513/2010	Traditional herbal medicinal product for the symptomatic treatment of minor inflammations of the oral mucosa.
Potentilla sp.	Rosaceae	sci	EMA	Blutwurz	syst	GAS	A/HMPC/5513/2010	Traditional herbal medicinal product for symptomatic treatment of mild diarrhoea.
Potentilla sp.	Rosaceae	sci	EMA	Blutwurz	syst	GAS	A/HMPC/5513/2010	Traditional herbal medicinal product for symptomatic treatment of mild diarrhoea.
Prunus sp.	Rosaceae	ant	DIOS	Pflaume	top	DER	110	Mit Essig aber eingerieben heilt es die Flechte bei den Kindern
Prunus sp.	Rosaceae	ant	DIOS	Pflaume	syst	GAS	110	ist dem Magen sehr zuträglich und stellt den Durchfall
Prunus sp.	Rosaceae	ant	DIOS	Pflaume	syst	GAS	110	Affectionen des Zäpfchens, des Zahnfleisches und der Mandeln
Prunus sp.	Rosaceae	ant	DIOS	Schlehe	syst	GAS	110	eingekochtem Most gesotten wird sie dem Magen wohltuender und den Durchfall mehr hemmen
Prunus sp.	Rosaceae	ant	DIOS	Pflaume	syst	URO	110	den Stein zu zertrümmern

Prunus sp.	Rosaceae	mon	HvB	Pflaumenbaum	top	DER	3-7	und wenn irgendwelche Würmer das Fleisch des Menschen fressen
Prunus sp.	Rosaceae	mon	HvB	Pflaumenbaum	top	DER	3-7	und wenn Dein Kopf schuppig ist oder welkt, wasche ihn oft mit dieser Lauge
Prunus sp.	Rosaceae	mon	HvB	Schlehdorn	top	DER	3-53	wenn Krebs an ihm nagen, Geschwüre
Prunus sp.	Rosaceae	mon	HvB	Pflaumenbaum	top	DER	3-7	und wenn einem Menschen die Lippen seines Mundes anschwellen und sich aufblasen
Prunus sp.	Rosaceae	mon	HvB	Schlehdorn	syst	GAS	3-53	schwach im Magen
Prunus sp.	Rosaceae	mon	HvB	Pflaumenbaum	top	NER	3-7	und wenn jemand durch magische oder verwünschende Worte von Sinnen kommt, dann nimm Erde, die um die Wurzeln jenes Baumes liegt . . .
Prunus sp.	Rosaceae	mon	HvB	Schlehdorn	syst	NER	3-53	Gichtkrank, so dass ihm die Sinne schwinden und er davon verrückt wird
Prunus sp.	Rosaceae	mon	HvB	Pflaumenbaum	syst	RES	3-7	trockener Husten
Prunus sp.	Rosaceae	mon	HvB	Schlehdorn	syst	SKE	3-53	Gicht in ihm
Prunus sp.	Rosaceae	mon	HvB	Pflaumenbaum	top	SKE	3-7	aber wem die Finger und die Hände von der Gicht sich ständig zitternd bewegen
Prunus sp.	Rosaceae	ren	LF	Zwetschgenbaum	top	DER	CCXXVI / CLIII	seind gut zu dem geschwollen zaepflin unnd mandeln (dann sie treiben den Fluss hinter sich)
Prunus sp.	Rosaceae	ren	LF	Schwarzdorn	top	DER	CCXXVII / CLIII	heylt es die geflecht, zittern oder rauden der kinder
Prunus sp.	Rosaceae	ren	LF	Zwetschgenbaum	syst	GAS	CCXXVI / CLIII	lindern den bauch
Prunus sp.	Rosaceae	ren	LF	Schwarzdorn	syst	GAS	CCXXVII / CLIII	ziehen sehr zusammen und stellen den bauchfluss
Prunus sp.	Rosaceae	ren	LF	Schwarzdorn	syst	GAS	CCXXVII / CLIII	zermalt den stein
Prunus sp.	Rosaceae	s pop	BVA	Schlehdorn - Schwa	syst	DER	p. 189	Entschlackung bei Hautkrankheiten
Prunus sp.	Rosaceae	s pop	BVA	Schlehdorn - Schwa	top	DER	p. 189	Schleimhautentzündungen, entzündetem Zahnfleisch, Zahnfleischbuten
Prunus sp.	Rosaceae	s pop	BVA	Schlehdorn - Schwa	syst	GAS	p. 189	Verstopfung (Kinder), Verdauungsstörungen, Magenstärkung
Prunus sp.	Rosaceae	s pop	BVA	Schlehdorn - Schwa	syst	HUM	p. 189	Stärkung, Blutreinigung
Prunus sp.	Rosaceae	s pop	BVA	Schlehdorn - Schwa	top	TEE	p. 189	entzündetem Zahnfleisch, Zahnfleischbuten
Prunus sp.	Rosaceae	s pop	BVA	Schlehdorn - Schwa	syst	URO	p. 189	Schliessmuskelschwäche der Blase, Afters, Bettnässen
Prunus sp.	Rosaceae	s pop	JK	Schlehe	syst	GAS	p. 416	Durchfall
Prunus sp.	Rosaceae	s pop	JK	Schlehe	syst	SKE	p. 416	Gichtige
Prunus sp.	Rosaceae	s pop	KN	Schlehe	syst	GAS	p. 462	Abführmittel, Magenmittel
Prunus sp.	Rosaceae	s pop	UB	Schwarzdorn / Schl	syst	TON	p. 378	Tonikum zur Herzkräftigung, in der Rekonvaleszenz, beim Altersherz
Prunus sp.	Rosaceae	s pop	WI	Schlehe	syst	DER	p. 461	Volksmedizin: Gurgelmittel bei Mund- und Hals- und Zahnfleischentzündungen
Prunus sp.	Rosaceae	s pop	WI	Schlehe	top	DER	p. 461	Volksmedizin: Gurgelmittel bei Mund- und Hals- und Zahnfleischentzündungen
Prunus sp.	Rosaceae	s pop	WI	Schlehe	syst	GAS	p. 461	Volksmedizin: Magenschwäche
Prunus sp.	Rosaceae	s pop	WI	Schlehe	syst	RES	p. 461	Volksmedizin: Expektorans
Prunus sp.	Rosaceae	s pop	WI	Schlehe	syst	URO	p. 461	Volksmedizin: Diurektikum
Prunus sp.	Rosaceae	s pop	WI	Schlehe	syst	URO	p. 461	Volksmedizin: Purgans und Diuretikum
Prunus sp.	Rosaceae	s pop	WI	Schlehe	syst	URO	p. 461	Volksmedizin: Diurektikum
Pyrus communis aggr.	Rosaceae	ant	DIOS	Birne	syst	ANT	109	hilft kräftig bei Vergiftungen durch Pilze
Pyrus communis aggr.	Rosaceae	ant	DIOS	Birne	top	DER	109	eignen sie sich zu verteilenden Umschlägen
Pyrus communis aggr.	Rosaceae	ant	DIOS	Birne	syst	GAS	109	stellen den Durchfall
Pyrus communis aggr.	Rosaceae	mon	HvB	Birnbaum	syst	GAS	3-2	jedoch bewirken sie bei ihm eine gute Verdauung, weil sie die Fäulnis mit abführt
Pyrus communis aggr.	Rosaceae	mon	HvB	Birnbaum	syst	NER	3-2	weil es die Migräne wegnimmt
Pyrus communis aggr.	Rosaceae	mon	HvB	Birnbaum	syst	RES	3-2	die Dämpfigkeit mindert, welche die rohen Birnen in der Brust der Menschen verursachen
Pyrus communis aggr.	Rosaceae	mon	HvB	Birnbaum	syst	RES	3-2	Und alle üblichen Säfte, die im Menschen sind, vernichtet sie und reinigt den Mensche so, wie ein Geschirr vom Schmutz gereinigt wird
Pyrus communis aggr.	Rosaceae	ren	TAB	Birne	syst	GAS	n.d.	laben und erquicken die Krancken/ denen die Speiss zuwider ist/ besc
Pyrus communis aggr.	Rosaceae	s pop	UB	Birne	syst	URO	p. 246	wassertreibende Früchte
Pyrus communis aggr.	Rosaceae	s pop	UB	Birne	syst	URO	p. 248	harndesinfizierend
Quercus robur aggr.	Fagaceae	ant	DIOS	Eiche	syst	ANT	104	Sie wirken gegessen gegen giftige Tiere; auch ihre Abkochung und die der Rinde mit Kuhmilch getrunken hilft gegen Gift.
Quercus robur aggr.	Fagaceae	ant	DIOS	Eiche	top	DER	104	lindern sie als Umschlag Entzündungen, mit gesalzenem Schweinefett sind sie als Umschlag ein gutes Mittel gegen bösartige Verhärtungen und schlimme Geschwüre
Quercus robur aggr.	Fagaceae	ant	DIOS	Eiche	top	DER	104	Die Blätter aber verkleben frisch geschlagene Wunden
Quercus robur aggr.	Fagaceae	ant	DIOS	Eiche	top	DER	104	Die Blätter aber verkleben frisch geschlagene Wunden
Quercus robur aggr.	Fagaceae	ant	DIOS	Eiche	syst	GAS	104	die am Magen, an Dysenterie und an Blutspeien leiden
Quercus robur aggr.	Fagaceae	ant	DIOS	Eiche	top	GYN	104	den an Fluss leidenden Frauen
Quercus robur aggr.	Fagaceae	ant	DIOS	Eiche	syst	URO	104	sind sie harntreibend
Quercus robur aggr.	Fagaceae	mon	HvB	Eiche	n.d.	OTH	3-25	Für ein Heilmittel ist weder das Holz noch die Frucht zu gebrauchen
Quercus robur aggr.	Fagaceae	ren	LF	Steineiche	syst	ANT	CXXIX / LXXXIII	widerstehen allerley gifft
Quercus robur aggr.	Fagaceae	ren	LF	Steineiche	top	DER	CXXIX / LXXXIII	zu den boesen herten geschwulsten un allten schaeden
Quercus robur aggr.	Fagaceae	ren	LF	Steineiche	top	DER	CXXIX / LXXXIII	heylen allerley wunden
Quercus robur aggr.	Fagaceae	ren	LF	Steineiche	top	DER	CXXIX / LXXXIII	allerley flüss so sie auf das zanfleysch oder zaepflin fallen, verhindern mundfeule
Quercus robur aggr.	Fagaceae	ren	LF	Steineiche	top	DER	CXXIX / LXXXIII	färben Haar schwarz
Quercus robur aggr.	Fagaceae	ren	LF	Steineiche	syst	GAS	CXXIX / LXXXIII	rote rhur, das blutspeien, stellen den bauchfluss
Quercus robur aggr.	Fagaceae	ren	LF	Steineiche	syst	GAS	CXXIX / LXXXIII	roten rhur
Quercus robur aggr.	Fagaceae	ren	LF	Steineiche	syst	GYN	CXXIX / LXXXIII	stellen der frawen krankheyt
Quercus robur aggr.	Fagaceae	ren	LF	Steineiche	top	GYN	CXXIX / LXXXIII	verhindern das aufgehen der muter undd der weiber bloedigkeyt
Quercus robur aggr.	Fagaceae	ren	LF	Steineiche	top	OTH	CXXIX / LXXXIII	stellen das blut
Quercus robur aggr.	Fagaceae	ren	LF	Steineiche	syst	URO	CXXIX / LXXXIII	treiben den harn
Quercus robur aggr.	Fagaceae	s pop	JK	Eiche	top	DER	p. 345	Wunden, Geschwüren und Lupus
Quercus robur aggr.	Fagaceae	s pop	JK	Eiche	top	DER	p. 345	Angina und sonstige Halsleiden
Quercus robur aggr.	Fagaceae	s pop	JK	Eiche	syst	GAS	p. 345	andauerndem Durchfall, Blutspeien
Quercus robur aggr.	Fagaceae	s pop	JK	Eiche	syst	URO	p. 345	unfreiwilligem Wasserlösen, Blutfluss
Quercus robur aggr.	Fagaceae	s pop	KN	Eiche	top	DER	p. 463	verdrängen die dicken Hälse, Kropfmittel, mit den Drüsen räume diese Wickel nicht weniger gründlich auf
Quercus robur aggr.	Fagaceae	s pop	KN	Eiche	top	DER	p. 463	Mastdarmvorfall, Mastdarmfisteln, harte Geschwülste
Quercus robur aggr.	Fagaceae	s pop	KN	Eiche	top	DER	p. 463	oberflächliche Wunden, oberflächliche Ekzeme, Frostbeulen
Quercus robur aggr.	Fagaceae	s pop	KN	Eiche	syst	GAS	p. 463	katarrhalischen Zustände des Verdauungsapparates, so auf Durchfälle
Quercus robur aggr.	Fagaceae	s pop	KN	Eiche	top	TON	p. 412	skrophulöse Kinder
Quercus robur aggr.	Fagaceae	s pop	UB	Eiche	top	DER	p. 328	allgemeine Behanldung von Ekzemen, Neurodermitis, Psoriasis
Quercus robur aggr.	Fagaceae	s pop	UB	Eiche	top	DER	p. 328	allgemeine Behanldung von Ekzemen, Neurodermitis, Psoriasis
Quercus robur aggr.	Fagaceae	s pop	UB	Eiche	top	DER	p. 328	allgemeine Behanldung von Ekzemen, Neurodermitis, Psoriasis
Quercus robur aggr.	Fagaceae	s pop	UB	Eiche	syst	GAS	p. 122	Durchfallerkrankungen
Quercus robur aggr.	Fagaceae	sci	EMA	Eiche	top	DER	A/HMPC/3203/2009	Traditional herbal medicinal product for symptomatic treatment of minor inflammation of the oral mucosa or skin
Quercus robur aggr.	Fagaceae	sci	EMA	Eiche	top	DER	A/HMPC/3203/2009	Traditional herbal medicinal product for itching and burning haemorrhoids
Quercus robur aggr.	Fagaceae	sci	EMA	Eiche	syst	GAS	A/HMPC/3203/2009	Traditional herbal medicinal prduct for symptomatic treatment of mild diarrhea
Quercus robur aggr.	Fagaceae	sci	WI	Eiche	syst	GAS	p. 474	Stomachikum, bei unspezifischen Durchfallerkrankungen
Raphanus sativus L.	Brassicaceae	ant	DIOS	Rettich	syst	ANT	142	ebenso erweist er sich mit Wein getrunken heilsam gegen den Biss der Hornschlange
Raphanus sativus L.	Brassicaceae	ant	DIOS	Rettich	syst	ANT	142	Gegessen oder getrunken hilft sie bei denen, die durch Essen oder Trinken von (giftigen) Pilzen Erstickungsanfälle bekommen
Raphanus sativus L.	Brassicaceae	ant	DIOS	Rettich	top	ANT	142	Sie hilft den von giftigen Tieren Gebissenen
Raphanus sativus L.	Brassicaceae	ant	DIOS	Rettich	top	DER	142	Mit Essig aufgelegt endlich reißt er Gangrän kräftig rund herum auf
Raphanus sativus L.	Brassicaceae	ant	DIOS	Rettich	top	DER	142	Bei Entzündung der Schlundmuskeln (Angina) hilft er gekocht als Gurgelmittel mit warmem Sauerhonig

Raphanus sativus L.	Brassicaceae	ant	DIOS	Rettich	top	DER	142	Mit Honig bringt sie fressende Geschwüre zum Stillstand und entfernt Sugillationen unter den Augen; und erzeugt nach der Fuchskrankheit dichtes Haar, Mit Taumellolchmehl entfernt sie Leberflecken
Raphanus sativus L.	Brassicaceae	ant	DIOS	Rettich	syst	GAS	142	Er bekömmt auch dem Bauche, wenn man ihn dazu (d.h. nach der Mahlzeit) nimmt, indem er die Verteilung der Nahrungssäfte befördert;
Raphanus sativus L.	Brassicaceae	ant	DIOS	Rettich	syst	GYN	142	befördert auch die Menstruation
Raphanus sativus L.	Brassicaceae	ant	DIOS	Rettich	syst	HUM	142	Vorher genossen ist er auch bei denen angebracht, die erbrechen wollen
Raphanus sativus L.	Brassicaceae	ant	DIOS	Rettich	syst	NER	142	Er schärft aber auch die Sinne
Raphanus sativus L.	Brassicaceae	ant	DIOS	Rettich	syst	OTH	142	reinigt die Milz
Raphanus sativus L.	Brassicaceae	ant	DIOS	Rettich	top	OTH	142	Als Umschlag ist sie auch bei Milzkranken angebracht
Raphanus sativus L.	Brassicaceae	ant	DIOS	Rettich	syst	RES	142	an chronischem Husten leiden und bei denen sich Verdichtungen in der Brust gebildet haben
Raphanus sativus L.	Brassicaceae	ant	DIOS	Rettich	syst	URO	142	treibt den Harn
Raphanus sativus L.	Brassicaceae	ant	DIOS	Rettich	syst	URO	142	ist aber den Wassersüchtigen wohltuend
Raphanus sativus L.	Brassicaceae	mon	HvB	Rettich	syst	GAS	1-89	vermindert die schädlichen Säfte der Eingeweide
Raphanus sativus L.	Brassicaceae	mon	HvB	Rettich	syst	HUM	1-89	Aber wer viel Schleim in sich hat . . .
Raphanus sativus L.	Brassicaceae	mon	HvB	Rettich	syst	NER	1-89	reinigt er das Gehirn
Raphanus sativus L.	Brassicaceae	ren	LF	Rettich	n.d.	ANT	CCCLXXV / CCLVI	gut denen von natern gestochen seind, bekommt wohl, denen so toedtlich pfifferling gegessen haben
Raphanus sativus L.	Brassicaceae	ren	LF	Rettich	syst	ANT	CCCLXXV / CCLVI	ist gut wider das gifft
Raphanus sativus L.	Brassicaceae	ren	LF	Rettich	syst	CAR	CCCLXXV / CCLVI	er ist den wasserüschtigen gut
Raphanus sativus L.	Brassicaceae	ren	LF	Rettich	top	DER	CCCLXXV / CCLVI	heylet er die umb sich fressenden schaeden, verzert auch das undergrunnen blut, macht har wachsen
Raphanus sativus L.	Brassicaceae	ren	LF	Rettich	top	DER	CCCLXXV / CCLVI	vertreibt er allerley maeler und flecken under dem angesicht
Raphanus sativus L.	Brassicaceae	ren	LF	Rettich	top	DER	CCCLXXV / CCLVI	vertreibt die schwarzen maeler und flecken am leib
Raphanus sativus L.	Brassicaceae	ren	LF	Rettich	top	DER	CCCLXXV / CCLVI	hals geschwaer
Raphanus sativus L.	Brassicaceae	ren	LF	Rettich	syst	GAS	CCCLXXV / CCLVI	macht speien und kotzen
Raphanus sativus L.	Brassicaceae	ren	LF	Rettich	syst	GAS	CCCLXXV / CCLVI	nach dem essen genommen würt, hilfft er zu der aufteylung der speiss in die glider, vor dem essen ist er nützlich denen so undewen wollen
Raphanus sativus L.	Brassicaceae	ren	LF	Rettich	syst	GAS	CCCLXXV / CCLVI	macht speien und treibt den harn
Raphanus sativus L.	Brassicaceae	ren	LF	Rettich	top	OTH	CCCLXXV / CCLVI	den milzsüchtigen
Raphanus sativus L.	Brassicaceae	ren	LF	Rettich	syst	OTH	CCCLXXV / CCLVI	macht er das milz kleiner
Raphanus sativus L.	Brassicaceae	ren	LF	Rettich	syst	RES	CCCLXXV / CCLVI	bekompt er wol dem langwerigen husten, zerteylet den zaehen schleim
Raphanus sativus L.	Brassicaceae	ren	LF	Rettich	syst	URO	CCCLXXV / CCLVI	und treibt den harn
Raphanus sativus L.	Brassicaceae	ren	LF	Rettich	syst	URO	CCCLXXV / CCLVI	zermalt den stein unnd treibt denselben aus
Raphanus sativus L.	Brassicaceae	s pop	UB	Rettich	syst	GAS	p. 184	Gallenstörungen
Raphanus sativus L.	Brassicaceae	s pop	UB	Rettich	syst	GAS	p. 184	Gallenstörungen
Raphanus sativus L.	Brassicaceae	s pop	UB	Rettich	syst	RES	p. 219	Katarrhe der Atemwege
Rosa sp.	Rosaceae	ant	DIOS	Rosen	syst	ANT	101	bei Fluss des Zahnfleisches
Rosa sp.	Rosaceae	ant	DIOS	Rosen	syst	ANT	101	auch werden sie den kräftigend und wundenheilenden Gegenmitteln zugesetzt
Rosa sp.	Rosaceae	ant	DIOS	Rose	top	DER	82	ist ein Nährmittel für hohle Geschwüre und besänftigt die bösartigen, es ist ein Mittel gegen Schorf und Ausschlag; gegen Verhärtungen der Augenlider
Rosa sp.	Rosaceae	ant	DIOS	Rosen	top	DER	101	Zahnfleisch-, After- [d.h. Mastdarm]
Rosa sp.	Rosaceae	ant	DIOS	Rosen	top	DER	101	roseartige Hautentzündungen,
Rosa sp.	Rosaceae	ant	DIOS	Rosen	top	DER	101	trocken aber und fein gerieben werden sie auf die innere Seite der Hüften gestreut
Rosa sp.	Rosaceae	ant	DIOS	Rosen	top	EAR	101	Ohren-
Rosa sp.	Rosaceae	ant	DIOS	Rosen	top	EYE	101	um die Augen zu verschönern
Rosa sp.	Rosaceae	ant	DIOS	Rosen	top	EYE	101	zum Einsalben der Augen
Rosa sp.	Rosaceae	ant	DIOS	Rosen	top	EYE	101	tut gut bei Kopfschmerzen
Rosa sp.	Rosaceae	ant	DIOS	Rosen	top	EYE	101	bei Augenschmerzen
Rosa sp.	Rosaceae	ant	DIOS	Rose	syst	GAS	82	Leib und mildert die Hitze des Magens,
Rosa sp.	Rosaceae	ant	DIOS	Rose	top	GAS	82	als Spülung gegen Zahnschmerzen
Rosa sp.	Rosaceae	ant	DIOS	Hundsrose	syst	GAS	99	stellt den Bauchfluss
Rosa sp.	Rosaceae	ant	DIOS	Rosen	syst	GAS	101	Bauchfluss und das Blutspeien auf
Rosa sp.	Rosaceae	ant	DIOS	Rosen	top	GAS	101	Magenfäule
Rosa sp.	Rosaceae	ant	DIOS	Rose	top	GYN	82	ist als Klysiter mit Erfolg zu gebrauchen gegen Reize der Eingeweide und der Gebärmutter
Rosa sp.	Rosaceae	ant	DIOS	Rosen	top	GYN	101	Mutterschmerzen
Rosa sp.	Rosaceae	ant	DIOS	Rosen	top	GYN	101	helfen gegen Unterleibsentzündungen
Rosa sp.	Rosaceae	ant	DIOS	Rose	top	NER	82	Anfang als Umschlag gegen Kopfschmerzen,
Rosa sp.	Rosaceae	mon	HvB	Hagrose	top	DER	3-52	der wasche oft seinen Kopf damit, und er wird dann gesund und stark
Rosa sp.	Rosaceae	mon	HvB	Hagrose	syst	GAS	3-52	und nur einen schwachen Magen hat
Rosa sp.	Rosaceae	mon	HvB	Rose	top	NER	1-22	jähzornig
Rosa sp.	Rosaceae	mon	HvB	Hagrose	syst	RES	3-52	und wer in der Lunge leidet
Rosa sp.	Rosaceae	mon	HvB	Rose	top	SKE	1-22	Krampf, Lähmung
Rosa sp.	Rosaceae	mon	MF	Rose	top	DER	21	Krankheiten des Mundes
Rosa sp.	Rosaceae	mon	MF	Rose	top	DER	21	reinigt es eiternde Verletzungen und füllt klaffende Wunden, hilft auch bei Brandwunden
Rosa sp.	Rosaceae	mon	MF	Rose	top	DER	21	beseitigt Darmjucken
Rosa sp.	Rosaceae	mon	MF	Rose	top	EYE	21	Augenlider erweichen
Rosa sp.	Rosaceae	mon	MF	Rose	n.d.	GAS	21	Erhitzung im Magen, mindert Durchfall
Rosa sp.	Rosaceae	mon	MF	Rose	syst	GAS	21	erweicht es den harten Leib und tilgt grosse Hitze des Magens
Rosa sp.	Rosaceae	mon	MF	Rose	n.d.	GYN	21	mindert sie Ausfluss der Gebärmutter
Rosa sp.	Rosaceae	mon	MF	Rose	top	GYN	21	kommt auch Krankheitsfällen der Gebärmutter zugute
Rosa sp.	Rosaceae	mon	MF	Rose	top	NER	21	Schmerzen und Hitze des Hauptes geheilt
Rosa sp.	Rosaceae	mon	MF	Rose	top	OTH	21	zähmt sie Antoniusfeuer
Rosa sp.	Rosaceae	mon	MF	Rose	top	TEE	21	Zahnschmerz lindern
Rosa sp.	Rosaceae	ren	LF	Rosen	top	DER	CCCLXXIIII / CCLV	verhindert das einem das har nit ausfelt
Rosa sp.	Rosaceae	ren	LF	Rosen	top	DER	CCCLXXIIII / CCLV	zahnfleyschs, affters
Rosa sp.	Rosaceae	ren	LF	Rosen	top	DER	CCCLXXIIII / CCLV	weetagen der daerm und muter
Rosa sp.	Rosaceae	ren	LF	Rosen	top	DER	CCCLXXIIII / CCLV	rotlauff
Rosa sp.	Rosaceae	ren	LF	Rosen	top	DER	CCCLXXIIII / CCLV	in die wunden gestrewt
Rosa sp.	Rosaceae	ren	LF	Rosen	top	DER	CCCLXXIIII / CCLV	feücht zanfleysch gethon trücknet dasselbige krefftiglich
Rosa sp.	Rosaceae	ren	LF	Rosen	top	EAR	CCCLXXIIII / CCLV	weetagen der ohren
Rosa sp.	Rosaceae	ren	LF	Rosen	top	EYE	CCCLXXIIII / CCLV	weetagen der augen
Rosa sp.	Rosaceae	ren	LF	Rosen	syst	FEV	CCCLXXIIII / CCLV	gut zu den hitzigen febern
Rosa sp.	Rosaceae	ren	LF	Rosen	syst	GAS	CCCLXXIIII / CCLV	stellen den bauchfluss un das blutspeien
Rosa sp.	Rosaceae	ren	LF	Rosen	syst	GAS	CCCLXXIIII / CCLV	krefftiget seer den magen, die leber
Rosa sp.	Rosaceae	ren	LF	Rosen	top	GAS	CCCLXXIIII / CCLV	legen und miltern die hitz so sich umb die leber oder das miltz ereyget, verzere die überige feüchtigkeit des magens
Rosa sp.	Rosaceae	ren	LF	Rosen	syst	GYN	CCCLXXIIII / CCLV	stellen die der frawen zeit in soderheyt aber den weissen fluss und das blutspeien(?)
Rosa sp.	Rosaceae	ren	LF	Rosen	top	NER	CCCLXXIIII / CCLV	weetagen des haupts
Rosa sp.	Rosaceae	ren	LF	Rosen	top	NER	CCCLXXIIII / CCLV	bringen den schlaaf
Rosa sp.	Rosaceae	ren	LF	Rosen	syst	URO	CCCLXXIIII / CCLV	treiben den harn und den lendenstein
Rosa sp.	Rosaceae	s pop	BVA	Heckenrose	syst	GYN	p. 84	stillende Mütter
Rosa sp.	Rosaceae	s pop	BVA	Heckenrose	syst	HUM	p. 84	Blutreinigung
Rosa sp.	Rosaceae	s pop	BVA	Heckenrose	syst	TEE	p. 84	Mund- und Zahnwasser, entzündetliches, leichtblutendes Zahnfleisch
Rosa sp.	Rosaceae	s pop	BVA	Heckenrose	syst	TEE	p. 84	Zahnfleischbluten
Rosa sp.	Rosaceae	s pop	BVA	Heckenrose	syst	TON	p. 84	Steigerung der Abwehrkräfte, Fieber
Rosa sp.	Rosaceae	s pop	BVA	Heckenrose	syst	URO	p. 84	Nierenfunktionsschwäche, Griess- und Stienbildung in Niere und Blase
Rosa sp.	Rosaceae	s pop	JK	Rose	top	DER	p. 406	Gesichtsrose

Rosa sp.	Rosaceae	s pop	JK	Rose	syst	EAR	p. 406	Schmerzen der Ohren
Rosa sp.	Rosaceae	s pop	JK	Rose	top	EAR	p. 406	Schmerzen der Ohren
Rosa sp.	Rosaceae	s pop	JK	Rose	syst	EYE	p. 406	Schmerzen der Augen
Rosa sp.	Rosaceae	s pop	JK	Rose	top	EYE	p. 406	Schmerzen der Augen
Rosa sp.	Rosaceae	s pop	JK	Rose	syst	FEV	p. 406	Fieber zu brechen
Rosa sp.	Rosaceae	s pop	JK	Rose	syst	GAS	p. 406	Würmer und Durchfall
Rosa sp.	Rosaceae	s pop	JK	Rose	syst	GYN	p. 406	Schmerzen des Unterleibs
Rosa sp.	Rosaceae	s pop	JK	Rose	top	GYN	p. 406	Schmerzen des Unterleibs
Rosa sp.	Rosaceae	s pop	JK	Hundsrose	syst	GYN	p. 366	Unterleibstockungen
Rosa sp.	Rosaceae	s pop	JK	Hundsrose	syst	GYN	p. 366	Unterleibstockungen
Rosa sp.	Rosaceae	s pop	JK	Hundsrose	syst	n.d.	p. 366	Brust- und Seitenstechen und inneren Brand
Rosa sp.	Rosaceae	s pop	JK	Hundsrose	syst	n.d.	p. 366	Brust- und Seitenstechen und inneren Brand
Rosa sp.	Rosaceae	s pop	JK	Rose	syst	NER	p. 406	Nerven zu stärken
Rosa sp.	Rosaceae	s pop	JK	Hundsrose	syst	SKE	p. 366	Rhemumatismus, Gicht und Ischias
Rosa sp.	Rosaceae	s pop	JK	Rose	syst	TEE	p. 406	Schmerzen der Zähne
Rosa sp.	Rosaceae	s pop	JK	Rose	top	TEE	p. 406	Schmerzen der Zähne
Rosa sp.	Rosaceae	s pop	JK	Rose	syst	URO	p. 406	fördert den Urinabgang
Rosa sp.	Rosaceae	s pop	JK	Hundsrose	syst	URO	p. 366	Nieren- und Blasenleiden
Rosa sp.	Rosaceae	s pop	JK	Hundsrose	syst	URO	p. 366	Nieren- und Blasenleiden
Rosa sp.	Rosaceae	s pop	JK	Hundsrose	syst	URO	p. 366	Wassersucht
Rosa sp.	Rosaceae	s pop	KN	Hundsrose	syst	URO	p. 468	Griess oder an Nieren- und Blasensteinen
Rosa sp.	Rosaceae	s pop	UB	Rose	syst	CAR	p. 378	psychogenen Herzleiden
Rosa sp.	Rosaceae	s pop	WI	Hagebutte	syst	GAS	p. 503	Volksmedizin: laxierend, Darmerkrankungen usw.
Rosa sp.	Rosaceae	s pop	WI	Hagebutte	syst	URO	p. 503	Volksmedizin: diuretisch
Rosa sp.	Rosaceae	s pop	WI	Hagebutte	syst	URO	p. 503	Volksmedizin: Diuretikum bei Nieren- und Blasenerkrankungen, bei Steinleiden
Rosa sp.	Rosaceae	sci	EMA	Rose	top	DER	HMPC/137299/2013	Traditional herbal medicinal product used for mild inflammations of the oral and pharyngeal mucosa.
Rosa sp.	Rosaceae	sci	EMA	Rose	top	DER	HMPC/137299/2013	Traditional herbal medicinal product used for relief of minor skin inflammation.
Rosa sp.	Rosaceae	sci	ESCOP	Rosae pseudo-fruct	syst	RES	II p. 217	as a supplement in the treatment of colds and chills
Rosa sp.	Rosaceae	sci	ESCOP	Rosae pseudo-fruct	syst	SKE	II p. 217	Adjuvant for the alleviation if pain and joint stiffness associated with osteoarthritis
Rosa sp.	Rosaceae	sci	WI	Hagebutte	syst	OTH	p. 503	Geschmackskorrigens
Rosa sp.	Rosaceae	sci	WI	Hagebutte	syst	RES	p. 503	Vorbeugung und Behandlung von Erkältungskrankheiten, grippalen Infekten
Rosa sp.	Rosaceae	sci	WI	Hagebutte	syst	RES	p. 503	Unterstützung der Therapie bei Vit. C Mangel
Rubia tinctorum L.	Rubiaceae	ant	DIOS	Färberwaid / Krapp	syst	ANT	208	hilft gegen den Biss giftiger Thiere
Rubia tinctorum L.	Rubiaceae	ant	DIOS	Färberwaid / Krapp	top	DER	208	heilt sie weisse Flecken
Rubia tinctorum L.	Rubiaceae	ant	DIOS	Färberwaid / Krapp	syst	GAS	208	bei Gelbsucht
Rubia tinctorum L.	Rubiaceae	ant	DIOS	Färberwaid / Krapp	top	GYN	208	befördert die Wurzel die Menstruation und die Nachgeburt
Rubia tinctorum L.	Rubiaceae	ant	DIOS	Färberwaid / Krapp	syst	OTH	208	erweicht die Milz
Rubia tinctorum L.	Rubiaceae	ant	DIOS	Färberwaid / Krapp	syst	SKE	208	Ischias und Paralyse
Rubia tinctorum L.	Rubiaceae	ant	DIOS	Färberwaid / Krapp	syst	URO	208	Sie treibt aber reichlichen und dicken Harn, oft ist es auch Blut
Rubia tinctorum L.	Rubiaceae	mon	HvB	Färberwaid	top	NER	1-208	ein Mensch, der in seinem Körper von irgendeiner Lähmung, von welcher Stärke sie auch sein möge
Rubia tinctorum L.	Rubiaceae	ren	LF	Färberröte	syst	ANT	CLV / CVVII	denen so von gifftigen thier gebissen seind
Rubia tinctorum L.	Rubiaceae	ren	LF	Färberröte	syst	ANT	CLV / CVVII	denen so von gifftigen thier gebissen seind
Rubia tinctorum L.	Rubiaceae	ren	LF	Färberröte	top	DER	CLV / CVVII	heylet sie allerley maeler und flecken der haut als seind die flechten und zittermaeler
Rubia tinctorum L.	Rubiaceae	ren	LF	Färberröte	syst	GAS	CLV / CVVII	reyniget die leber, miltz, nieren unnd mutter. Ist ein koestlich tranck zu der geelsucht
Rubia tinctorum L.	Rubiaceae	ren	LF	Färberröte	syst	GYN	CLV / CVVII	bring den frawen ihre krankheyt
Rubia tinctorum L.	Rubiaceae	ren	LF	Färberröte	top	GYN	CLV / CVVII	bringt sie den frawen ich krankheyt und zeücht heraus das nachbürdlin und die todten geburt
Rubia tinctorum L.	Rubiaceae	ren	LF	Färberröte	syst	OTH	CLV / CVVII	macht das milz klein
Rubia tinctorum L.	Rubiaceae	ren	LF	Färberröte	syst	SKE	CLV / CVVII	wol den hüfftsüchtigen unnd denen so lam in glidern seind
Rubia tinctorum L.	Rubiaceae	ren	LF	Färberröte	top	SKE	CLV / CVVII	wol den hüfftsüchtigen unnd denen so lam in glidern seind
Rubia tinctorum L.	Rubiaceae	ren	LF	Färberröte	syst	URO	CLV / CVVII	treibt den groben harn vielfeltig und mit gewalt also das auch zu zeiten das blut hernach geet
Rubia tinctorum L.	Rubiaceae	s pop	VAL	Färberröte	syst	GAS	p. 272	insufficance biliaire, constipation,
Rubia tinctorum L.	Rubiaceae	s pop	VAL	Färberröte	syst	URO	p. 272	oliguries, excès d'urée, lithiase urinaire
Rubus idaeus L.	Rosaceae	ant	DIOS	Himbeere	top	CAR	221	bei [Magen-] und Herzkrankheiten
Rubus idaeus L.	Rosaceae	ant	DIOS	Himbeere	top	DER	221	eignet sich zu Mundmitteln
Rubus idaeus L.	Rosaceae	ant	DIOS	Himbeere	top	DER	221	kräftigen das Zahnfleisch und heilen Soor.
Rubus idaeus L.	Rosaceae	ant	DIOS	Himbeere	top	DER	221	halten die Blätter als Umschlag kriechende Geschwüre auf und heilen Kopfgrind, das Vorfallen der Augen, Feigwarzen und Hämorrhoiden
Rubus idaeus L.	Rosaceae	ant	DIOS	Himbeere	top	EYE	221	Augenentzündungen
Rubus idaeus L.	Rosaceae	ant	DIOS	Himbeere	top	EYE	221	und lindert roseartige Entzündungen
Rubus idaeus L.	Rosaceae	ant	DIOS	Himbeere	syst	GAS	221	Magenleidenden
Rubus idaeus L.	Rosaceae	ant	DIOS	Himbeere	syst	GAS	221	nicht ganz ausgereifte Frucht stellt genossen den Bauch
Rubus idaeus L.	Rosaceae	ant	DIOS	Himbeere	top	GAS	221	bei Magen- [und Herzkrankheiten]
Rubus idaeus L.	Rosaceae	ant	DIOS	Himbeere	syst	GAS	221	stellt den Bauch
Rubus idaeus L.	Rosaceae	ant	DIOS	Himbeere	syst	GYN	221	und hält den Fluss der Frauen auf
Rubus idaeus L.	Rosaceae	ant	DIOS	Himbeere	syst	n.d.	221	heilt auch den Biss des Prester
Rubus idaeus L.	Rosaceae	mon	LO	Himbeere	syst	OTH	n.d.	n.d.
Rubus idaeus L.	Rosaceae	ren	LON	Himbeere	top	DER	n.d.	Mundgeschwür
Rubus idaeus L.	Rosaceae	ren	LON	Himbeere	syst	GAS	n.d.	rote rhur, bauchfluss
Rubus idaeus L.	Rosaceae	s pop	JK	Himbeere	syst	FEV	p. 372	innere Hitze mit Fieber
Rubus idaeus L.	Rosaceae	s pop	JK	Himbeere	syst	RES	p. 372	Lungenentzündung, Brustfellentzündung, Grippe
Rubus idaeus L.	Rosaceae	s pop	WI	Himbeere	top	DER	p. 512	Volksmedizin: adstringens bei Entzündungen im Mund und Rachenraum, bei chronischen Hauterkrankungen
Rubus idaeus L.	Rosaceae	s pop	WI	Himbeere	syst	GAS	p. 512	Volksmedizin: Antidiarrhoikum
Rubus idaeus L.	Rosaceae	s pop	WI	Himbeere	syst	GYN	p. 512	Volksmedizin:"Gynäkologikum"
Rubus idaeus L.	Rosaceae	sci	EMA	Himbeere	top	DER	/HMPC/44211/2012	Traditional herbal medicinal product for the symptomatic treatment of mild inflammation in the mouth or throat.
Rubus idaeus L.	Rosaceae	sci	EMA	Himbeere	syst	GAS	/HMPC/44211/2012	Traditional herbal medicinal product for the symptomatic treatment of mild diarrhoea.
Rubus idaeus L.	Rosaceae	sci	EMA	Himbeere	syst	GYN	/HMPC/44211/2012	Traditional herbal medicinal product for the symptomatic relief of minor spasm associated with menstrual periods.
Rumex spp.	Polygonaceae	ant	DIOS	Ampfer	syst	ANT	143	gegen Skorpionstich getrunken
Rumex spp.	Polygonaceae	ant	DIOS	Ampfer	syst	ANT	143	helfen gegen den Bisse des Skorpions
Rumex spp.	Polygonaceae	ant	DIOS	Ampfer	syst	APO	143	ihn vorher nimmt, hat er vom Stich nichts zu fürchten
Rumex spp.	Polygonaceae	ant	DIOS	Ampfer	other	APO	143	Wurzeln auch als Amulett gegen Drüsen an, indem sie dieselben um den Hals binden
Rumex spp.	Polygonaceae	ant	DIOS	Ampfer	top	DER	143	zerteilt er bösen Kopfausschlag
Rumex spp.	Polygonaceae	ant	DIOS	Ampfer	top	DER	143	heilen Aussatz, Flechten und schuppige Nägel
Rumex spp.	Polygonaceae	ant	DIOS	Ampfer	top	DER	143	Zusatz zum Bade beruhigt auch das Jucken
Rumex spp.	Polygonaceae	ant	DIOS	Ampfer	top	DER	143	sie zerteilen Drüsen am Halse und neben den Ohren als Umschlag
Rumex spp.	Polygonaceae	ant	DIOS	Ampfer	top	EAR	143	als Mundwasser Ohren- [und Zahnschmerzen]
Rumex spp.	Polygonaceae	ant	DIOS	Ampfer	syst	GAS	143	Das von allen gekochte Gemüse erweicht den Bauch
Rumex spp.	Polygonaceae	ant	DIOS	Ampfer	syst	GAS	143	gegen Dysenterie, krankhafte Verdauungszustände, Übelkeit aus dem Magen
Rumex spp.	Polygonaceae	ant	DIOS	Ampfer	syst	GAS	143	vollständig die Gelb- sucht
Rumex spp.	Polygonaceae	ant	DIOS	Ampfer	syst	GYN	143	befördern die Menstruation
Rumex spp.	Polygonaceae	ant	DIOS	Ampfer	top	GYN	143	stellen sie den Fluss der Frauen
Rumex spp.	Polygonaceae	ant	DIOS	Ampfer	top	OTH	143	wenn mit Essig (sind sie gut für) die Milz
Rumex spp.	Polygonaceae	ant	DIOS	Ampfer	top	TEE	143	als Mundwasser [Ohren-] und Zahnschmerzen
Rumex spp.	Polygonaceae	ant	DIOS	Ampfer	syst	URO	143	zertrümmern den Stein in der Blase
Rumex spp.	Polygonaceae	mon	LO	Ampfer	n.d.	OTH	n.d.	n.d.
Rumex spp.	Polygonaceae	mon	MF	Ampfer	top	DER	63	beissendes Jucken wie auch die Krätze
Rumex spp.	Polygonaceae	mon	MF	Ampfer	top	DER	63	beruhigt das geschwollene Rachenzäpfchen
Rumex spp.	Polygonaceae	mon	MF	Ampfer	top	DER	63	böse Halsdrüsen wie auch Mumpf

Rumex spp.	Polygonaceae	mon	MF	Ampfer	top	EAR	63	vertreibt den Ohrenschmerz
Rumex spp.	Polygonaceae	mon	MF	Ampfer	syst	GAS	63	stärkt S.A. den Magen und pflegt auch Windblähungen
Rumex spp.	Polygonaceae	mon	MF	Ampfer	syst	GAS	63	Blutstuhl und Bauchgrimmen
Rumex spp.	Polygonaceae	mon	MF	Ampfer	syst	GAS	63	ebenso holft sie den Gelbsüchtigen
Rumex spp.	Polygonaceae	mon	MF	Ampfer	syst	GYN	63	bezähmt den Monatsfluss
Rumex spp.	Polygonaceae	mon	MF	Ampfer	other	OTH	63	Die Wurzel um den Hals gehängt, soll böse Halsdrüsen schon am Entstehen hindern.
Rumex spp.	Polygonaceae	mon	MF	Ampfer	top	OTH	63	geschwollenen Milz
Rumex spp.	Polygonaceae	mon	MF	Ampfer	top	TEE	63	beruhigt [das geschwollene Rachenzäpfchen] und den Zahnschmerz
Rumex spp.	Polygonaceae	mon	MF	Ampfer	syst	URO	63	treibt auch Steine aus
Rumex spp.	Polygonaceae	ren	LF	Sauerampfer	syst	ANT	IX - CCLXII / CLXXV	ist gut denen so von einem scorpion gestochen seind
Rumex spp.	Polygonaceae	ren	LF	Sauerampfer	top	DER	IX - CCLXII / CLXXV	vewrzeren sie due geschwulst
Rumex spp.	Polygonaceae	ren	LF	Sauerampfer	syst	GAS	IX - CCLXII / CLXXV	machen ein linden bauch un stulgang
Rumex spp.	Polygonaceae	ren	LF	Sauerampfer	syst	GAS	IX - CCLXII / CLXXV	heylet die roten rhur, stellet den bauchfluss, das grawen
Rumex spp.	Polygonaceae	ren	LF	Sauerampfer	syst	OTH	IX - CCLXII / CLXXV	wan einer solchen tranck zuvor nimpt ist er sicher das im solches stechen nit schadet
Rumex spp.	Polygonaceae	s pop	VAL	Ampfer	syst	DER	p. 405	dermatose (dartres, eczéma, teigne)
Rumex spp.	Polygonaceae	s pop	VAL	Ampfer	top	DER	p. 405	dermatose (dartres, eczéma, teigne)
Rumex spp.	Polygonaceae	s pop	VAL	Ampfer	syst	GAS	p. 405	hépatisme
Rumex spp.	Polygonaceae	s pop	VAL	Ampfer	top	GYN	p. 405	leucorrhée
Rumex spp.	Polygonaceae	s pop	VAL	Ampfer	syst	OTH	p. 405	anémies, chlorose, diabète
Rumex spp.	Polygonaceae	s pop	VAL	Ampfer	syst	SKE	p. 405	thumatisme chronique
Ruta graveolens L.	Rutaceae	ant	DIOS	Raute	syst	ANT	180	ein Gegenmittel gegen Gifte
Ruta graveolens L.	Rutaceae	ant	DIOS	Raute	top	DER	180	heilt er roseartige Entzündungen, kriechende Geschwüre und bösen Grind
Ruta graveolens L.	Rutaceae	ant	DIOS	Raute	top	DER	180	aufgestrichen bessert es Flechten
Ruta graveolens L.	Rutaceae	ant	DIOS	Raute	top	DER	180	denselben Mitteln entfernt es im Umschlag Feigwarzen und gewöhnliche Warzen
Ruta graveolens L.	Rutaceae	ant	DIOS	Raute	top	DER	180	als Pulver eingeführt stillt es Nasenbluten
Ruta graveolens L.	Rutaceae	ant	DIOS	Raute	top	DER	180	Hodenentzündungen
Ruta graveolens L.	Rutaceae	ant	DIOS	Raute	top	DER	180	Hautausschlag mit Myrtenwachssalbe
Ruta graveolens L.	Rutaceae	ant	DIOS	Raute	top	DER	180	heilt es die weiße Ventiligo, und mit denselben Mitteln entfernt es im Umschlag Feigwarzen und gewöhnliche Warzen
Ruta graveolens L.	Rutaceae	ant	DIOS	Raute	top	EAR	180	Mittel bei Ohrenschmerzen
Ruta graveolens L.	Rutaceae	ant	DIOS	Raute	syst	EYE	180	Schärfe des Gesichts
Ruta graveolens L.	Rutaceae	ant	DIOS	Raute	top	EYE	180	mit Graupen im Kataplasma lindert es heftige Augenschmerzen
Ruta graveolens L.	Rutaceae	ant	DIOS	Raute	top	EYE	180	Stumpfsichtigkeit
Ruta graveolens L.	Rutaceae	ant	DIOS	Raute	syst	FEV	180	gegen periodische Frostschauer
Ruta graveolens L.	Rutaceae	ant	DIOS	Raute	syst	GAS	180	machen tödlichen Gifte unwirksam, In derselben Weise genommen sind sie ein gutes Mittel gegen Schlangenbisse
Ruta graveolens L.	Rutaceae	ant	DIOS	Raute	syst	GAS	180	gegessen sowohl wie getrunken stellen sie den Durchfall
Ruta graveolens L.	Rutaceae	ant	DIOS	Raute	syst	GAS	180	wirft es den Bandwurm hinaus
Ruta graveolens L.	Rutaceae	ant	DIOS	Raute	syst	GAS	180	Den Geruch und die Schärfe von Lauch und Zwiebeln mildert
Ruta graveolens L.	Rutaceae	ant	DIOS	Raute	syst	GAS	180	beruhigt es Leibschneiden, dann auch wirkt es gegen Seiten-
Ruta graveolens L.	Rutaceae	ant	DIOS	Raute	top	GAS	180	ferner gegen Aufblähen des Magens, [der Gebärmutter] und des Rektums mit Öl gekocht als Injektion
Ruta graveolens L.	Rutaceae	ant	DIOS	Raute	syst	GYN	180	vernichtet das Peganon die Leibesfrucht
Ruta graveolens L.	Rutaceae	ant	DIOS	Raute	syst	GYN	180	ferner gegen Aufblähen [des Magens], der Gebärmutter [und des Rektums mit Öl gekocht] als Injektion
Ruta graveolens L.	Rutaceae	ant	DIOS	Raute	top	GYN	180	Gebärmutterkrämpfe
Ruta graveolens L.	Rutaceae	ant	DIOS	Raute	top	NER	180	Rosensalbe und Essig hilft es bei Kopfschmerzen,
Ruta graveolens L.	Rutaceae	ant	DIOS	Raute	syst	RES	180	Brustschmerz, Atemnot, Husten, Brustfellentzündung
Ruta graveolens L.	Rutaceae	ant	DIOS	Raute	syst	SKE	180	Ischias- und Gelenkschmerzen
Ruta graveolens L.	Rutaceae	ant	DIOS	Raute	top	SKE	180	Gelenkschmerzen
Ruta graveolens L.	Rutaceae	ant	DIOS	Raute	syst	URO	180	mit Feigen gegen das unter dem Fleische gebildete Wasser
Ruta graveolens L.	Rutaceae	ant	DIOS	Raute	top	URO	180	mit Feigen gegen das unter dem Fleische gebildete Wasser
Ruta graveolens L.	Rutaceae	ant	DIOS	Raute	syst	URO	180	der den Harn nicht halten kann, und ihm wird geholfen
Ruta graveolens L.	Rutaceae	mon	HvB	Weinraute	top	EYE	1-63	auch ein Mensch, der triefende Augen hat,
Ruta graveolens L.	Rutaceae	mon	HvB	Weinraute	syst	GAS	1-63	auch wenn jemand eine andere Speise gegessen hat, wovon es ihn schmerzt
Ruta graveolens L.	Rutaceae	mon	MF	Raute	syst	ANT	7	widersteht man den Giften (Mithridates)
Ruta graveolens L.	Rutaceae	mon	MF	Raute	syst	APH	7	zähmt Liebesgier
Ruta graveolens L.	Rutaceae	mon	MF	Raute	syst	DER	7	räudige Krätze, Kopfgrundflechte, in der Nase entstehende Borken
Ruta graveolens L.	Rutaceae	mon	MF	Raute	syst	DER	7	stillt das ausströmende Blut (Nase) sehr gut
Ruta graveolens L.	Rutaceae	mon	MF	Raute	top	EAR	7	vertreibst du Ohrenschmerzen durch Einträufeln
Ruta graveolens L.	Rutaceae	mon	MF	Raute	syst	EYE	7	macht sie trübe Augen hellsichtig
Ruta graveolens L.	Rutaceae	mon	MF	Raute	syst	EYE	7	macht sie trübe Augen hellsichtig
Ruta graveolens L.	Rutaceae	mon	MF	Raute	syst	FEV	7	heilt das Fieber, wenn du sie grün in Öl abkochst
Ruta graveolens L.	Rutaceae	mon	MF	Raute	syst	GAS	7	Grimmdarm, nützt sämtlichen Darmkrankheiten
Ruta graveolens L.	Rutaceae	mon	MF	Raute	syst	GAS	7	helt Schmerzen von Flanke, Leber, wirkt verdünnend auf die Galle, erweichend auf den harten Leib, stärkt Magen
Ruta graveolens L.	Rutaceae	mon	MF	Raute	syst	GAS	7	nutzbringend dem Magen
Ruta graveolens L.	Rutaceae	mon	MF	Raute	syst	GAS	7	stillt Bauchgrimmen
Ruta graveolens L.	Rutaceae	mon	MF	Raute	syst	GAS	7	treibt sie die Spulwürmer hinaus
Ruta graveolens L.	Rutaceae	mon	MF	Raute	syst	GYN	7	hilft sie der Gebärmutter und einer Eingeweidegeschwulst
Ruta graveolens L.	Rutaceae	mon	MF	Raute	syst	GYN	7	beseitigt sie Geschwülste in der Gebärmutter
Ruta graveolens L.	Rutaceae	mon	MF	Raute	syst	GYN	7	beschelunigt die Kindsgeburt, sorgt für geordneten Monatsfluss
Ruta graveolens L.	Rutaceae	mon	MF	Raute	syst	GYN	7	hilft sie der Gebärmutter und einer Eingeweidegeschwulst
Ruta graveolens L.	Rutaceae	mon	MF	Raute	syst	NER	7	Kopfschmerzen
Ruta graveolens L.	Rutaceae	mon	MF	Raute	syst	OTH	7	Antoniusfeuer,
Ruta graveolens L.	Rutaceae	mon	MF	Raute	syst	RES	7	heilt Schmerzen von Brust
Ruta graveolens L.	Rutaceae	mon	MF	Raute	syst	RES	7	stillt Husten
Ruta graveolens L.	Rutaceae	mon	MF	Raute	syst	RES	7	erfreut Lunge und Brust und heilt das Rippenleiden
Ruta graveolens L.	Rutaceae	mon	MF	Raute	syst	SKE	7	Gichtkrampf und Hüftgicht
Ruta graveolens L.	Rutaceae	mon	MF	Raute	syst	URO	7	Wassersüchtigen
Ruta graveolens L.	Rutaceae	mon	MF	Raute	syst	URO	7	heilt Schmerzen Nieren
Ruta graveolens L.	Rutaceae	mon	MF	Raute	top	URO	7	geschwollene Hoden
Ruta graveolens L.	Rutaceae	ren	LF	Weinraute	syst	ANT	CCCL / CCXXXVI	widerstreben allerley gifft und dem pestilentzischen boesen lufft, wider die schlangen und natern
Ruta graveolens L.	Rutaceae	ren	LF	Weinraute	syst	ANT	CCCL / CCXXXVI	gut wider die schaedlichen pfifferling
Ruta graveolens L.	Rutaceae	ren	LF	Weinraute	syst	ANT	CCCL / CCXXXVI	nützlich denen so von den scorpion, spinnen, bynen, wesüen, hurneuss unnd wuetenden hunden gestochen oder gebissen seind
Ruta graveolens L.	Rutaceae	ren	LF	Weinraute	top	ANT	CCCL / CCXXXVI	nützlich denen so von den scorpion, spinnen, bynen, wesüen, hurneuss unnd wuetenden hunden gestochen oder gebissen seind
Ruta graveolens L.	Rutaceae	ren	LF	Weinraute	syst	ANT	CCCL / CCXXXVI	gut für allerley toedtlich gifft
Ruta graveolens L.	Rutaceae	ren	LF	Weinraute	syst	APH	CCCL / CCXXXVI	tilget aus den menschlichen Samen
Ruta graveolens L.	Rutaceae	ren	LF	Weinraute	syst	CAR	CCCL / CCXXXVI	vertreibt es die geschwulst der Wassersucht
Ruta graveolens L.	Rutaceae	ren	LF	Weinraute	top	CAR	CCCL / CCXXXVI	vertreibt es die geschwulst der Wassersucht
Ruta graveolens L.	Rutaceae	ren	LF	Weinraute	top	DER	CCCL / CCXXXVI	allerley flechten und zittermaeler am leib
Ruta graveolens L.	Rutaceae	ren	LF	Weinraute	top	DER	CCCL / CCXXXVI	heylen sie allerley maeler derselbigen [haut]
Ruta graveolens L.	Rutaceae	ren	LF	Weinraute	top	DER	CCCL / CCXXXVI	das rotlauff und den fliessenden grind des hauptes
Ruta graveolens L.	Rutaceae	ren	LF	Weinraute	top	DER	CCCL / CCXXXVI	stellen das bluten der nasen
Ruta graveolens L.	Rutaceae	ren	LF	Weinraute	top	DER	CCCL / CCXXXVI	so erwermen sie der erfrorenen glider
Ruta graveolens L.	Rutaceae	ren	LF	Weinraute	top	DER	CCCL / CCXXXVI	heylen allerley rauden grund und kropff
Ruta graveolens L.	Rutaceae	ren	LF	Weinraute	top	DER	CCCL / CCXXXVI	zerteylen sie die geschwulst [der gemecht] und lindern den schmerzen derselben
Ruta graveolens L.	Rutaceae	ren	LF	Weinraute	top	DER	CCCL / CCXXXVI	verzert das undergerunnen blut und allerley masen am leib
Ruta graveolens L.	Rutaceae	ren	LF	Weinraute	top	EAR	CCCL / CCXXXVI	weetagen der ohren, bring das gehoer widerumb unnd vertreibet das sausen und klingen derselbigen
Ruta graveolens L.	Rutaceae	ren	LF	Weinraute	syst	EYE	CCCL / CCXXXVI	scheffen das gesicht

Ruta graveolens L.	Rutaceae	ren	LF	Weinraute	top	EYE	CCCL / CCXXXVI	legen sie den weetagen der augen, macht ein klar und lauter gesicht
Ruta graveolens L.	Rutaceae	ren	LF	Weinraute	top	EYE	CCCL / CCXXXVI	macht er klare augen
Ruta graveolens L.	Rutaceae	ren	LF	Weinraute	syst	GAS	CCCL / CCXXXVI	stellen den bauchfluss
Ruta graveolens L.	Rutaceae	ren	LF	Weinraute	syst	GAS	CCCL / CCXXXVI	miltert das grimmen
Ruta graveolens L.	Rutaceae	ren	LF	Weinraute	top	GAS	CCCL / CCXXXVI	grimmen und weetagen der daerm
Ruta graveolens L.	Rutaceae	ren	LF	Weinraute	top	GAS	CCCL / CCXXXVI	miltert es die schmerzen der gleychen [würm]
Ruta graveolens L.	Rutaceae	ren	LF	Weinraute	syst	GAS	CCCL / CCXXXVI	fuert aus allerely würm
Ruta graveolens L.	Rutaceae	ren	LF	Weinraute	syst	GYN	CCCL / CCXXXVI	reyniget er die frawen nach der geburt und treibt aus das bürdlin und die todten frucht
Ruta graveolens L.	Rutaceae	ren	LF	Weinraute	top	GYN	CCCL / CCXXXVI	verhuetet das auffsteigender muter
Ruta graveolens L.	Rutaceae	ren	LF	Weinraute	syst	n.d.	CCCL / CCXXXVI	weetagen der seiten, brust und dergleichen
Ruta graveolens L.	Rutaceae	ren	LF	Weinraute	top	NER	CCCL / CCXXXVI	weetagen des haupts
Ruta graveolens L.	Rutaceae	ren	LF	Weinraute	vol	NER	CCCL / CCXXXVI	so mit dem tieffen schlaaf beladen seind fürgehalten, das sie daran reiechen mact dieselbigen widerum munder und wacker
Ruta graveolens L.	Rutaceae	ren	LF	Weinraute	syst	RES	CCCL / CCXXXVI	schwerlich athmen und husten zu den geschwaeren der lungen
Ruta graveolens L.	Rutaceae	ren	LF	Weinraute	syst	SKE	CCCL / CCXXXVI	weetagen der hüfft
Ruta graveolens L.	Rutaceae	ren	LF	Weinraute	top	SKE	CCCL / CCXXXVI	heylet die zerbrochnen glider
Ruta graveolens L.	Rutaceae	ren	LF	Weinraute	top	URO	CCCL / CCXXXVI	so einer nit harnen mag
Ruta graveolens L.	Rutaceae	ren	LF	Weinraute	n.d.	URO	CCCL / CCXXXVI	treibt den harn du bring den frawen ihre krankheyt
Ruta graveolens L.	Rutaceae	s pop	JK	Weinraute	syst	ANT	p. 439	Bisse von Schlangen, wütenden Hunden, Insektenstichen
Ruta graveolens L.	Rutaceae	s pop	JK	Weinraute	top	ANT	p. 439	Bisse von Schlangen, wütenden Hunden, Insektenstichen
Ruta graveolens L.	Rutaceae	s pop	JK	Weinraute	syst	CAR	p. 439	Schwindel, Herzbeklemmung
Ruta graveolens L.	Rutaceae	s pop	JK	Weinraute	top	DER	p. 439	Warzen
Ruta graveolens L.	Rutaceae	s pop	JK	Weinraute	top	EAR	p. 439	Ohrensausen, das nicht von Herzschwäche herrührt; Ohrenkanäle, die verschwollen sind
Ruta graveolens L.	Rutaceae	s pop	JK	Weinraute	vol	EAR	p. 439	Schwerhörigkeit
Ruta graveolens L.	Rutaceae	s pop	JK	Weinraute	top	EYE	p. 439	erhält uns stärkt die Sehkraft
Ruta graveolens L.	Rutaceae	s pop	JK	Weinraute	syst	GAS	p. 439	alle Arten von Würmern
Ruta graveolens L.	Rutaceae	s pop	JK	Weinraute	syst	NER	p. 439	Fallsucht
Ruta graveolens L.	Rutaceae	s pop	JK	Weinraute	vol	OTH	p. 439	Fliegen, Wanzen, usw. Kleidermotten
Ruta graveolens L.	Rutaceae	s pop	KN	Weinraute	syst	CAR	p. 481	Kongestion, das ist Blutandrang zum Kopfe, bei Eingenommenheit des Kopfes, Schwindel
Ruta graveolens L.	Rutaceae	s pop	KN	Weinraute	syst	CAR	p. 481	Kongestion, das ist Blutandrang zum Kopfe, bei Eingenommenheit des Kopfes, Schwindel
Ruta graveolens L.	Rutaceae	s pop	KN	Weinraute	syst	GAS	p. 481	Verdaunungsstörungen, Blähsucht und Koliken
Ruta graveolens L.	Rutaceae	s pop	KN	Weinraute	syst	GAS	p. 481	Verdaunungsstörungen, Blähsucht und Koliken
Ruta graveolens L.	Rutaceae	s pop	KN	Weinraute	syst	GAS	p. 481	Verdaunungsstörungen, Blähsucht und Koliken
Ruta graveolens L.	Rutaceae	s pop	KN	Weinraute	syst	GYN	p. 481	Unterleibsbeschwerden und -zuständen
Ruta graveolens L.	Rutaceae	s pop	KN	Weinraute	syst	GYN	p. 481	Unterleibsbeschwerden und -zuständen
Ruta graveolens L.	Rutaceae	s pop	KN	Weinraute	syst	GYN	p. 481	Unterleibsbeschwerden und -zuständen
Ruta graveolens L.	Rutaceae	s pop	KN	Weinraute	syst	RES	p. 481	Atmungsbeschwerden
Ruta graveolens L.	Rutaceae	s pop	KN	Weinraute	syst	RES	p. 481	Atmungsbeschwerden
Ruta graveolens L.	Rutaceae	s pop	KN	Weinraute	syst	RES	p. 481	Atmungsbeschwerden
Ruta graveolens L.	Rutaceae	s pop	UB	Weinraute	syst	CAR	p. 410	venöse Gefässerkrankungen
Salix alba aggr.	Salicaceae	ant	DIOS	Weide	vol	DER	103	sie vertreibt aber auch Kleingrind
Salix alba aggr.	Salicaceae	ant	DIOS	Weide	top	DER	103	Hautverhärtungen und Schwielen
Salix alba aggr.	Salicaceae	ant	DIOS	Weide	top	EAR	103	heilt mit Rosenöl zusammen Ohrenleiden
Salix alba aggr.	Salicaceae	ant	DIOS	Weide	n.d.	EYE	103	vertreiben, was die Pupille verdunkelt
Salix alba aggr.	Salicaceae	ant	DIOS	Weide	syst	GAS	103	hilft bei Blutspeien
Salix alba aggr.	Salicaceae	ant	DIOS	Weide	syst	GAS	103	hilft bei Blutspeien
Salix alba aggr.	Salicaceae	ant	DIOS	Weide	syst	GAS	103	sind bei Darmverschlingung angebracht
Salix alba aggr.	Salicaceae	ant	DIOS	Weide	syst	GYN	103	mit Wasser genommen verhindern sie die Empfängnis
Salix alba aggr.	Salicaceae	ant	DIOS	Weide	vol	SKE	103	Podagra
Salix alba aggr.	Salicaceae	mon	HvB	Weide	syst	OTH	3-36	aber sie taugt zu keinem Heilmittel
Salix alba aggr.	Salicaceae	ren	LF	Silberweide	syst	APH	XVIII - CXC / CXXVI	vertreiben den lust un neygung zur unkeüscheyt
Salix alba aggr.	Salicaceae	ren	LF	Silberweide	top	DER	XVIII - CXC / CXXVI	wartzen und hueneraugen
Salix alba aggr.	Salicaceae	ren	LF	Silberweide	top	EAR	osenoel, granatapfel	weetagen der oren
Salix alba aggr.	Salicaceae	ren	LF	Silberweide	top	EYE	XVIII - CXC / CXXVI	macht lautere augen und schoen angesicht
Salix alba aggr.	Salicaceae	ren	LF	Silberweide	n.d.	GAS	XVIII - CXC / CXXVI	denen so blut speien
Salix alba aggr.	Salicaceae	ren	LF	Silberweide	syst	GAS	XVIII - CXC / CXXVI	weetagen der zarten und subtieln daerm leiden
Salix alba aggr.	Salicaceae	ren	LF	Silberweide	n.d.	GAS	XVIII - CXC / CXXVI	denen so blut speien
Salix alba aggr.	Salicaceae	ren	LF	Silberweide	vol	SKE	XVIII - CXC / CXXVI	podagram
Salix alba aggr.	Salicaceae	s pop	BVA	Silberweide	top	DER	p. 197	Fussschweiss, Schuppenflechte, Kopfschuppen
Salix alba aggr.	Salicaceae	s pop	BVA	Silberweide	syst	FEV	p. 197	Fieber,Grippe
Salix alba aggr.	Salicaceae	s pop	BVA	Silberweide	syst	GYN	p. 197	Menstruationsbeschwerden
Salix alba aggr.	Salicaceae	s pop	BVA	Silberweide	syst	NER	p. 197	Kopfweh, Ischias
Salix alba aggr.	Salicaceae	s pop	BVA	Silberweide	syst	NER	p. 197	Nervenschwäche, Schlaflosigkeit, Beruhigung
Salix alba aggr.	Salicaceae	s pop	BVA	Silberweide	syst	RES	p. 197	Erkältung, Katarrh, Mandelschwellung
Salix alba aggr.	Salicaceae	s pop	BVA	Silberweide	syst	SKE	p. 197	Rheuma, Gicht, Arthritis, vermehrte Harnsäureausscheidung
Salix alba aggr.	Salicaceae	s pop	JK	Weiden	top	DER	p. 437	Schuppen vertreibendes Kopfwaschmittel
Salix alba aggr.	Salicaceae	s pop	JK	Weiden	top	DER	p. 437	stillen das Blut und fördern rasche und saubere Ausheilen der Wunde
Salix alba aggr.	Salicaceae	s pop	UB	Weide	syst	FEV	p. 201	Fiebersenkend
Salix alba aggr.	Salicaceae	s pop	UB	Weide	syst	SKE	p. 279	Arthrose
Salix alba aggr.	Salicaceae	sci	EMA	Weide	syst	FEV	HMPC/295338/2007	Traditional herbal medicinal product used for the relief of: fever associated with common cold
Salix alba aggr.	Salicaceae	sci	EMA	Weide	syst	FEV	HMPC/295338/2007	Traditional herbal medicinal product used for the relief of: fever associated with common cold
Salix alba aggr.	Salicaceae	sci	EMA	Weide	syst	FEV	HMPC/295338/2007	Traditional herbal medicinal product used for the relief of: fever associated with common cold
Salix alba aggr.	Salicaceae	sci	EMA	Weide	syst	NER	HMPC/295338/2007	Traditional herbal medicinal product used for the relief of: headache
Salix alba aggr.	Salicaceae	sci	EMA	Weide	syst	NER	HMPC/295338/2007	Traditional herbal medicinal product used for the relief of: headache
Salix alba aggr.	Salicaceae	sci	EMA	Weide	syst	NER	HMPC/295338/2007	Traditional herbal medicinal product used for the relief of: headache
Salix alba aggr.	Salicaceae	sci	EMA	Weide	syst	NER	HMPC/295338/2007	Traditional herbal medicinal product used for the relief of: headache
Salix alba aggr.	Salicaceae	sci	EMA	Weide	syst	SKE	HMPC/295338/2007	Traditional herbal medicinal product used for the relief of: minor articular pain
Salix alba aggr.	Salicaceae	sci	EMA	Weide	syst	SKE	HMPC/295338/2007	Traditional herbal medicinal product used for the relief of: minor articular pain
Salix alba aggr.	Salicaceae	sci	EMA	Weide	syst	SKE	HMPC/295338/2007	Traditional herbal medicinal product used for the relief of: minor articular pain
Salix alba aggr.	Salicaceae	sci	EMA	Weide	syst	SKE	HMPC/295338/2007	Traditional herbal medicinal product used for the relief of: minor articular pain
Salix alba aggr.	Salicaceae	sci	WI	Weide	syst	FEV	p. 518	leichte fieberhafte Infekte, Erkältungs- und Infektionskrankheiten
Salvia officinalis aggr.	Lamiaceae	ant	DIOS	Salbei	syst	ANT	177	Wunden des Stechrochen zu heilen
Salvia officinalis aggr.	Lamiaceae	ant	DIOS	Salbei	top	DER	177	ist ein Wund- und blutstillendes Mittel und reinigt böse Geschwüre
Salvia officinalis aggr.	Lamiaceae	ant	DIOS	Salbei	top	DER	177	Es färbt auch das Haar schwarz
Salvia officinalis aggr.	Lamiaceae	ant	DIOS	Salbei	vol	DER	177	Abkochung der Blätter und Zweige als Bähmittel beruhigt das Jucken an den Geschlechtsteilen
Salvia officinalis aggr.	Lamiaceae	ant	DIOS	Salbei	syst	GYN	177	die Katamenien und den Embryo hervorzuziehen
Salvia officinalis aggr.	Lamiaceae	ant	DIOS	Salbei	syst	URO	177	Abkochung der Blätter und Zweige als Trank hat die Kraft, den Urin zu treiben
Salvia officinalis aggr.	Lamiaceae	mon	HvB	Salbei	syst	GAS	1-63	oder wenn jemand stinkenden Atem hat
Salvia officinalis aggr.	Lamiaceae	mon	HvB	Salbei	syst	GAS	1-63	Widerwillen gegen das Essen
Salvia officinalis aggr.	Lamiaceae	mon	HvB	Salbei	syst	HUM	1-63	es vermindert den Überfluss der schlechten Säfte
Salvia officinalis aggr.	Lamiaceae	mon	HvB	Salbei	vol	OTH	1-63	und wer von irgendeiner schmutzigen Sache Gestank erleidet
Salvia officinalis aggr.	Lamiaceae	mon	HvB	Salbei	syst	SKE	1-63	etwas an Gicht leidet, Lähmung
Salvia officinalis aggr.	Lamiaceae	mon	MF	Salbei	syst	ANT	24	heilt er giftige Bisse
Salvia officinalis aggr.	Lamiaceae	mon	MF	Salbei	syst	DER	24	Blutfluss hemmen
Salvia officinalis aggr.	Lamiaceae	mon	MF	Salbei	top	DER	24	Jucken der weiblichen Scham und des männlichen Gliedes

Salvia officinalis aggr.	Lamiaceae	mon	MF	Salbei	syst	GAS	24	nützt den Bechwerden der Leber
Salvia officinalis aggr.	Lamiaceae	mon	MF	Salbei	syst	GYN	24	treibt die Leibesfrucht ab, sorgt für geordneten Monatsfluss
Salvia officinalis aggr.	Lamiaceae	mon	MF	Salbei	syst	RES	24	bezähmt er altverstockten Husten und Rippenfellschmerz
Salvia officinalis aggr.	Lamiaceae	mon	MF	Salbei	syst	URO	24	erregt den Harn
Salvia officinalis aggr.	Lamiaceae	ren	LF	Salbei	top	ANT	'II - CXXXVIII / XCII	heylet die biss der gifftigen thiern
Salvia officinalis aggr.	Lamiaceae	ren	LF	Salbei	top	DER	'II - CXXXVIII / XCII	gut zu allerley wunden, seubert dieselbe und heylet sie
Salvia officinalis aggr.	Lamiaceae	ren	LF	Salbei	top	DER	'II - CXXXVIII / XCII	stellet das blut so aus den wunden laufft
Salvia officinalis aggr.	Lamiaceae	ren	LF	Salbei	top	DER	'II - CXXXVIII / XCII	vertreiben das iucken an den gemechten
Salvia officinalis aggr.	Lamiaceae	ren	LF	Salbei	top	EAR	'II - CXXXVIII / XCII	zeucht die wuerm aus den ohren
Salvia officinalis aggr.	Lamiaceae	ren	LF	Salbei	syst	GAS	'II - CXXXVIII / XCII	gut der verstopffung der leber
Salvia officinalis aggr.	Lamiaceae	ren	LF	Salbei	syst	GAS	'II - CXXXVIII / XCII	rote rhur
Salvia officinalis aggr.	Lamiaceae	ren	LF	Salbei	syst	GYN	'II - CXXXVIII / XCII	bringen den frawen ihre zeit unnd treiben aus die todten frucht
Salvia officinalis aggr.	Lamiaceae	ren	LF	Salbei	top	OTH	'II - CXXXVIII / XCII	macht schwarz har
Salvia officinalis aggr.	Lamiaceae	ren	LF	Salbei	syst	RES	'II - CXXXVIII / XCII	vertreiben den husten un die weetagen der seiten
Salvia officinalis aggr.	Lamiaceae	ren	LF	Salbei	syst	URO	'II - CXXXVIII / XCII	treiben den harn
Salvia officinalis aggr.	Lamiaceae	s pop	JK	Salbei	top	DER	p. 409	Sänger, Kinder mit empfindlichem Hals
Salvia officinalis aggr.	Lamiaceae	s pop	JK	Salbei	top	DER	p. 409	Hauterkrankungen, Haare fein und glänzend
Salvia officinalis aggr.	Lamiaceae	s pop	JK	Salbei	top	DER	p. 409	Geschwüren, Ohrengeschwüre
Salvia officinalis aggr.	Lamiaceae	s pop	JK	Salbei	syst	FEV	p. 409	vertreibt Nachtschweiss, Lungenkranke, junge Leute in den Entwicklungsjahren und Frauen in den Wechseljahren
Salvia officinalis aggr.	Lamiaceae	s pop	JK	Salbei	syst	GAS	p. 409	Magen, fördert den Appetit
Salvia officinalis aggr.	Lamiaceae	s pop	JK	Salbei	syst	GAS	p. 409	Durchfall und Ruhr
Salvia officinalis aggr.	Lamiaceae	s pop	JK	Salbei	syst	GYN	p. 409	erwartenden Frauen
Salvia officinalis aggr.	Lamiaceae	s pop	JK	Salbei	top	GYN	p. 409	stärken den Unterleib
Salvia officinalis aggr.	Lamiaceae	s pop	JK	Salbei	syst	NER	p. 409	Gehirn
Salvia officinalis aggr.	Lamiaceae	s pop	JK	Salbei	syst	NER	p. 409	vom Schlag gerührten Patienten
Salvia officinalis aggr.	Lamiaceae	s pop	JK	Salbei	top	NER	p. 409	nehmen das Zittern
Salvia officinalis aggr.	Lamiaceae	s pop	JK	Salbei	top	OTH	p. 409	Kopfwaschungen tötet Ungezifer ab
Salvia officinalis aggr.	Lamiaceae	s pop	JK	Salbei	syst	RES	p. 409	säubert Lungen und heilt Schnupfen und Katarrh
Salvia officinalis aggr.	Lamiaceae	s pop	JK	Salbei	vol	RES	p. 409	engbrüstige und keuchende Männer
Salvia officinalis aggr.	Lamiaceae	s pop	JK	Salbei	syst	SKE	p. 409	Gicht und inneren Krämpfen
Salvia officinalis aggr.	Lamiaceae	s pop	JK	Salbei	top	SKE	p. 409	Kinderlähmung und deren Folgen
Salvia officinalis aggr.	Lamiaceae	s pop	JK	Salbei	top	TEE	p. 409	gute, frische Zähne, überdies auch den üblen Atem behebt
Salvia officinalis aggr.	Lamiaceae	s pop	JK	Salbei	syst	TON	p. 409	in Zeiten von ansteckenden Krankheiten
Salvia officinalis aggr.	Lamiaceae	s pop	JK	Salbei	syst	URO	p. 409	Nieren, Blase
Salvia officinalis aggr.	Lamiaceae	s pop	KN	Gartensalbei	top	DER	p. 483	entzündliche Veränderungen in Mund, Gaumen, Rachen, Zahnfleischblutungen und -eiterungen
Salvia officinalis aggr.	Lamiaceae	s pop	KN	Gartensalbei	top	DER	p. 483	entzündliche Veränderungen in Mund, Gaumen, Rachen, Zahnfleischblutungen und -eiterungen
Salvia officinalis aggr.	Lamiaceae	s pop	KN	Gartensalbei	syst	GAS	p. 483	akuten und katarrhalischen Veränderungen des Verdauungstraktes und ihrer Folgezustände
Salvia officinalis aggr.	Lamiaceae	s pop	KN	Gartensalbei	syst	GAS	p. 483	akuten und katarrhalischen Veränderungen des Verdauungstraktes und ihrer Folgezustände
Salvia officinalis aggr.	Lamiaceae	s pop	KN	Gartensalbei	syst	GYN	p. 483	bei verminderter Perionde
Salvia officinalis aggr.	Lamiaceae	s pop	KN	Gartensalbei	syst	GYN	p. 483	bei verminderter Perionde
Salvia officinalis aggr.	Lamiaceae	s pop	KN	Gartensalbei	syst	OTH	p. 483	Fettsucht
Salvia officinalis aggr.	Lamiaceae	s pop	KN	Gartensalbei	syst	OTH	p. 483	Fettsucht
Salvia officinalis aggr.	Lamiaceae	s pop	KN	Gartensalbei	syst	RES	p. 483	Lungenverschleimung und Lungentuberkulose mit Nachtschweiss
Salvia officinalis aggr.	Lamiaceae	s pop	KN	Gartensalbei	syst	RES	p. 483	Lungenverschleimung und Lungentuberkulose mit Nachtschweiss
Salvia officinalis aggr.	Lamiaceae	s pop	KN	Gartensalbei	syst	SKE	p. 483	Stoffwechselstörungen: Gicht, Rheumatismus
Salvia officinalis aggr.	Lamiaceae	s pop	KN	Gartensalbei	syst	SKE	p. 483	Stoffwechselstörungen: Gicht, Rheumatismus
Salvia officinalis aggr.	Lamiaceae	s pop	KN	Gartensalbei	syst	URO	p. 483	Nierenausscheidung wird vermehrt
Salvia officinalis aggr.	Lamiaceae	s pop	KN	Gartensalbei	syst	URO	p. 483	Nierenausscheidung wird vermehrt
Salvia officinalis aggr.	Lamiaceae	s pop	UB	Salbei	top	DER	p. 361	übermässiges Schwitzen
Salvia officinalis aggr.	Lamiaceae	s pop	UB	Salbei	syst	GAS	p. 157	Amara aromatica, Magenerkrankungen, Gallestörungen
Salvia officinalis aggr.	Lamiaceae	s pop	UB	Salbei	syst	GAS	p. 157	Amara aromatica, Magenerkrankungen, Gallestörungen
Salvia officinalis aggr.	Lamiaceae	s pop	UB	Salbei	syst	GAS	p. 157	Amara aromatica, Magenerkrankungen, Gallestörungen
Salvia officinalis aggr.	Lamiaceae	s pop	UB	Salbei	top	RES	p. 229	Schleimhautentzündungen im Mund-Rachen-Raum
Salvia officinalis aggr.	Lamiaceae	s pop	WI	Salbei	syst	GYN	p. 522	Volksmedizinisch: Abstillen, Menstruationsfördernd
Salvia officinalis aggr.	Lamiaceae	s pop	WI	Salbei	syst	OTH	p. 522	Volksmedizinisch: blutzuckersenkend
Salvia officinalis aggr.	Lamiaceae	sci	EMA	Salbei	syst	DER	HMPC/331653/2008	Traditional herbal medicinal product for relief of excessive sweating.
Salvia officinalis aggr.	Lamiaceae	sci	EMA	Salbei	top	DER	HMPC/331653/2008	Traditional herbal medicinal product for the symptomatic treatment of inflammations in the mouth or the throat
Salvia officinalis aggr.	Lamiaceae	sci	EMA	Salbei	top	DER	HMPC/331653/2008	Traditional herbal medicinal product for relief of minor skin inflammations.
Salvia officinalis aggr.	Lamiaceae	sci	EMA	Salbei	top	DER	HMPC/331653/2008	Traditional herbal medicinal product for the symptomatic treatment of inflammations in the mouth or the throat
Salvia officinalis aggr.	Lamiaceae	sci	EMA	Salbei	syst	GAS	HMPC/331653/2008	Traditional herbal medicinal product for symptomatic treatment of mild dyspeptic, complaints such as heartburn and bloating
Salvia officinalis aggr.	Lamiaceae	sci	EMA	Salbei	syst	GAS	HMPC/331653/2008	Traditional herbal medicinal product for symptomatic treatment of mild dyspeptic, complaints such as heartburn and bloating
Salvia officinalis aggr.	Lamiaceae	sci	EMA	Salbei	syst	GAS	HMPC/331653/2008	Traditional herbal medicinal product for symptomatic treatment of mild dyspeptic, complaints such as heartburn and bloating
Salvia officinalis aggr.	Lamiaceae	sci	ESCOP	Salviae officinalis foli	syst	DER	I p. 452	hyperhidrosis
Salvia officinalis aggr.	Lamiaceae	sci	ESCOP	Salviae officinalis foli	top	DER	I p. 452	inflammation and infections of the mouth and throat such as stomatitis, gingivitis and pharyngitis
Sambucus spp. (*S.ebulus*, *S. nigra*)	Adoxaceae	mon	HvB	Attich	top	DER	1-120	Und wenn ein Mensch an den Fingern und an den Füssen den Grind hat
Sambucus spp. (*S.ebulus*, *S. nigra*)	Adoxaceae	mon	HvB	Attich	top	NER	1 - 120	Aber wenn einem Menschen von üblen Säften der Kopf wie ein Sturzbach tost
Sambucus spp. (*S.ebulus*, *S. nigra*)	Adoxaceae	s pop	JK	Attich	syst	SKE	p. 327	Ischias, Rheumatismus, Gicht
Sambucus spp. (*S.ebulus*, *S. nigra*)	Adoxaceae	s pop	KN	Attich	syst	URO	p. 456	treibt mit ausserordentlicher Wirkung bei Wassersucht das Wasser ab und reinigt die Nieren
Sambucus spp. (*S.ebulus*, *S. nigra*)	Adoxaceae	s pop	KN	Attich	syst	URO	p. 456	Auch bei anderen Zufällen im Unterleib, die von schlechten Säften herrühren, wirkt er gut; er scheidet die Säfte durch den Urin aus
Sambucus spp. (*S.ebulus*, *S. nigra*)	Adoxaceae	ant	DIOS	Holunder	syst	ANT	258	Auch die Frucht, mit Wein getrunken, hat dieselbe Wirkung
Sambucus spp. (*S.ebulus*, *S. nigra*)	Adoxaceae	ant	DIOS	Holunder	syst	ANT	258	hilft sie auch bei Schlangenbiss
Sambucus spp. (*S.ebulus*, *S. nigra*)	Adoxaceae	ant	DIOS	Holunder	top	DER	258	hindern Entzündungen, ebenso sind sie als Kataplasma ein gutes Mittel bei Verbrennungen und Hundsbiss; Sie verbinden auch fistelartige Geschwüre
Sambucus spp. (*S.ebulus*, *S. nigra*)	Adoxaceae	ant	DIOS	Holunder	top	GYN	258	erweicht und öffnet sie die Gebärmutter und bringt Affectionen derselben in Ordnung
Sambucus spp. (*S.ebulus*, *S. nigra*)	Adoxaceae	ant	DIOS	Holunder	syst	HUM	258	Die Blätter, wie Gemüse gekocht, fahren Schleim und Galle ab
Sambucus spp. (*S.ebulus*, *S. nigra*)	Adoxaceae	ant	DIOS	Holunder	top	OTH	258	eingerieben färbt sie die Haare schwarz
Sambucus spp. (*S.ebulus*, *S. nigra*)	Adoxaceae	ant	DIOS	Holunder	top	SKE	258	helfen bei Podagra
Sambucus spp. (*S.ebulus*, *S. nigra*)	Adoxaceae	ant	DIOS	Holunder	syst	URO	258	Auch die Frucht, mit Wein getrunken, hat dieselbe Wirkung
Sambucus spp. (*S.ebulus*, *S. nigra*)	Adoxaceae	ant	DIOS	Holunder	syst	URO	258	ist für Wassersüchtige zuträglich
Sambucus spp. (*S.ebulus*, *S. nigra*)	Adoxaceae	mon	HvB	Holunder	syst	GAS	3-44	Gelbsucht
Sambucus spp. (*S.ebulus*, *S. nigra*)	Adoxaceae	mon	HvB	Holunder	vol	GAS	3-44	Gelbsucht
Sambucus spp. (*S.ebulus*, *S. nigra*)	Adoxaceae	mon	HvB	Holunder	top	GAS	3-44	Gelbsucht
Sambucus spp. (*S.ebulus*, *S. nigra*)	Adoxaceae	ren	LF	Schwarzer Holunde	syst	ANT	XXXVI - XXXVII / XX	nützlich, denen von natern gebissen seind
Sambucus spp. (*S.ebulus*, *S. nigra*)	Adoxaceae	ren	LF	Schwarzer Holunde	syst	CAR	XXXVI - XXXVII / XX	ist den wassersüchtigen seer gut, dan sie treibt gewaltig das wasser aus dem leib
Sambucus spp. (*S.ebulus*, *S. nigra*)	Adoxaceae	ren	LF	Schwarzer Holunde	top	DER	XXXVI - XXXVII / XX	gut zu dem brand un denen so von einem wuetenden hund gebissen seind, sie heylen auch die tieffen und holen wunden
Sambucus spp. (*S.ebulus*, *S. nigra*)	Adoxaceae	ren	LF	Schwarzer Holunde	syst	GYN	XXXVI - XXXVII / XX	erweycht sie und eroeffnet die hertten und verschwollne muter
Sambucus spp. (*S.ebulus*, *S. nigra*)	Adoxaceae	ren	LF	Schwarzer Holunde	top	GYN	XXXVI - XXXVII / XX	erweycht sie und eroeffnet die hertten und verschwollne muter
Sambucus spp. (*S.ebulus*, *S. nigra*)	Adoxaceae	ren	LF	Schwarzer Holunde	syst	HUM	XXXVI - XXXVII / XX	treiben aus den rotz pituitam genent und die gallen
Sambucus spp. (*S.ebulus*, *S. nigra*)	Adoxaceae	ren	LF	Schwarzer Holunde	top	OTH	XXXVI - XXXVII / XX	macht das har schwarz
Sambucus spp. (*S.ebulus*, *S. nigra*)	Adoxaceae	ren	LF	Schwarzer Holunde	n.d.	OTH	XXXVI - XXXVII / XX	vertreiben und toedten die floee und mucken
Sambucus spp. (*S.ebulus*, *S. nigra*)	Adoxaceae	ren	LF	Schwarzer Holunde	n.d.	OTH	XXXVI - XXXVII / XX	miltern die übrige hitz
Sambucus spp. (*S.ebulus*, *S. nigra*)	Adoxaceae	ren	LF	Schwarzer Holunde	top	SKE	XXXVI - XXXVII / XX	lindern den schmerzen des podagrams
Sambucus spp. (*S.ebulus*, *S. nigra*)	Adoxaceae	s pop	BVA	Holunder	syst	DER	p. 97	gegen üblen Körpergeruch, Hautunreinheiten
Sambucus spp. (*S.ebulus*, *S. nigra*)	Adoxaceae	s pop	BVA	Holunder	syst	FEV	p. 97	Fieber, Grippe, schweisstreibend

Sambucus spp. (*S.ebulus*, *S. nigra*)	Adoxaceae	s pop	BVA	Holunder	syst	FEV	p. 97	Infektionskrankheiten,
Sambucus spp. (*S.ebulus*, *S. nigra*)	Adoxaceae	s pop	BVA	Holunder	syst	HUM	p. 97	Blut-, Darmreinigung
Sambucus spp. (*S.ebulus*, *S. nigra*)	Adoxaceae	s pop	BVA	Holunder	syst	NER	p. 97	Neuralgien, Trigeminusneuralgie, Ischias Nervenentzündung
Sambucus spp. (*S.ebulus*, *S. nigra*)	Adoxaceae	s pop	BVA	Holunder	syst	RES	p. 97	Erkältung, Katarrh, Schnupfen, Lungenentzündung
Sambucus spp. (*S.ebulus*, *S. nigra*)	Adoxaceae	s pop	BVA	Holunder	syst	SKE	p. 97	Rheuma, Gicht
Sambucus spp. (*S.ebulus*, *S. nigra*)	Adoxaceae	s pop	BVA	Holunder	syst	TON	p. 97	Stärkung der körper eigenen Abwehrkräfte
Sambucus spp. (*S.ebulus*, *S. nigra*)	Adoxaceae	s pop	JK	Holunder	top	DER	p. 374	Wurm am Finger, Hundebiss
Sambucus spp. (*S.ebulus*, *S. nigra*)	Adoxaceae	s pop	JK	Holunder	top	EYE	p. 374	ziehen den Brand aus
Sambucus spp. (*S.ebulus*, *S. nigra*)	Adoxaceae	s pop	JK	Holunder	top	EYE	p. 374	Brand in den Augen
Sambucus spp. (*S.ebulus*, *S. nigra*)	Adoxaceae	s pop	JK	Holunder	syst	GAS	p. 374	räumt mit schlechten Magensäften gründlich auf
Sambucus spp. (*S.ebulus*, *S. nigra*)	Adoxaceae	s pop	JK	Holunder	syst	GAS	p. 374	Durchfall
Sambucus spp. (*S.ebulus*, *S. nigra*)	Adoxaceae	s pop	JK	Holunder	syst	RES	p. 374	Grippe und Erkältung
Sambucus spp. (*S.ebulus*, *S. nigra*)	Adoxaceae	s pop	JK	Holunder	syst	SKE	p. 374	Rheumatiker
Sambucus spp. (*S.ebulus*, *S. nigra*)	Adoxaceae	s pop	JK	Holunder	syst	URO	p. 374	reinigen das Blut und treiben zähe und verstockte Stoffe aus
Sambucus spp. (*S.ebulus*, *S. nigra*)	Adoxaceae	s pop	JK	Holunder	syst	URO	p. 374	Frühlingskur Säfte und Blut reinigen
Sambucus spp. (*S.ebulus*, *S. nigra*)	Adoxaceae	s pop	JK	Holunder	syst	URO	p. 374	Wassersüchtige und Korpulente, die wieder schlank werden möchten
Sambucus spp. (*S.ebulus*, *S. nigra*)	Adoxaceae	s pop	KN	Schwarzer Holunde	syst	FEV	p. 471	bei allen mit Fieber einhergehenden Krankheiten . . .
Sambucus spp. (*S.ebulus*, *S. nigra*)	Adoxaceae	s pop	KN	Schwarzer Holunde	syst	GAS	p. 472	reinigt den Magen
Sambucus spp. (*S.ebulus*, *S. nigra*)	Adoxaceae	s pop	KN	Schwarzer Holunde	syst	n.d.	p. 472	heftigem Abweichen
Sambucus spp. (*S.ebulus*, *S. nigra*)	Adoxaceae	s pop	KN	Schwarzer Holunde	syst	RES	p. 471	Erkrankungen der Luftwege, Schnupfen, Rachen- und Kehlkopf-, Luftröhrenentzündung . . .
Sambucus spp. (*S.ebulus*, *S. nigra*)	Adoxaceae	s pop	KN	Schwarzer Holunde	syst	URO	p. 472	Blutreinigungskur; wirkt auf die Urinausscheidung und günstig auf die Nieren
Sambucus spp. (*S.ebulus*, *S. nigra*)	Adoxaceae	s pop	KN	Schwarzer Holunde	syst	URO	p. 472	Wassersucht Einzug halten . . . treibt die Holunderwurzel so kräftig Wasser aus . . .
Sambucus spp. (*S.ebulus*, *S. nigra*)	Adoxaceae	s pop	UB	Schwarzer Holunde	syst	DER	p. 352	Gürtelrose
Sambucus spp. (*S.ebulus*, *S. nigra*)	Adoxaceae	s pop	UB	Schwarzer Holunde	syst	RES	p. 199	schweisstreibendes Mittel bei Erkältungskrankheiten
Sambucus spp. (*S.ebulus*, *S. nigra*)	Adoxaceae	s pop	UB	Schwarzer Holunde	syst	RES	p. 199	schweisstreibendes Mittel bei Erkältungskrankheiten
Sambucus spp. (*S.ebulus*, *S. nigra*)	Adoxaceae	s pop	UB	Schwarzer Holunde	top	RES	p. 199	schweisstreibendes Mittel bei Erkältungskrankheiten
Sambucus spp. (*S.ebulus*, *S. nigra*)	Adoxaceae	s pop	WI	Holunder	syst	NER	p. 529	Volksmedizin: Ischias und Neuralgien
Sambucus spp. (*S.ebulus*, *S. nigra*)	Adoxaceae	s pop	WI	Holunder	top	RES	p. 529	Volksmedizin: Gurgelmittel
Sambucus spp. (*S.ebulus*, *S. nigra*)	Adoxaceae	sci	EMA	Holunder	syst	RES	HMPC/283166/2007	Herbal medicinal product traditionally used for the relief of early symptoms of common cold.
Sambucus spp. (*S.ebulus*, *S. nigra*)	Adoxaceae	sci	EMA	Holunder	syst	RES	HMPC/283166/2007	Herbal medicinal product traditionally used for the relief of early symptoms of common cold.
Sambucus spp. (*S.ebulus*, *S. nigra*)	Adoxaceae	sci	WI	Holunder	syst	GAS	p. 529	Laxans
Sambucus spp. (*S.ebulus*, *S. nigra*)	Adoxaceae	sci	WI	Holunder	syst	RES	p. 529	Diaphoretikum bei Erkältungskrankheiten
Sambucus spp. (*S.ebulus*, *S. nigra*)	Adoxaceae	sci	WI	Holunder	syst	URO	p. 529	Diuretikum
Saponaria officinalis L.	Caryophyllace	ant	DIOS	Seifenkraut	top	DER	156	bessert es den Aussatz, zerteilt es die Geschwülste
Saponaria officinalis L.	Caryophyllace	ant	DIOS	Seifenkraut	top	EYE	156	Es wird den Kollyrien, welche das Gesicht schärfen und den Salben zugesetzt.
Saponaria officinalis L.	Caryophyllace	ant	DIOS	Seifenkraut	syst	GAS	156	Es hilft bei Leberleiden; Gelbsucht, Den Bauch regt es an
Saponaria officinalis L.	Caryophyllace	ant	DIOS	Seifenkraut	top	GYN	156	im Zäpfchen eingelegt, die Menstruation und tödtet sicher die Leibesfrucht
Saponaria officinalis L.	Caryophyllace	ant	DIOS	Seifenkraut	top	GYN	156	im Zäpfchen eingelegt, die Menstruation und tödtet sicher die Leibesfrucht
Saponaria officinalis L.	Caryophyllace	ant	DIOS	Seifenkraut	top	HUM	156	erregt ferner Niesen
Saponaria officinalis L.	Caryophyllace	ant	DIOS	Seifenkraut	syst	OTH	156	es erweicht auch die verhärtete Milz
Saponaria officinalis L.	Caryophyllace	ant	DIOS	Seifenkraut	syst	RES	156	Husten, Orthopnöe,
Saponaria officinalis L.	Caryophyllace	ant	DIOS	Seifenkraut	syst	URO	156	harntreibend.
Saponaria officinalis L.	Caryophyllace	ant	DIOS	Seifenkraut	syst	URO	156	zertrümmert es den Stein und lässt ihn mit dem Urin abgehen
Saponaria officinalis L.	Caryophyllace	mon	HvB	Seifenkraut	top	EAR	1-201	Ohrenklingeln
Saponaria officinalis L.	Caryophyllace	mon	HvB	Seifenkraut	top	EYE	1-201	Verdunkelung der Augen
Saponaria officinalis L.	Caryophyllace	mon	HvB	Seifenkraut	top	NER	1-201	im Körper schmerzen leide durch Geschwüre in der Eingeweide
Saponaria officinalis L.	Caryophyllace	mon	HvB	Seifenkraut	syst	RES	1-201	in der Brust dämpfig
Saponaria officinalis L.	Caryophyllace	ren	LF	Seifenkraut	top	DER	CCCCXLVI / CCCI	heylet sie die rauden unnd grind / verzeret sie geschwulst und beulen
Saponaria officinalis L.	Caryophyllace	ren	LF	Seifenkraut	n.d.	EYE	CCCCXLVI / CCCI	gebraucht zur Artzneyen damit man ein klar und lauter gesicht macht
Saponaria officinalis L.	Caryophyllace	ren	LF	Seifenkraut	syst	GAS	CCCCXLVI / CCCI	nützlich den lebersiechen
Saponaria officinalis L.	Caryophyllace	ren	LF	Seifenkraut	syst	GAS	CCCCXLVI / CCCI	machen einen linden bauch
Saponaria officinalis L.	Caryophyllace	ren	LF	Seifenkraut	syst	GAS	CCCCXLVI / CCCI	geelsucht
Saponaria officinalis L.	Caryophyllace	ren	LF	Seifenkraut	syst	GAS	CCCCXLVI / CCCI	lindert den bauch
Saponaria officinalis L.	Caryophyllace	ren	LF	Seifenkraut	syst	GYN	CCCCXLVI / CCCI	reinigt die muter
Saponaria officinalis L.	Caryophyllace	ren	LF	Seifenkraut	top	GYN	CCCCXLVI / CCCI	bring den frawen ihre zeit unnd treibt aus die todten frucht
Saponaria officinalis L.	Caryophyllace	ren	LF	Seifenkraut	top	HUM	CCCCXLVI / CCCI	in die nasen gethon macht sie niesen / aber mit honig vermegt unnd in die nasen gethon reyniget sie durch den mund
Saponaria officinalis L.	Caryophyllace	ren	LF	Seifenkraut	syst	OTH	CCCCXLVI / CCCI	macht die Milz kleiner
Saponaria officinalis L.	Caryophyllace	ren	LF	Seifenkraut	syst	RES	CCCCXLVI / CCCI	dem husten unnd denen so schwerlich athmen
Saponaria officinalis L.	Caryophyllace	ren	LF	Seifenkraut	syst	RES	CCCCXLVI / CCCI	zu den gebresten der brust
Saponaria officinalis L.	Caryophyllace	ren	LF	Seifenkraut	syst	RES	CCCCXLVI / CCCI	denen so einen schweren athemb haben
Saponaria officinalis L.	Caryophyllace	ren	LF	Seifenkraut	syst	URO	CCCCXLVI / CCCI	treibt den harn
Saponaria officinalis L.	Caryophyllace	ren	LF	Seifenkraut	syst	URO	CCCCXLVI / CCCI	treibt den harn
Saponaria officinalis L.	Caryophyllace	ren	LF	Seifenkraut	syst	URO	CCCCXLVI / CCCI	bricht sie den stein und treibt ihn durch den harn aus
Saponaria officinalis L.	Caryophyllace	s pop	UB	Seifenkraut	syst	RES	p. 222	Auswurfförderung
Saponaria officinalis L.	Caryophyllace	s pop	WI	Seifenkraut	syst	DER	p. 535	Volksmedizin: Hautkrankheiten
Saponaria officinalis L.	Caryophyllace	s pop	WI	Seifenkraut	syst	SKE	p. 535	Volksmedizin: rheumatischen Beschwerden
Saponaria officinalis L.	Caryophyllace	sci	WI	Seifenkraut	syst	RES	p. 535	Expektorans bei Bronchitis
Secale cereale L.	Poaceae	ant	DIOS	Roggen	syst	OTH	138	Zu derselben Getreideart wie der Dinkel gebart die Olyra, sie ist aber um etwas weniger nahrhaft als jener. Sie wird aber auch zur Brodbereitung verwandt, auch ein grobes Mehl wird davon gemacht
Secale cereale L.	Poaceae	mon	HvB	Roggen	top	DER	1-2	; und wenn Krebse nämlich dünne Würmlein, das Fleisch des Menschen zernagen
Secale cereale L.	Poaceae	mon	HvB	Roggen	syst	OTH	1-2	und für jene ist er gut, die fettes Fleisch haben, weil es ihr Fleisch mindert
Secale cereale L.	Poaceae	ren	LF	Roggen	syst	OTH	138	
Secale cereale L.	Poaceae	s pop	VOG	Roggen	syst	GAS	p. 297	Bei Magenstörungen . . .
Secale cereale L.	Poaceae	s pop	VOG	Roggen	syst	OTH	p. 89; 156	Kleinkindernahrung, Gesundheitspflege
Sinapis spp. (*Sinapis alba*, *Brassica nigra*	Brassicaceae	ant	DIOS	Weisser Senf	top	DER	153	Wenn sein Saft mit Honigmeth gemischt wird, so hilft er als Gurgelmittel bei geschwollenen Mandeln, gegen veraltete und verhärtete Rauheit der Luftröhre
Sinapis spp. (*Sinapis alba*, *Brassica nigra*	Brassicaceae	ant	DIOS	Weisser Senf	top	DER	153	heilt er die Fuchskrankheit; er reinigt das Gesicht und entfernt mit Honig, Fett oder Wachssalbe Sugillationen unter den Auge; Mit Essig wird er gegen Aussatz und wilde Flechten eingeschmiert
Sinapis spp. (*Sinapis alba*, *Brassica nigra*	Brassicaceae	ant	DIOS	Weisser Senf	top	DER	153	Mit Nutzen wird er den reizenden und Krätzesalben zugemischt
Sinapis spp. (*Sinapis alba*, *Brassica nigra*	Brassicaceae	ant	DIOS	Weisser Senf	top	EAR	153	Bei Schwerhörigkeit und Ohrensausen hilft er fein gestoßen mit Feigen in die Ohren gelegt
Sinapis spp. (*Sinapis alba*, *Brassica nigra*	Brassicaceae	ant	DIOS	Weisser Senf	top	EYE	153	Sein Saft, zusammen mit Honig eingestrichen, erweist sich heilsam gegen Stumpfsichtigkeit und schorfige Augenlider
Sinapis spp. (*Sinapis alba*, *Brassica nigra*	Brassicaceae	ant	DIOS	Weisser Senf	syst	FEV	153	Trocken wird er gegen die periodischen Fieber getrunken, indem er wie Graupen dem Getränk gestreut wird
Sinapis spp. (*Sinapis alba*, *Brassica nigra*	Brassicaceae	ant	DIOS	Weisser Senf	n.d.	GYN	153	und richtet die durch Mutterkrämpfe Gepeinigten auf
Sinapis spp. (*Sinapis alba*, *Brassica nigra*	Brassicaceae	ant	DIOS	Schwarzer Senf	n.d.	HUM	80	Es wirkt gegen chronische Leiden, indem es durch Öffnung der Poren die schlechten Säfte abführt
Sinapis spp. (*Sinapis alba*, *Brassica nigra*	Brassicaceae	ant	DIOS	Weisser Senf	other	HUM	153	Wird der Senf fein gestoßen in die Nase gebracht, so erregt er Niesen
Sinapis spp. (*Sinapis alba*, *Brassica nigra*	Brassicaceae	ant	DIOS	Weisser Senf	n.d.	NER	153	Er hilft bei Epilepsie
Sinapis spp. (*Sinapis alba*, *Brassica nigra*	Brassicaceae	ant	DIOS	Weisser Senf	top	NER	153	auch wird er denen, die an Schlafsucht leiden als Umschlag auf den geschorenen Kopf gelegt

Sinapis spp. (*Sinapis alba*, *Brassica nigra*	Brassicaceae	ant	DIOS	Weisser Senf	top	NER	153	Mit Feigen gemischt und bis zur Röte (der Haut) aufgelegt ist er ein gutes Mittel bei [Ischias- und Milzschmerzen] und überhaupt gegen jeden andauernden Schmerz, wo wir nach Art der einseitigen Leiden es aus der Tiefe an die Oberfläche ziehen wollen
Sinapis spp. (*Sinapis alba*, *Brassica nigra*	Brassicaceae	ant	DIOS	Weisser Senf	top	OTH	153	Mit Feigen gemischt und bis zur Röte (der Haut) aufgelegt ist er ein gutes Mittel bei [Ischias-] und Milzschmerzen [und überhaupt gegen jeden andauernden Schmerz, wo wir nach Art der einseitigen Leiden es aus der Tiefe an die Oberfläche ziehen wollen]
Sinapis spp. (*Sinapis alba*, *Brassica nigra*	Brassicaceae	ant	DIOS	Weisser Senf	top	SKE	153	Mit Feigen gemischt und bis zur Röte (der Haut) aufgelegt ist er ein gutes Mittel bei Ischias- und Milzschmerzen und überhaupt gegen jeden andauernden Schmerz, wo wir nach Art der einseitigen Leiden es aus der Tiefe an die Oberfläche ziehen wollen
Sinapis spp. (*Sinapis alba*, *Brassica nigra*	Brassicaceae	mon	HvB	Senf	syst	EYE	1-94	macht er die Augen klar
Sinapis spp. (*Sinapis alba*, *Brassica nigra*	Brassicaceae	mon	HvB	Senf	syst	OTH	1-94	so dass er eine gewisse Feuchtigkeit aus dem Kopf herauszieht
Sinapis spp. (*Sinapis alba*, *Brassica nigra*	Brassicaceae	ren	LF	Senf	n.d.	ANT	II - CCCIIII / CCIII	ist gut wider das gifft der pfifferlinge
Sinapis spp. (*Sinapis alba*, *Brassica nigra*	Brassicaceae	ren	LF	Senf	top	DER	II - CCCIIII / CCIII	verzert es das undergrunnen blut
Sinapis spp. (*Sinapis alba*, *Brassica nigra*	Brassicaceae	ren	LF	Senf	top	DER	II - CCCIIII / CCIII	heylet es die boese rauden und die geflecht
Sinapis spp. (*Sinapis alba*, *Brassica nigra*	Brassicaceae	ren	LF	Senf	top	DER	II - CCCIIII / CCIII	macht es die ausgefallene har wider wachsen, macht ein hübsch angesicht und vertreibt die maeler un flecken
Sinapis spp. (*Sinapis alba*, *Brassica nigra*	Brassicaceae	ren	LF	Senf	top	EAR	II - CCCIIII / CCIII	bringt es das gehoer wider und vertreibt das sausen in ohren
Sinapis spp. (*Sinapis alba*, *Brassica nigra*	Brassicaceae	ren	LF	Senf	top	EYE	II - CCCIIII / CCIII	macht klare augen und vertreibt die reuhe der augenbrawen
Sinapis spp. (*Sinapis alba*, *Brassica nigra*	Brassicaceae	ren	LF	Senf	syst	GAS	II - CCCIIII / CCIII	ist dem magen treflich gut
Sinapis spp. (*Sinapis alba*, *Brassica nigra*	Brassicaceae	ren	LF	Senf	syst	GAS	II - CCCIIII / CCIII	sterckt die dewung unnd zerteylet die groben speiss im magen / verzeret auch allerley überflüssige feüchtigkeyt des magens
Sinapis spp. (*Sinapis alba*, *Brassica nigra*	Brassicaceae	ren	LF	Senf	vol	GYN	II - CCCIIII / CCIII	wann man den frawen so due mutter über scih steigt den Senff mit essig vermengt fürhelt und daran riechen lasst so kommen sie wieder zu inen selbs
Sinapis spp. (*Sinapis alba*, *Brassica nigra*	Brassicaceae	ren	LF	Senf	syst	HUM	II - CCCIIII / CCIII	zeücht den zaehen schleim auss dem haupt und reynigt das hirn
Sinapis spp. (*Sinapis alba*, *Brassica nigra*	Brassicaceae	ren	LF	Senf	top	HUM	II - CCCIIII / CCIII	zeücht auss dem leib allerley feüchtigkeyt eüsserlich übergelegt
Sinapis spp. (*Sinapis alba*, *Brassica nigra*	Brassicaceae	ren	LF	Senf	top	HUM	II - CCCIIII / CCIII	senfffsamen gestossen in die nasen gethon macht niesen
Sinapis spp. (*Sinapis alba*, *Brassica nigra*	Brassicaceae	ren	LF	Senf	n.d.	NER	II - CCCIIII / CCIII	ist gut denen so die fallende sucht haben
Sinapis spp. (*Sinapis alba*, *Brassica nigra*	Brassicaceae	ren	LF	Senf	top	NER	II - CCCIIII / CCIII	ist gut denen so von dem schwaeren schlaff nit moegen erweckt werden
Sinapis spp. (*Sinapis alba*, *Brassica nigra*	Brassicaceae	ren	LF	Senf	top	OTH	II - CCCIIII / CCIII	ist gut denen so vonn den natern oder scorpion gestochen seind
Sinapis spp. (*Sinapis alba*, *Brassica nigra*	Brassicaceae	ren	LF	Senf	vol	OTH	II - CCCIIII / CCIII	veriagt er die natern unnd andere gifftige thier
Sinapis spp. (*Sinapis alba*, *Brassica nigra*	Brassicaceae	ren	LF	Senf	top	OTH	II - CCCIIII / CCIII	geschwollenem milz
Sinapis spp. (*Sinapis alba*, *Brassica nigra*	Brassicaceae	ren	LF	Senf	syst	RES	II - CCCIIII / CCIII	macht er leicht auswerffen
Sinapis spp. (*Sinapis alba*, *Brassica nigra*	Brassicaceae	ren	LF	Senf	top	RES	II - CCCIIII / CCIII	gegurgelt ist nützlich den geschwollnen mandeln und der rauhen kelen
Sinapis spp. (*Sinapis alba*, *Brassica nigra*	Brassicaceae	ren	LF	Senf	top	SKE	II - CCCIIII / CCIII	zu dem hüfftwee
Sinapis spp. (*Sinapis alba*, *Brassica nigra*	Brassicaceae	ren	LF	Senf	syst	URO	II - CCCIIII / CCIII	treibt den harn
Sinapis spp. (*Sinapis alba*, *Brassica nigra*	Brassicaceae	ren	LF	Senf	syst	URO	II - CCCIIII / CCIII	bring den frawen ir krankheyt
Sinapis spp. (*Sinapis alba*, *Brassica nigra*	Brassicaceae	ren	LF	Senf	syst	URO	II - CCCIIII / CCIII	treibt er den stein
Sinapis spp. (*Sinapis alba*, *Brassica nigra*	Brassicaceae	ren	LF	Senf	top	URO	II - CCCIIII / CCIII	den wassersüchtigen übergelegt vertreibt der die übrige feüchtigkeyt
Sinapis spp. (*Sinapis alba*, *Brassica nigra*	Brassicaceae	s pop	KN	Senf	top	DER	p. 414	hautreizend
Sinapis spp. (*Sinapis alba*, *Brassica nigra*	Brassicaceae	s pop	UB	Senf	top	CAR	p. 292	Notfallmittel bei Blutdruckkrisen
Sinapis spp. (*Sinapis alba*, *Brassica nigra*	Brassicaceae	s pop	UB	Senf	top	RES	p. 237	Husten, Atemwegserkrankungen
Sinapis spp. (*Sinapis alba*, *Brassica nigra*	Brassicaceae	s pop	UB	Senf	top	SKE	p. 292	degenerative rheumatische Erkrankungen
Sinapis spp. (*Sinapis alba*, *Brassica nigra*	Brassicaceae	s pop	WI	Schwarzer Senf	syst	GAS	p. 559	Volksmedizin: Appetitanregung
Sinapis spp. (*Sinapis alba*, *Brassica nigra*	Brassicaceae	s pop	WI	Schwarzer Senf	top	NER	p. 559	Volksmedizin: Pleuritiden, Neuritis
Sinapis spp. (*Sinapis alba*, *Brassica nigra*	Brassicaceae	s pop	WI	Schwarzer Senf	top	SKE	p. 559	Volksmedizin: rheumatischen Erkrankungen
Sinapis spp. (*Sinapis alba*, *Brassica nigra*	Brassicaceae	s pop	WI	Schwarzer Senf	top	URO	p. 559	Volksmedizin: Harnwegsinfekte
Sinapis spp. (*Sinapis alba*, *Brassica nigra*	Brassicaceae	sci	WI	Schwarzer Senf	top	OTH	p. 559	Segment-Therapie
Sinapis spp. (*Sinapis alba*, *Brassica nigra*	Brassicaceae	sci	WI	Schwarzer Senf	top	RES	p. 559	akute Bronchitis
Solanum spp. (*S. nigra*, *S. dulcamara*)	Solanaceae	ant	DIOS	Bittersüsser Nachts	top	EYE	231	Der Saft der Wurzel mit Honig als Salbe heilt Stumpfsichtigkeit
Solanum spp. (*S. nigra*, *S. dulcamara*)	Solanaceae	ant	DIOS	Bittersüsser Nachts	syst	NER	231	Seine Rinde wird den schmerzstillenden Mitteln und den Pastillen zugesetzt
Solanum spp. (*S. nigra*, *S. dulcamara*)	Solanaceae	ant	DIOS	Bittersüsser Nachts	syst	NER	231	hat schlafmachende Wirkung, aber milder als der Milchsaft des Mohns
Solanum spp. (*S. nigra*, *S. dulcamara*)	Solanaceae	ant	DIOS	Bittersüsser Nachts	top	TEE	231	im Munde behalten lindert sie Zahnschmerzen
Solanum spp. (*S. nigra*, *S. dulcamara*)	Solanaceae	ant	DIOS	Bittersüsser Nachts	syst	URO	231	Die Frucht ist stark harntreibend, den Wassersüchtigen werden etwa zwölf Fruchtbüschel gegeben, der Genuss von mehr bewirkt Geistesstörung
Solanum spp. (*S. nigra*, *S. dulcamara*)	Solanaceae	mon	HvB	Nachtschatten	top	CAR	1-121	Und wer Herzweh hat oder wer am Herzen schwach ist
Solanum spp. (*S. nigra*, *S. dulcamara*)	Solanaceae	mon	HvB	Nachtschatten	top	SKE	1-121	wer im Mark der Schenke leidet
Solanum spp. (*S. nigra*, *S. dulcamara*)	Solanaceae	mon	HvB	Nachtschatten	top	TEE	1-121	Zahnweh
Solanum spp. (*S. nigra*, *S. dulcamara*)	Solanaceae	mon	HvB	Nachtschatten	top	URO	1-121	wenn die Füsse anschwellen
Solanum spp. (*S. nigra*, *S. dulcamara*)	Solanaceae	ren	LF	Nachtschatten	top	DER	CCCXCII / CCLXV	Rotlauff
Solanum spp. (*S. nigra*, *S. dulcamara*)	Solanaceae	ren	LF	Nachtschatten	top	EAR	CCCXCII / CCLXV	vertreiben sie die ohrmützel
Solanum spp. (*S. nigra*, *S. dulcamara*)	Solanaceae	ren	LF	Nachtschatten	top	EYE	CCCXCII / CCLXV	so ein raesser fluss in die felt
Solanum spp. (*S. nigra*, *S. dulcamara*)	Solanaceae	ren	LF	Nachtschatten	top	GYN	CCCXCII / CCLXV	stellet der frawen krankheyt
Solanum spp. (*S. nigra*, *S. dulcamara*)	Solanaceae	ren	LF	Nachtschatten	top	NER	CCCXCII / CCLXV	Weetagen des Haupt
Solanum spp. (*S. nigra*, *S. dulcamara*)	Solanaceae	ren	LF	Nachtschatten	top	OTH	CCCXCII / CCLXV	und zu allen hitzigen gebresten
Solanum spp. (*S. nigra*, *S. dulcamara*)	Solanaceae	s pop	VAL	Nachtschatten	top	DER	p. 246	dermatose
Solanum spp. (*S. nigra*, *S. dulcamara*)	Solanaceae	s pop	VAL	Nachtschatten	top	DER	p. 370	affections curanées etc.
Solanum spp. (*S. nigra*, *S. dulcamara*)	Solanaceae	s pop	VAL	Nachtschatten	syst	DER	p. 246	dermatoses
Solanum spp. (*S. nigra*, *S. dulcamara*)	Solanaceae	s pop	VAL	Nachtschatten	top	DER	p. 246	dermatose
Solanum spp. (*S. nigra*, *S. dulcamara*)	Solanaceae	s pop	VAL	Nachtschatten	syst	GAS	p. 370	dyspepsies douloureuses, spasmes vésicaux
Solanum spp. (*S. nigra*, *S. dulcamara*)	Solanaceae	s pop	VAL	Nachtschatten	syst	RES	p. 370	coqueluche
Solanum spp. (*S. nigra*, *S. dulcamara*)	Solanaceae	s pop	VAL	Nachtschatten	syst	RES	p. 246	asthme, coqueluche, pneumonie
Solanum spp. (*S. nigra*, *S. dulcamara*)	Solanaceae	s pop	VAL	Nachtschatten	syst	SKE	p. 246	rhumatisme, goutte
Solanum spp. (*S. nigra*, *S. dulcamara*)	Solanaceae	sci	EMA	Bittersüsser Nachts	top	DER	IMPC/734361/2011	Traditional herbal medicinal product for the symptomatic relief of mild recurrent eczema
Sorbus domestica L.	Rosaceae	ant	DIOS	Speierling	syst	GAS	110	dass sie gegessen den Durchfall hemmen
Sorbus domestica L.	Rosaceae	ant	DIOS	Speierling	syst	GAS	110	leisten dasselbe [dass sie gegessen den Durchfall hemmen]
Sorbus domestica L.	Rosaceae	ant	DIOS	Speierling	syst	GAS	110	leisten dasselbe [dass sie gegessen den Durchfall hemmen]
Sorbus domestica L.	Rosaceae	mon	HvB	Speierling	n.d.	OTH	3 - 8	Aber seine Rinde, seine Blätter und sein Saft vermögen nicht viel zum Nutzen der Heilkunst
Sorbus domestica L.	Rosaceae	ren	LF	Speierling	syst	GAS	CCCXXVII/CCXVIII	stellen den Bauchfluss
Sorbus domestica L.	Rosaceae	s pop	VAL	Speierling	syst	GAS	p. 475	diarrhées, dysenterie
Symphytum officinale aggr.	Boraginaceae	ant	DIOS	Beinwell	top	DER	215	als Umschlag verkleben sie auch frische Wunden; Als Kataplasma dienen sie bei Entzündungen, besonders am After, und zwar vortheilhaft mit den Blättern der Kreuzwurz
Symphytum officinale aggr.	Boraginaceae	ant	DIOS	Beinwell	syst	GAS	215	welche an Blutspeien und inneren Abscessen leiden
Symphytum officinale aggr.	Boraginaceae	mon	HvB	Beinwell	syst	DER	1-145	Glied im Mensch preis gegeben und geschwürig und verwundet ist
Symphytum officinale aggr.	Boraginaceae	ren	LF	Beinwell, Wallwurz	top	DER	CCCXCVII / CCLXVI	trefflich zu allen wunden
Symphytum officinale aggr.	Boraginaceae	ren	LF	Beinwell, Wallwurz	top	DER	CCCXCVII / CCLXVI	lindert die hitz des afters
Symphytum officinale aggr.	Boraginaceae	ren	LF	Beinwell, Wallwurz	syst	GAS	CCCXCVII / CCLXVI	stellt die roten rhur
Symphytum officinale aggr.	Boraginaceae	ren	LF	Beinwell, Wallwurz	syst	GYN	CCCXCVII / CCLXVI	stellt der weiber kranckheyt
Symphytum officinale aggr.	Boraginaceae	ren	LF	Beinwell, Wallwurz	syst	GAS	CCCXCVII / CCLXVI	so blut ausspeien un inwendig gebrochen seind
Symphytum officinale aggr.	Boraginaceae	ren	LF	Beinwell, Wallwurz	syst	RES	CCCXCVII / CCLXVI	reynig auch die brust un lingen von dem eyter
Symphytum officinale aggr.	Boraginaceae	ren	LF	Beinwell, Wallwurz	top	SKE	CCCXCVII / CCLXVI	heylet die brüch, beinbrüche
Symphytum officinale aggr.	Boraginaceae	s pop	BVA	Wallwurz - Beinwell	top	DER	p. 243	Wundheilmittel, schlecht heilende Wunden, Narbenschmerzen, Schnitt- und Risswunden, Venenentzündungen,
Symphytum officinale aggr.	Boraginaceae	s pop	BVA	Wallwurz - Beinwell	top	DER	p. 243	Wundheilmittel, schlecht heilende Wunden, Narbenschmerzen, Schnitt- und Risswunden, Venenentzündungen,
Symphytum officinale aggr.	Boraginaceae	s pop	BVA	Wallwurz - Beinwell	top	EYE	p. 243	Prellungen des Augapfels
Symphytum officinale aggr.	Boraginaceae	s pop	BVA	Wallwurz - Beinwell	top	GAS	p. 243	Durchfall, Magen- Darmgeschwüre, Darmentzündungen
Symphytum officinale aggr.	Boraginaceae	s pop	BVA	Wallwurz - Beinwell	top	NER	p. 243	Ischias, Nervenschmerzen, Phatomschmerzen
Symphytum officinale aggr.	Boraginaceae	s pop	BVA	Wallwurz - Beinwell	top	NER	p. 243	Ischias, Nervenschmerzen, Phatomschmerzen
Symphytum officinale aggr.	Boraginaceae	s pop	BVA	Wallwurz - Beinwell	top	NER	p. 243	Ischias, Nervenschmerzen, Phatomschmerzen
Symphytum officinale aggr.	Boraginaceae	s pop	BVA	Wallwurz - Beinwell	top	RES	p. 243	Verschleimung der Atemwege
Symphytum officinale aggr.	Boraginaceae	s pop	BVA	Wallwurz - Beinwell	syst	SKE	p. 243	rheumatische Prozesse an Knochen, Gelenken, Muskeln

Symphytum officinale aggr.	Boraginaceae	s pop	BVA	Wallwurz - Beinwell	top	SKE	p. 243	Knochenerkrankungen, Knochenhautentzündun, Verstauchung, Quetschung, Sport- und Unfallverletzungen, Gelenkeserkrankungen, Kniegelenksbeschwerden, degenerativen Gelenksabnützugnen
Symphytum officinale aggr.	Boraginaceae	s pop	BVA	Wallwurz - Beinwell	top	SKE	p. 243	Knochenerkrankungen, Knochenhautentzündun, Verstauchung, Quetschung, Sport- und Unfallverletzungen, Gelenkeserkrankungen, Kniegelenksbeschwerden, degenerativen Gelenksabnützugnen
Symphytum officinale aggr.	Boraginaceae	s pop	JK	Beinwell	syst	DER	p. 332	Blutungen, auch innere Blutungen
Symphytum officinale aggr.	Boraginaceae	s pop	JK	Beinwell	top	DER	p. 332	Wunden, Schrunden
Symphytum officinale aggr.	Boraginaceae	s pop	JK	Beinwell	top	SKE	p. 332	Brüchen, Leistenbrüchen, Quetschungen, . . .
Symphytum officinale aggr.	Boraginaceae	s pop	MT	Beinwell, Wallwurz	top	DER	p. 12	äussere Wunden, Krampfadergeschwüre
Symphytum officinale aggr.	Boraginaceae	s pop	MT	Beinwell, Wallwurz	syst	GAS	p. 12	Beschwerden des Verdauungsapparates
Symphytum officinale aggr.	Boraginaceae	s pop	MT	Beinwell, Wallwurz	syst	RES	p. 12	Bronchialkatarrh
Symphytum officinale aggr.	Boraginaceae	s pop	MT	Beinwell, Wallwurz	top	SKE	p. 12	rheumatischen Muskelverdickungen, Nackenschmerzen, usw.
Symphytum officinale aggr.	Boraginaceae	s pop	MT	Beinwell, Wallwurz	top	SKE	p. 12	Quetschungen, Blutergüssen, Knochenbrüchen
Symphytum officinale aggr.	Boraginaceae	s pop	UB	Beinwell	top	SKE	p. 293	entzündliche rheumatische Erkrankungen
Symphytum officinale aggr.	Boraginaceae	s pop	UB	Beinwell	top	SKE	p. 321	Verstauchungen, Quetschungen, Prellungen
Symphytum officinale aggr.	Boraginaceae	s pop	UB	Beinwell	top	SKE	p. 321	Verstauchungen, Quetschungen, Prellungen
Symphytum officinale aggr.	Boraginaceae	s pop	UB	Beinwell	top	SKE	p. 321	Verstauchungen, Quetschungen, Prellungen
Symphytum officinale aggr.	Boraginaceae	s pop	WI	Beinwell	n.d.	GAS	p. 569	Volksheilkunde: Antidiarrhoikum
Symphytum officinale aggr.	Boraginaceae	s pop	WI	Beinwell	n.d.	RES	p. 569	Volksheilkunde: Bronchitis, Pleuritis
Symphytum officinale aggr.	Boraginaceae	sci	EMA	Beinwell	top	SKE	HMPC/572846/2009	Traditional herbal medicinal product used for the symptomatic treatment of minor sprains and bruises.
Symphytum officinale aggr.	Boraginaceae	sci	WI	Beinwell	top	DER	p. 569	Drüsenschwellungen, schlecht heilende Wunden
Symphytum officinale aggr.	Boraginaceae	sci	WI	Beinwell	top	DER	p. 569	Parodontose, Pharyngitis und Angina
Symphytum officinale aggr.	Boraginaceae	sci	WI	Beinwell	syst	GAS	p. 569	Gastritis, Magen- und Darmgeschwüren
Symphytum officinale aggr.	Boraginaceae	sci	WI	Beinwell	top	SKE	p. 569	Knochenhautreizungen, Gelenkentzündungen, Gichtknoten, zur Förderung der Kallusbildung
Teucrium spp.	Lamiaceae	ant	DIOS	Gamander	syst	ANT	196	Mit Wein getrunken und als Umschlag wirkt sie gegen den Biß giftiger Tiere
Teucrium spp.	Lamiaceae	ant	DIOS	Gamander	top	ANT	196	Mit Wein getrunken und als Umschlag wirkt sie gegen den Biß giftiger Tiere
Teucrium spp.	Lamiaceae	ant	DIOS	Gamander	syst	ANT	200	Wein wirkt es gegen den Biß der Schlangen und gegen tödliche Mittel
Teucrium spp.	Lamiaceae	ant	DIOS	Gamander	top	DER	196	mit Honig, um alte Wunden zu reinigen
Teucrium spp.	Lamiaceae	ant	DIOS	Gamander	top	DER	200	Weiter verklebt es Wunden, reinigt alte Geschwüre und bringt sie mit Honig zur Vernarbung, Trocken hält es Fleischwucherungen ein
Teucrium spp.	Lamiaceae	ant	DIOS	Gamander	top	EYE	196	Fein gestoßen mit Öl und eingestrichen entfernt sie Nebel auf den Augen
Teucrium spp.	Lamiaceae	ant	DIOS	Gamander	syst	GAS	196	die Kraft, bei Krämpfen, Husten
Teucrium spp.	Lamiaceae	ant	DIOS	Gamander	syst	GAS	200	gegen Magenstechen, Dysenterie
Teucrium spp.	Lamiaceae	ant	DIOS	Gamander	syst	GYN	196	Sie befördert auch die Menstruation und treibt den Embryo
Teucrium spp.	Lamiaceae	ant	DIOS	Gamander	top	GYN	200	befördert es die Menstruation
Teucrium spp.	Lamiaceae	ant	DIOS	Gamander	syst	OTH	196	erweicht die Milz, wenn sie mit Essig getrunken wird
Teucrium spp.	Lamiaceae	ant	DIOS	Gamander	syst	RES	196	Leberverhärtung
Teucrium spp.	Lamiaceae	ant	DIOS	Gamander	syst	RES	200	Auch reinigt es die Brust von eiterigem dickem Schleim, Ferner hat es gute Wirkung bei altem Husten
Teucrium spp.	Lamiaceae	ant	DIOS	Gamander	syst	RES	200	bei inneren Rupturen und Krämpfen, wenn es trocken mit Kresse, Honig und Harz zum Leckmittel gemischt wird
Teucrium spp.	Lamiaceae	ant	DIOS	Gamander	syst	RES	200	Krämpfen, wenn es trocken mit Kresse, Honig und Harz zum Leckmittel gemischt wird
Teucrium spp.	Lamiaceae	ant	DIOS	Gamander	top	SKE	200	bei Podagra leistet es gute Dienste, wenn es mit scharfem Essig eingerieben oder mit Wasser umgeschlagen wird
Teucrium spp.	Lamiaceae	ant	DIOS	Gamander	syst	URO	196	Harnverhaltung und beginnender Wassersucht zu helfen
Teucrium spp.	Lamiaceae	ant	DIOS	Gamander	syst	URO	200	harntreibende Kraft, wenn es frisch fein gestoßen getrunken wird; Harnverhaltung zu 2 Drachmen mit Honigwasser
Teucrium spp.	Lamiaceae	ant	DIOS	Gamander	top	URO	200	lindert es anhaltende Unterleibsentzündung
Teucrium spp.	Lamiaceae	mon	HvB	Gamander	top	DER	1 - 124	aber wer an der kleinen Krätze leidet, die zwischen Haut und dem Fleisch ist
Teucrium spp.	Lamiaceae	ren	LF	Gamander	syst	ANT	CXCVIII / CCCXXXV	widerstehen sie allerley gift
Teucrium spp.	Lamiaceae	ren	LF	Gamander	top	ANT	CXCVIII / CCCXXXV	widerstehen sie allerley gift, auch auswendig aufgelegt
Teucrium spp.	Lamiaceae	ren	LF	Gamander	top	DER	CXCVIII / CCCXXXV	reynigen sie alte Schaeden / mit oel vermischt . . . Heylen sie die brüch / wunden / unnd umb sich fressenden schaeden
Teucrium spp.	Lamiaceae	ren	LF	Gamander	top	EYE	CXCVIII / CCCXXXV	vertreibt die dunckelheyt der augen
Teucrium spp.	Lamiaceae	ren	LF	Gamander	syst	GYN	CXCVIII / CCCXXXV	bringen den frawen ihre zeit
Teucrium spp.	Lamiaceae	ren	LF	Gamander	syst	OTH	CXCVIII / CCCXXXV	machen das milz kleiner
Teucrium spp.	Lamiaceae	ren	LF	Gamander	syst	RES	CXCVIII / CCCXXXV	fürtrefflich denen so husten
Teucrium spp.	Lamiaceae	ren	LF	Gamander	syst	URO	CXCVIII / CCCXXXV	ein hart milz haben, nit wol harnen mögen und so newlich wassersüchtig worden
Teucrium spp.	Lamiaceae	s pop	FLU	Gamander	top	DER	p. 96	schlecht heilende Wunden
Teucrium spp.	Lamiaceae	s pop	FLU	Gamander	syst	GAS	p. 96	Magenbeschwerden und Appetitlosigkeit, Durchfälle
Teucrium spp.	Lamiaceae	s pop	JK	Gamander	syst	URO	361	treiben gewaltig den Urin
Thymus spp. (T. vulgaris, T. serpyllum)	Lamiaceae	ant	DIOS	Quendel	syst	ANT	179	auch bei Schlangenbissen
Thymus spp. (T. vulgaris, T. serpyllum)	Lamiaceae	ant	DIOS	Quendel	top	ANT	179	auch bei Schlangenbissen
Thymus spp. (T. vulgaris, T. serpyllum)	Lamiaceae	ant	DIOS	Thymian	top	DER	178	Mit Essig umgeschlagen verteilt er frische Ödeme, löst das geronnene Blut auf und vertreibt Feigwarzen und gestielte Warzen
Thymus spp. (T. vulgaris, T. serpyllum)	Lamiaceae	ant	DIOS	Thymian	syst	EYE	178	Stumpfsichtigkeit leiden
Thymus spp. (T. vulgaris, T. serpyllum)	Lamiaceae	ant	DIOS	Quendel	syst	GAS	179	hilft bei Leibschneiden, inneren Rupturen, Krämpfen und Leberanschwellungen
Thymus spp. (T. vulgaris, T. serpyllum)	Lamiaceae	ant	DIOS	Quendel	top	GAS	179	stillt er Blutbrechen
Thymus spp. (T. vulgaris, T. serpyllum)	Lamiaceae	ant	DIOS	Thymian	syst	GAS	178	treibt den Bandwurm
Thymus spp. (T. vulgaris, T. serpyllum)	Lamiaceae	ant	DIOS	Thymian	syst	GAS	178	hat er die Kraft, den Schleim durch den Bauch abzuführen
Thymus spp. (T. vulgaris, T. serpyllum)	Lamiaceae	ant	DIOS	Quendel	syst	GYN	179	denn getrunken befördert er die Katamenien
Thymus spp. (T. vulgaris, T. serpyllum)	Lamiaceae	ant	DIOS	Thymian	syst	GYN	178	den Embryo und die Nachgeburt aus und befördert die Menstruation
Thymus spp. (T. vulgaris, T. serpyllum)	Lamiaceae	ant	DIOS	Quendel	top	NER	179	Mit Essig gekocht lindert er unter Zusatz von Rosensalbe als Kompresse Kopfschmerzen; Besonders ist er ein gutes Mittel gehen Lethargie und Hirnwut
Thymus spp. (T. vulgaris, T. serpyllum)	Lamiaceae	ant	DIOS	Thymian	syst	RES	178	mit Honig hilft bei Orthopnöe und Asthm, Leckmittel ist er ein Expectoransa
Thymus spp. (T. vulgaris, T. serpyllum)	Lamiaceae	ant	DIOS	Thymian	top	SKE	178	als Aufschlag ein gutes Mittel bei Ischias
Thymus spp. (T. vulgaris, T. serpyllum)	Lamiaceae	ant	DIOS	Quendel	syst	URO	179	und treibt den Harn
Thymus spp. (T. vulgaris, T. serpyllum)	Lamiaceae	ant	DIOS	Thymian	syst	URO	178	Er ist auch ein harntreibendes Mittel
Thymus spp. (T. vulgaris, T. serpyllum)	Lamiaceae	mon	HvB	Quendel	syst	DER	1-32	Krätze
Thymus spp. (T. vulgaris, T. serpyllum)	Lamiaceae	mon	HvB	Quendel	top	DER	1-32	kleine Krätze / Grind
Thymus spp. (T. vulgaris, T. serpyllum)	Lamiaceae	mon	HvB	Thymian	top	DER	1-223	Lepra
Thymus spp. (T. vulgaris, T. serpyllum)	Lamiaceae	mon	HvB	Thymian	top	HUM	1-223	mindert die schlechten Säfte
Thymus spp. (T. vulgaris, T. serpyllum)	Lamiaceae	mon	HvB	Quendel	syst	NER	1-32	Gehrin krank und wie leer
Thymus spp. (T. vulgaris, T. serpyllum)	Lamiaceae	mon	HvB	Thymian	top	SKE	1-223	Lähmung und Stechen
Thymus spp. (T. vulgaris, T. serpyllum)	Lamiaceae	mon	MF	Quendel	syst	ANT	39	hilft er bei verderbenbringenden Bissen
Thymus spp. (T. vulgaris, T. serpyllum)	Lamiaceae	mon	MF	Quendel	top	ANT	39	hilft er bei verderbenbringenden Bissen
Thymus spp. (T. vulgaris, T. serpyllum)	Lamiaceae	mon	MF	Quendel	vol	APO	39	der Geruch des verbrannten Krautes treibt alle Schlangen in die Flucht
Thymus spp. (T. vulgaris, T. serpyllum)	Lamiaceae	mon	MF	Quendel	syst	GAS	39	stillt das Bauchgrimmen und hilft der Milz, Blutspeienden
Thymus spp. (T. vulgaris, T. serpyllum)	Lamiaceae	mon	MF	Quendel	syst	GAS	39	soll er den Leberschmerz beruhigen
Thymus spp. (T. vulgaris, T. serpyllum)	Lamiaceae	mon	MF	Quendel	syst	GYN	39	geordneten Monatsfluss bringen
Thymus spp. (T. vulgaris, T. serpyllum)	Lamiaceae	mon	MF	Quendel	top	NER	39	Kopfwehkranker
Thymus spp. (T. vulgaris, T. serpyllum)	Lamiaceae	mon	MF	Quendel	syst	URO	39	treibt er den Harn
Thymus spp. (T. vulgaris, T. serpyllum)	Lamiaceae	ren	LF	Feldthymian	syst	ANT	CXXXIX / XCIII	gut für das gift der würm und schlangen
Thymus spp. (T. vulgaris, T. serpyllum)	Lamiaceae	ren	LF	Feldthymian	top	ANT	CXXXIX / XCIII	gut für das gift der würm und schlangen
Thymus spp. (T. vulgaris, T. serpyllum)	Lamiaceae	ren	LF	Garten Thymian	syst	DER	CCLXXIIII / CCCXXI	gleiche würckung hat er so man ihn doert [wann die gemecht auffgeblasen und geschwollen seind]

Thymus spp. (T. vulgaris, T. serpyllum)	Lamiaceae	ren	LF	Garten Thymian	syst	DER	CCLXXIIII / CCCXXI	und zerteylt das gerunnen blut
Thymus spp. (T. vulgaris, T. serpyllum)	Lamiaceae	ren	LF	Garten Thymian	top	DER	CCLXXIIII / CCCXXI	wann die gemecht auffgeblasen und geschwollen seind
Thymus spp. (T. vulgaris, T. serpyllum)	Lamiaceae	ren	LF	Garten Thymian	top	DER	CCLXXIIII / CCCXXI	vertreibt die wartzen darüber gelegt
Thymus spp. (T. vulgaris, T. serpyllum)	Lamiaceae	ren	LF	Garten Thymian	top	DER	CCLXXIIII / CCCXXI	verzeret die geschwulst so von kalten flüssen kommen
Thymus spp. (T. vulgaris, T. serpyllum)	Lamiaceae	ren	LF	Garten Thymian	top	EYE	CCLXXIIII / CCCXXI	alle, die soe bloede augen haben
Thymus spp. (T. vulgaris, T. serpyllum)	Lamiaceae	ren	LF	Feldthymian	syst	GAS	CXXXIX / XCIII	stillt das grimmen, heylet also innerliche brüch (?) und entzündungen der leber
Thymus spp. (T. vulgaris, T. serpyllum)	Lamiaceae	ren	LF	Feldthymian	syst	GAS	CXXXIX / XCIII	stellet das blutspeien
Thymus spp. (T. vulgaris, T. serpyllum)	Lamiaceae	ren	LF	Garten Thymian	syst	GAS	CCLXXIIII / CCCXXI	treibt er auss durch den stulgang die zaehen feüchtigkeyt / Pituita geheyssen
Thymus spp. (T. vulgaris, T. serpyllum)	Lamiaceae	ren	LF	Garten Thymian	syst	GAS	CCLXXIIII / CCCXXI	macht lust zu essen
Thymus spp. (T. vulgaris, T. serpyllum)	Lamiaceae	ren	LF	Garten Thymian	top	GAS	CCLXXIIII / CCCXXI	vertreibt auch gewaltiglich die blaest und wind im bauch
Thymus spp. (T. vulgaris, T. serpyllum)	Lamiaceae	ren	LF	Garten Thymian	syst	GAS	CCLXXIIII / CCCXXI	trebit aus die würm
Thymus spp. (T. vulgaris, T. serpyllum)	Lamiaceae	ren	LF	Feldthymian	syst	GYN	CXXXIX / XCIII	bringt den frawen ire zeit und treibt den harn
Thymus spp. (T. vulgaris, T. serpyllum)	Lamiaceae	ren	LF	Garten Thymian	syst	GYN	CCLXXIIII / CCCXXI	[treibt aus] das bürdling, bringt den frawen ire zeit
Thymus spp. (T. vulgaris, T. serpyllum)	Lamiaceae	ren	LF	Feldthymian	syst	NER	CXXXIX / XCIII	stillt das haubtwee
Thymus spp. (T. vulgaris, T. serpyllum)	Lamiaceae	ren	LF	Feldthymian	top	NER	CXXXIX / XCIII	benimpt die wuetende haubtsucht / Phyretnitim genent
Thymus spp. (T. vulgaris, T. serpyllum)	Lamiaceae	ren	LF	Feldthymian	top	NER	CXXXIX / XCIII	stillt das haubtwee
Thymus spp. (T. vulgaris, T. serpyllum)	Lamiaceae	ren	LF	Garten Thymian	n.d.	NER	CCLXXIIII / CCCXXI	denen so die fallende sucht haben
Thymus spp. (T. vulgaris, T. serpyllum)	Lamiaceae	ren	LF	Garten Thymian	vol	NER	CCLXXIIII / CCCXXI	denen so die fallende sucht haben
Thymus spp. (T. vulgaris, T. serpyllum)	Lamiaceae	ren	LF	Feldthymian	syst	OTH	CXXXIX / XCIII	damit sie (Schmitter) vor gifftigen thiern sicher weren
Thymus spp. (T. vulgaris, T. serpyllum)	Lamiaceae	ren	LF	Feldthymian	vol	OTH	CXXXIX / XCIII	veriagt allerley gifftige thier
Thymus spp. (T. vulgaris, T. serpyllum)	Lamiaceae	ren	LF	Garten Thymian	syst	RES	CCLXXIIII / CCCXXI	macht ausswerffen
Thymus spp. (T. vulgaris, T. serpyllum)	Lamiaceae	ren	LF	Garten Thymian	syst	RES	CCLXXIIII / CCCXXI	ist bequem und nützlich denen so keichen und einen schweren athemb haben
Thymus spp. (T. vulgaris, T. serpyllum)	Lamiaceae	ren	LF	Garten Thymian	top	SKE	CCLXXIIII / CCCXXI	ist er hilfflich dem hüfftwee
Thymus spp. (T. vulgaris, T. serpyllum)	Lamiaceae	ren	LF	Feldthymian	syst	URO	CXXXIX / XCIII	und treibt den harn
Thymus spp. (T. vulgaris, T. serpyllum)	Lamiaceae	s pop	BVA	Wilder Thymian - Q	syst	NER	0. 230	Nervenschwäche
Thymus spp. (T. vulgaris, T. serpyllum)	Lamiaceae	s pop	BVA	Wilder Thymian - Q	syst	NER	0. 230	Nervenschwäche
Thymus spp. (T. vulgaris, T. serpyllum)	Lamiaceae	s pop	BVA	Wilder Thymian - Q	syst	RES	p. 230	Schnupfen, Bronchitis, Husten, Keuchhusten, Heiserkeit, Grippe, Erkältung, Asthma,
Thymus spp. (T. vulgaris, T. serpyllum)	Lamiaceae	s pop	BVA	Wilder Thymian - Q	syst	RES	p. 230	Schnupfen, Bronchitis, Husten, Keuchhusten, Heiserkeit, Grippe, Erkältung, Asthma,
Thymus spp. (T. vulgaris, T. serpyllum)	Lamiaceae	s pop	BVA	Wilder Thymian - Q	top	SKE	p. 230	Rheuma, Gicht, Gleiderschmerzen
Thymus spp. (T. vulgaris, T. serpyllum)	Lamiaceae	s pop	BVA	Wilder Thymian - Q	top	SKE	p. 230	Gliederschmerzen, Rheumatischen Erkrankungen
Thymus spp. (T. vulgaris, T. serpyllum)	Lamiaceae	s pop	BVA	Wilder Thymian - Q	syst	TON	p. 230	Blutarmut
Thymus spp. (T. vulgaris, T. serpyllum)	Lamiaceae	s pop	BVA	Wilder Thymian - Q	syst	TON	p. 230	Blutarmut
Thymus spp. (T. vulgaris, T. serpyllum)	Lamiaceae	s pop	BVA	Wilder Thymian - Q	top	TON	p. 230	schwächliche Kinder
Thymus spp. (T. vulgaris, T. serpyllum)	Lamiaceae	s pop	BVA	Wilder Thymian - Q	syst	URO	p. 230	Blasenleiden, Entzündungen der Harnwege
Thymus spp. (T. vulgaris, T. serpyllum)	Lamiaceae	s pop	BVA	Wilder Thymian - Q	syst	URO	p. 230	Blasenleiden, Entzündungen der Harnwege
Thymus spp. (T. vulgaris, T. serpyllum)	Lamiaceae	s pop	JK	Thymian	syst	DER	p. 429	Kopfschorf
Thymus spp. (T. vulgaris, T. serpyllum)	Lamiaceae	s pop	JK	Thymian	syst	GAS	p. 429	Magenverschleimung, Magenbrennen, für alle Beschwerden der Leber und der Gedärme, schlecht verdauliche und verdorbene Speisen
Thymus spp. (T. vulgaris, T. serpyllum)	Lamiaceae	s pop	JK	Thymian	top	NER	p. 429	bring guten Schlaf
Thymus spp. (T. vulgaris, T. serpyllum)	Lamiaceae	s pop	JK	Thymian	vol	NER	p. 429	stärkt Nerven von Ohren, Nase und Gehirn, bewahrt vor Ohnmachten und Schlaganfall
Thymus spp. (T. vulgaris, T. serpyllum)	Lamiaceae	s pop	UB	Thymian	top	DER	p. 357	Haut- und Fusspilz
Thymus spp. (T. vulgaris, T. serpyllum)	Lamiaceae	s pop	UB	Quendel	syst	RES	p. 218	Erkältungskrankheiten mit krampfhaftem Husten, Begleittherapie bei Keuchhusten und Asthma
Thymus spp. (T. vulgaris, T. serpyllum)	Lamiaceae	s pop	UB	Quendel	top	RES	p. 218	Erkältungskrankheiten mit krampfhaftem Husten, Begleittherapie bei Keuchhusten und Asthma
Thymus spp. (T. vulgaris, T. serpyllum)	Lamiaceae	s pop	UB	Quendel	vol	RES	p. 218	Erkältungskrankheiten mit krampfhaftem Husten, Begleittherapie bei Keuchhusten und Asthma
Thymus spp. (T. vulgaris, T. serpyllum)	Lamiaceae	s pop	UB	Thymian	syst	RES	p. 219	Auswurffördernd, usw.
Thymus spp. (T. vulgaris, T. serpyllum)	Lamiaceae	s pop	WI	Thymian	syst	GAS	p. 579	Volksmedizin: Stomachikum und Karminativum, Wurmmittel
Thymus spp. (T. vulgaris, T. serpyllum)	Lamiaceae	s pop	WI	Quendel	syst	n.d.	p. 556	Volksmedizin: Kräuterkuren und Bäder
Thymus spp. (T. vulgaris, T. serpyllum)	Lamiaceae	s pop	WI	Quendel	top	SKE	p. 556	Volksmedizin:rheumatischen Schmerzen, Verstauchungen
Thymus spp. (T. vulgaris, T. serpyllum)	Lamiaceae	s pop	WI	Quendel	syst	URO	p. 556	Volksmedizin: Blasen- und Nierenerkrankungen
Thymus spp. (T. vulgaris, T. serpyllum)	Lamiaceae	sci	EMA	Thymian	top	RES	HMPC/255527/2009	coughs and colds
Thymus spp. (T. vulgaris, T. serpyllum)	Lamiaceae	sci	EMA	Thymian	syst	RES	HMPC/342332/2013	productive cough
Thymus spp. (T. vulgaris, T. serpyllum)	Lamiaceae	sci	EMA	Thymian	syst	RES	HMPC/342332/2013	productive cough
Thymus spp. (T. vulgaris, T. serpyllum)	Lamiaceae	sci	ESCOP	Thymi herba	top	DER	I p. 505	stomatitis and halitosis
Thymus spp. (T. vulgaris, T. serpyllum)	Lamiaceae	sci	WI	Thymian	top	DER	p. 579	Entzündungen im Mund- und Rachenraum
Thymus spp. (T. vulgaris, T. serpyllum)	Lamiaceae	sci	WI	Thymian	top	DER	p. 579	hautreizendes Mittel in Einreibungen, Badezusätzen und Kräuterkissen
Triticum aestivum aggr.	Poaceae	ant	DIOS	Weizen	syst	ANT	135	hilft gegen Vipernbiss
Triticum aestivum aggr.	Poaceae	ant	DIOS	Weizen	top	ANT	135	aufgelegt hilft er gegen den Biss des wütenden Hundes
Triticum aestivum aggr.	Poaceae	ant	DIOS	Weizen	top	ANT	135	Das Mehl des Sommerweizens dient mit Essig oder Wein als Umschlag gegen den Biss giftiger Tiere.
Triticum aestivum aggr.	Poaceae	ant	DIOS	Weizen	syst	DER	135	den Aussatz und ist gleich anfangs umgeschlagen ein gutes Mittel gegen jede Entzündung
Triticum aestivum aggr.	Poaceae	ant	DIOS	Weizen	top	DER	135	Mit Sauerhonig entfernt es Leberflecken
Triticum aestivum aggr.	Poaceae	ant	DIOS	Weizen	top	DER	139	Höhlungen (Cavernen) und Pusteln
Triticum aestivum aggr.	Poaceae	ant	DIOS	Weizen	top	DER	135	Sauerteig ist erwärmend und reizend, erweicht aber ganz besonders die Geschwülste an den Fußsohlen, reift und öffnet auch mit Salz die anderen Geschwüre und Furunkeln
Triticum aestivum aggr.	Poaceae	ant	DIOS	Weizen	top	EYE	139	Es wirkt gegen Augenflüsse
Triticum aestivum aggr.	Poaceae	ant	DIOS	Weizen	syst	GAS	135	und trockene, für sich und gemischt mit irgend etwas, stellt den Bauchfluss
Triticum aestivum aggr.	Poaceae	ant	DIOS	Weizen	syst	GAS	139	Blutsturz
Triticum aestivum aggr.	Poaceae	ant	DIOS	Weizen	syst	GAS	135	und Leibschneiden
Triticum aestivum aggr.	Poaceae	ant	DIOS	Weizen	top	GAS	135	Aufblähen der Eingeweide
Triticum aestivum aggr.	Poaceae	ant	DIOS	Weizen	syst	GYN	135	erleichtert sie geschwollene Brüste
Triticum aestivum aggr.	Poaceae	ant	DIOS	Weizen	top	NER	135	Mehl dient mit Bilsenkrautsaft als Umschlag gegen Nerven-(Sehnen-)fluß
Triticum aestivum aggr.	Poaceae	ant	DIOS	Weizen	syst	RES	139	lindert die Schmerzen in der Luftröhre
Triticum aestivum aggr.	Poaceae	ant	DIOS	Weizen	syst	RES	135	hilft es als Leckmittel gegen Blutspeien, wirkt auch mit Pfefferminze und Butter gekocht gegen Husten und Rauheit der Luftröhre
Triticum aestivum aggr.	Poaceae	ant	DIOS	Weizen	syst	RES	135	hilft es als Leckmittel gegen Blutspeien, wirkt auch mit Pfefferminze und Butter gekocht gegen Husten und Rauheit der Luftröhre
Triticum aestivum aggr.	Poaceae	mon	HvB	Weizen	top	DER	1 -1	Wenn aber ein Hund mit den Zähnen einen Menschen beisst
Triticum aestivum aggr.	Poaceae	mon	HvB	Weizen	top	NER	1 -1	Aber wenn jemand ein leeres Gehirn hat und deswegen von Wahnsinn geplagt wird, wie wenn er verrückt wäre
Triticum aestivum aggr.	Poaceae	mon	HvB	Weizen	top	SKE	1 -1	Und wer im Rücken und in den Lenden Schmerzen hat,
Triticum aestivum aggr.	Poaceae	ren	LF	Weizen	top	DER	XIX - CCCLXX / CCLI	nehmen hiweg die raudenund allerley unsauberkeyt des leibs / sie seind auch dieser gestalt gebraucht gut zu den beulen so noch im anfang seind
Triticum aestivum aggr.	Poaceae	ren	LF	Weizen	top	DER	XIX - CCCLXX / CCLI	so man mit gemelltem wasser die biss der schlangen waescht heylt es dieselbigen
Triticum aestivum aggr.	Poaceae	ren	LF	Weizen	top	DER	XIX - CCCLXX / CCLI	legen sie nider die schwolnen brüst
Triticum aestivum aggr.	Poaceae	ren	LF	Weizen	top	DER	XIX - CCCLXX / CCLI	verzeret alle beulen
Triticum aestivum aggr.	Poaceae	ren	LF	Weizen	top	DER	XIX - CCCLXX / CCLI	frisch brodt in wasser und salz gebeyzt vertreibt die flechen und zittermaeler
Triticum aestivum aggr.	Poaceae	ren	LF	Weizen	top	DER	XIX - CCCLXX / CCLI	heylet die biss der hund
Triticum aestivum aggr.	Poaceae	ren	LF	Weizen	top	DER	XIX - CCCLXX / CCLI	vertreibt er die rossmucken under dem angesicht
Triticum aestivum aggr.	Poaceae	ren	LF	Weizen	top	DER	XIX - CCCLXX / CCLI	thut er auff und zeitiget allerley geschwaer
Triticum aestivum aggr.	Poaceae	ren	LF	Weizen	syst	GAS	XIX - CCCLXX / CCLI	das brodt nun so alt ist und hert worden stellt den bauchfluss
Triticum aestivum aggr.	Poaceae	ren	LF	Weizen	syst	GAS	XIX - CCCLXX / CCLI	nützlich denen so blut speien
Triticum aestivum aggr.	Poaceae	ren	LF	Weizen	n.d.	n.d.	XIX - CCCLXX / CCLI	der hefel von Weytzen ist einer warmen ausziehenden krafft
Triticum aestivum aggr.	Poaceae	ren	LF	Weizen	top	NER	XIX - CCCLXX / CCLI	ist gut zu den flüssen des geaeders oder der nerven
Triticum aestivum aggr.	Poaceae	ren	LF	Weizen	top	DER	XIX - CCCLXX / CCLI	heylt die geschwulst der mandeln

Triticum aestivum aggr.	Poaceae	ren	LF	Weizen	top	SKE	XIX - CCCLXX / CCLI	podagra
Triticum aestivum aggr.	Poaceae	ren	LF	Weizen	top	SKE	XIX - CCCLXX / CCLI	seind gut zu den brüchen
Triticum aestivum aggr.	Poaceae	s pop	UB	Weizen	top	DER	p. 338	Neurodermitis
Tussilago farfara L.	Asteraceae	ant	DIOS	Huflattich	top	DER	200	als Umschlag heilen roseartige und alle anderen Entzündungen
Tussilago farfara L.	Asteraceae	ant	DIOS	Huflattich	vol	DER	200	Es öffnet aber auch die Absetzesse in der Brust
Tussilago farfara L.	Asteraceae	ant	DIOS	Huflattich	vol	DER	200	Es öffnet aber auch die Absetzesse in der Brust
Tussilago farfara L.	Asteraceae	ant	DIOS	Huflattich	syst	GYN	200	Sie treibt in Honigwasser gekocht und getrunken den toten Embryo aus
Tussilago farfara L.	Asteraceae	ant	DIOS	Huflattich	vol	RES	200	trockenem Husten und Orthopnöe belästigt worden, wenn sie den Dampf mit geöffnetem Munde aufnehmen und herunterschlucken
Tussilago farfara L.	Asteraceae	ant	DIOS	Huflattich	vol	RES	200	trockenem Husten und Orthopnöe belästigt worden, wenn sie den Dampf mit geöffnetem Munde aufnehmen und herunterschlucken
Tussilago farfara L.	Asteraceae	mon	HvB	Huflattich	syst	GAS	1-211	von vielerlei Speisen unmässig gegessen, wird seine Leber geschädigt
Tussilago farfara L.	Asteraceae	ren	LF	Huflattich	top	DER	LXXVI / L	leschen allerley hitz und heylen das rotlauff
Tussilago farfara L.	Asteraceae	ren	LF	Huflattich	top	n.d.	LXXVI / L	artzney seien zu dem brandt
Tussilago farfara L.	Asteraceae	ren	LF	Huflattich	vol	RES	LXXVI / L	heylen sie die trucknen husten unnd das keichen ider enge des athembs / sie brechen auch die apostem der brust
Tussilago farfara L.	Asteraceae	ren	LF	Huflattich	vol	RES	LXXVI / L	gleiche krafft hat auch die wurtzel [heylen sie die trucknen husten unnd das keichen ider enge des athembs / sie brechen auch die apostem der brust]
Tussilago farfara L.	Asteraceae	s pop	BVA	Huflattich	syst	RES	p. 103	Erkrankung der Atmungsorgane, Husten, Verschleimung, Hieserkeit, Katarrh, Bronchitis, Engbrüstigkeit, Keuchhusten, Bronchialastmah
Tussilago farfara L.	Asteraceae	s pop	BVA	Huflattich	syst	RES	p. 103	Erkrankung der Atmungsorgane, Husten, Verschleimung, Hieserkeit, Katarrh, Bronchitis, Engbrüstigkeit, Keuchhusten, Bronchialastmah
Tussilago farfara L.	Asteraceae	s pop	BVA	Huflattich	syst	RES	p. 103	Erkrankung der Atmungsorgane, Husten, Verschleimung, Hieserkeit, Katarrh, Bronchitis, Engbrüstigkeit, Keuchhusten, Bronchialastmah
Tussilago farfara L.	Asteraceae	s pop	JK	Huflattich	top	DER	p. 376	offene Wunden, offene Beine, Gesichtsrose, Hitze auf der Brust und in der Nierengegend, Krampfadern, Geschwüren und Hühneraugen
Tussilago farfara L.	Asteraceae	s pop	JK	Huflattich	vol	OTH	p. 376	undschädlicher Tabak
Tussilago farfara L.	Asteraceae	s pop	JK	Huflattich	top	SKE	p. 376	Quetschungen, Verrenkungen
Tussilago farfara L.	Asteraceae	s pop	KN	Huflattich	top	DER	p. 474	auf offene Geschwüre, offene Füsse, Rotlauf,Gesichtsrose
Tussilago farfara L.	Asteraceae	s pop	KN	Huflattich	syst	GAS	p. 474	Wirkung auf Magen un Darm
Tussilago farfara L.	Asteraceae	s pop	KN	Huflattich	syst	RES	p. 474	reinigen der Brust und zum Säubern der Lungen; Engbrüstigkeit und Husten
Tussilago farfara L.	Asteraceae	s pop	KN	Huflattich	top	RES	p. 474	auf ein Tuch geheftet und auf die Brust gelegt
Tussilago farfara L.	Asteraceae	s pop	MT	Huflattich	top	EAR	p. 24	Ohrenschmerzen
Tussilago farfara L.	Asteraceae	s pop	MT	Huflattich	vol	RES	p. 24	Bronchitis, erstickender Atemnot
Tussilago farfara L.	Asteraceae	s pop	MT	Huflattich	top	RES	p. 24	schwere Lungenerkrankung
Tussilago farfara L.	Asteraceae	s pop	UB	Huflattich	syst	RES	p. 211	Husten
Tussilago farfara L.	Asteraceae	sci	WI	Huflattich	syst	RES	p. 215	katarrhalischen Entzündungen, trockenem Reizhusten usw.
Urtica dioica L.	Urticaceae	ant	DIOS	Brennnessel	top	ANT	237	Die Blätter beider, mit Salz als Kataplasma, heilen Hundsbisse [und Gangrän, böse, krebsartige und schmutzige Geschwüre, sowie Verrenkungen, Skrofeln, Drüsen an den Ohren und an den Schamtheilen und Abscesse]
Urtica dioica L.	Urticaceae	ant	DIOS	Brennnessel	syst	APH	237	reizt zum Beischlaf und öffnet die Gebärmutter
Urtica dioica L.	Urticaceae	ant	DIOS	Brennnessel	top	DER	237	Die Blätter beider, mit Salz als Kataplasma, heilen [Hundsbisse] und Gangrän, böse, krebsartige und schmutzige Geschwüre, sowie [Verrenkungen], Skrofeln, Drüsen an den Ohren und an den Schamtheilen und Abscesse
Urtica dioica L.	Urticaceae	ant	DIOS	Brennnessel	top	DER	237	helfen auch gegen Nasenbluten
Urtica dioica L.	Urticaceae	ant	DIOS	Brennnessel	top	DER	237	beseitigt die Entzündung des Zäpfchens
Urtica dioica L.	Urticaceae	ant	DIOS	Brennnessel	syst	GAS	237	erweichen die Blätter den Bauch, vertreiben Blähungen
Urtica dioica L.	Urticaceae	ant	DIOS	Brennnessel	top	GYN	237	Gebärmuttervorfall in Ordnung
Urtica dioica L.	Urticaceae	ant	DIOS	Brennnessel	top	GYN	237	befördern sie mit Myrrhe im Zäpfchen die Menstruation
Urtica dioica L.	Urticaceae	ant	DIOS	Brennnessel	syst	GYN	237	[reizt zum Beischlaf] und öffnet die Gebärmutter
Urtica dioica L.	Urticaceae	ant	DIOS	Brennnessel	syst	GYN	237	befördert die Katamenien
Urtica dioica L.	Urticaceae	ant	DIOS	Brennnessel	top	OTH	237	Milzkranken werden sie mit Wachssalbe
Urtica dioica L.	Urticaceae	ant	DIOS	Brennnessel	syst	RES	237	mit Honig als Leckmittel hilft er bei Orthopnöe, Lungen- und Brustfellentzündung, führt die Unreinigkeiten aus der Brust und wird den fäulnisswidrigen Mitteln zugesetzt
Urtica dioica L.	Urticaceae	ant	DIOS	Brennnessel	syst	RES	237	Ptisane gekocht reinigen sie die Brust
Urtica dioica L.	Urticaceae	ant	DIOS	Brennnessel	top	SKE	237	Die Blätter beider, mit Salz als Kataplasma, heilen [Hundsbisse und Gangrän, böse, krebsartige und schmutzige Geschwüre, sowie]Verrenkungen, [Skrofeln, Drüsen an den Ohren und an den Schamtheilen und Abscesse]
Urtica dioica L.	Urticaceae	ant	DIOS	Brennnessel	syst	URO	237	und treiben den Harn
Urtica dioica L.	Urticaceae	mon	HvB	Brennnessel	syst	GAS	1-100	reinigt den Magen
Urtica dioica L.	Urticaceae	mon	MF	Brennnessel	syst	APH	4	erregt Liebeskraft
Urtica dioica L.	Urticaceae	mon	MF	Brennnessel	syst	DER	4	fressende Geschwüre, eiternde Wunden, wirkt auch bei Hundebissen, vom Knochen abgefallenes Fleisch wir wieder hergestellt
Urtica dioica L.	Urticaceae	mon	MF	Brennnessel	syst	DER	4	Blutstillung
Urtica dioica L.	Urticaceae	mon	MF	Brennnessel	syst	DER	4	grosses Geschwulst des Rachenzäpfchens mässigen
Urtica dioica L.	Urticaceae	mon	MF	Brennnessel	syst	DER	4	heilt Krebs und Mumps
Urtica dioica L.	Urticaceae	mon	MF	Brennnessel	top	DER	4	in Nase eingeführt, lockt Saft oder auch nur ein Nesselblatt das Blut hervor
Urtica dioica L.	Urticaceae	mon	MF	Brennnessel	syst	GAS	4	treibt Windblähungen aus dem Bauch
Urtica dioica L.	Urticaceae	mon	MF	Brennnessel	syst	GAS	4	treibt Windblähungen aus dem Bauch
Urtica dioica L.	Urticaceae	mon	MF	Brennnessel	syst	GAS	4	Gelbsüchtigen
Urtica dioica L.	Urticaceae	mon	MF	Brennnessel	syst	GAS	4	Bauchgrimmen
Urtica dioica L.	Urticaceae	mon	MF	Brennnessel	syst	GYN	4	falls Gebärmutter, durch Körpersaft beschwert, die weibliche Scham schiesst . . .
Urtica dioica L.	Urticaceae	mon	MF	Brennnessel	syst	GYN	4	bringt Monatsblutung in Bewegung
Urtica dioica L.	Urticaceae	mon	MF	Brennnessel	syst	HUM	4	schädliche Körpersäfte zu trocknen
Urtica dioica L.	Urticaceae	mon	MF	Brennnessel	syst	HUM	4	salbt man den Kranken ein, so erregt das den Schweiss
Urtica dioica L.	Urticaceae	mon	MF	Brennnessel	syst	n.d.	4	erweicht es den Leib
Urtica dioica L.	Urticaceae	mon	MF	Brennnessel	syst	OTH	4	Milzschwellung
Urtica dioica L.	Urticaceae	mon	MF	Brennnessel	syst	RES	4	treibt Kälte aus den Lungen
Urtica dioica L.	Urticaceae	mon	MF	Brennnessel	syst	RES	4	treibt Kälte aus den Lungen
Urtica dioica L.	Urticaceae	mon	MF	Brennnessel	syst	RES	4	verstockter Husten
Urtica dioica L.	Urticaceae	mon	MF	Brennnessel	syst	RES	4	Galen spricht noch: wenn du das Haupt oftmals mit Saft des Nesselsamens salbst, wird der Katarrh zurück gedrängt
Urtica dioica L.	Urticaceae	mon	MF	Brennnessel	syst	RES	4	Rippenfellschmerzen, Brust- und Lungenkranke
Urtica dioica L.	Urticaceae	mon	MF	Brennnessel	syst	SKE	4	verrenkte Glieder
Urtica dioica L.	Urticaceae	mon	MF	Brennnessel	syst	SKE	4	kranke Glieder
Urtica dioica L.	Urticaceae	mon	MF	Brennnessel	syst	SKE	4	lindert die Fussgicht (Podagra), hilft bei allen Leiden der Gelenke
Urtica dioica L.	Urticaceae	mon	MF	Brennnessel	syst	URO	4	führt er Harnfluss im Übermass herbei
Urtica dioica L.	Urticaceae	ren	LF	Brennnessel	n.d.	ANT	LVII - LIX / XXXVII	ist ganz zuwider dem wutzerling un gifftigen schwammen
Urtica dioica L.	Urticaceae	ren	LF	Brennnessel	syst	APH	LVII - LIX / XXXVII	reitzet zur unkeüschheyt
Urtica dioica L.	Urticaceae	ren	LF	Brennnessel	top	DER	LVII - LIX / XXXVII	heylen die biss der unsinnigen hünd un die grosen geschwer / des gleichen über faule schaeden als Krebs unnd dergleichen gelegt reynigen sie die selbigen unnd heylens / in gleicher massen zerteylen sie auch allerley geschwulst also ormützel und dergeichen
Urtica dioica L.	Urticaceae	ren	LF	Brennnessel	top	DER	LVII - LIX / XXXVII	ist gut zu dem geschwollen zepfflin
Urtica dioica L.	Urticaceae	ren	LF	Brennnessel	top	DER	LVII - LIX / XXXVII	vertreibt das har aussfallen unnd den boesen grind
Urtica dioica L.	Urticaceae	ren	LF	Brennnessel	top	DER	LVII - LIX / XXXVII	desgleichen auch der sam [vertreibt das har aussfallen unnd den boesen grind]
Urtica dioica L.	Urticaceae	ren	LF	Brennnessel	syst	GAS	LVII - LIX / XXXVII	lindern den stulgang

Urtica dioica L.	Urticaceae	ren	LF	Brennnessel	syst	GAS	LVII - LIX / XXXVII	macht leicht speyen
Urtica dioica L.	Urticaceae	ren	LF	Brennnessel	syst	GAS	LVII - LIX / XXXVII	ist gut zu dem auffbleen des magens
Urtica dioica L.	Urticaceae	ren	LF	Brennnessel	syst	GYN	LVII - LIX / XXXVII	so bringen sie den frawen ihr krankheyt
Urtica dioica L.	Urticaceae	ren	LF	Brennnessel	top	GYN	LVII - LIX / XXXVII	über die muter gelegt die heraus begert so treiben sie dieselbigen wider hinder sich
Urtica dioica L.	Urticaceae	ren	LF	Brennnessel	top	GYN	LVII - LIX / XXXVII	bringen sie den frawen ir bloedigkeit
Urtica dioica L.	Urticaceae	ren	LF	Brennnessel	syst	GYN	LVII - LIX / XXXVII	und er eröffnet die muter
Urtica dioica L.	Urticaceae	ren	LF	Brennnessel	top	RES	LVII - LIX / XXXVII	über die stirn gelegt stellen das schweyssen zu der nasen auss.
Urtica dioica L.	Urticaceae	ren	LF	Brennnessel	syst	RES	LVII - LIX / XXXVII	ist gut für das keichen, seiten oder rippen unnnd lungen geschwer / er macht auch auswerffen und reyniget die brust
Urtica dioica L.	Urticaceae	ren	LF	Brennnessel	top	SKE	LVII - LIX / XXXVII	ist gut zu dem podagra und allerley weetagen der glider
Urtica dioica L.	Urticaceae	ren	LF	Brennnessel	syst	URO	LVII - LIX / XXXVII	treiben den harn
Urtica dioica L.	Urticaceae	s pop	BVA	Brennnessel	syst	DER	p. 46	Hautunreinigkeiten
Urtica dioica L.	Urticaceae	s pop	BVA	Brennnessel	syst	DER	p. 46	Hautunreinigkeiten
Urtica dioica L.	Urticaceae	s pop	BVA	Brennnessel	syst	GAS	p. 46	träger Darmperistaltik, Magen-Darm-Geschwürsneigung
Urtica dioica L.	Urticaceae	s pop	BVA	Brennnessel	syst	GAS	p. 46	träger Darmperistaltik, Magen-Darm-Geschwürsneigung
Urtica dioica L.	Urticaceae	s pop	BVA	Brennnessel	syst	GYN	p. 46	Menstruationsbeschwerden
Urtica dioica L.	Urticaceae	s pop	BVA	Brennnessel	syst	GYN	p. 46	Menstruationsbeschwerden
Urtica dioica L.	Urticaceae	s pop	BVA	Brennnessel	syst	GYN	p. 46	Milchbildung bei stillenden Müttern
Urtica dioica L.	Urticaceae	s pop	BVA	Brennnessel	syst	HUM	p. 46	Blutreinigung, Entschlackung,
Urtica dioica L.	Urticaceae	s pop	BVA	Brennnessel	syst	HUM	p. 46	Blutreinigung, Entschlackung,
Urtica dioica L.	Urticaceae	s pop	BVA	Brennnessel	syst	NER	p. 46	Erschöpfung, Leistungsschwäche, Gedächtnisschwäche
Urtica dioica L.	Urticaceae	s pop	BVA	Brennnessel	syst	OTH	p. 46	Stoffwechselstörungen
Urtica dioica L.	Urticaceae	s pop	BVA	Brennnessel	syst	OTH	p. 46	Stoffwechselstörungen
Urtica dioica L.	Urticaceae	s pop	BVA	Brennnessel	syst	SKE	p. 46	Entgiftung Rheuma und Gicht
Urtica dioica L.	Urticaceae	s pop	BVA	Brennnessel	syst	SKE	p. 46	Entgiftung Rheuma und Gicht
Urtica dioica L.	Urticaceae	s pop	BVA	Brennnessel	syst	TON	p. 46	Blutarmut
Urtica dioica L.	Urticaceae	s pop	BVA	Brennnessel	syst	TON	p. 46	Blutarmut
Urtica dioica L.	Urticaceae	s pop	BVA	Brennnessel	syst	URO	p. 46	Erkrankungen der Harnwege, Nierengriess
Urtica dioica L.	Urticaceae	s pop	BVA	Brennnessel	syst	URO	p. 46	Erkrankungen der Harnwege, Nierengriess
Urtica dioica L.	Urticaceae	s pop	JK	Brennnessel	top	DER	p. 339	Anschwellung des Zahnfleisches, Hundebiss, Eissen, Furunkeln und hartne Knollen
Urtica dioica L.	Urticaceae	s pop	JK	Brennnessel	top	DER	p. 339	Erfrischung und belebung der Kopfhaut
Urtica dioica L.	Urticaceae	s pop	JK	Brennnessel	syst	GAS	p. 339	Magengeschwüre und Geschwüre in Leber und Därmen
Urtica dioica L.	Urticaceae	s pop	JK	Brennnessel	syst	HUM	p. 339	Blutreinigung und blutbildend, Frühjahrskur
Urtica dioica L.	Urticaceae	s pop	JK	Brennnessel	syst	NER	p. 339	nervöse Menschen, Mittel stärkt auch das Gehirn
Urtica dioica L.	Urticaceae	s pop	JK	Brennnessel	top	SKE	p. 339	Rheumatismus, an einem Glied den Schwund
Urtica dioica L.	Urticaceae	s pop	JK	Brennnessel	syst	URO	p. 339	wassertreibend, Wassersucht
Urtica dioica L.	Urticaceae	s pop	KN	Brennnessel	syst	GAS	p. 462	räumt mit faueln Säften im Inneren gründlich auf
Urtica dioica L.	Urticaceae	s pop	KN	Brennnessel	syst	HUM	p. 462	unreines Blut, Frühjahrskur
Urtica dioica L.	Urticaceae	s pop	KN	Brennnessel	syst	RES	p. 462	lösen die Verschleimung der Brust
Urtica dioica L.	Urticaceae	s pop	KN	Brennnessel	top	SKE	p. 462	Rheumatismus
Urtica dioica L.	Urticaceae	s pop	KN	Brennnessel	syst	URO	p. 462	wirken auf die Nierenfunktion
Urtica dioica L.	Urticaceae	s pop	KN	Brennnessel	syst	URO	p. 462	eine beginnende Wassersucht . . . Behoben werden
Urtica dioica L.	Urticaceae	s pop	MT	Brennnessel	top	CAR	p. 14	
Urtica dioica L.	Urticaceae	s pop	MT	Brennnessel	syst	DER	p. 14	
Urtica dioica L.	Urticaceae	s pop	MT	Brennnessel	syst	GAS	p. 14	
Urtica dioica L.	Urticaceae	s pop	MT	Brennnessel	syst	OTH	p. 14	
Urtica dioica L.	Urticaceae	s pop	MT	Brennnessel	syst	RES	p. 14	
Urtica dioica L.	Urticaceae	s pop	MT	Brennnessel	top	SKE	p. 14	
Urtica dioica L.	Urticaceae	s pop	MT	Brennnessel	syst	TON	p. 14	
Urtica dioica L.	Urticaceae	s pop	MT	Brennnessel	syst	URO	p. 14	
Urtica dioica L.	Urticaceae	s pop	UB	Brennnessel	syst	SKE	p. 276	Arthrose
Urtica dioica L.	Urticaceae	s pop	UB	Brennnessel	syst	SKE	p. 276	Arthrose
Urtica dioica L.	Urticaceae	s pop	UB	Brennnessel	syst	SKE	p. 276, 281	Arthrose, entzündliche rheumatische Beschwerden
Urtica dioica L.	Urticaceae	s pop	UB	Brennnessel	top	SKE	p. 276	Arthrose
Urtica dioica L.	Urticaceae	s pop	UB	Brennnessel	syst	URO	p. 253, 262, 459	Durchspülung, Prostata, Aktivierung der Ausscheidung
Urtica dioica L.	Urticaceae	s pop	UB	Brennnessel	syst	URO	p. 253, 262, 459	Durchspülung, Prostata, Aktivierung der Ausscheidung
Urtica dioica L.	Urticaceae	s pop	UB	Brennnessel	syst	URO	p. 253	Durchspülung
Urtica dioica L.	Urticaceae	s pop	WI	Brennnessel	top	DER	p. 591	Volksmedizin: Hautleiden
Urtica dioica L.	Urticaceae	s pop	WI	Brennnessel	top	DER	p. 591	Volksmedizin: Pflege von Kopfhaut, Schuppen
Urtica dioica L.	Urticaceae	s pop	WI	Brennnessel	syst	GAS	p. 591	Volksmedizin: Gallenwegserkrankungen
Urtica dioica L.	Urticaceae	s pop	WI	Brennnessel	syst	OTH	p. 591	Volksmedizin: Enzymproduktion der Bauchspeicheldrüse, antidiabetische Tees
Urtica dioica L.	Urticaceae	s pop	WI	Brennnessel	top	SKE	p. 591	Volksmedizin: Rheuma
Urtica dioica L.	Urticaceae	s pop	WI	Brennnessel	syst	SKE	p. 591	Volksmedizin: Arthritis, Gelenk- Und Muskelrheumatismus
Urtica dioica L.	Urticaceae	s pop	WI	Brennnessel	syst	TON	p. 591	Volksmedizin: Tonikum und sog. "Biostimulans"
Urtica dioica L.	Urticaceae	sci	EMA	Brennnessel	syst	DER	HMPC/170261/2006	Traditional herbal medicinal product used in seborrhoeic skin conditions
Urtica dioica L.	Urticaceae	sci	EMA	Brennnessel	syst	SKE	HMPC/170261/2006	Traditional herbal medicinal product for relief of minor articular pain
Urtica dioica L.	Urticaceae	sci	EMA	Brennnessel	syst	SKE	HMPC/170261/2006	Traditional herbal medicinal product for relief of minor articular pain
Urtica dioica L.	Urticaceae	sci	EMA	Brennnessel	syst	SKE	HMPC/170261/2006	Traditional herbal medicinal product for relief of minor articular pain
Urtica dioica L.	Urticaceae	sci	EMA	Brennnessel	syst	SKE	HMPC/170261/2006	Traditional herbal medicinal product for relief of minor articular pain
Urtica dioica L.	Urticaceae	sci	EMA	Brennnessel	syst	SKE	HMPC/508015/2007	Traditional herbal medicinal product for relief of minor articular pain.
Urtica dioica L.	Urticaceae	sci	EMA	Brennnessel	syst	SKE	HMPC/508015/2007	Traditional herbal medicinal product for relief of minor articular pain.
Urtica dioica L.	Urticaceae	sci	EMA	Brennnessel	syst	URO	HMPC/170261/2006	Traditional herbal medicinal product to increase the amount of urine to achieve flushing of the urinary tract as an adjuvant in minor urinary complaints.
Urtica dioica L.	Urticaceae	sci	EMA	Brennnessel	syst	URO	HMPC/170261/2006	Traditional herbal medicinal product to increase the amount of urine to achieve flushing of the urinary tract as an adjuvant in minor urinary complaints.
Urtica dioica L.	Urticaceae	sci	EMA	Brennnessel	syst	URO	HMPC/170261/2006	Traditional herbal medicinal product to increase the amount of urine to achieve flushing of the urinary tract as an adjuvant in minor urinary complaints.
Urtica dioica L.	Urticaceae	sci	EMA	Brennnessel	syst	URO	HMPC/461160/2008	Traditional herbal medicinal product for the relief of lower urinary tract symptoms related to benign prostatic hyperplasia after serious conditions have been excluded by a medical doctor.
Urtica dioica L.	Urticaceae	sci	EMA	Brennnessel	syst	URO	HMPC/461160/2008	Traditional herbal medicinal product for the relief of lower urinary tract symptoms related to benign prostatic hyperplasia after serious conditions have been excluded by a medical doctor.
Urtica dioica L.	Urticaceae	sci	ESCOP	Urticae radix	syst	URO	I p. 528	Symptomatic treatment of micturition disorders in benign prostatic hyperplasia
Urtica dioica L.	Urticaceae	sci	WI	Brennnessel	syst	URO	p. 591	Diuretikum
Urtica dioica L.	Urticaceae	sci	WI	Brennnessel	syst	URO	p. 591	Diuretikum
Urtica dioica L.	Urticaceae	sci	WI	Brennnessel	syst	URO	p. 591	Diuretikum
Valeriana officinalis aggr.	Caprifoliaceae	ant	DIOS	Baldrian	syst	ANT	71	wird den Gegengiften zugemischt
Valeriana officinalis aggr.	Caprifoliaceae	ant	DIOS	Baldrian	syst	GYN	71	befördert die Katamenien
Valeriana officinalis aggr.	Caprifoliaceae	ant	DIOS	Baldrian	syst	OTH	71	und wirkt gegen Seitenschmerz
Valeriana officinalis aggr.	Caprifoliaceae	ant	DIOS	Baldrian	syst	URO	71	und den Urin zu treiben
Valeriana officinalis aggr.	Caprifoliaceae	ant	DIOS	Baldrian	syst	URO	71	leistet dasselbe [und den Urin zu treiben]
Valeriana officinalis aggr.	Caprifoliaceae	mon	HvB	Baldrian	syst	SKE	1-142	Brustfellentzündung, von der Gicht Schmerzen
Valeriana officinalis aggr.	Caprifoliaceae	ren	LF	Baldrian	syst	ANT	CCCCXCIII / CCCXXX	under die artzneyen vermischt so manfür gifft braucht, darumb ist sie zu der zeut der Oestilenz seer gut davon getruncken
Valeriana officinalis aggr.	Caprifoliaceae	ren	LF	Baldrian	n.d.	DER	CCCCXCIII / CCCXXX	ist auch trefflich gut den den wunden und schaeden dan sie heylet dieselbigen
Valeriana officinalis aggr.	Caprifoliaceae	ren	LF	Baldrian	top	EYE	CCCCXCIII / CCCXXX	macht ein klar gesicht
Valeriana officinalis aggr.	Caprifoliaceae	ren	LF	Baldrian	syst	GYN	CCCCXCIII / CCCXXX	bringt den frawen ihre zeit
Valeriana officinalis aggr.	Caprifoliaceae	ren	LF	Baldrian	syst	n.d.	CCCCXCIII / CCCXXX	stillt den weetagen der seiten
Valeriana officinalis aggr.	Caprifoliaceae	ren	LF	Baldrian	syst	URO	CCCCXCIII / CCCXXX	treibt den harn
Valeriana officinalis aggr.	Caprifoliaceae	ren	LF	Baldrian	syst	URO	CCCCXCIII / CCCXXX	treibt den harn
Valeriana officinalis aggr.	Caprifoliaceae	s pop	BVA	Baldrian	syst	GAS	p. 34	Krampfzustände im Magen-, Darmbereich, krampfartiges Erbrechen,

Valeriana officinalis aggr.	Caprifoliaceae	s pop	BVA	Baldrian	syst	GAS	p. 34	Krampfzustände im Magen-, Darmbereich, krampfartiges Erbrechen,
Valeriana officinalis aggr.	Caprifoliaceae	s pop	BVA	Baldrian	syst	GYN	p. 34	Wechseljahrbeschwerden, krampfartige Menstruation,
Valeriana officinalis aggr.	Caprifoliaceae	s pop	BVA	Baldrian	syst	GYN	p. 34	Wechseljahrbeschwerden, krampfartige Menstruation,
Valeriana officinalis aggr.	Caprifoliaceae	s pop	BVA	Baldrian	syst	NER	p. 34	Schlaflosigkeit, Nervosität, nervöse Herzstörungen, Herzklopfen, nervöse Kopfschmerzen, Migräne, Angst, Erregunszustände, nervöse Kinder
Valeriana officinalis aggr.	Caprifoliaceae	s pop	BVA	Baldrian	syst	NER	p. 34	Schlaflosigkeit, Nervosität, nervöse Herzstörungen, Herzklopfen, nervöse Kopfschmerzen, Migräne, Angst, Erregunszustände, nervöse Kinder
Valeriana officinalis aggr.	Caprifoliaceae	s pop	BVA	Baldrian	syst	NER	p. 34	Schlaflosigkeit, Nervosität, nervöse Herzstörungen, Herzklopfen, nervöse Kopfschmerzen, Migräne, Angst, Erregunszustände, nervöse Kinder
Valeriana officinalis aggr.	Caprifoliaceae	s pop	BVA	Baldrian	syst	OTH	p. 34	Schilddrüsenüberfunktion
Valeriana officinalis aggr.	Caprifoliaceae	s pop	BVA	Baldrian	syst	OTH	p. 34	Schilddrüsenüberfunktion
Valeriana officinalis aggr.	Caprifoliaceae	s pop	BVA	Baldrian	syst	OTH	p. 34	Abgewöhnung von Alkohol, Nikotin
Valeriana officinalis aggr.	Caprifoliaceae	s pop	BVA	Baldrian	syst	OTH	p. 34	Abgewöhnung von Alkohol, Nikotin
Valeriana officinalis aggr.	Caprifoliaceae	s pop	JK	Baldrian	syst	CAR	p. 328	Herzschwäche
Valeriana officinalis aggr.	Caprifoliaceae	s pop	JK	Baldrian	syst	EYE	p. 328	schwachen Augen
Valeriana officinalis aggr.	Caprifoliaceae	s pop	JK	Baldrian	syst	GAS	p. 328	reinigt Leber, Milz und Galle
Valeriana officinalis aggr.	Caprifoliaceae	s pop	JK	Baldrian	top	NER	p. 328	vertreibt Kopfschmerzen
Valeriana officinalis aggr.	Caprifoliaceae	s pop	JK	Baldrian	syst	NER	p. 328	bei allen nervösen Leiden
Valeriana officinalis aggr.	Caprifoliaceae	s pop	JK	Baldrian	syst	RES	p. 328	Verschleimung und Atembeschwerden
Valeriana officinalis aggr.	Caprifoliaceae	s pop	JK	Baldrian	top	SKE	p. 328	Gicht
Valeriana officinalis aggr.	Caprifoliaceae	s pop	JK	Baldrian	top	URO	p. 328	Blasensteinen
Valeriana officinalis aggr.	Caprifoliaceae	s pop	JK	Baldrian	syst	URO	p. 328	treibt den Urin; führt sogar den Stein aus
Valeriana officinalis aggr.	Caprifoliaceae	s pop	KN	Baldrian	syst	CAR	p. 460	werden Zustände von Herzunruhe mit Herzklopfen
Valeriana officinalis aggr.	Caprifoliaceae	s pop	KN	Baldrian	syst	NER	p. 460	Baldriantee beeinflusst das Nervensystem; Verwendung als Schlafmittel
Valeriana officinalis aggr.	Caprifoliaceae	s pop	KN	Baldrian	syst	NER	p. 460	Baldriantee beeinflusst das Nervensystem; Verwendung als Schlafmittel
Valeriana officinalis aggr.	Caprifoliaceae	s pop	KN	Baldrian	syst	NER	p. 460	lindert Kopfbeschwerden und behebt krampfhafte Zustände . . . Weils sie deren hauptsächlichste Ursache, die Gase nämlich ausscheidet
Valeriana officinalis aggr.	Caprifoliaceae	s pop	UB	Baldrian	syst	NER	p. 419	Nervöse Erregungs- und Unruhezustände
Valeriana officinalis aggr.	Caprifoliaceae	s pop	UB	Baldrian	top	NER	p. 419	Nervöse Erregungs- und Unruhezustände
Valeriana officinalis aggr.	Caprifoliaceae	s pop	UB	Baldrian	top	NER	p. 419	Nervöse Erregungs- und Unruhezustände
Valeriana officinalis aggr.	Caprifoliaceae	sci	EMA	Baldrian	syst	NER	HMPC/340779/2005	mental relxation and normal sleep
Valeriana officinalis aggr.	Caprifoliaceae	sci	EMA	Baldrian	syst	NER	HMPC/340779/2005	mental relxation and normal sleep
Valeriana officinalis aggr.	Caprifoliaceae	sci	EMA	Baldrian	syst	NER	HMPC/340779/2005	mental relxation and normal sleep
Valeriana officinalis aggr.	Caprifoliaceae	sci	WI	Baldrian	syst	NER	p. 604	sedierende Wirkung
Veratrum album aggr.	Melanthiaceae	ant	DIOS	Weisser Germer	n.d.	EYE	249	Er wird auch den Kollyrien zugesetzt, welche die Verdunkelungen von den Augen zu vertreiben vermögen
Veratrum album aggr.	Melanthiaceae	ant	DIOS	Weisser Germer	syst	GYN	249	Ferner befördert er die Menstruation, tödtet, im Zäpfchen eingelegt, den Embryo
Veratrum album aggr.	Melanthiaceae	ant	DIOS	Weisser Germer	syst	HUM	249	Er reinigt durch Erbrechen
Veratrum album aggr.	Melanthiaceae	ant	DIOS	Weisser Germer	syst	HUM	249	Auch die aus ihm gefertigten Zäpfchen, mit Essig dem After eingeführt, bewirken Erbrechen.
Veratrum album aggr.	Melanthiaceae	ant	DIOS	Weisser Germer	n.d.	OTH	249	erregt Niesen
Veratrum album aggr.	Melanthiaceae	mon	HvB	Germer	top	CAR	1-130	aber auch ein Mensch, der vom Herzen und in "vobin" Schmerzen leidet
Veratrum album aggr.	Melanthiaceae	mon	HvB	Germer	top	GYN	1-130	Und ein junges Mädchen, dem der Monatsfluss zur rechten Zeit ausbleibt
Veratrum album aggr.	Melanthiaceae	mon	HvB	Germer	top	NER	1-130	vertreibt . . . Sogar den Wahnsinnim Menschen
Veratrum album aggr.	Melanthiaceae	ren	LF	Germer	syst	CAR	CLI / CIIII	wassersucht
Veratrum album aggr.	Melanthiaceae	ren	LF	Germer	syst	DER	CLI / CIIII	aussatz
Veratrum album aggr.	Melanthiaceae	ren	LF	Germer	top	DER	CLI / CIIII	geflechten, rauden unnd allerley unreynigkeyt die sich aufwendig am leib erzeygt / Die wurtzek würt auch zu den geschwaeren grinden und alten schaeden dieselbigen darmit zu reynigen un seübern
Veratrum album aggr.	Melanthiaceae	ren	LF	Germer	top	EYE	CLI / CIIII	zu den artzneyen darmit man die augen klar und lauter macht
Veratrum album aggr.	Melanthiaceae	ren	LF	Germer	syst	GAS	CLI / CIIII	macht speien und treibt mit grossem gewalt mancherley feüchtigkeyt oben hinaus durch das undewen
Veratrum album aggr.	Melanthiaceae	ren	LF	Germer	syst	GAS	CLI / CIIII	macht speien
Veratrum album aggr.	Melanthiaceae	ren	LF	Germer	syst	GAS	CLI / CIIII	treibt dieser rettich zu dem stulgang
Veratrum album aggr.	Melanthiaceae	ren	LF	Germer	top	GYN	CLI / CIIII	bringt den frawen ire zeit
Veratrum album aggr.	Melanthiaceae	ren	LF	Germer	top	HUM	CLI / CIIII	in die nasen gethon reynigt das hirn und macht niessen
Veratrum album aggr.	Melanthiaceae	ren	LF	Germer	syst	NER	CLI / CIIII	die fallende sucht,schwindel, unsinnigkeyt, . . ., krampff
Veratrum album aggr.	Melanthiaceae	ren	LF	Germer	n.d.	OTH	CLI / CIIII	ist gut die ratten, meüss und dergleichen unzifer zu toedten und vertreiben / und den mucken fürgesetzt . . .
Veratrum album aggr.	Melanthiaceae	ren	LF	Germer	syst	SKE	CLI / CIIII	hüftwee
Veratrum album aggr.	Melanthiaceae	ren	LF	Germer	top	TEE	CLI / CIIII	so legen sie den weetagen [der zeen]
Veratrum album aggr.	Melanthiaceae	s pop	VAL	Germer	syst	CAR	p. 250	Palpitations de la maladie de Basedow, hypertensions artérielle
Veratrum album aggr.	Melanthiaceae	s pop	VAL	Germer	top	CAR	p. 250	Palpitations
Veratrum album aggr.	Melanthiaceae	s pop	VAL	Germer	top	SKE	p. 250	analgésique da la goutte
Verbascum thapsus aggr.	Scrophulariac	ant	DIOS	Königskerze	top	ANT	239	helfen auch gegen Skorpionsbisse
Verbascum thapsus aggr.	Scrophulariac	ant	DIOS	Königskerze	top	ANT	239	helfen auch gegen Skorpionsbisse
Verbascum thapsus aggr.	Scrophulariac	ant	DIOS	Königskerze	top	DER	239	Kataplasma gegen Oedeme; und mit Honig oder Wein gegen brandige Geschwüre. . . . werden als Umschlag bei Verbrennungen gebraucht
Verbascum thapsus aggr.	Scrophulariac	ant	DIOS	Königskerze	top	DER	239	Wunden
Verbascum thapsus aggr.	Scrophulariac	ant	DIOS	Königskerze	top	EYE	239	Augenentzündungen
Verbascum thapsus aggr.	Scrophulariac	ant	DIOS	Königskerze	syst	GAS	239	Die Wurzel der beiden erstgenannten Arten ist adstringirend; deshalb wird sie den an Durchfall Leidenden in der Grösse eines Würfels mit Wein vortheilhaft zu trinken gegeben; inneren Rupturen, Krämpfen
Verbascum thapsus aggr.	Scrophulariac	ant	DIOS	Königskerze	n.d.	OTH	239	färbt die Haare
Verbascum thapsus aggr.	Scrophulariac	ant	DIOS	Königskerze	syst	RES	239	chronischem Husten
Verbascum thapsus aggr.	Scrophulariac	ant	DIOS	Königskerze	syst	SKE	239	Quetschungen
Verbascum thapsus aggr.	Scrophulariac	ant	DIOS	Königskerze	top	TEE	239	Mundspülwasser lindert sie Zahnschmerzen
Verbascum thapsus aggr.	Scrophulariac	mon	HvB	Königskerze	syst	CAR	1-123	schwaches und trauriges Herz . . . Es stärkt sein Herz und macht es fröhlich
Verbascum thapsus aggr.	Scrophulariac	mon	HvB	Königskerze	syst	RES	1-123	in Stimme und Kehle heiser, in der Brust Schmerzen
Verbascum thapsus aggr.	Scrophulariac	ren	LF	Königskerze	syst	ANT	XXVIII / CCCXXVIII	gut denen so von einem Scorpion gestochen seind
Verbascum thapsus aggr.	Scrophulariac	ren	LF	Königskerze	top	ANT	XXVIII / CCCXXVIII	seind auch gut denen so von einem Scorpion gestochen seind
Verbascum thapsus aggr.	Scrophulariac	ren	LF	Königskerze	top	DER	XXVIII / CCCXXVIII	machen schoen har
Verbascum thapsus aggr.	Scrophulariac	ren	LF	Königskerze	top	DER	XXVIII / CCCXXVIII	in die geschwaer und wunden gethon heylen sie dieselben
Verbascum thapsus aggr.	Scrophulariac	ren	LF	Königskerze	top	DER	XXVIII / CCCXXVIII	verzeren die kalte geschwulst und andere beulen
Verbascum thapsus aggr.	Scrophulariac	ren	LF	Königskerze	top	DER	XXVIII / CCCXXVIII	heylen den brandt
Verbascum thapsus aggr.	Scrophulariac	ren	LF	Königskerze	syst	DER	XXVIII / CCCXXVIII	so ziehen sie doern und spreissel auss dem leib
Verbascum thapsus aggr.	Scrophulariac	ren	LF	Königskerze	syst	GAS	XXVIII / CCCXXVIII	stellt den bauchfluss
Verbascum thapsus aggr.	Scrophulariac	ren	LF	Königskerze		OTH	XXVIII / CCCXXVIII	wan man feygen über ein jar unversert behalten will
Verbascum thapsus aggr.	Scrophulariac	ren	LF	Königskerze	syst	RES	XXVIII / CCCXXVIII	nützlich . . . Zu allerley gebresten der brust
Verbascum thapsus aggr.	Scrophulariac	ren	LF	Königskerze	syst	RES	XXVIII / CCCXXVIII	item dem langwierigen husten
Verbascum thapsus aggr.	Scrophulariac	ren	LF	Königskerze	syst	SKE	XXVIII / CCCXXVIII	ist gut denen die glider auseinander seind
Verbascum thapsus aggr.	Scrophulariac	ren	LF	Königskerze	syst	SKE	XXVIII / CCCXXVIII	bekompt sie wohl den gebrochnen unnd denen so ettwas im leib zerissen und zerknütschet ist
Verbascum thapsus aggr.	Scrophulariac	ren	LF	Königskerze	top	TEE	XXVIII / CCCXXVIII	im mund warm gehalten lindert die schmerzen der zaen
Verbascum thapsus aggr.	Scrophulariac	s pop	BVA	Königskerze	top	DER	p. 125	Hömorrhoidenknoten, Afterjucken
Verbascum thapsus aggr.	Scrophulariac	s pop	BVA	Königskerze	top	NER	p. 125	Neuraligien
Verbascum thapsus aggr.	Scrophulariac	s pop	BVA	Königskerze	syst	RES	p. 125	Heiserkeit, Kehlkopfkatarrh, Husten, Bronchitis
Verbascum thapsus aggr.	Scrophulariac	s pop	BVA	Königskerze	syst	RES	p. 125	Heiserkeit, Kehlkopfkatarrh, Husten, Bronchitis
Verbascum thapsus aggr.	Scrophulariac	s pop	JK	Königskerze	top	DER	p. 384	Ausschlägen, Geschwüren, Hämorrhoiden
Verbascum thapsus aggr.	Scrophulariac	s pop	JK	Königskerze	syst	GAS	p. 384	Gelbsucht

Verbascum thapsus aggr.	Scrophulariac	s pop	JK	Königskerze	syst	RES	p. 384	Brust- und Lungenleiden
Verbascum thapsus aggr.	Scrophulariac	s pop	JK	Königskerze	syst	URO	p. 384	mühsames Wasserlösen
Verbascum thapsus aggr.	Scrophulariac	s pop	KN	Königskerze	top	DER	p. 492	Gurgelwasser
Verbascum thapsus aggr.	Scrophulariac	s pop	KN	Königskerze	syst	RES	p. 492	Husten, Katarrhe, . . .
Verbascum thapsus aggr.	Scrophulariac	s pop	UB	Königskerze	syst	RES	p. 213	unproduktiver Reizhusten
Verbascum thapsus aggr.	Scrophulariac	s pop	WI	Königskerze	top	DER	p. 609	Volksmedizin: Wundbehandlung
Verbascum thapsus aggr.	Scrophulariac	s pop	WI	Königskerze	syst	SKE	p. 609	Volksmedizin: Antirheumatikum
Verbascum thapsus aggr.	Scrophulariac	sci	EMA	Königskerze	syst	RES	HMPC/395213/2007	Herbal medicinal product traditionally used to relieve symptoms of sore throat associated with dry cough and cold.
Verbena officinalis L.	Verbenaceae	ant	DIOS	Eisenkraut	syst	ANT	p. 226	wirksam gegen Schlangen(bisse).
Verbena officinalis L.	Verbenaceae	ant	DIOS	Eisenkraut	top	DER	p. 226	lindert es roseartige Entzündungen und heilt faulige Geschwüre; ferner verklebt es Wunden und bringt alte mit Honig zur Vernarbung
Verbena officinalis L.	Verbenaceae	ant	DIOS	Eisenkraut	top	DER	p. 226	Langwierige Oedeme und Entzündungen heilen sie als Umschlag, reinigen auch schmutzige Geschwüre
Verbena officinalis L.	Verbenaceae	ant	DIOS	Eisenkraut	top	DER	p. 226	Langwierige Oedeme und Entzündungen heilen sie als Umschlag, reinigen auch schmutzige Geschwüre; Die ganze Pflanze mit Wein gekocht reisst den Schorf auf Mandelgeschwüren ringsum auf und heilt als Gurgelmittel fressende Geschwüre im Munde.
Verbena officinalis L.	Verbenaceae	ant	DIOS	Eisenkraut	syst	FEV	p. 226	Den am dreitägigen Fieber Leidenden wird von der Erde an der dritte Knoten mit den daran stehenden Blättern zu trinken gegeben, den am viertägigen Fieber Leidenden der vierte
Verbena officinalis L.	Verbenaceae	ant	DIOS	Eisenkraut	syst	GAS	p. 226	Bei Gelbsucht werden die Blätter zu 1 Dachme . . .
Verbena officinalis L.	Verbenaceae	ant	DIOS	Eisenkraut	top	GYN	p. 226	als Zäpfchen eingelegt die Schmerzen der Gebärmutter vertreiben
Verbena officinalis L.	Verbenaceae	ant	DIOS	Eisenkraut	other	OTH	p. 226	Man nennt diese Pflanze die heilige, weil sie bei den Sühneopfern als Amulett sehr im Gebrauch ist.
Verbena officinalis L.	Verbenaceae	mon	HvB	Eisenkraut	top	DER	1-154	wenn infolge von Geschwüren oder Würmern fauliges Fleisch im Menschen ist, faulige Wunden
Verbena officinalis L.	Verbenaceae	mon	HvB	Eisenkraut	top	RES	1-154	Kehle anschwillt
Verbena officinalis L.	Verbenaceae	mon	MF	Eisenkraut	syst	ANT	58	Wunde, widersetzt sich sämtlichen Giften
Verbena officinalis L.	Verbenaceae	mon	MF	Eisenkraut	top	DER	58	verderbliche Bisse
Verbena officinalis L.	Verbenaceae	mon	MF	Eisenkraut	top	DER	58	heilt Wunden in der Mundhöhle
Verbena officinalis L.	Verbenaceae	mon	MF	Eisenkraut	top	DER	58	Mumps
Verbena officinalis L.	Verbenaceae	mon	MF	Eisenkraut	syst	FEV	58	Wechselfieber
Verbena officinalis L.	Verbenaceae	mon	MF	Eisenkraut	syst	GAS	58	heilsam für alle eingeweide, für Seiten- und Rippenfellschmerzen, Beschwerden der Leber
Verbena officinalis L.	Verbenaceae	mon	MF	Eisenkraut	syst	GAS	58	nützt den Gelbsüchtigen
Verbena officinalis L.	Verbenaceae	mon	MF	Eisenkraut	vol	NER	58	soll sie allesamt heiteren Sinnes machen
Verbena officinalis L.	Verbenaceae	mon	MF	Eisenkraut	other	OTH	58	Kranz bringt schnelle Heilung
Verbena officinalis L.	Verbenaceae	mon	MF	Eisenkraut	other	OTH	58	Orakel
Verbena officinalis L.	Verbenaceae	mon	MF	Eisenkraut	syst	RES	58	Leiden der Brust, Lungenkrankheiten und Schwindsucht
Verbena officinalis L.	Verbenaceae	mon	MF	Eisenkraut	top	RES	58	Eiterfäule im Mund
Verbena officinalis L.	Verbenaceae	mon	MF	Eisenkraut	syst	URO	58	Steinleidenden
Verbena officinalis L.	Verbenaceae	ren	LF	Eisenkraut	top	ANT	CCXXXVII / CCXXVI	seind trefflich gut wider allerley gifftige thier
Verbena officinalis L.	Verbenaceae	ren	LF	Eisenkraut	syst	DER	CCXXXVII / CCXXVI	die newlich mit dem aussatz seind überfallen worden
Verbena officinalis L.	Verbenaceae	ren	LF	Eisenkraut	top	DER	CCXXXVII / CCXXVI	lindern sie die hitzige geschwulst / reynigen auch unnd seubern die unreynen schaeden / heylet die mundfeule und die geschwaer des munds, die umb sich fressen
Verbena officinalis L.	Verbenaceae	ren	LF	Eisenkraut	top	DER	CCXXXVII / CCXXVI	gut zu dem rotlauff / heylen auch die faulen unnd unreynen schaeden / desgleichen auch die frischen wunden unnd die allten mit honig vermischt
Verbena officinalis L.	Verbenaceae	ren	LF	Eisenkraut	top	DER	CCXXXVII / CCXXVI	seind trefflich gut wider allerley gifftige thier
Verbena officinalis L.	Verbenaceae	ren	LF	Eisenkraut	syst	GAS	CCXXXVII / CCXXVI	denen so die roten rhur haben
Verbena officinalis L.	Verbenaceae	ren	LF	Eisenkraut	syst	GAS	CCXXXVII / CCXXVI	heylen sie die geelsucht
Verbena officinalis L.	Verbenaceae	ren	LF	Eisenkraut	syst	GAS	CCXXXVII / CCXXVI	wider das grimmen der daerm
Verbena officinalis L.	Verbenaceae	ren	LF	Eisenkraut	top	GYN	CCXXXVII / CCXXVI	legen die schmerzen der muter
Verbena officinalis L.	Verbenaceae	ren	LF	Eisenkraut	syst	NER	CCXXXVII / CCXXVI	ist nützlich denen so mit dem fallenden siechtagen beladen seind
Verbena officinalis L.	Verbenaceae	ren	LF	Eisenkraut	top	NER	CCXXXVII / CCXXVI	gut zu den weetagen des haupts
Verbena officinalis L.	Verbenaceae	ren	LF	Eisenkraut	top	NER	CCXXXVII / CCXXVI	verhueten auch das einem die har nit aussfallen
Verbena officinalis L.	Verbenaceae	ren	LF	Eisenkraut	top	NER	CCXXXVII / CCXXVI	gut zu den weetagen des haupts
Verbena officinalis L.	Verbenaceae	ren	LF	Eisenkraut		OTH	CCXXXVII / CCXXVI	den saal oder das gemach darin man isset darmit besprengt sol die gest froelich machen
Verbena officinalis L.	Verbenaceae	ren	LF	Eisenkraut	top	SKE	CCXXXVII / CCXXVI	podagram, weetagen der hüfft und die wunden
Verbena officinalis L.	Verbenaceae	ren	LF	Eisenkraut	top	TEE	CCXXXVII / CCXXVI	mildert die weetage der zaen, macht dieselbigen fest unnd steiff und heylet die geschwaer des munds
Verbena officinalis L.	Verbenaceae	ren	LF	Eisenkraut	syst	URO	CCXXXVII / CCXXVI	es treibt auch den stein
Verbena officinalis L.	Verbenaceae	s pop	JK	Eisenkraut	top	DER	p. 346	Hunden, Schlangen oder tollwütigen Menschen gebissen
Verbena officinalis L.	Verbenaceae	s pop	JK	Eisenkraut	top	DER	p. 346	Haarausfall
Verbena officinalis L.	Verbenaceae	s pop	JK	Eisenkraut	top	DER	p. 346	Hundebiss und Frostbeulen, böse Flechten
Verbena officinalis L.	Verbenaceae	s pop	JK	Eisenkraut	top	DER	p. 346	austretendem Mastdarm, äussere Geschwülste
Verbena officinalis L.	Verbenaceae	s pop	JK	Eisenkraut	top	DER	p. 346	Zahnweh, Mundfäule, entzündetem Zahnfleisch, Heiserkeit und Mandelgeschwür
Verbena officinalis L.	Verbenaceae	s pop	JK	Eisenkraut	top	EAR	p. 346	Ohrenweh
Verbena officinalis L.	Verbenaceae	s pop	JK	Eisenkraut	top	EYE	p. 346	Augenweh, triefenden Augen, benimmt das Flimmern und macht klare Augen
Verbena officinalis L.	Verbenaceae	s pop	JK	Eisenkraut	syst	GAS	p. 346	Gelbsucht, Gallensteine
Verbena officinalis L.	Verbenaceae	s pop	JK	Eisenkraut	top	GAS	p. 346	Gallenleiden
Verbena officinalis L.	Verbenaceae	s pop	JK	Eisenkraut	syst	NER	p. 346	Nervenschmerzen, Fallend Weh
Verbena officinalis L.	Verbenaceae	s pop	JK	Eisenkraut	top	NER	p. 346	stillt sinnliche Erregungszustände
Verbena officinalis L.	Verbenaceae	s pop	JK	Eisenkraut	top	NER	p. 346	Kopfschmerzen, Nervenschmerzen
Verbena officinalis L.	Verbenaceae	s pop	JK	Eisenkraut	syst	RES	p. 346	Gebrechen des Kopfes, die von Verkältungen her kommen, Lungenaffektionen, Husten, Lungenleiden, Astmah, Grippe
Verbena officinalis L.	Verbenaceae	s pop	JK	Eisenkraut	top	SKE	p. 346	Ischias und Rheumatismus
Verbena officinalis L.	Verbenaceae	s pop	JK	Eisenkraut	top	TEE	p. 346	Zahnweh
Verbena officinalis L.	Verbenaceae	s pop	JK	Eisenkraut	syst	URO	p. 346	Nieren- Blasensteine
Verbena officinalis L.	Verbenaceae	s pop	JK	Eisenkraut	top	URO	p. 346	Nieren- und Milzleiden
Verbena officinalis L.	Verbenaceae	s pop	UB	Eisenkraut	syst	CAR	p. 376	funktionelle Herzbeschwerden
Verbena officinalis L.	Verbenaceae	s pop	WI	Eisenkraut	syst	GYN	p. 612	Volksmedizin:Galaktagogum
Verbena officinalis L.	Verbenaceae	s pop	WI	Eisenkraut	syst	SKE	p. 612	Volksmedizin: Antirheumatikum
Vinca minor L.	Apocynaceae	ant	DIOS	Immergrün	syst	ANT	p. 214	die von der Aspisschlange Gebissenen heilen
Vinca minor L.	Apocynaceae	ant	DIOS	Immergrün	top	ANT	p. 214	helfen aufgelegt beim Biss giftiger Thiere
Vinca minor L.	Apocynaceae	ant	DIOS	Immergrün	syst	GAS	p. 214	Ihre Blätter und Stengel, mit Wein getrunken, beschwichtigen Durchfall und Dysenterie
Vinca minor L.	Apocynaceae	ant	DIOS	Immergrün	top	GYN	p. 214	mit Milch und Rosen- oder Cyperngrassalbe in Zäpfchen eingelegt heilen sie Gebärmutterleiden
Vinca minor L.	Apocynaceae	ant	DIOS	Immergrün	top	TEE	p. 214	Zahnschmerzen
Vinca minor L.	Apocynaceae	mon	LO	Immergrün	n.d.	OTH	n.d.	n.d.
Vinca minor L.	Apocynaceae	ren	LF	Immergrün	syst	DER	CCIII / CXXXV	stellt das bluten der nasen
Vinca minor L.	Apocynaceae	ren	LF	Immergrün	syst	DER	CCIII / CXXXV	ist fürtzrefflich gut denen so blut speien oder ausspürtzen
Vinca minor L.	Apocynaceae	ren	LF	Immergrün	top	DER	CCIII / CXXXV	heylet es dieselbigen [biss der gifftigen thier]
Vinca minor L.	Apocynaceae	ren	LF	Immergrün	syst	GAS	CCIII / CXXXV	stellet den bauchfluss und die roten rhur
Vinca minor L.	Apocynaceae	ren	LF	Immergrün	top	GYN	CCIII / CXXXV	legt den schmerzen derselbigen [muter]
Vinca minor L.	Apocynaceae	ren	LF	Immergrün	top	TEE	CCIII / CXXXV	so legt des den schmerzen der zaen
Vinca minor L.	Apocynaceae	s pop	BVA	Kleines Immergrün	syst	CAR	p. 107	Arteriosklerose, Alterserscheinungnen als Geriatricum, zerebralen Durchlbutungsstörungen, Durchblutungsstörungen im Gehirn, Augen und Innenohr, Schwindel, Konzentrationsschwäche, Kopfschmerzen im Alter, Ohrensausen, Bluthochdruck
Vinca minor L.	Apocynaceae	s pop	BVA	Kleines Immergrün	syst	CAR	p. 107	Arteriosklerose, Alterserscheinungnen als Geriatricum, zerebralen Durchlbutungsstörungen, Durchblutungsstörungen im Gehirn, Augen und Innenohr, Schwindel, Konzentrationsschwäche, Kopfschmerzen im Alter, Ohrensausen, Bluthochdruck
Vinca minor L.	Apocynaceae	s pop	BVA	Kleines Immergrün	syst	EAR	p. 107	Ohrensausen, Altersschwerhörigkeit, Durchblutungsstörungen im Innenohr

Vinca minor L.	Apocynaceae	s pop	BVA	Kleines Immergrün	syst	EAR	p. 107	Ohrensausen, Altersschwerhörigkeit, Durchblutungsstörungen im Innenohr
Vinca minor L.	Apocynaceae	s pop	BVA	Kleines Immergrün	syst	EYE	p. 107	Durchblutungsstörungen im Auge
Vinca minor L.	Apocynaceae	s pop	BVA	Kleines Immergrün	syst	EYE	p. 107	Durchblutungsstörungen im Auge
Viola hirta aggr.	Violaceae	ant	DIOS	Veilchen	syst	EYE	p. 243	bei Augenentzündung
Viola hirta aggr.	Violaceae	ant	DIOS	Veilchen	top	EYE	p. 243	bei Augenentzündung
Viola hirta aggr.	Violaceae	ant	DIOS	Veilchen	syst	GAS	p. 243	Erhitzung des Magens, [bei Augenentzündung] und Mastdarmvorfall.
Viola hirta aggr.	Violaceae	ant	DIOS	Veilchen	top	GAS	p. 243	Erhitzung des Magens, bei [Augenentzündung] und Mastdarmvorfall.
Viola hirta aggr.	Violaceae	ant	DIOS	Veilchen	syst	NER	p. 243	Epilepsie der Kinder
Viola hirta aggr.	Violaceae	ant	DIOS	Veilchen	syst	SKE	p. 243	bei Schlundmuskelentzündung
Viola hirta aggr.	Violaceae	mon	HvB	Veilchen	top	DER	1-103	Wessen Fleisch die Krebse zerfressen, oder irgendwelche Geschwüre in seinem Körper
Viola hirta aggr.	Violaceae	mon	HvB	Veilchen	top	DER	1-103	Krebs und andere Würmer den Menschen zerfressen
Viola hirta aggr.	Violaceae	mon	HvB	Veilchen	top	EYE	1-103	vertreibt die Verdunkelung der Augen
Viola hirta aggr.	Violaceae	mon	HvB	Veilchen	top	EYE	1-103	feurige Augen und in ihnen verdunkelt ist
Viola hirta aggr.	Violaceae	mon	HvB	Veilchen	syst	FEV	1-103	dreitägigem Fieber
Viola hirta aggr.	Violaceae	mon	HvB	Veilchen	top	NER	1-103	Kopfweh
Viola hirta aggr.	Violaceae	mon	HvB	Veilchen	top	NER	1-103	Schwere im Kopf oder in den Nieren, oder der irgendwo von einer Lähmung geplagt wird
Viola hirta aggr.	Violaceae	mon	MF	Veilchen	top	DER	40	fressende Kopfgeschwüre heilen
Viola hirta aggr.	Violaceae	mon	MF	Veilchen	syst	DER	40	Halsgeschwüre
Viola hirta aggr.	Violaceae	mon	MF	Veilchen	top	DER	40	Blatterbläschen
Viola hirta aggr.	Violaceae	mon	MF	Veilchen	top	DER	40	Schrunden im After
Viola hirta aggr.	Violaceae	mon	MF	Veilchen	top	DER	40	Schuppen oder Kleie auf dem Kopf kannst du mit diesem Öl vertreiben.
Viola hirta aggr.	Violaceae	mon	MF	Veilchen	top	DER	40	lindert entzündete Körperstellen
Viola hirta aggr.	Violaceae	mon	MF	Veilchen	syst	DER	40	stillt eine unregelmässig blutende Wunde
Viola hirta aggr.	Violaceae	mon	MF	Veilchen	top	EAR	40	hilft gegen Ohrenklingen und Schmerz
Viola hirta aggr.	Violaceae	mon	MF	Veilchen	top	EYE	40	entzündete Augen
Viola hirta aggr.	Violaceae	mon	MF	Veilchen	syst	GAS	40	tötet es Spulwürmer
Viola hirta aggr.	Violaceae	mon	MF	Veilchen	top	GYN	40	Geschwulst der Gebärmutter
Viola hirta aggr.	Violaceae	mon	MF	Veilchen	syst	GYN	40	führen geordneten Monatsfluss herbei
Viola hirta aggr.	Violaceae	mon	MF	Veilchen	vol	NER	40	dem, der getrunken hat und nun am Rausch leidet
Viola hirta aggr.	Violaceae	mon	MF	Veilchen	syst	NER	40	fallsüchtige Knaben heilen
Viola hirta aggr.	Violaceae	mon	MF	Veilchen	top	NER	40	nützt auch dem Haupte allgemein, an welchem Schmerz es immer leiden mag . . . . Den Körper in Schlummer löst
Viola hirta aggr.	Violaceae	mon	MF	Veilchen	top	OTH	40	trocknen die Milz, wenn man die Mischung trinkt oder der Milz auflegt
Viola hirta aggr.	Violaceae	mon	MF	Veilchen	syst	RES	40	Kinderhusten sowie gegen das Keuchen
Viola hirta aggr.	Violaceae	mon	MF	Veilchen	top	SKE	40	Schädelknochen durch einen Schlag eingedellt, so dass der Betroffenen die Sprache verloren hat, . . .
Viola hirta aggr.	Violaceae	mon	MF	Veilchen	top	SKE	40	Fussgicht zur Milde zwingen
Viola hirta aggr.	Violaceae	ren	LF	Veilchen	syst	DER	CLXXIIII / CXVII	hals geschwaeren oder geschwulsten
Viola hirta aggr.	Violaceae	ren	LF	Veilchen	top	DER	CLXXIIII / CXVII	gut denen so der hinder heraus geet
Viola hirta aggr.	Violaceae	ren	LF	Veilchen	top	EYE	CLXXIIII / CXVII	unnd [hitzigen] augen
Viola hirta aggr.	Violaceae	ren	LF	Veilchen	syst	FEV	CLXXIIII / CXVII	lescht aus unnd dempfft die scherpffe der gallen und die übrige hitz des febers
Viola hirta aggr.	Violaceae	ren	LF	Veilchen	syst	GAS	CLXXIIII / CXVII	linderung des stulgangs
Viola hirta aggr.	Violaceae	ren	LF	Veilchen	top	GAS	CLXXIIII / CXVII	gut dem hitzigen magen
Viola hirta aggr.	Violaceae	ren	LF	Veilchen	syst	NER	CLXXIIII / CXVII	und den jungen kindern so mit der fallenden sucht belaen seind
Viola hirta aggr.	Violaceae	ren	LF	Veilchen	syst	NER	CLXXIIII / CXVII	schmerzen des haupts so vin hitz und trückne kompt
Viola hirta aggr.	Violaceae	ren	LF	Veilchen	vol	NER	CLXXIIII / CXVII	sie machen schlafen so man daran reucht
Viola hirta aggr.	Violaceae	ren	LF	Veilchen	syst	OTH	CLXXIIII / CXVII	leschung des dursts
Viola hirta aggr.	Violaceae	ren	LF	Veilchen	n.d.	OTH	CLXXIIII / CXVII	ganz widerwertig den scorpionen
Viola hirta aggr.	Violaceae	ren	LF	Veilchen	syst	RES	CLXXIIII / CXVII	[linderung] der rauhen kelen
Viola hirta aggr.	Violaceae	ren	LF	Veilchen	syst	RES	CLXXIIII / CXVII	ist gut zu dem trucknen husten
Viola hirta aggr.	Violaceae	s pop	JK	Veilchen	syst	GAS	p. 429	Brechreiz förderndes Mittel
Viola hirta aggr.	Violaceae	s pop	JK	Veilchen	syst	RES	p. 429	Husten und Katarrh
Viola hirta aggr.	Violaceae	s pop	JK	Veilchen	syst	URO	p. 429	Nieren- und Blasenentzündung
Viola hirta aggr.	Violaceae	s pop	KN	Veilchen	top	DER	p. 487	erhitzte Geschwülste
Viola hirta aggr.	Violaceae	s pop	KN	Veilchen	top	DER	p. 487	Angina, probates Gurgelwasser
Viola hirta aggr.	Violaceae	s pop	KN	Veilchen	syst	NER	p. 487	Kopfweh und grosser Hitze im Kopf
Viola hirta aggr.	Violaceae	s pop	KN	Veilchen	syst	RES	p. 487	Husten oder Keuchhusten, Schwindsüchtigen lindert er den Husten
Viola hirta aggr.	Violaceae	s pop	KN	Veilchen	top	SKE	p. 487	Podagra
Viola hirta aggr.	Violaceae	s pop	UB	Veilchen	syst	RES	p. 223	Auswurfförderung
Viola hirta aggr.	Violaceae	sci	ESCOP	Violae herba cum Fl	syst	DER	II p. 280	skin disorders such as eczema, seborrhoea, impetigo and acne, as well as craddle cap and nappy rash of infants
Viola hirta aggr.	Violaceae	sci	ESCOP	Violae herba cum Fl	top	DER	II p. 280	skin disorders such as eczema, seborrhoea, impetigo and acne, as well as craddle cap and nappy rash of infants
Vitis vinifera aggr.	Vitaceae	ant	DIOS	Weinrebe	n.d.	ANT	p. 264	Die Trester endlich helfen bei [Verrenkungen], Schlangenbiss [und Milzentzündungen], wenn sie mit Rosenöl, Raute und Essig angewandt werden.
Vitis vinifera aggr.	Vitaceae	ant	DIOS	Weinrebe	top	DER	p. 264	heilt die am After gebildeten Geschwülste und Feig- warzen
Vitis vinifera aggr.	Vitaceae	ant	DIOS	Weinrebe	top	DER	p. 264	Eingestrichen heilt sie auch Flechten, Krätze und Aussatz, man muss aber vorher die Stelle mit Natron reinigen; Mit Oel dauernd eingesalbt vertreibt sie die Haare, besonders (thut dies) auch die aus den angebrannten Zweigen ausschwitzende Flüssigkeit; aufgestrichen bringt diese auch Warzen weg
Vitis vinifera aggr.	Vitaceae	ant	DIOS	Weinrebe	syst	GAS	p. 264	bei Dysenterie, Blut- auswurf, Magenschmerzen und falschem Appetit schwangerer Frauen
Vitis vinifera aggr.	Vitaceae	ant	DIOS	Weinrebe	top	GAS	p. 264	mit Graupen Entzündung und Brand des Magens
Vitis vinifera aggr.	Vitaceae	ant	DIOS	Weinrebe	syst	GAS	p. 264	zertrümmert den Stein, wenn sie mit Wein genommen wird
Vitis vinifera aggr.	Vitaceae	ant	DIOS	Weinrebe	top	NER	p. 264	fein gestossen als Umschlag, lindern Kopfschmerzen
Vitis vinifera aggr.	Vitaceae	ant	DIOS	Weinrebe	n.d.	OTH	p. 264	Die Trester endlich helfen bei [Verrenkungen, Schlangenbiss und] Milzentzündungen, wenn sie mit Rosenöl, Raute und Essig angewandt werden.
Vitis vinifera aggr.	Vitaceae	ant	DIOS	Weinrebe	n.d.	SKE	p. 264	Die Trester endlich helfen bei Verrenkungen, [Schlangenbiss und Milzentzündungen], wenn sie mit Rosenöl, Raute und Essig angewandt werden.
Vitis vinifera aggr.	Vitaceae	mon	HvB	Weinrebe	top	DER	3-54	Geschwüre, Wunden
Vitis vinifera aggr.	Vitaceae	mon	HvB	Weinrebe	top	EAR	3-54	Ohrenschmerzen
Vitis vinifera aggr.	Vitaceae	mon	HvB	Weinrebe	top	EYE	3-54	trübe Augen
Vitis vinifera aggr.	Vitaceae	mon	HvB	Weinrebe	syst	GAS	3-54	Schmerzen im Magen
Vitis vinifera aggr.	Vitaceae	mon	HvB	Weinrebe	top	NER	3-54	Kopfschmerzen
Vitis vinifera aggr.	Vitaceae	mon	HvB	Weinrebe	syst	RES	3-54	Schmerzen in der Brus
Vitis vinifera aggr.	Vitaceae	mon	HvB	Weinrebe	top	TEE	3-54	Fleisch um die Zähne fault, Zähne schwach sind
Vitis vinifera aggr.	Vitaceae	mon	HvB	Weinrebe	syst	URO	3-54	Harn wegen Kält im Magen nicht halten kann
Vitis vinifera aggr.	Vitaceae	ren	LF	Weinrebe	top	DER	XLVI / XXIX	heylet es geflecht, rauden unnd allerley grind
Vitis vinifera aggr.	Vitaceae	ren	LF	Weinrebe	top	DER	XLVI / XXIX	heylet die blatter unnd runtzel am hindern
Vitis vinifera aggr.	Vitaceae	ren	LF	Weinrebe	vol	DER	XLVI / XXIX	vertreibt das har unnd macht glatt
Vitis vinifera aggr.	Vitaceae	ren	LF	Weinrebe	top	EYE	XLVI / XXIX	ist auch gut den augen
Vitis vinifera aggr.	Vitaceae	ren	LF	Weinrebe	syst	GAS	XLVI / XXIX	seind sie dem magen dienstlich, bringen lust zu essen unnd krefftigen die schwachen
Vitis vinifera aggr.	Vitaceae	ren	LF	Weinrebe	syst	GAS	XLVI / XXIX	seind dem ziehen zusammen unnd seind dem magen angenem
Vitis vinifera aggr.	Vitaceae	ren	LF	Weinrebe	syst	GAS	XLVI / XXIX	rote rhur unnd den bauchfluss haben
Vitis vinifera aggr.	Vitaceae	ren	LF	Weinrebe	top	GAS	XLVI / XXIX	roten rhur und kein speis behalten
Vitis vinifera aggr.	Vitaceae	ren	LF	Weinrebe	syst	GAS	XLVI / XXIX	die roten rhur haben, blut auspürtzenunnd einen schwachen magen haben / un den weibern die seltzam ding zu essen gelüstet
Vitis vinifera aggr.	Vitaceae	ren	LF	Weinrebe	top	GAS	XLVI / XXIX	miltern die entzündung unnd die übrige hitz des magens

Vitis vinifera aggr.	Vitaceae	ren	LF	Weinrebe	syst	GAS	XLVI / XXIX	haben sie gleiche krafft unnd würckung [die roten rhur haben, blut auspürtzenunnd einen schwachen magen haben / un den weibern die seltzam ding zu essen gelüstet]
Vitis vinifera aggr.	Vitaceae	ren	LF	Weinrebe	syst	GYN	XLVI / XXIX	stellen auch der weiber bloedigkeit
Vitis vinifera aggr.	Vitaceae	ren	LF	Weinrebe	top	NER	XLVI / XXIX	ziehen die zaehen kalten feüchtigkeyten an sich unnd reynigen das haubt
Vitis vinifera aggr.	Vitaceae	ren	LF	Weinrebe	top	NER	XLVI / XXIX	lindern die weetagen des haubts
Vitis vinifera aggr.	Vitaceae	ren	LF	Weinrebe	n.d.	RES	XLVI / XXIX	dienstlich dem husten
Vitis vinifera aggr.	Vitaceae	ren	LF	Weinrebe	top	SKE	XLVI / XXIX	gut den verruckten gliedern
Vitis vinifera aggr.	Vitaceae	ren	LF	Weinrebe	n.d.	URO	XLVI / XXIX	[dienstlich] nieren und blasen
Vitis vinifera aggr.	Vitaceae	ren	LF	Weinrebe	syst	URO	XLVI / XXIX	treibt den stein
Vitis vinifera aggr.	Vitaceae	s pop	UB	Weinrebe	syst	CAR	p. 410	Venöse Insuffizienz
Vitis vinifera aggr.	Vitaceae	sci	EMA	Weinrebe	top	DER	A/HPMC/5816/2010	discomfort and heaviness of legs releated to minor venous circulatory disturbances
Vitis vinifera aggr.	Vitaceae	sci	ESCOP	Vitis viniferae folium	syst	DER	I p. 285	symptomatic treatment of problems releated to varicosis such as painful and heavy legs
Vitis vinifera aggr.	Vitaceae	sci	ESCOP	Vitis viniferae folium	top	DER	I p. 285	topical treatment of varicosis and couperosis

Abbreviations:
era and herbal cf. Table 1, maintext
use-categories cf. Table 2, maintext
